# Comprehensive analysis of pathogen-responsive wheat NAC transcription factors: new candidates for crop improvement

**DOI:** 10.1093/g3journal/jkac247

**Published:** 2022-09-21

**Authors:** Monika Vranic, Alexandre Perochon, Harriet Benbow, Fiona M Doohan

**Affiliations:** UCD School of Biology and Environmental Science and Earth Institute, College of Science, University College Dublin, Dublin 4, Ireland; UCD School of Biology and Environmental Science and Earth Institute, College of Science, University College Dublin, Dublin 4, Ireland; UCD School of Biology and Environmental Science and Earth Institute, College of Science, University College Dublin, Dublin 4, Ireland; UCD School of Biology and Environmental Science and Earth Institute, College of Science, University College Dublin, Dublin 4, Ireland

**Keywords:** wheat, NAC, transcription factor, phylogeny, pathogen

## Abstract

Wheat NAC (TaNAC) transcription factors are important regulators of stress responses and developmental processes. This study proposes a new *TaNAC* nomenclature and identified defense-associated *TaNAC*s based on the analysis of RNA-sequencing datasets of wheat tissue infected with major fungal pathogens. A total of 146 *TaNAC*s were pathogen-responsive, of which 52 were orthologous with functionally characterized defense-associated *NAC*s from barley, rice, and *Arabidopsis*, as deduced via phylogenetic analysis. Next, we focused on the phylogenetic relationship of the pathogen-responsive *TaNAC*s and their expression profiles in healthy and diseased tissues. Three subfamilies (“a,” “e,” and “f”) were significantly enriched in pathogen-responsive *TaNAC*s, of which the majority were responsive to at least 2 pathogens (universal pathogen response). Uncharacterized *TaNAC*s from subfamily “a” enriched with defense-associated *NAC*s are promising candidates for functional characterization in pathogen defense. In general, pathogen-responsive *TaNAC*s were expressed in at least 2 healthy organs. Lastly, we showed that the wheat NAM domain is significantly divergent in sequence in subfamilies “f,” “g,” and “h” based on HMMER and motif analysis. New protein motifs were identified in both the N- and C-terminal parts of TaNACs. Three of those identified in the C-terminal part were linked to pathogen responsiveness of the *TaNAC*s and 2 were linked to expression in grain tissue. Future studies should benefit from this comprehensive in silico analysis of pathogen-responsive *TaNAC*s as a basis for selecting the most promising candidates for functional validation and crop improvement.

## Introduction

An estimated 21.5% of global wheat yield is lost due to pests and pathogens (Savary *et al.* 2019). Breeding for resistance is an environmentally friendly and sustainable method to control diseases and an important component of an integrated disease management strategy. Recent advances in sequencing, bioinformatics, and data analytics have delivered the genomic and transcriptomic tools to expedite breeding via the identification of genes and genetic loci underpinning critical agronomic traits in wheat, including disease resistance [International Wheat Genome Sequencing Consortium (IWGSC) *et al.* 2018; Ramírez-González *et al.* 2018]. Transcription factor (TF) families regulate gene expression and are thus master regulators of cellular events. Many plant TF families involved in biotic stress responses have been identified. The NAC [no apical meristem (NAM), *Arabidopsis thaliana* transcription activation factor (ATAF1/2), and cup-shaped cotyledon (CUC2)] TFs represent one of the largest plant families of transcriptional regulators. They are delineated by their conserved NAC domains containing 5 subdomains A–E, of which A–D form the NAM domain (Ooka *et al.* 2003). The NAC domains are usually situated in the N-terminal (NT) part of the protein and are associated with DNA binding, transcriptional control, and homo- and heterodimerization (Welner *et al.* 2016). NAC proteins also encode a highly divergent transcriptional activation region (TAR) in the C-terminal (CT) part of the protein, associated with transcriptional activation and protein–protein interactions (Welner *et al.* 2016). NACs have been known to regulate developmental processes and stress response traits, including disease resistance (Xie *et al.* 2000; Jensen *et al.* 2007; Wu *et al.* 2009). NACs have been shown to play important roles in plant defense against pathogens with diverse lifestyles, via the regulation of plant immunity, modulation of the hypersensitive response, stomatal immunity, involvement in hormonal signaling pathways, and as targets of pathogen effectors (Yuan *et al.* 2019). Cross-talk between NAC proteins, phytohormones, and other signaling molecules, such as reactive oxygen species were shown to enhance or suppress the host response to pathogens (Yuan *et al.* 2019). However, the signaling pathways regulating NAC responses to pathogens remain elusive. The overexpression or suppression of *NAC*s often leads to significantly improved resistance or tolerance to plant pathogens, making them promising candidates for improvement of disease resistance in crops (Yuan *et al.* 2019).

Four studies identified high confidence *Triticum aestivum* (wheat) *NAC*s (*TaNAC*s) in the cultivar Chinese Spring; Borrill *et al.* (2017) identified 453 *TaNAC* genes in the TGACv1 genome assembly, while Guérin *et al.* (2019) and Ma *et al.* (2021) identified, respectively, 460 and 462 *TaNAC*s using a more recent assembly (RefSeq v1.0) and Lv *et al.* (2020) identified 460 *TaNAC*s in the RefSeq v1.1 genome annotation. Studies from Borrill *et al.* (2017) and Guérin *et al.* (2019) mined RNA-sequencing (RNA-seq) datasets to determine the expression profiles of *TaNAC*s in different tissues, and in response to biotic and/or abiotic stresses. Borrill *et al.* (2017) delineated *TaNAC*s responsive to biotic stress, while other studies (Lv *et al.* 2020; Ma *et al.* 2021) delineated *TaNAC* genes responsive to specific pathogens, i.e. races of the causal agents of stripe rust disease (*Puccinia striiformis* f. sp. *tritici*) and powdery mildew disease (*Blumeria graminis* f. sp. *tritici*) in the rust and mildew resistant wheat line N9134*.*Lv *et al.* (2020) highlighted the significantly higher proportion of transcript variants of fungus-responsive *TaNAC*s compared to nonresponsive *TaNAC*s. Ma *et al.* (2021) concluded that the neofunctionalization of *TaNAC*s plays a role in wheat adaptation to biotic stresses.

The aim of the current study was to go further than previous studies to (1) explore the phylogenetic relationship between all wheat *TaNAC*s and well-characterized *NAC*s from other plants and (2) delineate all high confidence *TaNAC*s responsive to pathogens with diverse lifestyles and to explore their phylogenetic relationship. This study determined whether there were any associations between the pathogen responsiveness of *TaNAC*s (within the wheat genome version RefSeqv1.1) and either the lifestyle of the pathogen, phylogeny, or divergence in their NAC subdomain structures or motif composition. A joined phylogenetic tree of NACs from monocots wheat, barley, rice, and the model dicot *Arabidopsis* was constructed to identify *TaNAC* orthologs closely related to characterized *NAC* genes involved in disease resistance. This study identified *TaNAC*s responsive to a diverse set of diseases; 5 publicly available RNA-seq datasets were mined to identify *TaNAC*s associated with *Fusarium* head blight (FHB), *Fusarium* crown rot, *Septoria tritici* blotch, powdery mildew, and stripe rust diseases. Focusing on the pathogen-responsive *TaNAC*s, we studied their phylogenetic relationship, and their expression profiles in healthy and diseased tissue. In addition, in-depth motif analysis of full-length TaNAC amino acid sequences was done to investigate the divergence and structural diversity of TaNAC proteins and the potential association between the pathogen-responsiveness of *TaNAC*s and their protein motif composition.

## Materials and methods

### Identification of *NAC* genes in *T. aestivum*, *Hordeum vulgare*, *Oryza sativa* subsp. *japonica*, and *A. thaliana*

High confidence wheat proteome sequences were downloaded from the IWGSC sequence repository (IWGSC RefSeq v1.1, https://wheat-urgi.versailles.inra.fr/Seq-Repository/Annotations, July 2017). Proteomes for *Arabidopsis*, rice IRGSP 1.0, and barley proteomes (splice variants from longest coding DNA sequences) were downloaded from the TAIR10 sequence repository (https://www.arabidopsis.org, June 2018) and Ensembl Plants release 37 (http://plants.ensembl.org, June 2018). The full alignment of the NAM domain (PF02365) was downloaded from the Pfam database (http://pfam.xfam.org/, August 2017) and used to build Hidden Markov Model (HMM) profile with *hmmbuild* in HMMER v.3.1 b2 (http://hmmer.org/). The HMM profile of the NAM family was then used as a query against wheat, barley, rice, and *Arabidopsis* proteomes with *hmmsearch* (default parameters; HMMER v.3.1 b2, http://hmmer.org/) and all nonredundant hits (*E* < 0.01) were retrieved (Supplementary Table 1).

### Phylogenetic analysis

Multiple sequence alignment (MSA) of the full-length protein sequences was performed using MAFFT v7.407 (Katoh and Standley 2013) and the default parameters. The completeness of MSA was evaluated using AliStat v1.3 (Wong *et al.* 2020). Sequences that did not align with any of the sequences in the MSA and whose completeness score was *C_ij_* < 0.5 (Wong *et al.* 2020) were removed from the MSA used for joined phylogenetic tree (Supplementary Table 2). The MSA was masked in AliStat v1.3 (Wong *et al.* 2020) with a cutoff *C_c_* = 0.6 (Wong *et al.* 2020), and manually inspected and corrected in Jalview v2.10.5 (Waterhouse *et al.* 2009). Identical masked sequences were removed to lower the sample size. Homo v1.3 (Rouse *et al.* 2013) confirmed that pairs of sequences in the MSA were consistent with evolution under stationary, reversible, and globally homogeneous conditions. ModelFinder (Kalyaanamoorthy *et al.* 2017) identified the best substitution model according to the lowest Bayesian information criterion. The best fitting substitution model used to infer joined phylogeny was Jones–Taylor–Thornton (JTT; Jones *et al.* 1992) with 7 categories of probability distribution free (PDF) model of rate heterogeneity across sites (RHAS; Kalyaanamoorthy *et al.* 2017), and JTT with 6 categories of PDF model of RHAS for phylogeny of TaNAC sequences. IQ-TREE (Nguyen *et al.* 2015) was used to estimate maximum likelihood phylogeny with an ascertainment bias correction used to correct for likelihood conditioned solely on variable sites (Lewis 2001). The goodness of fit of branch nodes with phylogenetic trees was estimated by ultrafast bootstrap approximation (UFBoot; Hoang *et al.* 2018) with 1,000 bootstrap alignments. After the tree inference, protein identifiers (IDs) of surplus sequences were added alongside the ID of its identical pair in the joined phylogenetic tree. The resulting trees were unrooted, although outgroup taxon TaNAC121-A1 (TraesCS1A02G190100.1) was randomly selected as a root in IQ-TREE. The joined phylogenetic tree was rerooted and subfamily “b” was selected as a root. A consensus tree was used for further analysis due to a higher maximum likelihood than the true tree. Tree figures were created in iTOL (https://itol.embl.de/; Letunic and Bork 2019).

### Wheat *NAC* naming system

A naming system of *TaNAC* was defined, taking into account *NAC* rice nomenclature and their phylogenetic relationship, paralogy, gene tree and subgenome location, as described in detail in Schilling *et al.* (2020), with a few modifications. *TaNAC* homoeologs, paralogs, and rice orthologs were downloaded from Ensembl Plants (Biomart, release 45 of Ensembl Plants). The 145 rice *NAC* MSU IDs identified in Shen *et al.* (2009) were converted into RAP-DB IDs with ID converter on https://rapdb.dna.affrc.go.jp/tools/converter/run (September 2020). From these 145 *O. sativa NAC (OsNAC)* genes with MSU IDs, 4 were converted into 8 RAP-DB *OsNAC*s and 8 were missing in RAP-DB. The RAP-DB *OsNAC*s Os02g0745250, Os04g0508400, and Os12g0135850 not present in MSU DB did not have a name in any of the databases (RAP-DB, CGSNL, Oryzabase), so they were assigned the names *ONAC150*, *ONAC151*, and *ONAC152*, respectively, using the same nomenclature as described in Shen *et al.* (2009) (i.e. using the prefix O instead of the more widely used Os abbreviation for rice), resulting in a total of 144 *OsNAC* genes.

*TaNAC* homoeologs on subgenomes A, B, and D were primarily identified based on their phylogenetic relationship. In the case of *TaNAC* homoeologs orthologous to the rice *ONAC007* and *ONAC038* (Os06g0131700 and Os01g0946200, respectively), this approach was impossible due to either clade complexity and/or where the bootstrap value of the cluster’s nodes was <70%, and thus, the homoeologs were determined according to the Ensembl Plants. *TaNAC* paralogs were determined based on phylogeny and confirmed according to Ensembl Plants. In cases when orthologous clusters comprised multiple *TaNAC* paralogs located on the same chromosome with at least 80% protein identity, the paralogs were considered as duplicate genes (inparalogs) belonging to the same homoeologous group.

*TaNAC* genes were primarily named based on phylogeny to the closest rice ortholog. In cases where *TaNAC*s were orthologous to more than 1 *OsNAC* paralog located on the same chromosome (e.g. *ONAC020* [Os01g0104500] and *ONAC026* [Os01g0393100]), they were named after the first *OsNAC* identified on the chromosome. *TaNAC*s without a rice ortholog were named consecutively according to their position in a tree starting with subfamily “b” and starting with number 153*.* Genes from an “unknown” chromosome U that clustered with 2 putative homoeologs located on other subgenomes were given a common name (e.g. this was the case for *TaNAC158-U1*, *TaNAC158-B1*, and *TaNAC158-D1*).

### NAC subfamily classification

NAC subfamilies a-h in the joined phylogenetic tree of wheat, barley, rice, and *Arabidopsis* were defined based on previous studies of NAC subfamilies from barley (Christiansen *et al.* 2011), rice, and *Arabidopsis* (Shen *et al.* 2009). Unclassified clades were those that either did not cluster with the subfamilies (with BS ≥ 70%) or were unsupported by bootstrap analysis. *TaNAC* sequences were assigned to the same subfamily as their characterized rice, barley, and *Arabidopsis* orthologs in the joined tree, and for those not included in the joined tree, the subfamily was assigned based on their location in the wheat NAC tree (e.g. TraesCS6D02G266000 and TraesCS7A02G152400). The lists of *NAC* sequences and their corresponding subfamilies in wheat, barley, rice, and *Arabidopsis* are given in Supplementary Table 3.

### Gene expression analysis

Differential expression data from 5 independent RNA-seq studies (Table 1) on fungal diseases of wheat carried out under controlled environmental conditions were obtained from Benbow *et al.* (2019). Genes differentially expressed upon wheat infection with bacterial pathogen *Xanthomonas translucens* were extracted from Expression Atlas release 36 (log2-fold change >1; *P < *0.05) (Table 1). Baseline RNA-seq expression data with transcript per million (tpm) above 0 were downloaded from wheat expression browser (http://wheat-expression.com/, October 2017; Choulet *et al.* 2014), gene-wise normalized, and expression values of developmental stages in 5 organs were averaged (cutoff = 0.1 tpm). IBM SPSS Statistics for Windows v24 (IBM Corp., Armonk, NY, USA) was used to define the expression range as either low (1st quartile, below 0.3 tpm), moderate (2nd and 3rd quartiles, between 0.3 and 3.7 tpm) or high (4th quartile, above 3.7 tpm). Baseline and differential expression data are presented alongside the tree in a heatmap generated using iTOL. Hierarchical clustering with Euclidian distance of the pathogen-responsive *TaNAC*s was done in Genesis v1.8.1 (Sturn *et al.* 2002). Sets of differentially expressed genes were visualized using the IntercatiVenn (http://www.interactivenn.net/, March 2021) web-based tool (Heberle *et al.* 2015). Differential expression data of *HvSNAC1* (HORVU5Hr1G111590) and *ONAC060* (Os12g0610600) during pathogen infection were extracted from Expression Atlas release 36 (https://www.ebi.ac.uk/gxa/home, June 2021).

**Table 1.** RNA-seq experiments used for the analysis of *Triticum aestivum* (bread wheat) *NAC* (*TaNAC*) genes in this study.

Experiment accession	Organ	Treatment	Wheat line/cultivar^a^	Timepoint	Reference
E-MTAB-4289^b^	Leaf	*Puccinia striiformis*	N9134 (R)	1, 2, and 3 dpi	Zhang *et al.* (2014)
E-MTAB-4289^b^	Leaf	*Blumeria graminis*	N9134 (R)	1, 2, and 3 dpi	Zhang *et al.* (2014)
E-MTAB-4222^b^	Spikelet	*Fusarium graminearum*	NIL38 (R), NIL54 (S)	3, 6, 12, 24, 36, and 48 hpi	Schweiger *et al.* (2016)
E-MTAB-4308^b^	Shoot	*F. pseudograminearum*	NIL (R), NIL1S (S)	3 and 5 dpi	Ma *et al.* (2014)
E-MTAB-4470^b^	Leaf	*Zymoseptoria tritici*	cv. Sevin (S)	4, 10, and 13 dpi	Yang *et al.* (2013)
E-MTAB-4116^b^	Leaf	*Z. tritici*	cv. Riband (S)	4, 9, 14, and 21 dpi	Rudd *et al.* (2015)
E-MTAB-5891^b^	Leaf	*Xanthomonas translucens*	cv. Chinese Spring (S)	1 dpi	Garcia-Seco *et al.* (2017)
PRJEB12497^c^	Leaf	*P. striiformis*	cv. Vuka (S)	1 dpi	Dobon *et al*. (2016)
E-MTAB-1729^c^	Spikelet	*F. graminearum*	cv. CM-82036 (R)	50 hpi	Kugler *et al*. (2013)
SRP078208^c^	Coleoptile	*F. pseudograminearum*	cv. Chara (S)	3 dpi	Powell *et al*. (2017)

dpi, days postinoculation; hpi, hours postinoculation.

a R, resistant; S, susceptible; NIL, near isogenic line; cv., cultivar; N9134 is a resistant wheat line carrying *PmAS846* introgressed from wild emmer accession As846 (*Triticum dicoccoides*); NIL38 and NIL54 originate from a cross between cv. CM-82036 and cv. Remus, where NIL38 is homozygous for FHB-resistant alleles at *Fhb1* and *Qhfs.ifa*-*5A* QTL, and NIL51 is homozygous for susceptible alleles at both QTL; NIL1R and NIL1S originate from the population of “Janz”*2/“CSCR6” using the heterogeneous inbred family method for the *Fusarium* crown rot QTL on chromosome arm 3BL (*Qcrs-3B*), where NIL1R carries the resistant allele and NIL1S carries the susceptible allele at the *Qcrs-3B* locus.

b Used in the analysis of the response of all wheat *NAC* genes to the pathogen.

c Used to validate the pathogen-responsiveness of specific *NAC* genes.

Publicly available tpm data from 3 additional RNA-seq studies on wheat infected with *Fusarium graminearum*, *Fusarium pseudograminearum*, and *P. striiformis* (Kugler *et al.* 2013; Dobon *et al.* 2016; Powell *et al.* 2017) (Table 1) were mined for select genes using the Wheat expression browser (http://wheat-expression.com/) and tpm data were analyzed to validate the pathogen-responsiveness of select genes for validation purposes.

### Protein motif analysis

The HMM profile of NAM family proteins (PF02365) was aligned with the TaNAC amino acid sequences using *hmmalign* in HMMER. NAM subdomains (A–D) defined the NT part of the protein, and the remainder was trimmed (manually in Jalview v2.10.5) to extract CT sequences. When sequences had 2 NAM domains, the output given by *hmmsearch* was used to identify the coordinates of the second domain (output gives conditional *E*-values for each domain and their position in a sequence). Identification of motifs in the NT and CT of TaNACs was done separately in MEME v5.0.5 (http://meme-suite.org/, May 2019; Bailey *et al.* 2009). To search motifs in the NT, the parameters were: motif discovery mode—classic, motif site distribution—any number of repetitions, maximum number of motifs—20, and motif width—between 5 and 20 residues. For the CT, the parameters were: motif discovery mode—classic, motif site distribution—zero or 1 site per sequence, maximum number of motifs—40, and motif width—between 5 and 20 residues. Motifs were presented alongside the phylogenetic tree in iTOL. The position of the discovered motifs was compared with positions of subdomains A–E of the NAC domain defined by Ooka *et al.* (2003).

### Statistical analysis

Statistical analysis was performed using IBM SPSS Statistics for Windows v24. The Shapiro–Wilk test was used to evaluate whether the data were normally distributed. The Kruskal–Wallis test with step-down comparison was used to analyze significant differences among distributions of sequence bit scores of subfamilies a-h, and a significance level was *P < *0.05. In addition, Fisher’s exact test (conducted in Microsoft Excel 2011) was used to analyze differences in the proportion of pathogen-responsive *TaNAC*s among subfamilies. The χ^2^ test (conducted in Microsoft Excel 2011) was used to analyze difference in proportions of pathogen-responsive *TaNAC*s within the *TaNAC* family as compared to the proportion of a total number of pathogen-responsive high confidence genes within a wheat genome. The Moses test was used to analyze difference between tpm values in mock sample vs. pathogen-treated samples.

## Results

### Wheat *NAC* nomenclature

We propose a new *T. aestivum* (bread wheat) *TaNAC* nomenclature to simplify and streamline the naming of this gene family within this complex genome. Genes were named based on the relationship with their *O. sativa* (rice) *OsNAC* orthologs and conventional wheat gene nomenclature (McIntosh *et al.* 2013; Schilling *et al.* 2020) (Supplementary Tables 4 and 5). Thus, *TaNAC*s were named according to the name of the phylogenetically closest *OsNAC* ortholog (*TaNACXXX*), their wheat subgenome (A, B, or D), the number of consecutive *TaNAC* singletons or homoeologous genes (1–8) that are orthologs of the *OsNAC* and, if relevant, the number of the consecutive *TaNAC* paralogs (2–5) within the gene cluster (*TaNACXXX-A/B/D* (*1-8*)-(*2-5*)). For example, *TaNAC048-A1* (TraesCS3A02G406000), *TaNAC048-B1* (TraesCS3B02G439600), and *TaNAC048-D1* (TraesCS3D02G401200) are the only triad of homoeologs from A, B, and D wheat subgenomes named after the rice ortholog *ONAC048.* One of the more complex clusters consists of 10 *TaNAC*s that are orthologs of *ONAC066* (Os03g0777000); this includes the subgenome B singleton *TaNAC066-B1* (TraesCS4B02G384700) orthologous to *ONAC066* and 9 *TaNAC*s from subgenomes A, B, and D that form a second homeologous cluster within the *ONAC066* clade and that are inparalogs with >80% ID [e.g. *TaNAC066-A2-1* (TraesCS5A02G411700), *TaNAC066-A2-2* (TraesCS5A02G411800), and *TaNAC066-A2-3* (TraesCS5A02G411900) are 3 consecutive inparalogs residing on chromosome A].

### Phylogenetic relationship of defense-associated wheat, rice, barley, and *Arabidopsis* NACs

A total of 460, 138, 101, and 113 NAC proteins were respectively identified in the genome assemblies of wheat [IWGSC RefSeq v1.1; ∼17 Gb (2*n* = 6*x* = 42)], barley [IBSC v2; ∼5.3 Gb (2*n* = 14)], rice [IRGSP 1.0; ∼500 Mb (2*n* = 24)], and *Arabidopsis* [TAIR 10; ∼135 Mb (2*n* = 10)] (Supplementary Table 6). Although not further analyzed herein, a total of 766 low confidence wheat NAC proteins encoded by a total of 765 low confidence genes were also identified in this study (Supplementary Table 6). Of the 460 wheat high confidence *TaNAC* genes, 413–460 were also identified in previously mentioned studies (Borrill *et al.* 2017; Guérin *et al.* 2019; Lv *et al.* 2020; Ma *et al.* 2021). Notable differences between our results and other studies were (1) the wheat gene TraesCS5B02G271800 identified in Lv *et al.* (2020) was not identified in this study, (2) genes TraesCS5B01G550800 and TraesCS7B01G481500 identified in Ma *et al.* (2021) and Guérin *et al.* (2019) were not identified in this study, and (3) gene *TaNAC118-D4* (TraesCS5D02G537600) from this study was not identified in Lv *et al.* (2020) and Guérin *et al.* (2019) (Supplementary Table 7).

From these 812 wheat, barley, rice, and *Arabidopsis* NACs, the evolutionary relationships of 751 unambiguous nonidentical NAC protein sequences were estimated using a joined maximum-likelihood phylogenetic tree. NACs excluded during tree construction (see Supplementary Table 2) were 21 that had insufficient overlap (*C_ij_* score <0.5) and 40 identical/surplus masked sequences (these were later positioned alongside their identical sequence in the tree). The final high-quality MSA (Supplementary File 1) used for the tree inference had 179 sites/columns and a completeness score (Ca) of 0.88.

**Fig. 1.**
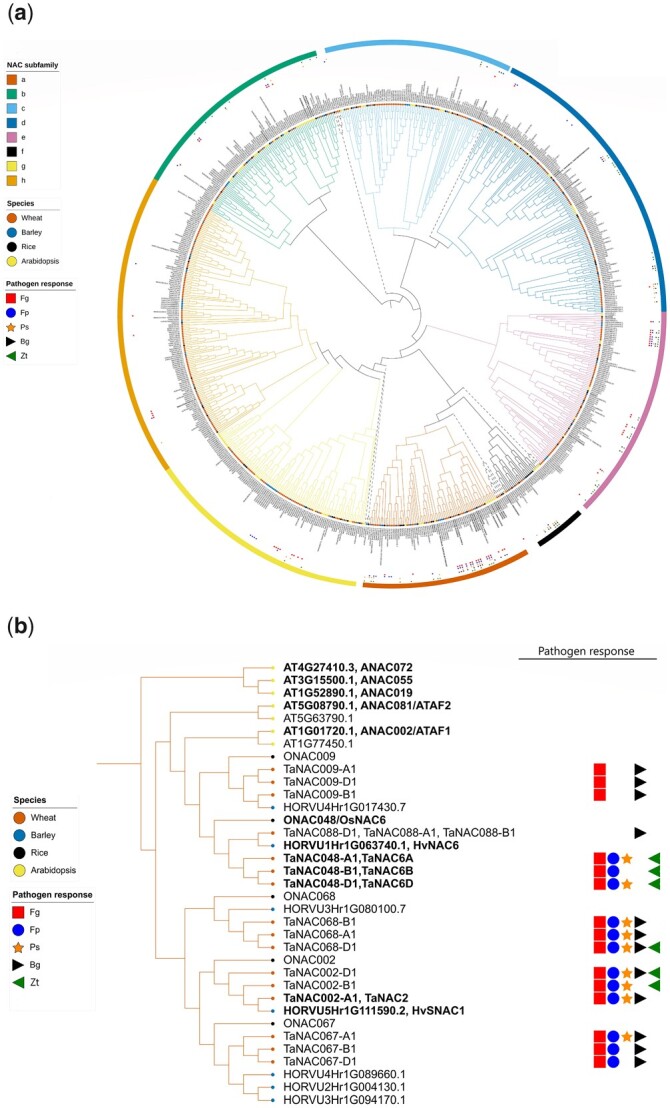
Phylogenetic relationship of NACs in wheat, rice, barley, and *Arabidopsis*. a) Unrooted consensus maximum likelihood joined phylogenetic tree of wheat, barley, rice and *Arabidopsis* NACs (https://itol.embl.de/tree/372282299412371613249495) and b) a close-up of the “*TaNAC2* subclade” of subfamily “a.” Bootstrap scores >50% are indicated on each node in the online version. Branch lines are colored according to NAC subfamily and terminate in a colored circle, which denotes the plant species; alongside the tree, the colored symbols denote the *TaNAC*s that are responsive (at the transcriptome level) to pathogens. All *NAC*s genetically characterized for their role in diseases are highlighted in bold. Identical sequences positioned on the same branch were assigned the pathogen symbol only if all of them were responsive to the specific fungus. Pathogen abbreviations: Fg, *Fusarium graminearum*; Fp, *Fusarium pseudograminearum*; Ps, *Puccinia striiformis*; Bg, *Blumeria graminis*; Zt, *Zymoseptoria tritici*.

As illustrated in Fig. 1a, there are 734 NACs, including 451 TaNACs, distributed in 8 subfamilies (a–h) and 17 unclassified NACs positioned outside of these subfamilies (7 of those were TaNACs)*.* Similarly, 453 *TaNAC*s were grouped in 8 subfamilies in the phylogenetic tree constructed by Borrill *et al.* (2017), and the majority of *TaNAC*s delineated in Borrill *et al.* (2017) and the current study (i.e. 411 *TaNAC*s) were placed in the same subfamily (Supplementary Table 3). Subfamilies “b” and “g” partitioned into 3 individual subclades based on the bootstrap analysis. All studied plants had NACs in all subfamilies (Fig. 1a and Table 2), with 1 subclade within subfamily “g” being *Arabidopsis* specific. Subfamily “d” was the most abundant subfamily in barley and rice with 23 and 20 *NAC*s, respectively, while “b” was the subfamily most expanded in *Arabidopsis* with 33 *NAC*s. Subfamily “h” was the most abundant for wheat and was almost exclusively restricted to monocots (including just 1 *Arabidopsis NAC*) and it is the largest subfamily in the plants we studied. It contained 23% (106), 16% (21), 12% (11), and 1% (1) of the wheat, barley, rice, and *Arabidopsis NAC*s, respectively.

**Table 2.** Number of *NAC* genes in subfamilies a-h in wheat, barley, rice, and *Arabidopsis*.

Subfamily	Number of *NAC*s per subfamily (% of total *NAC*s^a^)				
	**Wheat** ^b^	Barley	Rice	*Arabidopsis*	Total
a	41 (9)	17 (13)	12 (13)	12 (11)	82 (10)
b	43 (9)	18 (14)	10 (10)	33 (30)	104 (13)
c	53 (12)	12 (9)	10 (10)	13 (12)	88 (11)
d	78 (17)	23 (18)	20 (20)	14 (13)	135 (17)
e	64 (14)	17 (13)	11 (11)	8 (7)	100 (13)
f	12 (3)	3 (2)	8 (8)	3 (3)	26 (3)
g	56 (12)	16 (12)	9 (9)	21 (19)	102 (13)
h	106 (23)	21 (16)	11 (12)	1 (1)	139 (18)
Unclassified	5 (1)	1 (1)	5 (5)	4 (4)	15 (2)
Total number^c^	458	128	96	109	791

a Number expressed as a percentage of the total number of *NAC*s in the given species is given in parenthesis.

b The final classification of wheat NACs was based on the phylogenetic tree constructed for *TaNAC*s and described below rather than the combined tree constructed for all plants studied. There were differences in wheat NAC classification based on which tree was used; for example, the joined phylogenetic tree had 7 unclassified *TaNAC*s but 2 of those were classified into subfamilies based on the *TaNAC* tree below.

c Note that the total number of classified *NAC* genes differs from the total number of identified *NAC* genes since some of the *NAC* genes were excluded from the phylogenetic analysis as explained in the main text.

The 29 wheat, barley, rice, and *Arabidopsis NAC*s functionally characterized (with genetic evidence) for their role in defense against pathogens (Table 3) are highlighted in bold within the NAC phylogenetic tree (Fig. 1a). These were distributed across each subfamily, except “f” and “g,” with “a” containing the highest number (15) of them (Fig. 1a and Table 3). Ten of them were from wheat and were distributed in subfamilies “a,” “c,” “d,” “e,” and “h.” Five uncharacterized defense-associated *TaNAC*s were positioned directly next to their functionally characterized ortholog (*TaNAC002-A1* and *TaNAC048* homoeologs in subfamily “a” next to *HvSNAC1* and *HvNAC6*, respectively, and *TaNAC060-B1* in subfamily “d” next to rice *ONAC060*) (Fig. 1b). Orthologs *TaNAC002-A1* and *HvSNAC1* were differentially expressed in response to *B. graminis* (Supplementary Files 2 and 3, respectively). Expression of orthologs *TaNAC060-B1* and *ONAC060* was, respectively, shown to regulate defense against the obligate biotroph *P. striiformis* and the hemibiotroph *Magnaporthe oryzae* (Supplementary File 4 and Table 3). *TaNAC048* homoeologs and their characterized ortholog *HvNAC6* were involved in positive defense against *B. graminis* (Table 3) and thus are likely isofunctional orthologs.

**Table 3.** Functionally characterized NACs in *Triticum aestivum*, *Hordeum vulgare*, *Oryza sativa* and *Arabidopsis thaliana*.

Subfamily	Species	Cultivar/ecotype	Gene ID	Gene name^a^	mRNA/protein AC	Gene function	Genetic evidence	Reference
a	*T. aestivum*	Suwon11	TraesCS5A02G468300	*TaNAC2, TaNAC002-A1*	AAU08786.1^b^	Suppressed resistance to *Puccinia striiformis* f. sp. *tritici*	VIGS	Zhang *et al.* (2018)
		NAU9918, Yangmai158, OEStpk-V	TraesCS3A02G406000	*TaNAC6A, TaNAC048-A1*	FN396829.1^b^	Enhanced resistance to *Blumeria graminis*	Overexpression and VIGS	Zhou *et al.* (2018)
			TraesCS3B02G439600	*TaNAC6B, TaNAC048-B1*	FN396830.1^b^	Enhanced resistance to *B. graminis*	VIGS	Zhou *et al.* (2018)
			TraesCS3D02G401200	*TaNAC6D, TaNAC048-D1*	FN396831.1^b^	Enhanced resistance to *B. graminis*	VIGS	Zhou *et al.* (2018)
		NIL-R from Sumai 3	TraesCS3B02G194000	*TaNAC032, TaNAC160-B1*	MT512636^b^	Enhanced resistance to *Fusarium graminearum*	VIGS	Soni et al, (2021)
	*H. vulgare*	P-01	HORVU1Hr1G063740	*HvNAC6*	CAM57978.1^b^	Enhanced resistance to *B. graminis*	Overexpression, VIGS, RNA interference	Chen *et al.* (2013); Jensen *et al.* (2007)
		Haruna Nijo	HORVU5Hr1G111590	*HvSNAC1*	AK249102.1^b^	Enhanced resistance to *Ramularia collo-cygni*	Overexpression	McGrann *et al.* (2015)
	*O. sativa*	Nipponbare	Os11g0126900	*ONAC122*	LOC_Os11g03300.1^c^	Enhanced resistance to *Magnaporthe oryzae*	VIGS	Sun *et al.* (2013)
			Os12g0123700	*ONAC131*	LOC_Os12g03040.1^c^	Enhanced resistance to *M. oryzae*	VIGS	Sun *et al.* (2013)
			Os01g0884300	*OsNAC6, ONAC048*	BAG90892.1^b^	Enhanced resistance to *M. oryzae*	Overexpression	Nakashima *et al.* (2007)
	*A. thaliana*	Col-0	At1g01720	*ATAF1, ANAC002*	BT024513.1^b^	Enhanced resistance to *B. graminis*	T-DNA insertion	Jensen *et al.* (2007)
						Conferred susceptibility to *Botrytis cinerea*	Overexpression	Wu *et al.* (2009)
						Suppressed resistance to *B. cinerea*, *Pseudomonas syringae* pv. Tomato *(PsPto)* strain DC3000 or *Alternaria brassicicola*	T-DNA insertion, overexpression	Wang *et al.* (2009)
						Age‐related resistance to *Hyaloperonospora parasitica*	T-DNA insertion	Carviel *et al.* (2009)
			At5g08790	*ATAF2, ANAC081*	BAF00699.1^b^	Suppressed resistance to *B. cinerea*	Overexpression	Delessert *et al.* (2005)
			At1g52890	*ANAC019*	AAM51299.1^b^	Suppressed resistance to *Fusarium oxysporum*	T-DNA insertion, overexpression	Bu *et al.* (2008)
						Suppressed resistance to *P. syringae* pv*. maculicola (Psm)* strain ES4326	T-DNA insertion	Zheng *et al.* (2012)
			At3g15500	*ATNAC3, ANAC055*	ABF74720.1^b^	Suppressed resistance to *F. oxysporum*	T-DNA insertion, overexpression	Bu *et al.* (2008)
						Suppressed resistance to *Psm* strain ES4326	T-DNA insertion	Zheng *et al.* (2012)
						Age‐related resistance to *H. parasitica*	T-DNA insertion	Carviel *et al.* (2009)
			At4G27410	*ANAC072*	NM_001084983.1^b^	Suppressed resistance to *Psm* strain ES4326	T-DNA insertion	Zheng *et al.* (2012)
b	*O. sativa*	Nipponbare	Os03g0119966	*RIM1, ONAC054*	BAH03170.1^b^	Conferred susceptibility to Rice Dwarf Virus (RDV)	Tos17 insertion, overexpression	Yoshii *et al.* (2009)
	*A. thaliana*	Col-0	At5g24590	*TIP, ANAC091*	AAF87300.1^b^	Conferred resistance to Turnip Crinkle Virus (TCV)	Resistant Di-17 inbred line	Ren *et al.* (2000)
			At3g49530	*NTL6, ANAC062*	AAL24091.1^b^	Enhanced resistance to *PsPto* strain DC3000	Overexpression of truncated NTL6-6ΔC, RNA interference	Seo *et al.* (2010)
			At4g35580	*NTL9, CBNAC*	NM_001125650.1^b^	Suppressed resistance to *PsPto* strain DC3000	T-DNA insertion	Kim *et al.* (2012)
c	*T. aestivum*	Suwon11	TraesCS2D02G576400	*TaNAC30, TaNAC031-D3-2*	Tae062585^c^	Suppressed resistance to *P. striiformis* f. sp. *tritici*	VIGS	Wang *et al.* (2018)
d			TraesCS5B02G054200	*TaNAC21/22, TaNAC060-B1*	AGV08300.1^b^	Suppressed resistance to *P. striiformis* f. sp. *tritici*	VIGS	Feng *et al.* (2014)
		Taichung 29	TraesCS7A02G305200	*TaNAC1, TaNAC104-A2*	ADG85703.1^b^	Suppressed resistance to *P. striiformis* f. sp. *tritici*, and to *PsPto* strain DC3000	VIGS (Wheat), ectopic overexpression (Arabidopsis)	Wang *et al.* (2015)
	*O. sativa*	Nipponbare	Os12g0610600	*OMTN3, ONAC060*	XM_015765089.2^b^	Enhanced resistance to *M. oryzae*	RNA interference	Wang *et al.* (2018)
	*A. thaliana*	Col-0	At5g39610	*ANAC092*	AY091191.1^b^	Age‐related resistance to *H. parasitica*	T-DNA insertion	Carviel *et al.* (2009)
e	*T. aestivum*	Thatcher + Lr14b	TraesCS3D02G398200	*TaNAC35, TaNAC075-D1*	KY461080.1^b^	Suppressed resistance to *Puccinia triticina*	VIGS	Zhang *et al.* (2021)
	*O. sativa*	Nipponbare	Os11g0154500	*OsNAC111, ONAC017*	XM_015760375.2^b^	Enhanced resistance to *M. oryzae*	Overexpression	Yokotani *et al.* (2014)
			Os03g0777000	*ONAC066*	LOC_Os03g56580.1^c^	Enhanced resistance to *M. oryzae* and *Xanthomonas oryzae* pv. *Oryzae* [*Xoo*]	Overexpression	Liu *et al.* (2018)
H	*T. aestivum*	CM82036	TraesCS5D02G111300	*TaNACL-D1, TaNAC175-D1*	MG701911.1^b^	Enhanced resistance to *F. graminearum*	Overexpression	Perochon *et al.* (2019)
Unknown	*A. thaliana*	Col-0, Bur-0	At5g64530	*XND1, ANAC104*	NM_001345642.1^b^	Enhanced resistance to *Ralstonia solanacearum*	T-DNA insertion, complementation	Tang *et al.* (2018)

AC, accession number; VIGS, virus-induced gene silencing.

a *T. aestivum NAC* names include the ones that are established based on the nomenclature proposed in this study.

b GeneBank AC.

c TFDB (Transcription factor database) AC.

A total of 146 out of the total 460 *TaNAC*s have been shown to be pathogen responsive based on 5 publicly available RNA-seq studies (Supplementary File 2) and their phylogenetic relationship with the 29 *NAC*s with a validated role in defense was investigated (Fig. 1a). A total of 52 of these pathogen-responsive *TaNAC*s were positioned within the same subclade as a functionally characterized defense-associated *NAC*, and 35 of them were positioned within the same subclade as a defense-associated *TaNAC.* Interestingly, 14 of them were within the “*TaNAC2* subclade,” which includes the functionally characterized *TaNAC048* homoeologs and *TaNAC002-A1* (Fig. 1b).

### The expression pattern and phylogenetic relationship of pathogen-responsive *TaNAC*s

**Fig. 2.**
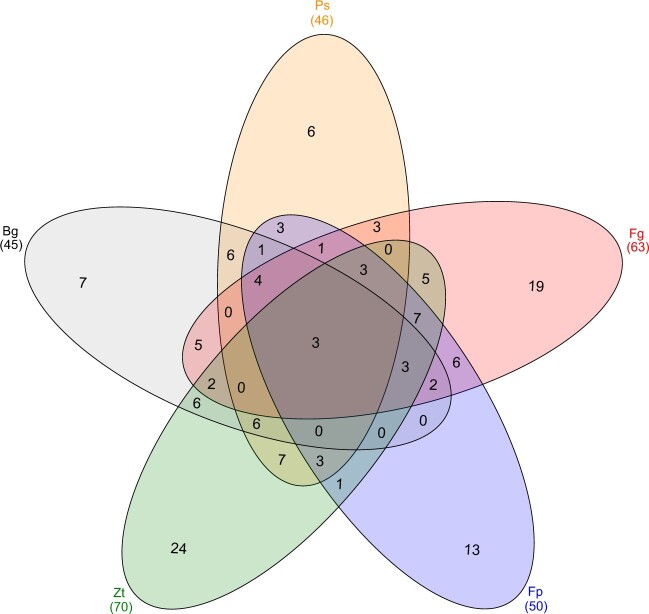
Venn diagram showing the numbers of *TaNAC* genes that are positively and/or negatively transcriptionally responsive to pathogens. Each colored oval represents a specific pathogen response*.* Pathogen abbreviations: Fg, *Fusarium graminearum*; Fp, *Fusarium pseudograminearum*; Ps, *Puccinia striiformis*; Bg, *Blumeria graminis*; Zt, *Zymoseptoria tritici*. The total number of *TaNAC*s responsive to each pathogen is indicated between brackets.

**Fig. 3.**
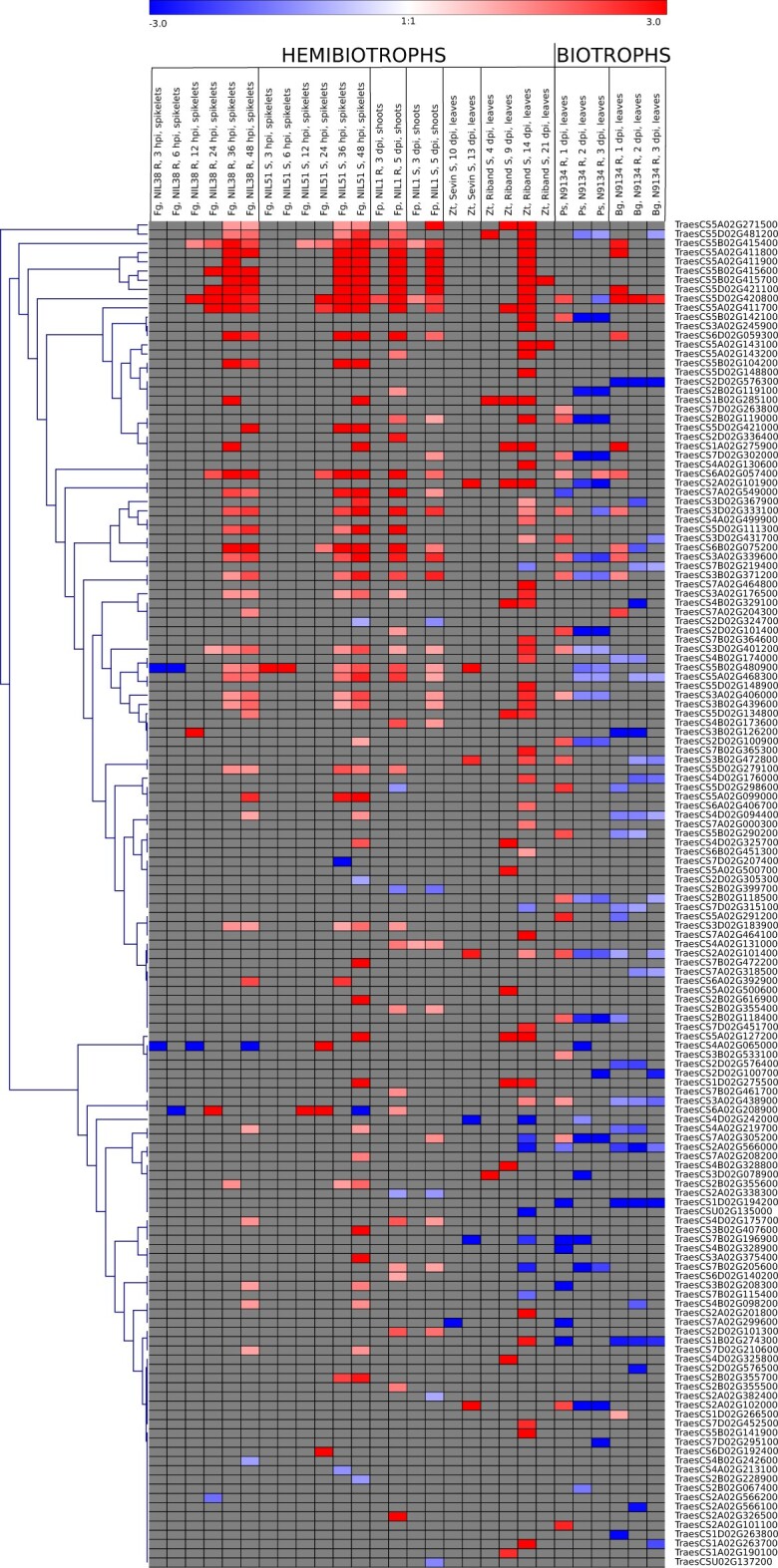
A heatmap of hierarchically clustered *TaNAC* genes indicating the temporal responsiveness to hemibiotrophic and biotrophic fungal pathogens. Color scale at the top of the heatmap represents log2-fold change of gene expression: blue represents downregulated expression and red upregulated gene expression, relative to mock treatment. “R” and “S” next to the cultivar or NIL (near-isogenic line) names indicate resistance and susceptibility to a pathogen, respectively. Pathogen abbreviations: Fg, *Fusarium graminearum*; Fp, *Fusarium pseudograminearum*; Ps, *Puccinia striiformis*; Bg, *Blumeria graminis*; Zt, *Zymoseptoria tritici*.

We investigated the expression profile of the 146 pathogen-responsive *TaNAC*s (Supplementary File 2) and examined if there was any association with the lifestyle (hemibiotrophic or biotrophic) of the activating fungus. Some were specific for a disease (as illustrated in Fig. 2). Fifty-one percentage of all pathogen-responsive *TaNAC*s were specific to hemibiotrophs (*F. graminearum*, *F. pseudograminearum*, *Z. tritici*), and 13% to biotrophs (*B. graminis* and *P. striiformis*) (Figs. 2 and 3 and Supplementary Table 8). Within the 127 *TaNAC*s that were responsive to hemibiotrophic pathogens, a larger proportion were responsive to only hemibiotrophs (53–59% for the different pathogens) as compared to both hemibiotrophs and biotrophs (41–47%). In contrast, a larger proportion of the 71 *TaNAC*s responsive to biotrophic pathogens were responsive to both biotrophs and hemibiotrophs (71–74%) as compared to only biotrophs (26–29%). It is clear from the heatmap (Fig. 3), and time point gene expression data (Supplementary Table 9) that many more *TaNAC*s were upregulated by hemibiotrophs as compared to biotrophs, while many more *TaNAC*s were downregulated by biotrophs as compared to hemibiotrophs. The number of upregulated *TaNAC*s generally increased with time postinfection with hemibiotrophic pathogens (except in cv. Riband infected with *Z. tritici*) and decreased with time postinfection for biotrophic pathogens (Fig. 3 and Supplementary Table 9). The *TaNAC* family had more *Fusarium* (*F. graminearum* and *F. pseudograminearum*)- responsive genes than expected (14% and 11%, respectively), as compared to the whole genome response to these fungi (10% and 6%, respectively; χ^2^ test, *P*-value <0.001); the same was not observed with the other 3 diseases (Supplementary Table 10). Out of curiosity, we also investigated whether fungal-responsive *TaNAC*s were responsive to a bacterial pathogen; of the 146 *TaNAC*s responsive to fungal pathogens, 39 (27%) were also responsive to a bacterial hemibiotroph (*X. translucens*) and 92% of those clustered in subclades enriched with pathogen-responsive *TaNAC*s (see Supplemental Results). For validation purposes, we also conducted analysis to determine if the *TaNAC*s that were most induced by *F. graminearum*, *F. pseudograminearum*, and *P. striiformis* (up to 5 per pathogen) were induced by the same pathogens in other distinct RNA-seq studies (Kugler *et al.* 2013; Dobon *et al.* 2016; Powell *et al.* 2017). All such *TaNAC*s were significantly induced by pathogens in these studies (see Supplemental Results and Supplementary Table 11). In addition, we compared *TaNAC*s responsive to *B. graminis* and/or *P. striiformis* with those delineated in studies by Lv *et al.* (2020) and Ma *et al.* (2021) who analyzed the same RNA-seq study (E-MTAB-4289). There were likely differences in analysis and cross-comparison was not directly possible for all RNA-seq results from Lv *et al.* (2020), which may explain why many of our pathogen-responsive TaNACs were not detected as being so in their studies and vice versa. But 18 and 11 *TaNAC*s, respectively, responsive to *B. graminis* and *P. striiformis* herein were also responsive to the same pathogens in Lv *et al.* (2020) and/or Ma *et al.* (2021) (see Supplemental Results and Supplementary Tables 12 and 13).

**Fig. 4.**
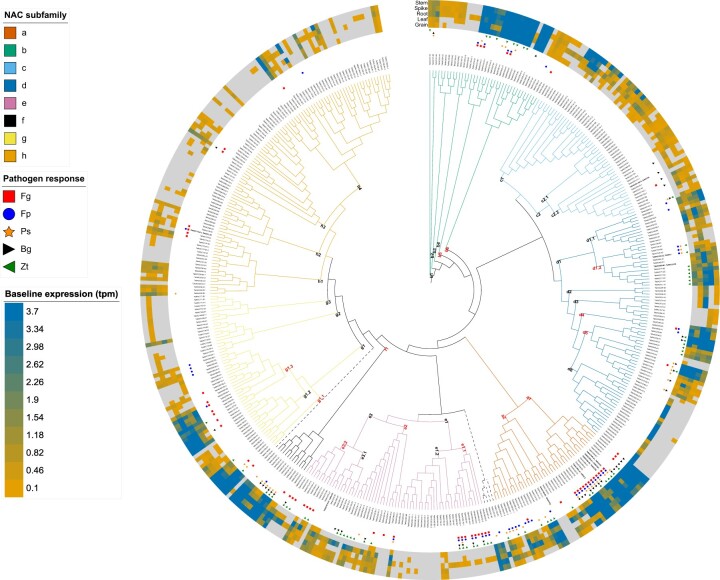
Consensus maximum likelihood tree of TaNAC proteins with their corresponding healthy tissue and disease-responsive gene expression profiles indicated (https://itol.embl.de/tree/372282299294211613865063). Branch lines are colored according to NAC subfamily (unclassified branches and clades are indicated with a dashed line). Within a subfamily, TaNAC subclades are coded at the node based on the subfamily letter and a specific number. For example, “e1” is the first subclade of subfamily “e,” and “e1.1” and “e1.2” are two distinct subclades within “e1” subclade. Red font indicates subclades enriched with pathogen-responsive *TaNAC*s. TaNACs highlighted in bold are functionally characterized in pathogen defense (Table 3). The colored symbols denote the TaNACs that are responsive (at the transcriptome level) to pathogens. Pathogen abbreviations: Fg, *Fusarium graminearum*; Fp, *Fusarium pseudograminearum*; Ps, *Puccinia striiformis*; Bg, *Blumeria graminis*; Zt, *Zymoseptoria tritici*. *TaNAC* expression within healthy tissues (grain, leaf, root, spike, and stem) is represented by a color scale that represents baseline expression in tpm values, and the color gradient maximum is set to 3.7 tpm.

A wheat-specific phylogenetic tree was constructed to examine the distribution of pathogen-responsive *TaNAC*s within NAC subfamilies (Fig. 4). The tree was inferred based on the MSA of 446 *TaNAC* sequences and 218 sites/columns with completeness score (Ca) of 0.9 (Supplementary File 6); 14 surplus identical sequences were removed from the phylogenetic analysis (Supplementary Table 2). Eighty-six percentage (119) of the pathogen-responsive *TaNAC*s were densely clustered into 13 pathogen-responsive-enriched subclades within subfamilies “a,” “b,” “d,” “e,” “f,” and “g” (delineated by red labels at the nodes of the tree; ≥60% of *TaNAC*s within these subclades being pathogen responsive). All pathogen-responsive *TaNAC*s in subclade “g1.3” were only responsive to hemibiotrophs. At the subfamily level, “a,” “e,” and “f” were significantly enriched in pathogen-responsive *TaNAC*s, as compared to “c” and “h” (representing 80, 45, 67, 11, and 8% of the total number of *TaNAC*s in the subfamily, respectively; *P*-value <0.05, Fisher’s exact test) (Supplementary Table 14). The majority (>66%) of the *TaNAC*s in subfamilies “a,” “e,” and “f” were responsive to more than 1 pathogen, while most (>78%) of the pathogen-responsive *TaNAC*s in subfamilies “c” and “h” were responsive to only 1 pathogen (Supplementary Table 14). Subfamilies “a” and “e” had the highest number (10-22) of *TaNAC*s responsive to *F. graminearum*, *B. graminis*, and *Z. tritici*, as compared to other subfamilies, and subfamily “a” had the highest number of *TaNAC*s responsive to *F. pseudograminearum* and *P. striiformis* (20 and 17, respectively) (Fig. 4 and Supplementary Fig. 1).

In general, pathogen-responsive *TaNAC*s within subclades did not follow similar expression patterns for different pathogens (Supplementary Fig. 2). However, all *TaNAC*s in the “a1” subclade (the “*TaNAC2* subclade”) were pathogen responsive, with 83% and 61% of the *TaNAC*s upregulated by *F. graminearum* and *F. pseudograminearum*, respectively, and 56% up- and/or downregulated to *B. graminis*, in at least 1 timepoint (Fig. 1b and Supplementary Fig. 2). Similarly, >80% of the genes in the “e1.1” subclade were upregulated by *F. graminearum, F. pseudograminearum*, and *Z. tritici*.

The expression profile of *TaNAC*s in healthy wheat organs (grain, leaf, root, spike, and stem) was assessed to determine if this differed for pathogen vs. nonpathogen responsive genes (Fig. 4 and Supplementary File 7; Choulet *et al.* 2014). The majority (92%) of pathogen-responsive *TaNAC*s were expressed in healthy tissue and in at least 2 organs, while 6% were expressed in 1 organ and 2% were not expressed in any organ. The baseline expression of pathogen-responsive *TaNAC*s was high compared to nonpathogen responsive *TaNAC*s (mean tpm in all tissues >4.7 as compared to <1.2 for all other *TaNAC*s).

### *TaNAC*s in subfamilies “f,” “g,” and “h” encode a diverged NAM domain

**Fig. 5.**
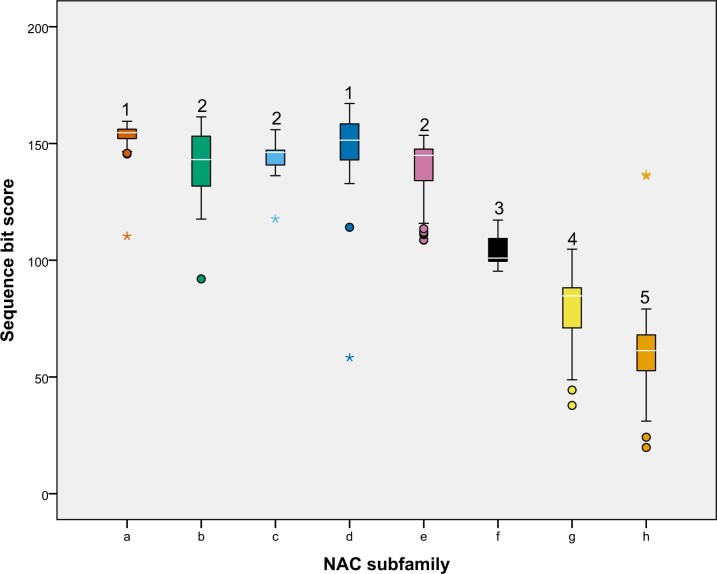
The sequence bit scores from HMMER output across the TaNAC subfamilies. The sequence bit scores indicate the probability of a TaNAC sequence being homologous to the *hmm* profile of the NAM domain (Finn *et al.* 2011). Within the boxplots, the white line denotes the median. Outliers and extreme values, below or above whiskers, are marked with the circle or the star, respectively. Boxplots that do not share the same number above whiskers are significantly different (*P*-value <0.05).

**Fig. 6.**
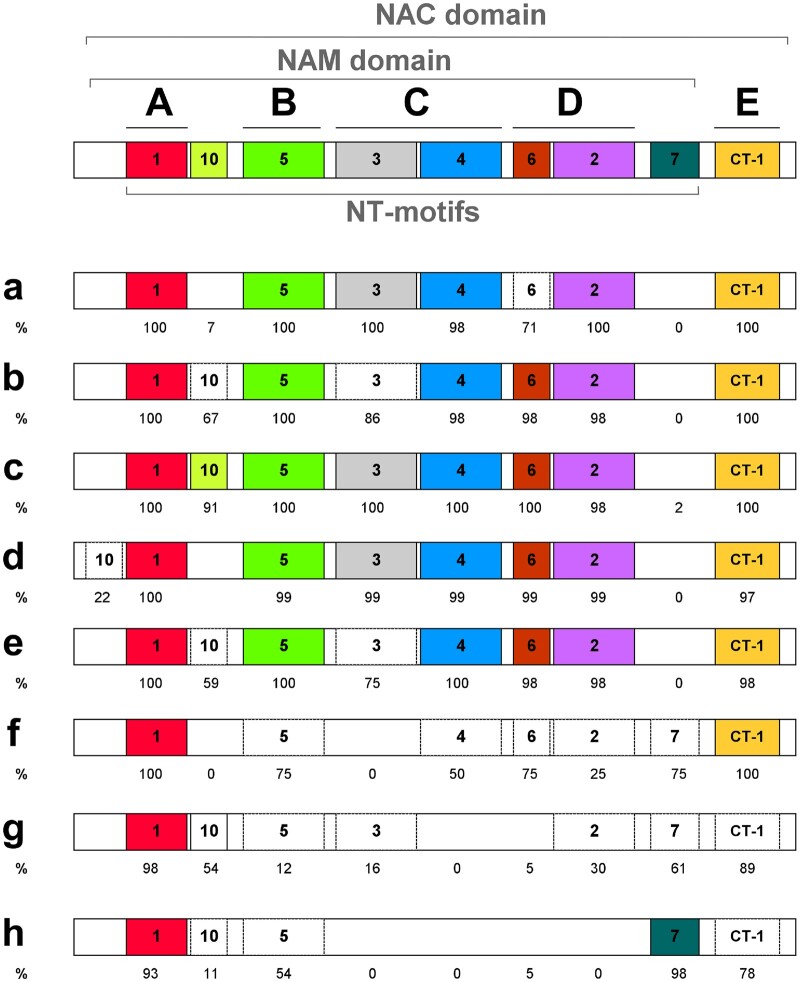
Schematic representation of a conserved NAC domain (on the top) within TaNAC proteins and the distribution of NAC motifs within the protein subfamilies. Only highly conserved motifs present in at least 90% of the TaNACs in at least 1 subfamily are presented in the scheme. Based on Ooka *et al.* (2003), a canonical NT NAC domain comprises the NAM domain (encompassing subdomains A–D) and a subdomain E. Motif distribution within subdomains is illustrated; a motif is colored if it considered a signature of a subfamily, being present in more than 90% of TaNACs, while a motif is uncolored if present in 10–90% of TaNACs in the subfamily; a motif is absent when less than 10% of TaNACs in the subfamily contain it. The values below each motif represent the percentage of TaNACs in the subfamily that contain it.

The highly conserved NAM domain has not previously been analyzed in wheat and hence was investigated herein based on HMM (the higher the score the better the match with the profile of the NAM domain), motif and domain analysis (Figs. 5 and 6). Bit scores within subfamilies a–e were high (116–167), with some small but significant differences between these subfamilies. In comparison, there was greater NAM domain divergence within subfamilies f–h as indicated by the lower scores (31–117) (Fig. 5). We examined the distribution of a total of 21 motifs within the NT NAC domain and 39 motifs within the putative CT TAR of all wheat TaNACs (Supplementary Fig. 3, a and b and Supplementary Tables 15 and 16). Eight NT motifs were specific for a subfamily and were present in 8–61% of subfamily members. The 7 most conserved motifs (NT-1 to NT-6, and CT-1) were all present in more than 50% of TaNACs (Supplementary Fig. 3a); all others were present in less than 40% of the TaNACs. Figure 6 schematically illustrates how the motifs map to the conserved NAC subdomains A–E within each TaNAC subfamily. MEME analysis deduced that the 7 conserved motifs mapped to subdomains A–E. An additional 2 NT motifs (NT-7 and NT-10) were found to be positioned between the NAC subdomains.

All subfamilies had well-conserved subdomains A and E (motifs NT-1 and CT-1, respectively; Fig. 6). In addition, subdomains B, C, and D (motifs NT-2, -3, -4, -5, and -6) were well conserved within subfamilies a–e, while in subfamilies f–h, these subdomains were partially present or absent. Thus, this confirms the significant divergence of NAM domain in subfamilies f–h. Interestingly, motif NT-7 is specific to subfamilies f–h and found nearly in all TaNACs from family “h” (98%). It may have diverged from NT-2 because they both have conserved amino acid residues T[x]W[x]MHE when comparing motif logos (Supplementary Table 15). None of the 39 motifs within TAR were conserved within the TaNAC family, all being present in <19% of the total number of TaNACs. But 28 of them were conserved within subfamilies, being specific to a subfamily (Supplementary Fig. 3b) and present in 10–34% of the total number of TaNACs in the subfamily (Supplementary Fig. 4). Twenty-six of these 28 CT motifs were specific for a subclade (all except CT-7 and CT-17) (Supplementary Fig. 4).

### CT motifs of TaNACs specific for the subclade may be linked to a pathogen responsiveness or an expression in grain

**Figure 7:**
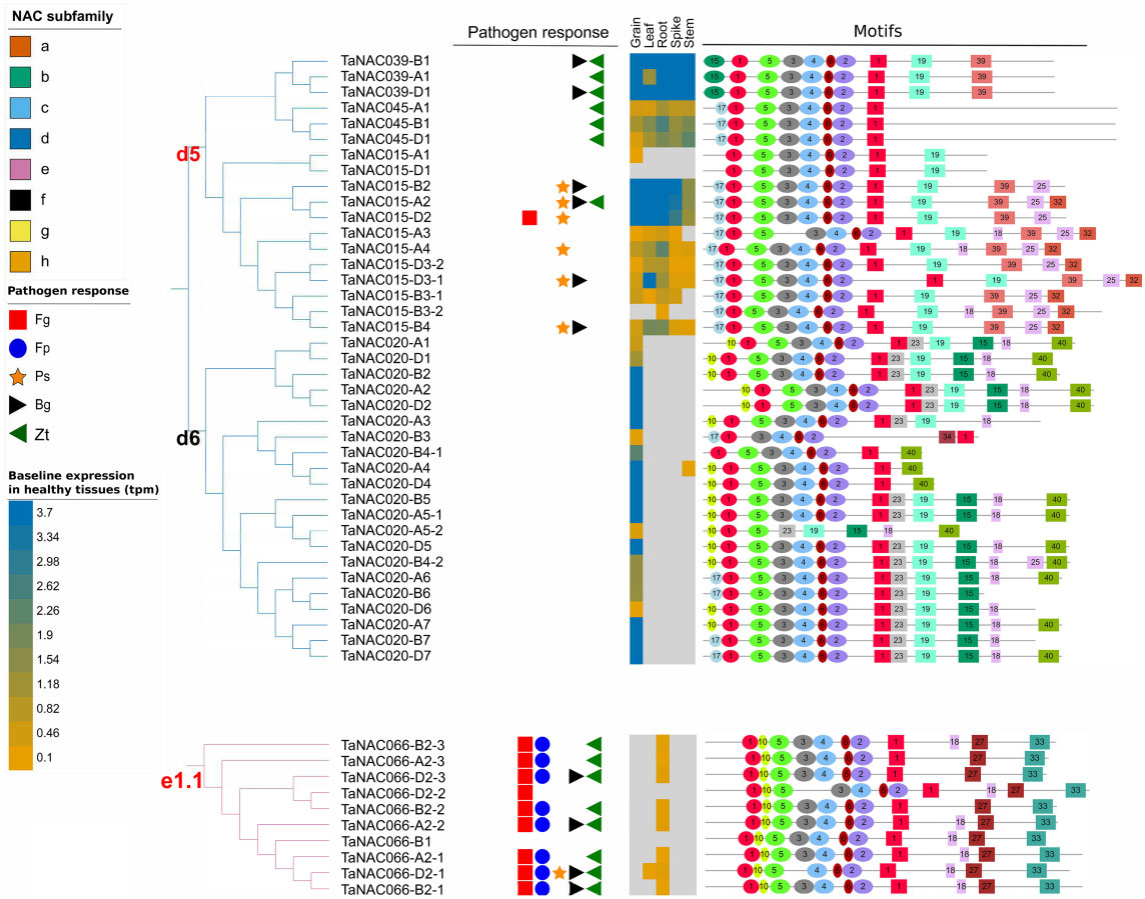
Motif composition of TaNACs in subclades “d5” and “e1.1” enriched with pathogen-responsive *TaNAC*s, and the grain-specific subclade “d6” as part of consensus maximum likelihood tree of TaNAC amino acid sequences (https://itol.embl.de/tree/372282299294211613865063). “D5” and “d6” are 2 of the 6 subclades within subfamily “d,” and “e1.1” is one of the 2 distinct subclades within “e1” subclade within subfamily “e.” Pathogen responsiveness is indicated with colored symbols (see legend). Pathogen abbreviations: Fg, *Fusarium graminearum*; Fp, *Fusarium pseudograminearum*; Ps, *Puccinia striiformis*; Bg, *Blumeria graminis*; Zt, *Zymoseptoria tritici*. For tissue-specific expression, the colored scale represents tpm baseline expression values for the genes in healthy tissues (grain, leaf, root, spike, and stem) (see baseline expression legend). Motifs: NT (circle) and CT (rectangle) motifs are represented with a different colors and numbers. Gray lines flanking the motifs represent nonconserved regions of TaNACs.

The association, if any, between NT or CT motif composition and expression pattern of *TaNAC*s in either healthy tissues, or in response to pathogens, was investigated (detailed motif composition is illustrated in Supplementary Fig. 4). In general, the developmental and pathogen response of *TaNAC*s was not associated with any specific NT or CT motif. The exceptions are illustrated in Fig. 7. Motifs CT-27 and CT-33 specific for the “e1” subclade were found in all pathogen-responsive *TaNAC*s within that subclade. In addition, CT-39 specific for the “d5” subclade that is enriched with pathogen-responsive *TaNAC*s was present in 72% of *TaNAC*s of the subclade. In terms of tissue-specific expression patterns, all *TaNAC*s in the subclade “d6” were expressed only in grain, except 1 gene that was expressed also in stem tissue at a relatively low level. Motifs CT-23 and CT-15 were specific for the subclade “d6” and present in 81% and 76% of the TaNACs within the subclade, respectively.

## Discussion

This study represents the first in-depth in silico analysis of *TaNAC*s responsive to diverse fungal diseases, which included assessment of their phylogenetic relationship with defence-associated *NAC*s from *Arabidopsis*, rice, and barley. Subfamily “a” had the highest number of functionally characterized defense-associated *NAC*s from wheat, barley, rice, and *Arabidopsis*. Therefore, the function in pathogen defense seems to be conserved and characteristic for subfamily “a,” as first suggested by Shen *et al.* (2009). Subfamily “a” has the lowest evolutionary rate, being the most conserved NAC subfamily that originated in mosses (Zhu *et al.* 2012; Jin *et al.* 2017), and this is supported by our findings. Hence, subfamily “a” NACs may be a crucial component of plants’ immune systems since the origin of basal land plants.

Within subfamily “a,” 14 pathogen-responsive *TaNAC*s orthologous to defense-associated *NAC*s were positioned in the “*TaNAC2* subclade.” Several heterospecies and isospecies homologs within the “*TaNAC2* subclade” share the same proven function, e.g. *HvNAC6*, *ATAF1*, and *TaNAC048* homoeologs were positive regulators of penetration resistance against *B. graminis* (Jensen *et al.* 2007; Chen *et al.* 2013; Zhou *et al.* 2018), and *ATAF1* and *ATAF2* were both negative regulators of resistance to *Botrytis cinerea* (Delessert *et al.* 2005; Wang *et al.* 2009). Therefore, one could speculate regarding the function of uncharacterized *NAC*s in the “*TaNAC2* subclade.” For example, *TaNAC067-A1*, *TaNAC002-B1* and *-D1* homoeologs, and *TaNAC068* homoeologs were all responsive to *P. striiformis* and were the closest homologs of the *TaNAC2* involved in *P. striiformis* defense based on their phylogenetic relationship. Thus, these *NAC*s are very likely involved in defense against *P. striiformis*. Eighty-three percentage of *TaNAC*s within the “*TaNAC2* subclade” were responsive to *F. graminearum* and are likely involved in defense against that fungus. In addition, the “TaNAC2 subclade” contains orthologs (1) from all 4 species included in this study that are pathogen responsive (based on the data in the public Expression Atlas database; results not shown) and (2) from rice and *Arabidopsis* that are functionally characterized as playing a role in pathogen defense. Thus, the “TaNAC2 subclade” contains *NAC*s that are important for immunity in both monocot and dicot plants.

Many of the disease-responsive *TaNAC*s were within phylogenetic subfamilies “a,” “e,” and “f,” and many were universally responsive to pathogens. Interestingly, the *Arabidopsis* ortholog of defense-associated rice *ONAC017* and *TaNAC017* from pathogen-enriched tree subclade “e3.2” (AT3G44350) was identified as a “core immunity response gene” upregulated by 6 different elicitors of pattern-triggered immunity (Bjornson *et al.* 2021). Universal response *TaNAC*s warrant further investigation for their potential as broad-spectrum defense-associated signaling hub genes, as do subclade “g1.3” for their potential as hemibiotroph-specific *TaNAC*s. *TaNAC*s in defense-associated subfamilies “a,” “e,” and “f” were also responsive to abiotic stressors (Borrill *et al.* 2017) or have a proven role in abiotic stress resistance (see Supplemental Results), suggesting that these subfamilies evolved to regulate biotic and abiotic responses in wheat. Several characterized NACs within defense-associated subfamily “a” were proven to be multifunctional and 6 uncharacterized *TaNAC*s shared a subfamily with orthologs functionally characterized as playing a role in either abiotic stress responses, senescence, and/or starch synthesis (results not shown). Genes providing resistance against multiple pathogens or stressors are highly valued in breeding programs. The rapid advances in wheat genome sequencing will give us insights into the level of allelic diversity within *TaNAC*s and follow-on studies will determine whether allelic divergence affects function and the potential of *TaNAC*s as target genes in wheat breeding programs.

This study demonstrated that response of *TaNAC*s to pathogens is conserved in certain subfamilies and subclades. Similarly, Borrill *et al.* (2017) previously demonstrated that more phylogenetically related *TaNAC*s shared more similar expression patterns compared to the more distant *TaNAC*s. Thus, this study supports the previous finding. Our study also highlighted *TaNAC*s previously identified as being pathogen-responsive. By comparing *TaNAC*s responsive to *B. graminis* and *P. striiformis* delineated herein with the ones determined in studies by Lv *et al.* (2020) and Ma *et al.* (2021), we highlighted pathogen-responsive genes common to all studies. But many could not be compared (due to data available) or were not commonly pathogen-responsive across studies. We attempted to determine if the latter was due to differences in methodology used for differential expression analysis, but the available information was insufficient to enable such a comparison. For the *TaNAC*s, commonly responsive across studies to *B. graminis* and *P. striiformis* were placed in the subclades enriched with pathogen-responsive *TaNAC*s and the majority of them exhibited a universal pathogen response, and as such should be further explored for their function in biotic stress responses. Within the subclades enriched with pathogen-responsive *TaNAC*s, the ones with the universal pathogen response and candidates in the “TaNAC2 subclade” could be the strongest candidates for further functional validation and breeding purposes. Of course, since this study mined pathogen-responsive *TaNAC*s by analyzing a limited number of RNA-seq studies, it is expected that other candidates responsive to certain pathogens in specific cultivars and at specific timepoints and the ones that are not regulated at the transcriptional level were missed herein.

TaNAC2 subclade genes identified herein exhibit allelic variation and locate within disease resistance quantitative trait loci (QTL). Based on the current data in the EnsemblPlants website (https://plants.ensembl.org/index.html), all except 3 *TaNAC*s (*TaNAC088-B1*, *TaNAC002-B1*, and *TaNAC068-A1*) within the “TaNAC2 subclade” had at least 1 gene variant that would result in a truncated protein or a protein with a missense mutation (results not shown). Three genes from the “TaNAC2 subclade” (*TaNAC088-A1, TaNAC088-B1*, *TaNAC009-A1*, and *TaNAC009-A1*) are, respectively, located within QTL MQTL1A.2, MQTL1B.4, MQTL4A.1, and MQTL4D.1 (Saini *et al*. 2022), which were associated with resistance to multiple diseases. Thus, future studies should use new available resources to explore natural allelic and copy number variation for *TaNAC*s within the wheat pangenome. These genetic resources could provide the tools for further functional exploration of the gene family and for breeding purposes as explained in detail in a previous review (Borrill *et al*. 2019). These and other genomic tools can be used to determine the functional importance of *TaNAC*s located within QTL associated with disease resistance.

The observation that the majority of *TaNAC*s were specific to the hemibiotrophs (at least those tested herein) is interesting, and further studies should determine whether they are involved in 1 or both phases (biotrophic and necrotrophic) of these diseases. The observation that the number of upregulated *TaNAC*s increased with time for hemibiotrophs suggests their implication in the later necrotrophic stage of the infection, but this remains to be determined. The majority of *TaNAC*s responsive to the hemibiotrophic bacteria *X. translucens* (76%) were also responsive to hemibiotrophic fungi, further supporting the hypothesis that many *TaNAC*s might had preferably evolved to regulate defense against hemibiotrophic pathogens. This hypothesis can be tested as more wheat disease transcriptional data becomes available in the future. Our data showed that *TaNAC2* was downregulated upon infection with the biotrophs and upregulated upon infection with *Fusarium* fungi at the later stage of infection (36 hpi), most likely being the necrotrophic phase. Silencing of *TaNAC2* and *TaNAC30*, respectively, from subfamilies “a” and “c,” enhanced resistance against biotrophic *P. striiformis* via accumulation of hydrogen peroxide and decreased hyphal growth at the early stage of infection (Wang *et al.* 2018; Zhang *et al.* 2018). Thus, it is very likely that these 2 *TaNAC*s negatively regulate programmed cell death at the early stage of biotrophic infection.

In the joined phylogenetic tree of wheat, barley, rice, and *Arabidopsis* NACs, the proteins were distributed in 8 distinct subfamilies a-h, which agrees with the classification first proposed by Shen *et al.* (2009). Herein, it was shown that subfamily “h” expanded in grasses, particularly wheat; wheat contained 5 and 10 times more *NAC* genes than in barley and rice, respectively, while *Arabidopsis* had only 1 *NAC* in subfamily “h.” Shen *et al.* (2009) reported a higher proportion of subfamily “h” *NAC* sequences in rice (24%) than in this study (12%), which can be explained by the lower number of rice sequences used to infer phylogeny herein. It should also be noted, compared to this study, respectively, 29, 50, and 4 more *NAC*s were identified in extensive research of barley, rice, and *Arabidopsis* (Nuruzzaman *et al.* 2010; Murozuka *et al.* 2018), which can be explained by differences in analyses. Expansion of the *TaNAC* family has been explained through small-scale duplication and retroposition events (Guérin *et al.* 2019), but the ecological drivers remain to be determined.

To our knowledge, this is the first study to quantitatively assess the divergence of the NAM domain of TaNAC proteins. It deduced that the NAM domain significantly diverged in sequence in subfamilies f–h. These subfamilies partially or completely lost subdomains C and D, which are implicated in DNA binding. Motif NT-4 in subdomain C contained the DNA-recognition motif (WKATGTDK) that binds DNA (Welner *et al.* 2012). Also, the NT-2 motif in subdomain D contained conserved amino acids of the β4–β5 loop, which is also a possible DNA-binding site (Duval *et al.* 2002; Ernst *et al.* 2004). The NT-4 motif was absent in NACs in subfamilies “g” and “h,” and NT-2 was partially lost in subfamilies “f” and “g” and absent in “h.” Whether or not this affects their DNA-binding activity remains to be determined. Only subdomains A and E were conserved in all subfamilies suggesting that these subdomains could be the most important for the NAC domain function. Indeed, it has been proven that the disruption of the NAC dimer interface located in subdomain A reduced the affinity for DNA-binding (Ernst *et al.* 2004; Olsen *et al.* 2005). Similarly, subdomain A was proven to be the dimerization domain in wheat NAM-A1 and mutation within the subdomain impaired the physiological function of NAM-A1 (Harrington *et al.* 2019). However, in vitro site-directed mutagenesis studies of NAC subdomains in TaNAC69 showed that all 5 subdomains were important for the DNA-binding function, and subdomains A, D and E were necessary for dimerization (Xue *et al.* 2006). In total, we found 14 newly identified putative NT motifs that were not associated with conserved subdomains A–E and the majority were present in divergent subfamilies “f,” “g,” and “h.” These features may contribute to the functional diversity of wheat NACs in these divergent and as yet uninvestigated subfamilies.

Within the TAR, we identified 39 motifs, 5 of where were previously identified in Borrill *et al.* (2017) who annotated *TaNAC* genes from the previous wheat genome version. The CT-7 motif matched with a transcriptional activation domain called the “WQ-box” (Ko *et al.* 2007). The “WQ-box” was found in NST (NAC secondary wall-thickening promoting factor) and VND (vascular-related NAC domain) proteins in subfamily “c” and is specific for vascular plants (Shen *et al.* 2009). The motif CT-24 was identified in subfamily “h” (Borrill *et al.* 2017), and in this study also in subfamilies “a,” “e,” “f,” and “g.” The motif was part of the nuclear localization signal recently functionally characterized in the FHB resistance gene *TaNACL-D1* described by Perochon *et al.* (2019), which belonged to subfamily “h.” TaNACL-D1 did not have subdomain C and beyond that, this study differed from Perochon *et al.* (2019) in that our analysis indicates it also lacked the subdomains D and E. The differences between the 2 studies are most likely reflective of different parameters and numbers of sequences used for analysis, the larger number of sequences used in this study increasing the chances of detecting intraspecies motif differences.

Variations in NT motifs were not associated with *TaNAC*s expressed in healthy or diseased wheat organs. However, within the TAR, CT-27 and CT-33 were specific for the subclade “e1.1,” and CT-39 was specific for the subclade “d5,” and both subclades were enriched in pathogen-responsive *TaNAC*s. But to date, no NAC motifs have been experimentally proven to directly influence their function in pathogen defense. Similarly, CT-15 and CT-23 in the TAR were specific for the subclade “d6” wherein *TaNAC*s were specifically expressed in grain. The “grain NACs” in maize (*ZmNAC128* and *ZmNAC130*), and rice (*ONAC020* and *ONAC026*) positively regulated starch and storage protein synthesis in grain (Zhang *et al.* 2019; Wang *et al.* 2020) and had a CT motif that matched CT-23 described herein (Murozuka *et al.* 2018). The 2 rice “grain NACs,” *ONAC020* and *ONAC026*, were orthologs of all grain-specific *TaNAC*s in subclade “d6” described herein. Of those grain-specific *TaNAC*s, *TaNAC020-A1* had a very short CT compared to other “grain NACs” from the subclade “d6” and it lacked the CT-15 and CT-23 motifs. *TaNAC020-A1* was proven to be transcriptional repressor that negatively affected grain weight and starch synthesis (Liu *et al.* 2020). However, whether the presence or absence of CT-15 and CT-23 in the “grain-NACs” is associated with positive or negative effect on grain development remains to be investigated.

In conclusion, this study provided *TaNAC* nomenclature, which will facilitate consistent naming of all such wheat genes and insights into the phylogeny and pathogen responsive of this gene family in wheat. We determined that subfamilies “a,” “e,” and “f” and subclades enriched with pathogen-responsive *TaNAC*s are rich in defense-associated *TaNAC*s. For the first time, this study demonstrated that the NAC domain diverged in wheat subfamilies “f,” “g,” and “h.” Diverged motifs determined herein may be implicated in yet uninvestigated diverged function of the NAC domain in these subfamilies. Lastly, we identified 3 CT motifs associated with pathogen responsiveness and 2 associated with specific expression in grain. Their functional validation will provide a “fingerprint” to identify defense- or grain-related function in otherwise noncharacterized *NAC*s in wheat and related crops.

## Supplementary Material

jkac247_Supplemental_Figure_S1

jkac247_Supplemental_Figure_S2

jkac247_Supplemental_Figure_S3

jkac247_Supplemental_Figure_S4

jkac247_Supplemental_File_S1

jkac247_Supplemental_File_S2

jkac247_Supplemental_File_S3

jkac247_Supplemental_File_S4

jkac247_Supplemental_File_S5

jkac247_Supplemental_File_S6

jkac247_Supplemental_File_S7

jkac247_Supplemental_Tables_S1_S16

jkac247_Supplemental_Results

## Data Availability

The data generated/analyzed during the study are available in the supplementary files, supplementary figures, and supplementary tables. The supplemental files contain the Supplemental Results (Supplemental_Results.docx) and the following data: Supplementary Files 1–7, Supplementary Tables 1–16, and Supplementary Figs. 1–4. Online versions of Figs. 1a and 4 are available at https://itol.embl.de/tree/372282299412371613249495 and https://itol.embl.de/tree/372282299294211613865063, respectively. DE_dataset_Vranic_et_al.xlsx contains the DESeq2 output used to extract differential expression profiles of pathogen-responsive *TaNAC*s and was submitted on Figshare: https://doi.org/10.25387/g3.19326374. Supplemental material is available at G3 online.
