# Supplementary material for: Comprehensive analysis of pathogen-responsive wheat NAC transcription factors: new candidates for crop improvement
Source: G3 (Bethesda). 2022 Sep 21;12(11):jkac247. doi: 10.1093/g3journal/jkac247 (PMC9635653; doi:10.1093/g3journal/jkac247)
Supplement: jkac247_Supplemental_Figure_S1 [file jkac247_supplemental_figure_s1.pdf]

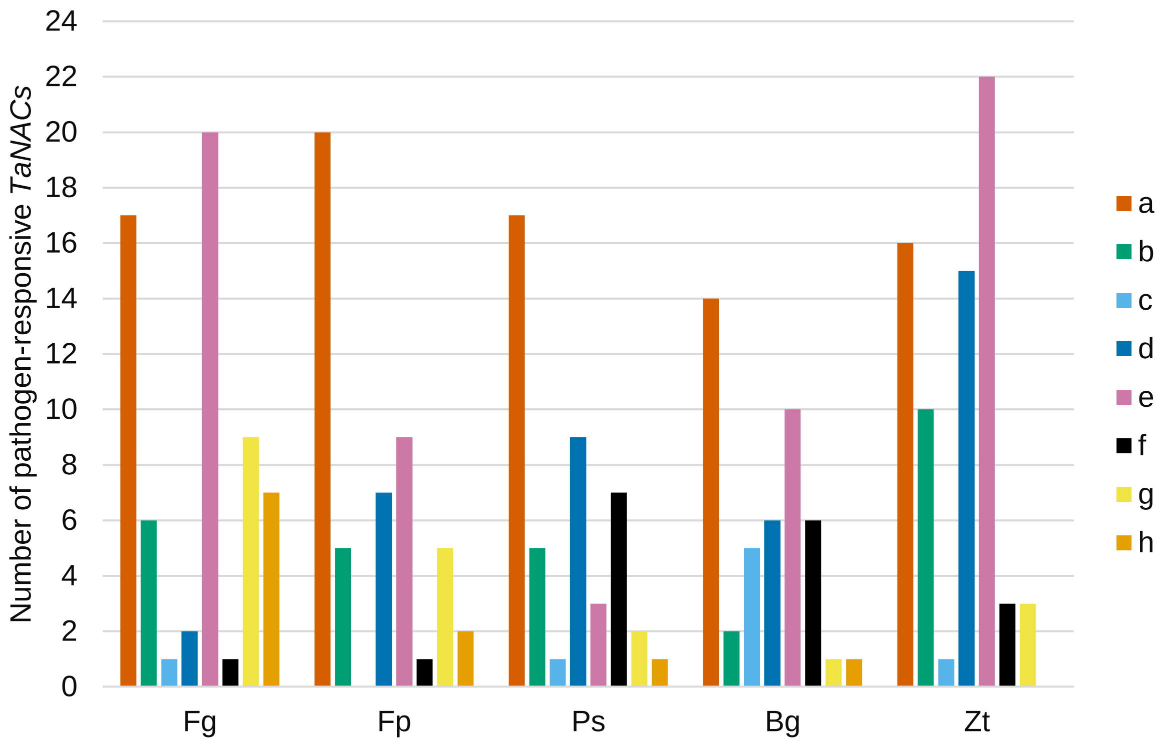

Figure S1 Number of *TaNAC* genes in subfamilies a-h responsive to a certain pathogen: Fg, *Fusarium graminearum*; Fp, *Fusarium pseudograminearum*; Ps, *Puccinia striiformis*; Bg, *Blumeria graminis*; Zt, *Zymoseptoria tritici*.
