# Supplementary figures and images for: Comprehensive analysis of pathogen-responsive wheat NAC transcription factors: new candidates for crop improvement
Source: G3 (Bethesda). 2022 Sep 21;12(11):jkac247. doi: 10.1093/g3journal/jkac247 (PMC9635653; doi:10.1093/g3journal/jkac247)

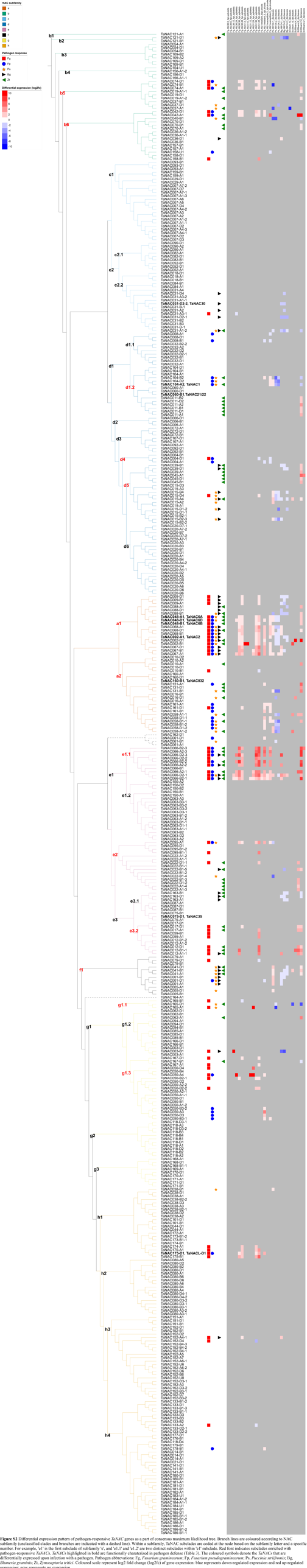

Supplement: jkac247_Supplemental_Figure_S2 [file jkac247_supplemental_figure_s2.pdf]
