# Supplementary material for: Comprehensive analysis of pathogen-responsive wheat NAC transcription factors: new candidates for crop improvement
Source: G3 (Bethesda). 2022 Sep 21;12(11):jkac247. doi: 10.1093/g3journal/jkac247 (PMC9635653; doi:10.1093/g3journal/jkac247)
Supplement: jkac247_Supplemental_Figure_S3 [file jkac247_supplemental_figure_s3.pdf]

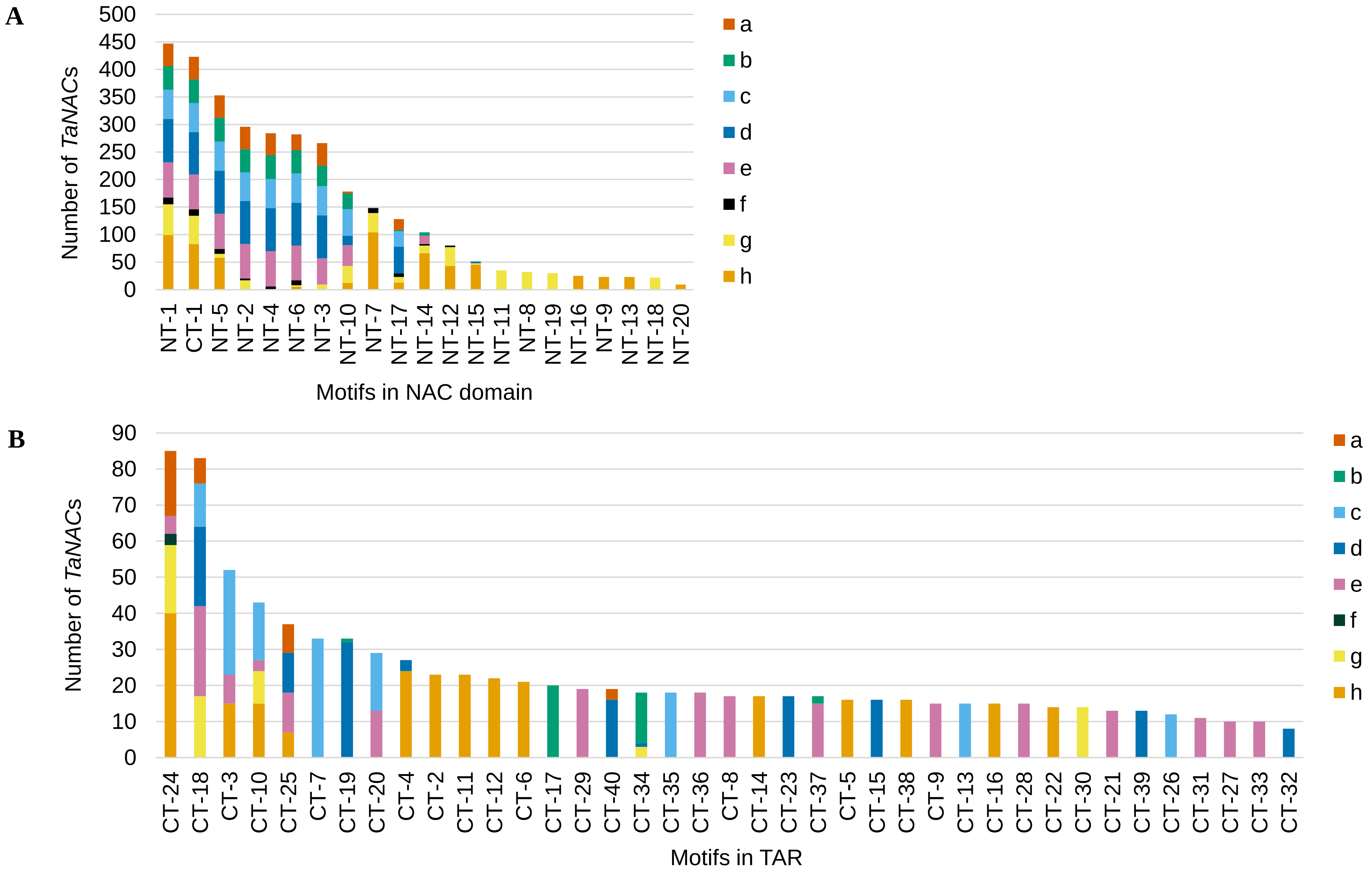

Figure S3 Distribution of protein motifs across TaNAC subfamilies a-h within **(A)** NAC domain and **(B)** transcriptional activation region (TAR).
