# Supplementary material for: Comprehensive analysis of pathogen-responsive wheat NAC transcription factors: new candidates for crop improvement
Source: G3 (Bethesda). 2022 Sep 21;12(11):jkac247. doi: 10.1093/g3journal/jkac247 (PMC9635653; doi:10.1093/g3journal/jkac247)
Supplement: jkac247_Supplemental_File_S1 [file jkac247_supplemental_file_s1.docx]

File S1 Masked multiple sequence alignment of unique 751 wheat, barley, rice, and Arabidopsis NAC protein sequences.

>TraesCS1A02G190100.1

--TLPPGFRFHPTDDELVGYYLKRRVDNLKIEEVIPVIDLYKCEPWELPEKSFLPKREWFFFVPRDRKYPNG

SRTNRATTTGYWKATGKDRKVSCD-GAVCGVRKTLVFYKGRAPGGERTDWVMHEYRLCQGNFI-GAYALCRV

IKRTEAGLSSSQQGADFTFS--EASLDENN-----

>TraesCS1A02G275900.1

MG-LPPGYRFYPTEEELVRFYLRHMLDGRR-GRVIPVADVCSLDPWQLPEVH--RAGEWFYFCARQDREARG

GRPSRTTPSGYWKAAGTPGLVYSA-GCPVGTKKTMVFYRGRAPAGAKTKWKMNEYRAF--QVR--EFSLCRL

YTRSGSLR--P---V--PASAPNDRSVQQQ---DW

>TraesCS1A02G276000.1

MA-LPPGYRFYPTEEELVRFYLRHKLDGRR-ARVIPVADVCSLDPWQLPEVH--RASEWFYFCARQDREARG

GRPSRTTPSGYWKAAGTPGLVYSA-GHLVGTKKTMVFYRGRAPAGAKTKWKMNEYRAL-PQVR--EFSLCRL

YTSSGNMR--P---A----SALNDRHVQQQ---NW

>TraesCS1B02G113000.1

MAR---GLIFAPGDADLITIYLQRKISGLPLPPYIHNADVYAAEPAALP-SAS-G--RWYFFTSV-RQSSRG

TRRCRAVAGGTWHEKA-RCDVLDTGGAVVGYRQPFT-YEK---NG----WLMLEFS------QRPMPALCKI

YRARSA-S------S-S--PSDNA------TSSSW

>TraesCS1B02G113200.1

MAR---GLIFAPGDADLITIYLQRKISGLPLPPYIHNADVYAAEPAAVP-SAS-G--RWYFFTSV-RQSSRD

TRRCRAVAGGTWHEKA-RCDVLDAGGAVVGYRQPFT-YEK---NG----WLMLEFS------QRPMPALCKI

YRARSA-S------S-S--PSDNA------TSSSW

>TraesCS1B02G192200.1

--TLPPGFRFHPTDDELVGYYLKRRVDNLKIEEVIPVIDLYKCEPWELPEKSFLPKREWFFFVPRDRKYPNG

SRTNRATTTGYWKATGKDRKVSCD-GAVCGVRKTLVFYKGRAPGGERTDWVMHEYRLCQGNFI-GAYALCRV

IKRTEAGLSSSQQSADFTFSGTEASLDENN-----

>TraesCS1B02G277300.1

MA-LPPGFRFHPTDVELVSYYLKRKIMGKKLFEAISEVELYKFAPWDLPKSCL--SKEWFFFCPRDKKYPKG

SRTNRATPNGYWKTSGKDRTIELN--RIVGLKKTLIFHEGKAPKGNRTDWVMYEYKMEDAGFSKDAYVLCKI

FKKSGLGPFAPDDQ-PKELYGD--IGLDDLLPGDF

>TraesCS1B02G285100.1

MG-LPPGYRFYPTEEELVCFYLRHMLDGRR-GRVIPVVDVCSLDPWQLPEVH--RAGEWFYFCARQDREARG

GRPSRTTPSGYWKAAGTPGLVYSA-GRPVGTKKTMVFYRGRAPAGAKTKWKMNEYRAF--EVR--EFSLCRL

YTRSGSLR--P---A--PASALNDRSVQQQ---DW

>TraesCS1B02G285200.1

MA-LPPGYRFYPTEEELVRFYLRHKLDGRR-AHVIPVVDVCSLDPWQLPEVH--RAGEWFYFCARQDREARG

GRPSRTTPSGYWKAAGTPGLVYSA-GRPVGTKKTMVFYRGRAPAGAKTKWKMNEYRAL-PQVR--EFSLCRL

YTSSGNMR--P---T---SSAPNDRPVQQQ---DW

>TraesCS1D02G004800.1

AASLPAAV-FTPTDVELVQDYL-RPIQRLPTKDLC-------SGPHPLPDLP-MG--RKYYLT---D--AL-

AS---CFGYGIWKKT--DMAIRHNG--LVGVKRTMKFP--YTYTGINTNWTM-NYSLNG----MD-LVLCHV

F-------SAPDSKSPN-PPG-DITHLARH---D-

>TraesCS1D02G094300.1

MAR---GLIFAPCDADLVTIYLQRKISGSPLPRYIHDADVYAAEPAALP-SAS-G--RWYFFTSV-RQSSRG

TRRCRAVAGGTWHEKA-RCDVLDAGGAAVGYRQPFTYEK---NG-----WLMLEFS------QRPMPALCKI

YRARSA-S------S-S--PSDNA------TSSSW

>TraesCS1D02G194200.2

--TLPPGFRFHPTDDELVGYYLKRRVDNLKIEEVIPVIDLYKCEPWELPEKSFLPKREWFFFVPRDRKYPNG

SRTNRATTTGYWKATGKDRKVSCD-AAVCGVRKTLVFYKGRAPGGERTDWVMHEYRLCQGNFI-GAYALCRV

IKRTEAGLSSSQQSADFAFSGTEASLDENN-----

>TraesCS1D02G261800.1

MSDVMPGFRFHPTEEELIEFYLRRKVEGRRFNELITFLDLYRFDPWELPAMAVIGEKEWFFYVPRDRKYRNG

DRPNRVTASGYWKATGADRMIR--NSRPIGLKKTLVFYSGKAPKGVRSSWIMNEYRL--PLFYSE--SLCRV

YKRS----ARPVSPAP--PPSA--DELSRLLPSDW

>TraesCS1D02G263800.1

MSQLPPGFRFHPTDEELVMHYLCRRCAGLPISPIIAEVDLYKFDPWQLPRMALYGEKEWYFFSPRDRKYPNG

SRPNRSAGTGYWKATGADKPVGT--PKPLAIKKALVFYAGKAPKGDKTNWIMHEYRLADSSLRLDDWVLCRI

YNKKGASE--PASS-PDSMPADSC--ISEWVNPDL

>TraesCS1D02G266500.1

MA-LPPGFRFHPTDVELVSYYLKRKIMGKKLFEAISEVELYKFAPWDLPKSCL--SKEWFFFCPRDKKYPKG

SRTNRATPNGYWKTSGKDRTIELN--RIVGLKKTLIFHEGKAPKGNRTDWVMYEYKMEDAGFSKDAYVLCKI

FKKSGLGPFAPEDQ-PKELYGD--IGLDDLLPGDF

>TraesCS1D02G275500.1

MG-LPAGYRFYPTEEELVRFYLRHMLDGRR-GRVIPVADVCSLDPWQLPEVH--RAGEWFYFCARQDREARG

GRPSRTTPSGYWKAAGTPGLVYSA-GRPVGTKKTMVFYRGRAPAGAKTKWKMNEYRAF--QVR--EFSLCRL

YTRSGSLR--P---A--PASALNDRSVQQQ---DW

>TraesCS2A02G053500.1

MC-LGPTEDYSWSDEELVRF-LERK-ADDSLPNVVG--DLSLIHPQD-P-----G--NWYLNQSDDQPYGNG

EDIRKA-KGGYWKPTSK----------STGVKFSLEFYEGEAPSGKRTQWLMHEYQVEQ-EANQEYKSLCTI

LKESTNAPNALDGAAP-SPPAMDQSHVEQSSSS-Y

>TraesCS2A02G063200.1

MEELMPGFRFHPTDEELVSFYLKKKIQQKPISELIRQLDIYKFDPWDLPKLASTSETESYFYCPRDRKYRNS

ARPNRVTAAGFWKATGTDRPIYS-DTRCVGLKKSLVFYKGRAARGVKTDWMMHEFRL--PKSRIDSWTICRI

FKKTSSMATLPQQQSP-QFG--DQ--LGRLLGA-W

>TraesCS2A02G063300.1

MEELMPGFRFHPTDEELVSFYLKKKIQQKPISELIRQLDIYKFDPWDLPKLANTGETEWYFYCPRDRKYRNS

ARPNRVTAAGFWKATGTDRPIYS-GTRCVGLKKSLVFYKGRAARGIKTDWMMHEFRL--PK-RIDSWTICRI

FKKTSSMATAPQQQSP-QFG--DQ---GRLLGA-W

>TraesCS2A02G063400.1

MEELLPGFRFHPTDEELVSFYLKKKIQQKPISELIRQQDIYKFDPWDLPKLASTSETEWYFYCPRDRKYRKS

TRPNRVTATGFWKATGTDRPIYS-GT--------------------------------------------RI

FKKTSSMAVAP---SP------DQ---ERLLVE-W

>TraesCS2A02G101000.1

MSALPPGFRFHPTDEEVVTHYLTRKVLRS-FSQVIADVDLNKTEPWDLPGKAKMGEKEWFFFVHKGRKYPTG

TRTNRATEKGYWKATGKDKEIFRGDAVLVGMKKTLVFYTGRAPSGGKTPWVMHEYRLE-PTAK-DDWAVCRV

FNKD---LDLPDSPSPQFMPGGDQASSSA--LDSW

>TraesCS2A02G101100.1

MSALPPGFRFHPTDEEVVTHYLTRKVLRS-FSQVITDVDLNKNEPWELPGKAKMGEKEWFFFVHKGRKYPTG

TRTNRATKKGYWKATGKDKEIFRGDAVLVGMKKTLVFYTGRAPSGGKTPWVMHEYRLE-PTAK-DDWAVCRV

FNKD---LDLPDSPSPQFMPGGDQASSSA--LDSW

>TraesCS2A02G101200.1

MSALPPGFRFHPTDEEVVTHYLTPKIRND-FSLMVAYVNLNNTEPWDLPKKAKMGETEWFFFVHKDRKYPTG

TRTNRATKSGYWKATGKDKEIFRGDAVLVGMKKTLVFYRGRAPGGQKTSWVMHEYRLE-PSAK-DDWAVCRL

FNKE---LDPPDYPSPQ-----------------W

>TraesCS2A02G101400.1

MSSLPPGFRFHPTDEEVVTHYLTPKAVNA-FSLVIADVDLNKTEPWDLPGKAKMGEKEWYFFVHKDRKYPTG

TRTNRATEKGYWKATGKDKEVFRGDAVLVGMKKTLVFYTGRAPRGDKTPYVMHEYRLE-PSAR-NDWAVCRV

FDKD---LDLPDSPSPQFMPGGEQASSSAL-FDSW

>TraesCS2A02G101900.1

MASLPPGFRFHPTDEELILHYLRNRAAAAPCPPIIADVDIYKFDPWDLPSQAVYGDCEWYFFSPRDRKYPNG

IRPNRAAGSGYWKATGTDKPIHDATGQGVGVKKALVFYKGRPPKGTKTAWIMHEYRLAAASMRLDDWVLCRI

YKKTGLAS-VPYACLPEHVVYAEE-------SAGL

>TraesCS2A02G102000.1

MASLPPGFRFHPTDEELILHYLRNRAAAAPCPSIIADVDIYKFDPWDLPSQAVYGDCEWYFFSPRDRKYPNG

IRPNRAAGSGYWKATGTDKPIHDPTGQGVGVKKALVFYKGRPPKGTKTAWIMHEYRLAAASMRLDDWVLCRI

YKKTGLAS-VPDACMPEHGVYAEE-------SASL

>TraesCS2A02G201800.2

MRDLPPGFRFHPTDEELVVHYLKKKAAKVPLPTIIAEVDLYKFDPWELPEKATFGEQEWYFFSPRDRKYPNG

ARPNRAATSGYWKATGTDKPILASGREKVGVKKALVFYRGKPPKGLKTNWIMHEYRLTD-SLRLDDWVLCRI

YKKINKAA---DATM-GAGALASSNR-SR-LSSDL

>TraesCS2A02G306800.1

MANLPPGFHFFPSDEELVIHFLRRKAAL----DIVPTLPQNRYDPWELN--A-LG--QWYFFSQAT----Q-

---SRASRNGCWNPVGADEAVSSG-H--VGLKKTLVFSIGEPFQATKTNWVMHEYHLLDASHK-ENWVVCRV

FESSYDSQ--------E-------DDYDEV-----

>TraesCS2A02G311500.1

MA-VPPGFRFHPTDEELLLYYLKKKIGFEKFDEVIREVDLNKIEPWDLQERCRIGAPEWYFFSHKDRKYPTG

SRTNRATTAGFWKATGRDKCIRTS--RKIGMRKTLVFYRGRAPHGQKSDWIMHEYRLEET--SEDGWVVCRV

FKKKCFFKGGGDHD--QGLPPGAEQPMDGF---EW

>TraesCS2A02G325900.2

FN-LPAGVKFDPTGLELLE-HLEGKVAP--HDDFIPTTEICYTHPENLP-----GKMRNHFFHK--NAYDVG

QRKRRKNSDVRWHKTGKSKKIL--G--VIGWKKILVLMKDG-ITSRTTNWTMHQYHLGV----QDELVVSKV

FKKQSVLVYPPSPRAPS-----EDSCLDERLQGDW

>TraesCS2A02G326300.2

---LPIGVKFDPTDLELLG-HLEGKIAV--HVDFIPTIEICYTHPENLP-----GKLISHFFHK--NAYDVG

TRKRRKSNDERWHKTGKSRHIL--G--DIGWKKIMVL-KGG-AKAEKTNWTMHQYHLGV----ENELVVCKV

FEKQSAAVYPLSPEF-E---------FDEHLQGDW

>TraesCS2A02G326400.2

HL-LPIGVKFDPTDLELLG-HLEGKIAM--HVDFIPTIEICYTHPENLP-----GRLVSHFFHK--NAYDVG

TRKRRKNSDERWHKTGKSRHIL--G--DIGWKKIMVL-KGG-YKAEKTNWTMHQYHLGV----ENELVVCKV

FEKQSVAVYAPSPEF-E-----DDPALDEHLQGDW

>TraesCS2A02G326500.1

FF-LPAGVKFDPTDLELLE-HLEGKVAA--HVDFIPTIGICYTHPENLP-----GKMSGHFFHK--NAYVVG

KRKRRKNSDERWHKTGKSRSIL--G--VIGWKKILVLYKGG-GKTEKTNWRMHQYHLGV----QDELVVSKV

FMNQSLGVYPLSPGS-E-----EDSGLDEHLQEDW

>TraesCS2A02G326600.1

FN-LPAGVKFDPTGLELLE-HLEGKVAP--HNDFIPTIEICYTHPENLP-----GKMRSHFFHK--NAYDVG

QRKRRKNSDVRWQKTGKSKEIS--G--VIGWKKILVLMKDG-ITSRTTNWTMHQYHLGV----QDEPVVSKV

FKKQPVPVYPPSPRAPS-----ENSGLDECLQGDW

>TraesCS2A02G328100.1

MAGLRAGAVFRPSGRQLITLYL-RNLRGRAAP--VAGVDIFAASPDALPHR-K-G--EWYFFAAKPMRAP-G

---------GWWMQYGDAKGYEFR--EVYARHRRFEFRADGG--KVET-WLMKEYALHKP-----DLVVYKV

FTK-----PPPEA-A-------DSIDLNN------

>TraesCS2A02G328200.1

MANLTAGYVFQPTGRELIDHYL-PRLGG--FP--IEGVDVLSLPPRELPHI-R-G--EWFFFAAKSTPTP-G

A-------GGCWVQYGTEKPY-YGGREAVAFRRRFAYRKG-----ASA-WLMKEYRLNRP-----VFVVHKV

YRK-----PPPD--S-------EE-----------

>TraesCS2A02G328300.1

MANLTAGYVFQPTGLELIDHYL-PRLGG--FP--INGVDVLSLPPRELPHI-R-G--EWFFFAAKLTPTP-G

A-------GGCWVQYGTEKPY-YGGREAVAFRRRFAYRKG-----AST-WLMKEYRLNRP-----VFVVHKV

YRK-----PPPD--S-------EE-----------

>TraesCS2A02G328400.1

MANAAGGYVFQPTGCELVGYYL-PRLGG--LP--IADVDVLSLRPRVLSHR-R-G--EWFFFAAKPTPTP-G

A-------GGCWEQYGQEKAY-YDSREAVALRCRFAYRRG-----EST-WRMKEYWLNRP-----VFVVHKV

YRK-----PPPD--S-------DEPQLDE------

>TraesCS2A02G328500.1

MASLPGGMRFDPNGRCLVRTYL-PKIRG--IP----DVDVYGTRPEALPNR-G-G--KWHFFTTRPAGGP-G

S---RVV-GGLWVRYDEEKAY---DGDVLGYRCRFAFH------GEKT-WRMKEFRLNEP-------VAWKV

YND-----EEPDDG--E-----DDDDLTQ------

>TraesCS2A02G338300.1

--DLPPGFRFHPTDEELITHYLAKKVADR-FALAVSVADLNKCEPWDLPSLARMGEKEWYFFCLKDRKYPTG

LRTNRATESGYWKATGKDKDIFRG--ALVGMKKTLVFYTGRAPKGDKSGWVMHEYRIN-SSSR-NEWVLCRV

FKKS---L-LPDMST--FNPGLDMGERERLLTSEW

>TraesCS2A02G354300.1

MAVLPPGHAFEPSGHQIITQYL-PKLRG--TP--VAGVDVFAASPPALPSR-R-G--EWYFYGAQAAPAP-G

---------GCWARYGREKGYVHGAAEAVAFRSRFAFHKCGG---AAT-WLMKEYRLNKP-----DCVVRKI

FTK-----PPPDE-S-D-----ED-SIEG------

>TraesCS2A02G363700.1

LKDLPPNSRFDPALDAIVARYLRRQIAGKLYPQVVQAVDVFAAHPAVLP--A--ADREWFFAAHRRRELENK

ARPRRA-GPGYVPREGR----------VVGIGYRRVFY--RVRRVSRTEWWMEEYGFGKP---EDELVVYKL

CRR--------------------------------

>TraesCS2A02G382400.1

--TLPPGFRFYPSDQELVCHYLCKKVTN-RASGTLVEVDLHAREPWELPDVAKLTASEWYFFSFRDRKYATG

SRTNRATKTGYWKATGKDREVRSPTRAVVGMRKTLVFYQGRAPNGSKTSWVMHEFRLD-PSHVQEDWVLCRV

FQKQK---SSP---S--APP--DQSVSPTMF--GW

>TraesCS2A02G386300.1

MNHVPPGFRFHPTDEELVDYYLRKKVQLKRIDDIIKDVDLYKIEPWDLQERCKIGEDDWFFFSHKDKKYPTG

TRTNRATTAGFWKATGRDKPIYV-C--LVGMRKTLVFYKGRAPNGQKLDWIMHEYRLETA---DEGWVVCKV

FKKRVA--AF-DHA-H-QLPPGQPRDLDKFSTPDW

>TraesCS2A02G409900.1

---KPVGFRFAPTDEELVEYYL-PRLQGQHVPAIIQ--NVYQCHPDDL-GK-K-GQ-NWFFLTSRTRKYVNG

GRPARTTGRGRWKSTGTT--------EVVGKESGLAYHEG--PIKTKTKWLMHEYKLDKP---LDEYVMCRI

YKRDEAGPKRPDGQTP-AAPPSMQKQQQPYVPAD-

>TraesCS2A02G459700.1

VYNLPPGYHFAPSGDELIVHYLRRKLDG--LPPIFNDVPIIDYRPEQI-DE-RFGAR-WYFFTKRTRKYATG

SRPDRTPGNGFWKATGPVREIPCP-GKLVGRARTLVFYTG--PEK-PTHWTMYEYNRTS-NDKVDEWVLCTI

Q--EAGG-EVPDDE-PTVAPNN--------IPCTW

>TraesCS2A02G462500.1

SSRLPPGFRFHPRDHELVLDYLCHKLSG----VDMVDVDLNKCEPWELPDAACVGGKEWYFFSRHDRKYATG

QRTNRATHTGYWKATGKDRVIT--AAAVVGMRKTLVFYLGRAPRGTKTEWVMHEFRVE-AL-E-EDWVLCRV

FYKSTTAAPVAD-G--A-------PKPE--VPQ-W

>TraesCS2A02G561700.1

MA-RP-GFRFHPTDQELVSFYLRRKVLGHGGC-FIPEVDLYKLQPHHLPGKS-S-STEWYLFAPRGRKYPTG

LRMERATPRGFWKSTGKDRPVMHK-I-VVGMKKTLVFHIGKAPSGTRTDWVMHEYRLHG----QDTYALCRV

FNKNMAS-FVPGGKPPDANA----EGEKQFQPMNW

>TraesCS2A02G561800.1

MAPRP-GIRFYPTDQELVGWLLRSKVLGHAGLDFIPVVNAYKFEPQELPDKS-S-STESYFFAPRGRKYPTG

LRMKRATENGFWKSTGKDHPIMHN-T-IIGMKKTLVFHAGRAPKGTRTDWVMHEYRLHG-CN-QDTYALCRV

FNKNTVT-FLPEGK-PD-------KGGEQFQPLDW

>TraesCS2A02G565900.1

MDCVPPGFRFHPTEVELVGYYLARKVAAQKIEDIIQEVDLYRIESWDLQGRC---LSEWYFFSFKDRKYPSG

TRTNRATAAGFWKAIGRDKRVTSSG--VMGMRKTLVLFRTPAPNGRKTDWIIHEYRRQNPT-QEEGWVVCRA

FQKPIPNQPAP------------------------

>TraesCS2A02G566000.1

--CVPPGFRFHPTEEELVGYYLARKVAAQTIDDIIQEVDLYRIEPWDLQDRC-VGQSEWYFFSFKDRKYPSG

TRTNRATAAGFWKATGRDKPVTSSG--VIGMRKTLVFYQGRAPNGRKTDWIIHEYRLQTPT-QEEGWVVCRA

FQKPTPNQ---DAP-G-NIPPPGDNFLDSL---H-

>TraesCS2A02G566100.1

MECVPPGFRFHPTEEELVGYYLARKVAAQKIDDIIQEVDLYRIEPWDLQERC-GGEQEWYFFSFKDRKYPSG

TRTNRATAAGFWKATGRDKPVTSSG--VIGMRKTLVFYRGRAPNGRKTDWIIHEYRLQTPT-QEEGWVVCRA

FQKPTPNQATPDAP-G-NIPPPGDNFLDSL---Q-

>TraesCS2A02G566200.1

MECVPPGFRFHPTEEELVGYYLARKVAAQKIDDIIQEVDLYRIEPWDLQEKC-GGQSEWYFFSFKDRKYPSG

TRTNRATAAGFWKATGRDKPVMSSG--VTGMRKTLVFYRGRAPNGRKTDWIIHEYRLQTPT-QEEGWVVCRA

FQKPAPNQAAPNAP-G-NIPPPGDNFLDSL---Q-

>TraesCS2A02G566300.1

MDCVPPGFRFHPTEEELVGYYLARKVAAQNIDGIIQEVDLYRTEPWDLQEKC-GGQSEWYFFSFKDRKYPSG

TRTNRATAAGFWKATGRDKPVTSSS--VIGMRKTLVFYRGRAPNGRKTDWIIHEYRLQTPT-QEDGWVVCRA

FQKPTPNQTALDAP-G-NIPPPSENFLDIL---NY

>TraesCS2A02G566400.1

M-CVPPGFRFHPTEEELVGYYLAQKVAAQKIDDIIPEVDLYRIEPWDLQERC-GGQSDWYFFSFKDRKYPSG

TRTNRATAAGFWKATGRDKPVMSSG--VIGMRKTLVFYRGRAPNGRKTDWIIHEYRLQTPT-QEEGWVVCRA

FQKPTPNQAAPDAS---NIPPPGDNFLDSL-----

>TraesCS2B02G067400.1

MC-LGPTENYSWSDEELVRF-LERK-AEDSLPNVVG--DLTLIHPLD-P-----G--NWYLNQSDDQPYGNG

EDIRKA-KGGYWKPTSK----------STGVKFSLEFYEGEAPSGKRTQWLMHEYQVEQ-EAKQEYKSLCTI

FKESTNAPNALDGAAP-SPPAMDQSHVEQSSSS-Y

>TraesCS2B02G075900.1

MEELMPGFRFHPTDEELVSFYLKKKIQQKPISELIRQLDIYKFDPWDLPKLASTGETEWYFYCPRDRKYRNS

ARPNRVTAAGFWKATGTDRPIYS-DTRCVGLKKSLVFYKGRAARGIKTDWMMHEFRF--PKSRIDSWTICRI

FKKTSSMAALPQQLSP-QSG--DQ--LGRLLGA-W

>TraesCS2B02G076000.1

MEELMPGFRFHPTDEELVSFYLKKKIQQKPISELIRQLDIYKFDPWDLPKLANTGETEWYFYCPRDRKYRNS

ARPNRVTAAGFWKATGTDRPIYS-GTRCVGLKKSLVFYKGRAARGIKTEWMMHEFRL--PK-RIDSWTICRI

FKKTSSMAAAPQQQS-------DQ---GRLLGA-W

>TraesCS2B02G076100.1

MEELLPGFRFHPTDEELVSFYLKKKIQQKPISELIKQQDIYKFDPWDLPKLASTSETEWYFYCPRDRKYHNS

ARPNRVTAAGFWKATGTDRPIYS-GTRCVGLKKSLVFYKGRAARGIKTDWMMHEFRL--PK-RIDSWTICRI

FKKPGSMAVAP---SP-HFG--DQ---GRLLVA-W

>TraesCS2B02G100600.1

MV-LTRGYKLEPHDDEAVEYYLLRRLLG-PLE-IILEDDPLSAPPWEL---NK--ELDAFFFAHGQT---DG

SRNKRTCGGGCWEGQKVDKELI-PS----GKKYQLNF-NGK-----STGWVMHEYSVTAP-----SLRLYRI

LKKKREGGRSSDG-APGELP----------RPN--

>TraesCS2B02G118200.1

MSALPPGFRFHPTDEEVVTHYLTRKVLRS-FSQVITDVDLNKNEPWELPGLAKMGEKEWFFFVHKGRKYPTG

TRTNRATKKGYWKATGKDKEIFRGDAVLVGMKKTLVFYTGRAPSGGKTPWVMHEYRLE-PTAK-DDWAVCRV

FNKD---LDLPDSPSPQFMPGGDQASSSAL-LDSW

>TraesCS2B02G118300.1

MSALPPGFRFHPTDEE---------VLRS-FSQVITDVDLNKNEPWELPGLAKMGEKEWFFFVHKSRKYPTG

TRTNRATKNGYWKATGKDKEIFRGDAVLVGMKKTLVFYTGRAPSGGKTPWVMHEYRLE-PTAK-DDWAVCRV

INKD---LDLPDSQSPQFMPGGDQASSSAL-LDSW

>TraesCS2B02G118400.1

MSALPPGFRFHPTDEEVVTHYLTRKVLRS-FSQVITDVDLNKNEPWELPGLAKMGEKEWFFFVHRGRKYPTG

TRTNRATKNGYWKATGKDKEIFRGDAVLVGMKKTLVFYTGRAPSGGKTPWVMHEYRLE-PTAK-DDWAVCRV

INKD---LDLPDSPS-QFMPGGDQASSSAL-LDSW

>TraesCS2B02G118500.1

MSSLPPGFRFHPTDEEVVTHYLTPKAVNA-FSLVIADVDLNKTEPWDLPGKAKMGEKEWYFFVHKDRKYPTG

TRTNRATEKGYWKATGKDKEIFRGDAVLVGMKKTLVFYTGRAPRGDKTPYVMHEYRLE-PSAK-NDWAVCRV

FDKD---LDLPDSPSPQFMPGGDQASSSAL-FDSW

>TraesCS2B02G119000.1

MASLPPGFRFHPTDEELILHYLRNRAAAAPCPPIIADVDIYKFDPWDLPSQAVYGDCEWYFFSPRDRKYPNG

IRPNRAAGSGYWKATGTDKPIHDATGQGVGVKKALVFYTGRPPKGTKTAWIMHEYRLAAASMRLDDWVLCRI

YKKTGLAS-VPDACMPEHVVYSEE-------SAGL

>TraesCS2B02G228900.1

MRELPPGFRFHPTDEELVVHYLKKKAAKVPLPTIITEVDLYKFDPWELPEKATFGEQEWYFFSPRDRKYPNG

ARPNRAATSGYWKATGTDKPILASGREKVGVKKALVFYRGKPPKGLKTNWIMHEYRLTD-SLRLDDWVLCRI

YKKINKAA---DATM-GAGPLASSNR-SR-LSSDL

>TraesCS2B02G323500.1

MTNLPPGFHFFPSDEELVIHFLRRKAAL----DIVPTLPQNRYDPWELN--A-LG--QWYFFSQAT----Q-

---SRTSRNGCWNPIGADEAVSSG-H--VGLKKTLVFSIGEPFQATKTNWVMHEYHLLDTSHK-ENWVVCRV

FESSYDSQ--------E-------DDYDEV-----

>TraesCS2B02G343600.1

--DLPPGFRFHPTDEELITHYLAKKVADR-FALAVSVADLNKCEPWDLPSLARMGEKEWYFFCLKDRKYPTG

LRTNRATESGYWKATGKDKDIFRG--ALVGMKKTLVFYTGRAPKGDKSGWVMHEYRLN-SSSK-NEWVLCRV

FKKS---L-LPDMST--FNPGLDMGERERLLTSEW

>TraesCS2B02G353800.1

MAVLPLGYVFRPKARELIQHYL-PKLGG--TP--VAGVDVFSAAPDALPHR-R-G--EWYFFAAHPRPAP-G

---------GCWITYGPEKAY--RGGEAVAFRRNLAYYRGGG--DGAT-WLMAEYRLNKP-----DCVVRKV

FMK-----PPPDDSS-A-----E------------

>TraesCS2B02G353900.1

MANLTAGYVFQPTGRELIDHYL-PRLGG--FP--IEGVDVLSLPPSELPHI-R-G--EWFFFAAKPRPTP-G

A-------VGCWVQYGTEKPY-YGGREAVAFRRRFAYRKG-----AST-WLMKEYRLNRP-----VFVVHKV

YRK-----PPPD--S-------EE-----------

>TraesCS2B02G355300.2

FN-LPVGVKFDPTGLELLE-HLEGKVAP--HDDFIPTIEICYTHPENLP-----GKMTGHFFHQ--NAYDVG

QRKRRKNSDVRWHKTGKSKQIS--G--VIGWKKILVLRKDG-ITSHKTNWTMHQYHLGV----QHELVVSKV

FEKQPVPVYPPSQRAPS-----EDFGLDECLQGDW

>TraesCS2B02G355400.1

FF-LPAGVKFDPTDLELLE-HLEGKVVA--HVDFIPTIKICYTHPENLP-----GKMSGHFFHK--NAYVVG

KRKRRKNSDERWHKTGKSRSIL--G--VIGWKKILVLYKGG-GKTEKTNWRMHQYHLGV----QDELVVSKV

FMNQSLGVYPLSPGS-E-----DDSGLDEHLLGDW

>TraesCS2B02G355500.1

FF-LPAGVKFDPTDLELLE-HLEGKVAA--HVDFIPTIEICYTHPENLP-----GKMSGHFFHK--NAYVVG

KRKRRKNSDERWHKTGKSRSIL--G--VIGWKKILVLYKGG-GKTEKTNWRMHQYHLGV----QDELVVSKV

FMNPSA---PLSPGS-E-----EDSGLDEHLQGDW

>TraesCS2B02G355600.1

ME-LPTGVKFDPTDLQLLG-HLEGKIAV--HVDFIPTIEICYTHPENLP-----GKSTSHFFHK--NAYDVG

TRKRRKNSDERWHKTGKSRHIL--G--DIGWKKIMVL-KGG-AKAEKTNWTMHQYHLGV----ENELVVCKV

FEKQSVAVYPPSPEF-E-----DDPGLDEHLQDDW

>TraesCS2B02G355700.1

ME-LPIGVKFDSTDLELLG-HLEGKIAV--HVDFIPTIEICYTHPENLP-----GKLISHFFHK--NAYDVG

TRKRRKSNDKRWHKTGKSRHIL--G--DIGWKKIMVL-KGG-AKAEKTNWTMHQYHLGV----ENEMYVC--

----------------S------------------

>TraesCS2B02G359200.1

MANAEAGYVFQPTGRELVGHYL-PRLGG--LP--IEGVDVLSLRPRALSHR-R-G--EWFFFAAKPTPTP--

---------GCWVQYGQEKAY-YGGREAVAFRRRFAYRRG-----EST-WRMKEYRLNRP-----VFVVHKV

YRK-----PRPD--S-------DEPRLDE------

>TraesCS2B02G375000.1

MAALPPGHAFEPSGHQIITQYL-PKLRG--TP--VAGVDVFSASPAALPSR-R-G--EWYFYGAQAAPAP-G

---------GCWARYGREKGYVHGAAEAVAFRRRFAFHQCGR---AAT-WLMKEYRLNKP-----DCVVRKI

FTK-----PPTDE-S-D-----ED-SIEG------

>TraesCS2B02G376900.1

---IGLGLRFNPTDQELME-RLEAKVGGRSDPKFIPTIE--YPHPQQLP-----GTMTKHFFCR--RAFESG

RRKHRKAENDAWHKTGKSMALK--G--RLGWKNILVLYK--------TNWVMHEYRLSD----EDELVLCKI

FKR-------------------PTGGMDV------

>TraesCS2B02G381700.1

--ELPPKSRFDPTPDVIVGRYLRRQIAGKLYTQVVQATDVFAAHPAVLP--A--ADREWFFAVHRRRELENK

ARPRRA-GPGYVLREGR----------VVGIGYRRVFY--RVRRVSRTEWWMEEYGFRKP---EDELVVYKL

YRR--------------------------------

>TraesCS2B02G399700.1

--TLPPGFRFYPSDQELVCHYLYKKVTN-RASGTLVEVDLHAREPWELPDVAKLTASEWYFFSFRDRKYATG

SRTNRATKTGYWKATGKDREVRSPTRAVVGMRKTLVFYQGRAPNGSKTSWVMHEFRLD-PTCRQEDWVLCRV

FQKQK---SSP---S--VAP--DQGSSPMMF--GW

>TraesCS2B02G403300.1

MNHVPPGFRFHPTDEELVDYYLRKKVQLKRIDDIIKDVDLYKIEPWDLQERCKIGEDDWFFFSHKDKKYPTG

TRTNRATTAGFWKATGRDKPIYV-C--LVGMRKTLVFYKGRAPNGQKLDWIMHEYRLETA---DEGWVVCKV

FKKRVA--PF-DHA-H-QLPPGQPRDLDKFSTPDW

>TraesCS2B02G481400.1

--NLPPGYHFTPTEAELIVHYLRRKLDG--LPPIFNDVPITDYRPEQI-DE-RFGGR-WYFFTKRTRKYATG

SRPDRTPGKGFWKGTGPVREIPNP-GKLVGHARTLVFYTG--PDE-PTYWTMYEYNHTS-NDKIDEWVLCTI

---KAGG-EVPDDE-PTVAPNS--------IPCTW

>TraesCS2B02G484100.1

SSRLPPGFRFHPRDHELVLDYLCHKLSG----VDMVDVDLNKCEPWELPDAACVGGKEWYFFSRHDRKYATG

QRTNRATHTGYWKATGKDRVIT--AAAVVGMRKTLVFYLGRAPRGTKTEWVMHEFRAE-AL-E-EDWVLCRV

FYKSTTAAPIAD-S--A-------PKPE--MPQ-W

>TraesCS2B02G616900.1

ME-LPPGYRFHPTDVELTLYYLKRKLLGKKLLNAVAEVDIYKHAPWDLPKSSM--TGQWYFFCTRGRKYSVG

QRANRSTEGGYWKATGKDRQVVYE--RTVGMKRTLVFHAGKAPKGTRTDWVMYEYRLVQAGVRLDDSVLCKV

HKKSGPGPLSPAAH-LDVVPCD--MELNDLVSNDV

>TraesCS2B02G627000.1

MDCVPPGFRFHPTEEELVGYYLARKVAAQTIDDIIQEVDLYRIEPWDLQERC-SGQSEWYFFSFKDRKYPSG

TRTNRATAAGFWKATGRDKPVTSSG--VIGMRKTLVFYRGRAPNGRKTDWIIHEYRLQTPT-QEEGWVVCRA

FQKPAPNQAAPDAP-G-NIPPPGDNFLDSL---H-

>TraesCS2B02G627100.1

--CVPPGFRFHPTEEELVGYYLARKVAAQKIDDIIQEVDLYRIEPWDLQERCGGGQSEWYFFSFKDRKYPSG

TRTNRATAAGFWKATGRDKPVISSG--VIGMRKTLVFYRGRAPNGRKTDWIIHEYRLQTPT-QDEGWVVCRA

FQKPAPNQAAPDAP-G-NIPPQGDNFLDSL---H-

>TraesCS2B02G627200.1

MECVPPGFRFHPTEEELVGYYLARKVAAQKIDDIIQEVDLYRIEPWDLQERC-GGEQEWYFFSFKDRKYPSG

TRTNRATAAGFWKATGRDKPVMSSG--VIGMRKTLVFYRGRAPNGRKTDWIIHEYRLQTPT-QEEGWVVCRA

FQKPTPNQAAPDPP-G-NIPPPGDNFLDSL---Q-

>TraesCS2D02G061500.1

MEELMPGFRFHPTDEELVSFYLKKKIQQKPISELIRQLDIYKFDPWDLPKLASTGETESYFYCPRDRKYRNS

ARPNRVTAAGFWKATGTDRPIYS-GTRCVGLKKSLVFYKGRAARGIKTDWMMHEFRL--PKSRIDSWTICRI

FKKTSSMAALPQQQSP-QFG--EQ---GRLLGA-W

>TraesCS2D02G061600.1

MEELLPGFRFHPTDEELVSFYLKKKIQQKPISELIMQQDIYKFDPWDLPKLASTSETEWYFYCPRDRKYRNS

ARPNRVTAAGFWKATGTDRPIYS-GTRCVGLKKSLVFYKGRAARGIKTDWMMHEFRL--PK-RIDSWTICRI

FKKTGSMAVAP---SP-HFG--DQ---GRLLVE-W

>TraesCS2D02G083700.1

MV-LTRGYKLEPLDEEAVEYYLLRRLLG-PLE-IILEDDPLSAPPWEL---NK--ERDAFFFAYGQT---DE

SRNKRTCGGGCWEGQKVDKELI-PS----GKKYQLNF-KGK-----STGWVMHEYSVTAP-----SLRLYRI

LKKKRESG-SPDC-APGELP----------RPN--

>TraesCS2D02G100600.1

MSVLPPGFRFHPTDEEVVTHYLTRKVLRS-FSQVIADVDLNKTEPWDLPGKAKMGEKEWFFFVHKGRKYPTG

TRTNRATEKGYWKATGKDKEIFRGDAVLVGMKKTLVFYTGRAPSGGKTPWVMHEYRLE-PTAK-DDWAVCRV

INKD---LDLPDSPSSQFMPGGDQASSSAL-LDSW

>TraesCS2D02G100700.1

MSALPPGFRFHPTDEEVVTHYLTRKVLRS-FSQVIADVDLNKTEPWDLPGKAKMGEKEWFFFVHKGRKYPTG

TRTNRATEKGYWKATGKDKEIFRGDAVLVGMKKTLVFYTGRAPSGGKTPWVMHEYRLE-PTAKQDDWAVCRV

INKD---LDLPDSPSSQFMPGGDQASSSAL-LDSW

>TraesCS2D02G100800.1

MSALPPGFRFHPTDEEVVTHYLTPKIRND-FSLMVSYVNLNNTEPWDLPKKAKMGQSEWFFFVHKDRKYPTG

TRTNRATKSGYWKATGKDKEIFRGGAVLVGMKKTLVFYRGRAPGGQKTPWVMHEYHLE-PSAK-DDWAVCRL

LNKD---MDPPDSPSPQ-----------------W

>TraesCS2D02G100900.1

MSSLPPGFRFHPTDEEVVTHYLTPKAVNA-FSLVIADVDLNKTEPWDLPGKAKMGEKEWYFFVHKDRKYPTG

MRTNRATEKGYWKATGKDKEIFRGDAVLVGMKKTLVFYTGRAPRGDKTPYVMHEYRLE-PSAK-NDWAVCRV

FDKD---LDLPDSPSPQYMPGGDQASSSAL-FDSW

>TraesCS2D02G101300.1

MASLPAGFRFHPTDEELILHYLRNRAAAAPCPPIIADVDIYKFDPWDLPSQAVYGDCEWYFFSPRDRK----

---------------------------ASAVKKALVFYTGRPPKGTKTAWIMHEYRLAAASMRLDDWVLCRI

YKKTGLAS-VPNACMPEHVVYAEE-------PAGL

>TraesCS2D02G101400.1

MASLPPGFRFHPTDEELILHYLRNRAAAAPCPPIIADVDIYKFDPWDLPSQAVYGDCEWYFFSPRDRKYPNG

IRPNRAAGSGYWKATGTDKPIHDATGQGVGVKKALVFYKGRPPKGTKTAWIMHEYRLAAASMRLDDWVLCRI

YKKTGLAS-VPDACMPEHGVYAEE-------SAGL

>TraesCS2D02G214100.1

MRELPPGFRFHPTDEELVVHYLKKKAAKVPLPTIIAEVDLYKFDPWELPEKATFGEQEWYFFSPRDRKYPNG

ARPNRAATSGYWKATGTDKPIMASGREKVGVKKALVFYRGKPPKGLKTNWIMHEYRLTD-SLRLDDWVLCRI

YKKINKAA---DATM-GAGALASSNR-SR-LSSDL

>TraesCS2D02G305300.1

--NLPPGFHFFPSDEELVIHFLRRKAAL----DIVPTLPQNRYDPWELNGKA-LG--QWYFFSQAT----Q-

---SRTSRNGCWNPIGADEAVSSG-H--VGLKKTLVFSIGEPFQATKTNWVMHEYHLLDTSHK-ENWVVCRV

FESSYDSQ--------E-------DDYDEV-----

>TraesCS2D02G309800.1

MA-VPPGFRFHPTDEELLLYYLKKKIGFEKFDEVIREVDLNKIEPWDLQERCRIGAPEWYFFSHKDRKYPTG

SRTNRATTAGFWKATGRDKCIRTS--RKIGMRKTLVFYRGRAPHGQKSDWIMHEYRLEET--SEDGWVVCRV

FKKKCFFKGAGDHD--QGLPPGAEQPMDGF---EW

>TraesCS2D02G324700.1

--DLPPGFRFHPTDEELITHYLAKKVADR-FALAVSVADLNKCEPWDLPSLARMGEKEWYFFCLKDRKYPTG

LRTNRATESGYWKATGKDKDIFRG--ALVGMKKTLVFYTGRAPKGDKSGWVMHEYRLN-SSSR-NEWVLCRV

FKKS---L-LPDMST--FNPGLDMGERERLLTSEW

>TraesCS2D02G334200.1

MAVLPPGYVFRPKARELIQHYL-PKLGG--TP--VAGVDVFSAAPDALPHR-R-G--EWYFFAAHPRPAP-G

---------GCWIPYGPEKAY--RAGEAVAFRRRLAYYRGDG--DGAT-WLMAEYRLNKP-----DCVVRKV

FTK-----PPPDDSS-A-----EAETWDGR-----

>TraesCS2D02G334300.1

MANLTAGYVFQPTGRELIHHYL-PRLGG--FP--IEGVDVLSLPPRELPHI-R-G--EWFFFAVKPTPTP-G

A-------GGCWVQYGTEKPY-YGGREAVAFRRRFAYRKG-----AST-WLMKEYRLNRP-----VFVVHKV

YRK-----PPPD--S-------QE-----------

>TraesCS2D02G334400.1

MANAAAGCVFQPTGCELIGHYL-PRLGG--FP--IEGVDVLSLCPRALSHR-R-D--EWFFFAAKPTPTP-D

A-------GGCWEQYGQEKAY-YGGREAVAFRRRFAYRRG-----EST-WRMKEYRLNRP-----VFVVHKV

YRK-----TPPD--S-------DEPQLDE------

>TraesCS2D02G334500.1

MANLTAGYVFQPTGRELIHHYL-PRLGG--FP--IEGVDVLSLPPRELPHV-R-G--EWFFFAAKPTPTP-G

A-------GGCWVQYGTEKAY-YGGREAVAFRR-------------------------RP-----VFVVHKV

YRK-----PPPD--S-------EE-----------

>TraesCS2D02G334600.1

MANAAAGCVFQPTGRELIGHYL-PRLGG--FP--IEGVDVLSLRPRALSHR-R-G--EWFFFAAKPTPMP-D

A-------GGCWEQYGQEKAY-YGGREAVAFRRRFAYRRG-----EST-WRMKEYRLNRP-----VFVVHKV

YRK-----PPPD--S-------DEPQLDE------

>TraesCS2D02G334800.1

MAGLRPGVVFRPSGRQLITLYL-RNLRGRAAP--VAGVDIFAASPDALPHR-K-G--EWYFFAAKPMRAP-G

---------GWWMQYGDAKGYEFR--EVYARHRRFAFRADGG--KVET-WLMKEYALHKP-----DLVVYKV

FTK-----PPPEA-A--------SIDLNN------

>TraesCS2D02G336300.1

FN-LPAGVKFDPTGLELLE-HLEGKVAP--HEDFIPTIEICYTHPENLP-----GKMRLHFFHK--NAYDVG

QRKRRKNSDVRWHKTGKSKEIL--G--VIGWKKILVLIKDG-VTSRTTNWTMHQYHLGV----QDELVVSKV

FNKQPVPVYPPSPRAPS-----EDSGLDERLQGDW

>TraesCS2D02G336400.1

FF-LPAGVKFDPTDLELLE-HLEGKVAA--HVDFIPTIEICYTHPENLP-----GKNSCHFFHK--NAYVVG

KRKRRKNSDERWHKTGKSRSIL--G--DIGWKKILVLYKGG-GKTEKTNWRMHQYHLGV----QDELVVSKV

FMNQSLGVYPLSPGS-E-----EDSGLDEHLQEDW

>TraesCS2D02G336500.1

ME-LPIGVKFDPTDLELLG-HLEGKIAV--HVDFIPTIEICYTHPENLP-----GKLISHFFHK--NAYDVG

TRKRRKNSDERWHKTGKSRHIL--G--DIGWKKIMVL-KGG-YKAEKTNWTMHQYHLGV----ENELVVCKV

FEKQCVAVYPPSPEF-E-----DDPDLDEHLQGDW

>TraesCS2D02G354700.1

MAVLPPGHAFEPSGHQIITQYL-PKLRG--TP--VAGVDVFAASPAALPTR-R-G--EWYFYGAQAAPAP-G

---------GCWARYGREKGYVHGAAEAVAFRSRFAFHLGGG---AAT-WLMKEYRLNKP-----DCVVRKI

FTK-----PPPDE-S-D-----ED-SIEG------

>TraesCS2D02G361500.1

LKDLPPTSRFDPTPDAIVGRYLRRQIAGKLYPQVVQATDVFAAHPAVLP--A--ADREWFFAVHRRRELENK

ARPRRA-GPGYVLREGR----------VVGIGYRRAFY--RVRRVSRTEWWMEEYGFGKP---EDELVVYKL

YRR--------------------------------

>TraesCS2D02G378800.1

--TLPPGFRFYPSDQELVCHYLYKKVTN-RASGTLVEVDLHAREPWELPDVAKLTASEWYFFSFRDRKYATG

SRTNRATKTGYWKATGKDREVRSPTRAVVGMRKTLVFYQGRAPNGSKTSWVMHEFRLD-PTCIQEDWVLCRV

FQKQK---SSP---S--AAS--GQGSSPMMF--GW

>TraesCS2D02G382800.1

MNHVPPGFRFHPTDEELVDYYLRKKVQLKRIGDIIKDVDLYKIEPWDLQERCKIGEDDWFFFSHKDKKYPTG

TRTNRATTAGFWKATGRDKPIYV-C--LVGMRKTLVFYKGRAPNGQKLDWIMHEYRLETA---DEGWVVCKV

FKKRVA--PF-DHA-H-QLPPGQPRDLDKFSTPDW

>TraesCS2D02G460000.1

--NLPPGYHFAPSEDELIVHYLRPKLDGPAAPDAHHRLPGKDH---------RFGGR-WYFFTKRTRKYATG

SRPDRTPGKGFWKATGPVREIPRP-GKLVGRARTLVFYTG--PDE-PTHWTMYEYNRTS-NDKIDEWVLCTI

Q--KAGG-EVPDDE-PTVAPNS--------IPCTW

>TraesCS2D02G462900.1

SSRLPPGFRFHPRDHELVLDYLCHKLSG----VDMVDVDLNKCEPWELPDAACVGGREWYFFSRHDRKYATG

QRTNRATHTGYWKATGKDRVIT--AAAVVGMRKTLVFYLGRAPRGTKTEWVMHEFRVE-AL-E-EDWVLCRV

FYKSTTASPVAD-G--A-------PKPE--VPQ-W

>TraesCS2D02G567000.1

ME-LPPGYRFHPTDVELTLYYLKRKLLGKKLLNAVAEVDIYKHAPWDLPKSSM--TGQWYFFCTRGRKYSVG

HRANRSTEGGYWKATGKDRQVVYE--RTVGMKRTLVFHSGKAPKGTRTDWVMYEYRLVQAGVRLDDSVLCKV

HKKSGPGPLSPAAH-PDVVPCD--VELNDLVSNDV

>TraesCS2D02G568000.1

MA-RP-GFRFHPTDQELVGFFLRRKVLGHGG--FIPEVDLYKFEPHHLPAIS-S-STEWYFFAPRGRKYPTG

FRMVRATVKGFWKSTGKDRPVMHN-I-VVGMKKTLVFHMGQAPGGTRTDWVMHEYRLHG-RNNEDTYALCRV

FNKNMAS-FMPGNSSPDANAVE--EGEKQF---DW

>TraesCS2D02G576200.1

MDCVPPGFRFHPTEEELVGYYLARKVAAQTIDDIIQEVDLYRIEPWDLQERC--GQSEWYFFSFKDRKYPSG

TRTNRATAAGFWKATGRDKPVMSSG--VIGMRKTLVFYRGRAPNGRKTDWIIHEYRLQTPT-QEEGWVVCRA

FQKPAPNQTTPDAP-G-NIPPPGDNFLDSL---H-

>TraesCS2D02G576300.1

MECVPPGFRFHPTEEELVGYYLARKVAAQKIDDIIQEVDLYRIEPWDLQERC-GGEQEWYFFSFKDRKYPSG

TRTNRATAAGFWKATGRDKPVMSSG--VIGMRKTLVFYRGRAPNGRKTDWIIHEYRLQTPT-QEEGWVVCRA

FQKPAPNQAAPDTP-G-NIPPPGDNFLDSL---Q-

>TraesCS2D02G576400.1

MDCVPPGFRFHPTEEELVGYYLARKVAAQKIDDIIQEVDLYRIEPWDLQERC-GGEQEWYFFSFKDRKYPSG

TRTNRATAAGFWKATGRDKPVTSSG--VIGMRKTLVFYRGRAPNGRKTDWIIHEYRLQTPT-QEEGWVVCRA

FQKPTPNQAAPEAP-G-NIPPPGDNFLDSL---Q-

>TraesCS2D02G576500.2

M-CVPPGFRFHPTEEELVGYYLARKVAAHKIDDIIQEVDLYRIEPWDLQERC-GGQSEWYFFSFKDRKYPSG

TRTNRATAAGFWKATGRDKPVTSSG--VIGMRKTLVFYRGRAPNGRKTDWIIHEYRLQTPTHQEEGWVVCRA

FQKPTPNQAAPNAP---NIPPPGDNFLDSL---P-

>TraesCS3A02G077900.1

--ELPMGFRFHPTDEEIITSYLAPKILNPAFDTAIGEVDLNKNEPWELPKKAKMGENEWYFYCQKDRKYPTG

IRTNRATKAGYWKTTGKDKEIVNPTSMLIGMKKTLVFYKGRAPSGEKTNWVMHEYRLKIPSS--KEYVVCRI

FHKNA-------------------SRLSSM---DC

>TraesCS3A02G078400.1

MTGLPPGFRFYPTDEELIVHYLRRRAAAAPCPAVIAEVDIYKLDPWELPSRAVFEDNEWYFFSPRDRKYPNG

VRPNRAAGSGYWKATGTDKPITAGGGEVVGVKKALVFYQGRPPKGLKTNWIMHEYRLADASMRLDDWVLCRI

YKKPN---PSP----PDPPPTASN------MNGD-

>TraesCS3A02G107400.1

---LPAGVKFDPTDQELIE-HLEAKVEGRSHPEFIPTIDICYTHPEKLP-----GTMLKHFFHR--KAYTTG

TRKRRKTE-GRWHKTGKTRPVM--G--RQGCKKILVLYKHR--KPEKTNWVMHQYHLGD----EEELVVCKI

FRQ-AAVQ---EAEG-DSRPTSD-AGLEEL--G-W

>TraesCS3A02G113300.1

ME-TPPAFKFDPTDADIVAGYLLPRALG-PYA-AIIEDDPASAPPWEL---RR-GGRDAFFFGPP------G

RRKSRTIGAGVWQGQKGTVTLL-PG----GKRYDLTFRKG------STGYVMHEYEIISP--------LSRV

NKK---KRERP---SAGAFP--NS------APWE-

>TraesCS3A02G157600.1

MAR---GLIFAPGDADLITIHLQRKSSGSSLHRYIHNADVYAAEPAALP-SAR-G--RWYFFTSV-RQSSRD

TRRCRAVAGGTWHEKA-RCVVLDGGGAAVGYRQSFTYEK---NG-----WLMLEFS------QRRMPVLCKI

YRARSA-S------S-S--PSDNA------TSSSW

>TraesCS3A02G176500.1

VWALPPGFGFHPKDTELVAHYLKKKILGQKIEDIIPEVDIYKHEPWDLPAKC-V--QKWHFFAARDRKYPNG

ARSNRATVAGYWKSTGKDRAI---KKRTIGTKKTLVFHEGRPPTGKRTEWIMHEYYIDEPDLKFDNTAVDSI

--SAQLSPMAPDFS-PNQLPGGSEKFVDNNITSNL

>TraesCS3A02G247500.1

MV-------FHPCEQELIAAYLGPRVTDGDMSKFIHEGDVYAAHPEDLA-VS--G--EWYFFTAV-RKG--G

GRRGRTVDSQCWHEAG-SKPVVSAHGGLLGHRQNFSFVK---EDGVRSGWLMVELGLHG-DGQ--EVTLCKV

YRAKNN-KDSP--RS----PPS---TYEEL-----

>TraesCS3A02G269900.1

LASAAQRPDFHPSDQVLIKSYLTPRVASGQHPQFTHDVDVYTASPGALP-MA--G--KWYFFTLL-PKSAHG

QRRPRTVGTGCWHEAG-VKPVLDG-DHPIGWRQFFSFMK---EEGQRSGWIMVEIGLDH--GKPSELVLCKV

YRAP-V-E-GDTLPSR-CGMSRSE-DEDDPMTG--

>TraesCS3A02G336300.1

MDYLVPGVRFVPTDQELILCYLRSKLRGDPPPSLVRDDDVYAEHPEYLP--S-V-ED-WYVFTQRSRKYAKG

GRPSRSGDTGRWKSVGKNTPVTYG-KATIGFRNSLAYEDGDSKKKRKTEWKMTEFDVDSPNFMLNDWVLCRI

TRKKKKGETAPDSASPHRTPPS--SSSSGG---D-

>TraesCS3A02G339600.1

MMNLPPGFRFHPTDDELVVHYLCRKVAGQPQPPIIAEVDLYKFNPWDLPERALFGSREWYFFTPRDRKYPNG

SRPNRSAGTGYWKATGADKPVAPRGGRTVGIKKALVFYSGRAPRGVKTDWIMHEYRIAEASLKLDEWVLCRL

YNKKNNWEAGPGEMASD---QQEDS-MDDLMNMNS

>TraesCS3A02G377700.2

---FPAGVKFDPTDQELIE-HLESKV--RAHSDFIPTIEICYTHPEKLP-----GTRQKHFFHR--KAYTTG

TRKRRKAEGARWHKTGKTRPLM--G--QHGCKKILVLYKTR--KAEKTNWVMHQYHLGD----EDELIVSKV

FRQTAATS-EVAAQK-------D-AGLEGL--G--

>TraesCS3A02G387500.1

MA--APGFSFCPTDSELVSFYLRPRISGQPLPQFFHEADVYATDPASLP-PAR-G--KWYFFSLV-KRSAQD

VRKCRIVGGGTWKERG-N-DVVGAEGHAVGRLEKFTYTS---PKEDPPEWLMMEFS--V-G-QGEVLCLCKI

YRFKFA-S------PP---PNPNW---EEL----C

>TraesCS3A02G403300.1

SSDVMPGFRFHPTEEELIEFYLRRKVEGKRFNELITFLDLYRYDPWELPALAAIGEKEWFFYVPRDRKYRNG

DRPNRVTASGYWKATGADRMIR--NNRSIGLKKTLVFYSGKAPKGVRSSWIMNEYRL--PRYHTE--SLCRV

YKRT----AEAGSSPPQVKP--N-DELSTLLPADW

>TraesCS3A02G406000.1

MSNLPPGFRFHPTDEELVTHYLCRRCAGAPIAPIITEIDLYKFDPWQLPKMALYGEKEWYFFSPRDRKYPNG

SRPNRAAGSGYWKATGADKPVGT--PKPLAIKKALVFYAGKAPKGEKTNWIMHEYRLADSSLRLDDWVLCRI

YNKKGGME--PPAMGPDSMPADEF--ISEWVNPDL

>TraesCS3A02G438900.1

MAGLPVGFRFRPTDEELLLHYLRRKALSCPLPDIIPVADLARLHPSDLP-----GEAERYFF-----HLPTG

GGAGRAGGSGVWRASGKERLVVAP--RPIGAKRTLVFC----PGGARTGWAMHEYRLL-PSLHKD-WVVCRV

FKKAT-----P----A-MTP----E--SG--AS--

>TraesCS3A02G471300.1

MA-LTPGYKFMPTDEEVIAFYLIPRLRGQPLPVIIDR----SAPPWKL-----FGEVEHAFFSASG-EYASA

KRKVRAY-GGTWVKVGNKGKLR-------GQVYRMNYQLGVGRRTGSTGWVMLEYRVPDPAGGEATWT----

YQETTTTA-FPEAPTPK-MP--DPSFSD-F---N-

>TraesCS3A02G485400.1

ME-MKEEVSFEPTEDELVLHFLRPRLRG-RVA-AVVEADPCAAPPWEL---ERL-LGGGYFFAARRR---K-

--VRRTPGGGAWMHSGNRRSVT--L----GCMTRYCFYRGGAQQGRSTGWVMSEYEI---------------

-RCRRA-----DQ-I--------------------

>TraesCS3A02G485500.1

EE-VKEGLSFEPTEDELVLHFLRPQLRG-RVA-AVVEADPCAATPWEL---ARL-LRGGYFFAARRR---K-

AQARRTPGGGAWMHSGNRRSVT--L----GSMTRYCFYR-WA-QGRSTGWVMSEYEI---------------

-RCRRA-----DH-V--------------------

>TraesCS3A02G486500.1

ME-VKEEESFEPTEDELVLHFLRPQLRG-RVA-AVVEADPCAAPPWDL---ERL-LRGGYFFHARRR---KP

VQVRRTPGGGTWMHSGNRRSVT--L----GSMTRYCFYR-SA-QGRSTGWVMSEYEI---------------

-RCRRA-----EE-T--------------------

>TraesCS3B02G092800.1

--ELPPGFRFHPTDEEIITSYLAPKILNPAFNTAIGEVDLNKNEPWELPNKAKMGENEWYFYCQKDRKYPTG

IRTNRATKAGYWKATGKDKEIVNPMSMLMGMKKTLVFYKGRAPSGEKTNWVMHEYRLEIPSS--KEYVVCRI

FHKNT-------------------SGVSSMPGNGC

>TraesCS3B02G092900.3

--KLPSGFRFHPTDVEIITAYLVPKVLKKPFDREVGEVDLNKHDPWELPEMANMGEKEWYFFSQKDHKYPTG

IRTNRATTAGYWKATGKDKEIFHPTMSLIGTKKTLVFYKGRAHKGEKTNWIMHEYRLERPSSK-EEYVVCRI

FHKSI-GLTLPDYNS-SFLPHQDQSEPSSILSKDW

>TraesCS3B02G093300.1

MTGLPPGFRFYPTDEELIVHYLRRRAAAAPCPAVIAEVDIYKLDPWELPSRAVFEDNEWYFFSPRDRKYPNG

VRPNRAAGSGYWKATGTDKPITAGGGEVVGVKKALVFYQGRPPKGLKTNWIMHEYRLADVSMRLDDWVLCRI

YKKPN---PSP----PDPPPTASN------MNGD-

>TraesCS3B02G184300.1

MAR---GLIFAPGDANLITIHLQRKISGSSLPRYIHNADVYAAEPAALS-SAR-G--RWYFFTSV-RQSSRD

TRRCRAVAGGTWHEKA-RCVVLDGDGGIVGYRQSFTYEK---NG-----WLMLEFS------QRRMPVLCKI

YRARSA-S------S-S-MPSDNA------TSS--

>TraesCS3B02G184500.1

MAR---GLIFAPGDANLITIHLQRKISGSSLPRYIHNADVYAAEPAALS-SAR-G--RWYFFTSV-RQSSRD

TRRCRAVAGGTWHEKA-RCVVLDGDGGIVGYRQSFTYEK---NG-----WLMLEFS------QRRMPVLCKI

YRARSA-S------S-S-MPSDNA------TSSRW

>TraesCS3B02G208300.2

--ALPPGFGFHPKDTELVAHYLKKKILGQKIEDIIPEVDIYKHEPWDLPAKC-V--QKWHFFAARDRKYPNG

ARSNRATVAGYWKSTGKDRAI---KKRTIGTKKTLVFHEGRPPTGKRTEWIMHEYYIDEPDLKFDNTAVDSV

--SAQLSPMAPDFS-PNQLPGGSEKFVDNNITCNL

>TraesCS3B02G271900.1

MV-------FHPCEQELIAAYLGPRVTEGDMSKFIHEGDVYAAHPEDLA-VA--G--EWYFFTAV-RKG--G

GRRGRTVDSQCWHEAG-SKPEVSAHGGLLGHRQNFSFVK---EDGVRSGWLMVELGLHG-DGQ--EVTLCKV

YRAKNN-KGSP--RS----PPS---TYEEL-----

>TraesCS3B02G303800.1

LTSAAQRPDFHPSDQVLIKSYLTPRVASGQHPQFTHDADVYTASPGALP-MA--G--KWYFFTLL-PKSAHG

QRRPRTVGTGCWHEAG-VKPVLDG-GHPIGWRQFFSFMK---EEGQRSGWIMVEIGLDH--GQPSELVLCKV

YRAP-V-E-GDTLPSR-CGMSHSE-DEDDPMTG--

>TraesCS3B02G367400.1

LEYLVPGVRFVPTDQELILCYLRSKLRGDPPPTLVHDDDVYAEHPQILP--S-V-ED-WYVFTQRNRKYAKG

GRPSRNGDTGRWKSVGKNMPVTYG-KATIGFRNSLAYEDGDSKKKRKTEWKMAEFDVDSPNFMLNEWVLCRI

TRKKKKGETASDSASPHRTPPS--SSTDDG---D-

>TraesCS3B02G371200.1

MMNLPPGFRFHPTDDELVVHYLCRKVAGQPQPPIIAEVDLYKFNPWDLPERALFGSREWYFFTPRDRKYPNG

SRPNRSAGTGYWKATGADKPVAPKGGRTVGIKKALVFYSGRAPRGVKTDWIMHEYRIAEASLKLDEWVLCRL

YNKKNNWEAGPGEMASD---QGEDS-MDDLMNMDS

>TraesCS3B02G407600.1

MA-LPPGYRFYPTEEELICFYLRNKLDGR--GRVIPVVDVYSVDPLQLSEIH--EGGEWFYFCARQEREARG

GRPSRTTPSGYWKAAGTPGVVYSA-RRPIGLRKTMVFYRGRAPSGTKTKWKMNEYRAFQPQLR--EFSLCRL

YTKSGTLR--P---A--PGPAT--GSMQPL---DL

>TraesCS3B02G410500.1

---FPAGVKFDPTDQELIE-HLESKV--RAHSDFIPTIEICYTHPEKLP-----GTRQKHFFHR--KAYTTG

TRKRRKAEGARWHKTGKTRPLM--G--RHGCKKILVLYKAR--KPEKTNWVMHQYHLGD----EDELIVSKV

FRQSAATS-EVAAQN-------D-AGLEGP--G--

>TraesCS3B02G421300.1

MA--APGFAFCPTDSELVSFYLRPRISGQPLPQFFHEADVYATDPASLP-PAR-G--KWYFFSLV-KKSAQN

SRKSRTVGGGTWKERG-N-DVVDAEGHAVGRFEKFTYTN---PKEDPPEWLMMEFS--V-G-QGQVLCLCKI

YRFKSA-S------PP---PNPNW---EEL----C

>TraesCS3B02G436900.1

SSDVMPGFRFHPTEEELIEFYLRRKVEGKRFNELITFLDLYRYDPWELPALAAIGEKEWFFYVPRDRKYRNG

DRPNRVTASGYWKATGADRMIR--SSRPIGLKKTLVFYSGKAPKGVRSSWIMNEYRL--PRYHTE--SLCRV

YKRT----AAAGSS-PQVKP--N-DELSTLLPTDW

>TraesCS3B02G439600.1

MSNLPPGFRFHPTDEELVTHYLCRRCAGAPIAPIITEIDLYKFDPWQLPKMALYGEKEWYFFSPRDRKYPNG

SRPNRAAGSGYWKATGADKPVGT--PKPLAIKKALVFYAGKAPKGEKTNWIMHEYRLADSSLRLDDWVLCRI

YNKKGGLE--PPAMPQDSMPADDF--ISEWVNPDL

>TraesCS3B02G446100.1

MA--APGFAFCPTDRELVSFYLRPRISGQPLPHYFHEADVYATDPASLP-PAK-G--KWYFFSLV-KRSAQD

SRKCRIVGGGTWKERG-N-DVAGAEGRAIGRLERFTYTS---PKEDPPEWLMTEFS--V-D-QGQVLCLCKI

YRFKSA-S------PP---PN-NF---EEF----C

>TraesCS3B02G472800.1

MAGLPIGFRFRPTDEELLLHYLRRKALSCPLPDIIPVADLARLHPWDLP-----GEAERYFF-----HLPTG

GGTGRAGGSGVWRASGKERLVVAP--RPIGAKRTLVFC----PGGARTGWAMHEYRLL-PSLHKD-WVVCRV

FKKAT-----P----A-MTP----ESRSG--AS--

>TraesCS3B02G533100.1

EE-VKQEVEFEPTEDELVLHFLRPQLRG-RVA-AVVEADPCASPPWEL---ARL-LRGGYFFAARRR---K-

--VRRTPGGGAWMHSGNRRSVT--L----GTMTRYCFYRGGAQQGRSTGWVMSEYEI---------------

-RCRRA-----DQ-I--------------------

>TraesCS3B02G533200.1

---AGEEEEFEPTEDELVLHFLRPQLRG-RVA-AVVEADPCAATPWEL---ARL-LRGGYFFAARRR---K-

AQARRTPGGGAWMHSSNRRSVT--L----GSMTRYCFYR-WA-QGRSTGWVMSEYEI---------------

-RCRRA-----DH-V--------------------

>TraesCS3B02G534500.1

EE-VKQEVEFEPTEDELVLHFXXXXXXX-XXX-XXXXXXXXXXXPWEL---ARL-LRGGYFFAARRR---K-

AQARRTPGGGTWMHSGNRCSMT--L----GSMTRYCLYR-GA-QGRSTGWVMSEYEI---------------

-RCRRA-----DQ-I--------------------

>TraesCS3B02G599700.1

MN--PPGFRFTPTREELIRYYL-GKVQG------VA--DIYGEDPAAL-SR-RFG--NWYFLCV--RKGNRG

GRTSRTVGGGTWHGYGKRVAVP---EA--G-RQAFEYHRSRASEPLK-DWAMKD---EPPTNNQEPWPLEML

TEKTNNALNLPDQS-PEPLPGA--AVVEEF----W

>TraesCS3B02G599900.1

MD--PPGFRFTPTQEELILYYL-GKDLG------VA--DVYGEDPAAL-SR-RFG--NWYFLCV--RKGNRG

GRASRTVGGGTWHGSGKRVAVA---GA--G-------HRSRAPEALK-DWAMEDWELEPPTNNQEPW-----

-EKTNNASNLPDQS-PEPLPGA--ATVQEL----W

>TraesCS3B02G600000.1

MN--PPGFRFTPTREELIGYYL-GKVRG------VA--DIYGEDPAAL-SR-RFG--NWYFLCV--RKG-AG

GRASRAVGGGTWHGYGKRVAVA---EA--G-RQAFEY-RSRAPEPLK-DWAMEDWELEQPTNNQEPWPLEML

TEKTNNVSNLPDQS-QEPLPGA--AAVEEF----W

>TraesCS3D02G078500.1

--ELPPGYRFHPTDEEIITSYLAPKILNPAFDTAIGEVDVNKNEPWELPKKAKMGENEWYFYCQKDRKYPTG

IRTNQATKAGYWKTTGKDKEIVNPTSMLIGMKKTLVFYKGRAPSGEKTNWVMHEYRLEIPSS--KEYVVCRI

FHKNT-------------------SGLSSMPGNDC

>TraesCS3D02G078900.1

MTGLPPGFRFYPTDEELIVHYLRRRAAAAPCPAVIAEVDIYKLDPWELPSRAVFEDNEWYFFSPRDRKYPNG

VRPNRAAGSGYWKATGTDKPITAGGGEVVGVKKALVFYQGRPPKGLKTNWIMHEYRLADASMRLDDWVLCRI

YKKPN---PSP----PDPPPTGSN------MNGD-

>TraesCS3D02G109400.2

---LPAGVKFDPTDQELIE-HLEAKVEGRSHPEFIPTIDICYTHPEKLP-----GTMLKHFFHR--KAYTTG

TRKRRKTE-GRWHKTGKTRPVM--G--RQGCKKILVLYKHR--KPEKTNWVMHQYHLGD----EEELVVCKI

FRQ-AAVQ---AAEG-DSRPTSD-AGLEEL--G-W

>TraesCS3D02G165200.1

MAR---GLVFAPGDADLITIHLQRKISGSSLPRYIHNADVYGAEPAALS-SAR-G--RWYFFTSV-RQSSRD

TRRCRAVAGGTWHEKA-RCVVLDGGGAVVGYRQSFTYEK---NG-----WLMLEFS------QRRMPILCKI

YRARSA-S------S-S--PSDNA------TSSSW

>TraesCS3D02G170000.1

MEELPPGFRFQPTDQEIIVCYLKKVASAASAVSIIADVDIYKFDPWELPDKAQFGEGEWFFFSPRDRKYPNG

ARPNRTAGSGYWKATGTDKPILAAGARCLGVKKALVFYQGRSPRGTKTEWVMHEYRLLHASMRLDDWVLCRV

RKKGVAVA-------P----VDEV--------SD-

>TraesCS3D02G183900.2

VWALPPGFGFHPKDTELVSHYLKKKILGQKIEDIIPEVDIYKHEPWDLPAKC-V--QKWHFFAARDRKYPNG

ARSNRATVAGYWKSTGKDRAI---KKRTIGTKKTLVFHEGRPPTGKRTEWIMHEYYIDEPDLKFDNTAVDSV

--SAQLSPMAPDFS-PNQLPGGSEKFVDNNIASNL

>TraesCS3D02G243900.1

MV-------FHPCEQELIAAYLGPRVTDGDMSKFIHEGDVYAAHPEDLA-VA--G--EWYFFTAV-RKG--G

GRRGRTVDSQCWHEAG-SKPVVSAQGGLLGHRQNFSFVK---EDGVRSGWLMVELGLHG-DGQ--EVTLCKV

YRAKNN-KGSP--RS----PPS---TYEEL-----

>TraesCS3D02G245500.1

NNQLPAGVKFDPTDQELLE-HLEGKADTKLHPEFIPAIEICYTHPERLP-----GGKLRHFFHR--KAYTTG

TRKRRKTDGGRWHKTGKTRPVF--G--KLGYKKILVLYKQR--KPEKTNWVMHQYHLGS----EEELVVSKV

FRQ-GGAA---GAS------AASP--AAAL--GAM

>TraesCS3D02G269200.1

MA--------HPSEPELIKSYLLGR--TKDLSEFIHDADVYTADPASLP-SD--D--KWYFFSPV-RKNVRG

QRKARTLESGCWHEAG-TKAVEDGDGHRVGCRQFFSFVK---HNGQRTGWLMVELRLDR-EQVPSDLVLCKI

YRVS----------A-STTPAPSC-AEDEPFPAR-

>TraesCS3D02G269600.1

LASVAQRPDFHPSDQVLIKSYLTPRVASGQHPQFTHDADVYTASPAALP-LA--G--KWYFFTLL-PKSAHG

QRRPRTVGTGCWHEAG-VKPVLDG-DHPIGWRQFFSFMK---EEGQRSGWIMVEIGLDH--GKPSELVLCKV

YRAP-V-E-GDTLPSRRCGMSHSE-DEDDPMTG--

>TraesCS3D02G329300.1

MEYLVPGVRFVPTDQELILFYLRSKLRGDPPPSLVRDDDVYAEHPQILP--S-V-ED-WYVFTQRSRKYAKG

GRPSRSGDTGRWKSVGKNTPVTYG-KATIGFRNSLAYEDGDSKKKRKTEWKMMEFDVDSPNFMLNDWVLCRI

TRKKKKGETAPDSASPHRTPPS--SSADDG---D-

>TraesCS3D02G333100.1

MMNLPPGFRFHPTDDELVVHYLCRKVAGQPQPPIIAEVDLYKFNPWDLPERALFGSREWYFFTPRDRKYPNG

SRPNRSAGTGYWKATGADKPVAPKGGRTVGIKKALVFYSGRAPRGVKTDWIMHEYRIAEASLKLDEWVLCRL

YNKKNNWEAGPGEMASD---QGEDS-MDDLMNMNS

>TraesCS3D02G367900.1

MA-LPPGYRFYPTEEELICFYLRNKLDGR--GRVIPVVDVYSVDPLQLSEIH--EGGEWFYFCARQEREARG

GRPSRTTPSGYWKAAGTPGVVYSA-RRPIGLRKTMVFYRGRAPSGTKTKWKMNEYRAFQPQLR--EFSLCRL

YTKSGTLR--P---A--PGPAT--GSMHPL---DL

>TraesCS3D02G370800.1

---FPAGVKFDPTDQELIE-HLESKM--RAHSDFIPTIEICYTHPEKLP-----GTRQKHFFHR--KAYTTG

TRKRRKAEGARWHKTGKTRPLM--G--RHGCKKILVLYKAR--KPEKTNWVMHQYHLGD----EDELIVSKV

FRQSAATS-EVAAQN-------D-AGLEGL--G--

>TraesCS3D02G381900.1

MA--APGFAFCPTDSELVSFYLRPRISGQPLPQFFHEADVYATDPASLP-PAR-G--KWYFFSLV-KKSAQN

SRKSRIVGGGTWKERG-N-DVVDGEGHAVGRFEKFTYTN---PKEDPPEWLMMEFS--V-G-QGQVLCLCKI

YRFKSA-S------PP---PNPNW---EEL----C

>TraesCS3D02G398200.1

SSDVMPGFRFHPTEEELIEFYLRRKVEGKRFNELITFLDLYRYDPWELPALAAIGEKEWFFYVPRDRKYRNG

DRPNRVTASGYWKATGADRMIR--NNRSIGLKKTLVFYSGKAPKGVRSSWIMNEYRL--PRYHTE--SLCRV

YKRT----AEAGSSPPQLKP--N-DELSTLLPTDW

>TraesCS3D02G401200.1

MSNLPPGFRFHPTDEELVTHYLCRRCAGAPIAPIITEIDLYKFDPWQLPKMALYGEKEWYFFSPRDRKYPNG

SRPNRAAGSGYWKATGADKPVGT--PKPLAIKKALVFYAGKAPKGEKTNWIMHEYRLADSSLRLDDWVLCRI

YNKKGGME--PPAMGQDSMPADEF--ISEWVNPDL

>TraesCS3D02G406800.1

MA--APGFAFCPTDSELVSFYLRPRISGQPLPRFFHEADVYATDPASLP-PAR-G--KWYFFSLV-KRSAQD

SRKCRIVGGGTWKERG-N-DVVGAKGRAVGRLERFTYTN---PKEDPPEWLMTEFS--V-D-QGQVLCLCKI

YRFKSA-S------AP---PN-NF---EEF----C

>TraesCS3D02G431700.1

MAGLPIGFRFRPTDEELLLHYLRRKALSCPLPDIIPVADLARLHPWDLP-----GEAERYFF-----HLPTG

GGAGRAGGSGVWRASGKERLVVAP--RPIGAKRTLVFC----PGGARTGWAMHEYRLL-PSLHKD-WVVCRV

FKKAT-----P----A-MTP----ESRSG--AS--

>TraesCS3D02G467000.1

MA-LTPGYKFMPADEEVIEFYLIPRLRGQPLPVIIDR----SAPPWKL-----FGEVEHAFFFTSG-EYASA

KRKVRAC-GGTWVKVGNKGKLR-------GQVYRMNYQWGAGRRTGSTGWVMLEYRVPDPAGGEATWT----

YQE----APVPEAPTPK-MP--DPSFSD-F---N-

>TraesCS3D02G480700.1

ME-VKEEVLFEPTEDELVLHFLRPQLRG-RVA-VVVEADPCAAPPWEL---ARL-LTGGYFFAARRR---K-

--VRRTPGGGAWMHSGNRRSVT--L----GSMTRYCFYRGGAQQGRSTGWVMSEYEI---------------

-RCRRA-----DQ-I--------------------

>TraesCS3D02G480900.1

EE-VKEELSFEPTEDELVLHFLRPQLRG-RVA-AVVEADPCAATPWEL---ARL-LRGGYFFAARRR---K-

AQARRTPGGGAWMHSGNRRSVT--L----GSMTRYCFYR-WA-QGRSTGWVMSEYEI---------------

-RCRRA-----DH-I--------------------

>TraesCS3D02G481900.1

MG-VKQEVEFEPTEDELVLHFLRPQLRG-RVA-AVVEADPCAAPPWEL---ERL-LRGGYFFAARRR---K-

AQARRTPGGGTWMHSGNRRSVT--L----GSMTRYCFYR-GA-QGRSTGWVMSEYEI---------------

-RCRRA-----DH-T--------------------

>TraesCS3D02G535500.1

MD--PPGFRFTPTPEELINFYL-GKVQG------VA--DVYGEDPAAL-SR-RFG--NWYFLCM--RKG-AG

GRASRAVGGGTWHGYGKRVTVA---GA--G-------HRSRAPEPLK-DWAMEDWELEPP----EPWPLEML

TEKTNNALNLPDQS-PEPLPGA--AAVEEF----W

>TraesCS4A02G065000.5

IDSLPRGVEFNPSDSDLLWHLAAEMGNGHPFIEFIKSGDPRDMP----------GRQHLYFFHKK-----NN

NENDKDI---SWQKN-------GPG----G-----TF-NPR------TDWKLHQYYIKNT------LVLSKV

FKSKSHASMYPDK-ASGEGPKAPKDSSEKVAPGDW

>TraesCS4A02G130600.1

MSDLPPGFRFHPTDEEIISHYLTPKALDR-FCGVIGEVDLNKCEPWHLPGKAKMGEKEWYFFCHKDRKYPTG

TRTNRATESGYWKATGKDKEIFRG--LLVGMKKTLVFYLGRAPRGEKTGWVMHEFRLE-PSAK-DEWAVSKV

FNKE---LELPDPPSQHSLP--DMGQTSALLSTAW

>TraesCS4A02G131000.1

M--LPPGFRFHPTDEELILHYLRNRAAESPCPSIIADVDIYKFDPWALPSKASYGDREWYFFTPRDRKYPNG

VRPNRAAGSGYWKATGTDKPIRCSTGESVGVKKALVFYKGRPPKGIKTNWIMHEYRLAAASMRLDDWVLCRI

YKKTSQAS-VP-AYMPE-MPMDED------VTG-L

>TraesCS4A02G213100.1

--SLPPGV-FSPTREESV-ALL-DRIAGKE--------------------------------------CP-G

ARAGRKVDTGYWRAEGG------------GFKSYFVFFPG--PSRKKT-WVAQEF-----SAK-DVPALYML

YRARGIGEKKP----PAAPPDG--ASR-----A--

>TraesCS4A02G219700.1

MDQLPPGFRFHPTDEELVMYYLCRKCGGLPIAPVIAEVDLYKFEPWRLPEKAAGGAKEWYFFSPRDRKYPNG

SRPNRAAGTGYWKATGADKPVGS--PRPVAIKKALVFYAGKPPKGVKTNWIMHEYRLADAALRLDDWVLCRI

YNKKGVIEVKPPARGPESMPTDSPP-GDDWIDGDL

>TraesCS4A02G242700.1

--GLPPGFRFHPTDEELITYYLSRKVSDS-FARAIADADLNKCEPWDLPSKASMGEKEWYFFSMRDRKYPTG

IRTNRATESGYWKTTGKDKEIFHG--RLVGMKKTLVFYGGRAPKGEKTSWVMHEYRIQ--PNK-EEWVVCRV

FKKSQ--ISPCEA-S--FAP--NQAAPGLLCS-HW

>TraesCS4A02G419900.1

MV-VPPGFRFHPTEEELLTYYLARKVASQRIDDVIPNVDLNKLEPWDIQELCRVGGPDWYLFSHKDKKYPTG

TRTNRATAAGFWKATGRDKAIYSVG--RIGMRKTLVFYKARAPHGHKSDWIMHEYRLHHPSTQEDGWVICRV

FKKKNIAVKCSDHK-GHKLPVV--DALDRL-PADW

>TraesCS4A02G420000.1

MV-VPPGFRFHPTEEELLTYYLAKKVASQRIDDVIPDIDLNKLEPWDIQERCRIGGPDWYLFSHKDKKYPTG

TRTNRATAAGFWKATGRDKAIYSAG--RIGMRKTLVFYKGRAPRGHKSDWIMHEYRLDDPSAQEDGWVICRV

FRKKNIVVNCSDKK-AHKLPVV--DALDRL-PADW

>TraesCS4A02G420100.1

MV-VPPGFRFHPTEEELLTYYLAKKVASQRIDDVIPDVDLNKLEPWDIQERCRIGGPDWYLFSHKDKKYPTG

TRTNRATTAGFWKATGRDKAIYSAG--RIGMRKTLVYYKGRAPHGHKSDWIMHEYRLDDPSTQEDGWVICRV

FKKKNIVVNCSANK-AHKLPVV--DALDRL-PADW

>TraesCS4A02G420200.1

MV-VPPGFRFHPTEEELLTYYLAKKVASQRIDDVIPDVDLNKLEPWDIQERCRIGGPDWYLFSHKDKKYPTG

TRTNRATAAGFWKATGRDKGIYSAG--RIGMRKTLVFYKGRAPHGHKSDWIMHEYRLDDPPAQEDGWVICRV

FRKKNIVVNCSDKK-AHKLPVV--DDLDRL-PADW

>TraesCS4A02G420900.1

MV-VPPGFRFHPTEEELLTYYLAKKVASQRIDDVIRDVDLNKLEPWDIQERCRIGGPDWYLFSHKDKKYPMG

TRTNRATAAGFWKATGRDKAIYSAG--RIGMRKTLVFYKGRAPHGRKSDWIMHEYRLEDPSTQEDGWVICRV

FKKKNIVANCSDHK-GHKLPVV--EALDRL-PADW

>TraesCS4A02G421000.1

MV-VPPGFRFHPTEEELLTYYLAKKVASQPIDDVIPDVDLNKLEPWDIQERCRIGGPDWYLFSHKDKKYSTG

TRTNRATAAGFWKATGRDKAIYSAG--RIGMRKTLVFYKGRAPHGHKSDWIMHEYRLDDPSAQEDGWVICRV

FKKKNIVVNCSEKK-AHKLPVI--EALERL-PTDW

>TraesCS4A02G421100.1

ME-VPPGFRFHPTEEELLTYYLAKKAASQRIDDVIRDVDLNKLEPWDIQERCRIGGPDWYLFSHKDKKYPTG

TRTNRATGAGFWKATGRDKAIYSAG--TIGTRKTLVFYKGRAPHGHKSDWIMHEYRLDDP--QEDGWVICRV

FKKKNIVVNCSDHK-AHRLPVV--DALDRL-PADW

>TraesCS4A02G421200.1

MV-VPPGFRFHPTEEELLTYYLAKKVASQRIDDVIPDVDLNKLEPWDIQERCRIGGPDWYLFSHKDKKYPTG

TRTNRATAAGFWKATGRDKAIYSAG--LIGMRKTLVFYKGRAPHGHKSDWIMHEYRLDAPSAQEDGWVICRV

FKKKNIVVNCSDHK-AHKLPVV--YTLDRL-PADW

>TraesCS4A02G421300.1

MV-VPPGFRFHPTEEELLTYYLAKKVASQRIDDVIPDVDLNKLEPWDIQERCRIGGPDWYLFSHKDKKYPTG

TRTNRATAAGFWKATGRDKAIYSAG--RIGMRKTLVFYKGRAPHGHKSDWIMHEYRLDDPSGQEDGWVICRV

FKKKNIVVNCSNHK-AHKLPVVGNDSLDRLVASDW

>TraesCS4A02G499900.1

MTALPVGFRFRPTDEELVRHYLKAKIAGRAHPLAIPDVDLAAVEPWDLPARS-I-SDEWFFFARRDRKYPGS

SRSCRSTAAGYWKATGKDRLIRGPKGALIGVKKTLVFHRGRAPRGARTPWIMHEYTATDPQSQQNSFVLYRL

FNKQ---DLTPDHSSPMWTPIGES-EED--MAPDW

>TraesCS4B02G069400.1

MA-LAPGWTFNPPDRELTDVYLRREIDGQPIASFMHHVDVYSAAPEKLP-EAQ-E--RWYFFTTL-RAGKNP

RRMSRTIAGAWWHEGS-LKAVKGS---AGGALQKLTYKES--ASGS--GWLMTEYSI-------HDLVLCKV

YRRRLALSEAPEEEAP-AAATSNP-LDDTTTTS-C

>TraesCS4B02G072400.1

--GLPPGFRFHPTDEELITYYLSRKVSDS-FVRAIADVDLNKCEPWDLPSKASMGEKEWYFFSMRDRKYPTG

IRTNRATESGYWKTTGKDKEIFHA--RLVGMKKTLVFYGGRAPKGEKTSWVMHEYRIQ--PNK-EEWVVCRV

FKKSQ--ISPCDG-S--FAP--NQAAPGLLCS-HW

>TraesCS4B02G098200.1

MDQLPPGFRFHPTDEELVMYYLCRKCGGLPIAPVIAEVDLYKFEPWRLPEKAAGGSKEWYFFSPRDRKYPNG

SRPNRAAGTGYWKATGADKPVGS--PRPVAIKKALVFYAGKPPKGVKTNWIMHEYRLADAALRLDDWVLCRI

YNKKGVIEVKPPAKGPESMPTDSPP-GDDWIDGDL

>TraesCS4B02G102700.1

MANLPPGV-FSPTREESV-ALL-DRIAGKEVPGFVSHADIYGDSPDALPGSAR-G--TWWFLCE--RQSP-G

ARAGRKVDTGYWRAEGG------------AVKSYFGFF-G--PSRKKT-WLTQEF-----SAT-DVPALYML

YRARGIGEKKP----PAAPPDG--ASSADL-PA--

>TraesCS4B02G132500.1

MTC----MRFNPKGEEAIAVYLVPWLLRQPLPVIIHEAEVYKSEPKDLR-----H---RFFFTTCRRKAGSG

FRMKRTAGAGRWVSDKT--EVKNSADETIGYHEKLRYEKG--QSG-KSEWLMDEYHCRG-FDEHDERVLCRL

YNASVA-LRRPTQQAPPKRP--QS----EFEDVD-

>TraesCS4B02G173600.1

M--LPPGFRFHPTDEELILHYLRNRAAESPCPSIIADVDIYKFDPWALPSKASYGDREWYFFTPRDRKYPNG

VRPNRAAGSGYWKATGTDKPIRCSTGESVGVKKALVFYKGRPPKGIKTNWIMHEYRLAAASMRLDDWVLCRI

YKKTSQVS-VP-DYMPE-MPMDED------VTG-L

>TraesCS4B02G174000.1

MSDLPPGFRFHPTDEEIISHYLTPKALDR-FCGVIGEVDLNKCEPWHLPGKAKMGEKEWYFFCHKDRKYPTG

TRTNRATESGYWKATGKDKEIFRG--ILVGMKKTLVFYLGRAPRGEKTGWVMHEFRLE-PSAK-DEWAVSKV

FNKE---LELPDPPSQHSLP--DMGQTSALLSTAW

>TraesCS4B02G242600.3

IDSLPRGAQFNPSDSDLLWHLAAEMGNGHPFIEFIKYGDPRDMP----------GRQHLYFFHKK-----NN

NENDKDI---SWQKS-------GPG----G-----AF-NPR------TDWELHQYHIKNT------LVLSKI

FKSKCHAPMYPDK-ASGEGPKAPKDSSEKVAPGDW

>TraesCS4B02G328600.1

MADDLPGFRFHPTEEELLGFYLSRVALGKKLHDIIGTLNIYRHDPWDLPGIAKIGEREWYFFVPRDRKAGSG

GRPNRTTERGFWKATGSDRAIRSTPKRVIGLKKTLVFYQGRAPRGTKTDWVMNEYRLP-P---ED--VLCKV

YRKATPLKAC---SHP----LANPSNLCSLLPADW

>TraesCS4B02G328700.1

MADDLPGFRFHPTEEELLGFYLSRVALGKKLHDIIGTLNIYRHDPWDLPGMAKIGEREWYFFVPRDRKAGSG

GRPNRTTERGFWKATGSDRAIRSTPKRVIGLKKTLVFYQGRAPRGTKTDWVMNEYRL--P---ED--VLCKV

YRKATPFKAC--SPHP----VANPPNMSTLLPADW

>TraesCS4B02G328800.1

MADDLPGFRFHPTEEELLGFYLSRVALGKKLHDIIGTLNIYRHDPWDLPGMAKIGEREWYFFVPRDRKAGSG

GRPNRTTERGFWKATGSDRAIRSTPKRVIGLKKTLVFYQGRAPRGTKTDWVMNEYRL--P---ED--VLCKV

YRKATPLKACP-SSHL----VANP--ISSLLPADW

>TraesCS4B02G328900.1

MADDLPGFRFHPTEEELLGFYLSRVALGKKLHDIIGTLNIYRHDPWDLPGMAKIGEREWYFFVPRDRKAGSG

GRPNRTTERGFWKATGSDRAIRSTHKRVIGLKKTLVFYQGRAPRGTKTDWVMNEYRL--P---ED--VLCKV

YRKATPLKAC--HSHP----VANPTNMSSLLPADW

>TraesCS4B02G329100.1

MAEDLPGFRFHPTEEELLGFYLSRVALGKKLHDIIGTLNIYRHDPWDLPGLAKIGEREWYFFLPRDRKAGSG

GRPNRTTERGFWKATGSDRAIRSTPKRVIGLKKTLVFYQGRAPRGTKTDWVMNEYRL--P---ED--VLCKV

YRKATPLKAC--PS-P----VGNPPNMSSLLPADW

>TraesCS4B02G384700.1

MEDVLPGYRFHPTDEELVTFYLRRKVAGKPLSEVIREMDIYKHDPWDLPKGSTVGEKEWYFFCLRGRKYRNS

IRPNRVTGSGFWKATGIDRPIYSASGVLIGLKKSLVYYLGKFGKCTKTDWMMHEFRL--LSMQAEVWTICRI

FRRTITYRIAPQYSTP---PPS--EEIARMVTDD-

>TraesCS4D02G071200.1

--GLPPGFRFHPTDEELITYYLSRKVSDS-FARAIADVDLNKCEPWDLPSKASMGEKEWYFFSMRDRKYPTG

IRTNRATESGYWKTTGKDKEIFHG--RLVGMKKTLVFYGGRAPKGEKTSWVMHEYRIQ--PNK-EEWVVCRV

FKKSQ--ISPCDA-S--FTP--NQAAPGLLCS-HW

>TraesCS4D02G094400.1

MDQLPPGFRFHPTDEELVMYYLCRKCGGLPIAPVIAEVDLYKFEPWRLPEKAAGGAKEWYFFSPRDRKYPNG

SRPNRAAGTGYWKATGADKPVGS--PRPVAIKKALVFYAGKPPKGVKTNWIMHEYRLADAALRLDDWVLCRI

YNKKGVIEVKPPAKGPESMPTDSPP-GDDWIDGDL

>TraesCS4D02G099300.1

--NLPPGV-FSPMREESV-ALL-DRIA-KEVPGFVSRADIYGESPDALPASAR-G--TWWFLCE--RQCP-G

SRAGRKVDTGHWRAEGG------------GVESHFVFF-G--PSRKKT-WLVQEF-----SAN-DVPA---L

YRARGIGEKKP-----AAPPDG--ASAAGVSPA-W

>TraesCS4D02G099900.1

MASLPPGV-FSPTREESV-ALL-DRIA-KEVPGFVCHADIYGESPDALPASAR-G--TWWFLCE--RQCP-G

SRAGRKVDTGHWRAEGG------------GVESHFVFF-G--PSRKKT-WLVQEF-----SAN-DVPALYML

YRARGIGEKKH----PAAPPDG--ASPAGVVPA--

>TraesCS4D02G127300.1

MAC----MRFNPTGEQAIARYLVPWLLDQPLPIIIHEAEVYKSEPKDLR-----H---RFFFTTCIRKAGTG

FRMKRTAGAGKWVSDKT--EVKNSADETIGYHEKLRYAKG--QSG-KSEWLMDEYHCRG-FDQHDERVLCRL

YNASLA-LRRPTQQAPPKRP--QS---DEFEDV--

>TraesCS4D02G175700.1

M--LPPGFRFHPTDEELILHYLRNRAAESPCPSIIADVDIYKFDPWALPSKASYGDREWYFFTPRDRKYPNG

VRPNRAAGSGYWKATGTDKPIRCSTGESVGVKKALVFYKGRPPKGIKTNWIMHEYRLAAASMRLDDWVLCRI

YKKTSQVS-VP-GCMPENMSMDED------VTG-L

>TraesCS4D02G176000.1

MSDLPPGFRFHPTDEEIISHYLTPKALDR-FCGVIGEVDLNKCEPWHLPGKAKMGEKEWYFFCHKDRKYPTG

TRTNRATESGYWKATGKDKEIFRG--VLVGMKKTLVFYLGRAPRGEKTGWVMHEFRLE-PSAK-DEWAVSKV

FNKE---LELPDPPSQHSLP--DMGQTSALLSTAW

>TraesCS4D02G242000.3

IDSLPRGVEFNPSDSDLLWHLAAEMGNGHPFIEFIKSGDPRDMP----------GRQHLYFFHKK-----NN

NENDKDI---SWQKS-------APG----G-----AF-NPR------TDWELHRYYIKNI------LVLSKV

FKSKGHASMYPDK-ASGEGPKAPKDSSEKVAPGDW

>TraesCS4D02G325700.1

MADDLPGFRFHPTEEELLGFYLSRVALGKKLHDIIGTLNIYRHDPWDLPGMAKIGEREWYFFVPRDRKAGSG

GRPNRTTERGFWKATGSDRAIRSTPKRVIGLKKTLVFYQGRAPRGTKTDWVMNEYRL--P---ED--VLCKV

YRKATPLKAC---SCP----IANPPNMCSLLPADW

>TraesCS4D02G325800.1

MADDLPGFRFHPTEEELLGFYLSRVALGKKLHDIIGTLNIYRHDPWDLPGMAKIGEREWYFFVPRDRKAGSG

GRPNRTTERGFWKATGSDRAIRSTPKRVIGLKKTLVFYQGRAPRGTKTDWVMNEYRL--P---ED--VLCKV

YRKATPLKAC--SSHP----VANPSNMSSLLPADW

>TraesCS5A02G099000.1

ME-MAAAFKFDPTDADIVASYLLPRAVG-PHGRAVIDDDPMSLPPWDL---EK-NGTHAFFFGPP------G

GPVKRVVGGGTWQGQNGTVTLFGDA----GRRYDLTY-RGK-----PSGWVMSEYQITSP--------LTRI

GRER------P----PG--P--A-------SPPD-

>TraesCS5A02G127200.1

L--STPGFRFYPTEDELLGFYLRHRLAGTR-PRVIPVVDVYSHHPSHLRSMA--GVQEWFFFCPRAERELRG

GRPARTTPSGYWKATGSPSCVFSSNSKVIGVKRTMVFYQGRAPTGAKTTWKMNEYKAVAPRLR--EFSVCRL

YISTGTLR--P---G--HAPNT--GQADNY---A-

>TraesCS5A02G143100.1

MVSLPPGFRFHPTDEEIITSYLLRKFLDS-FVRAVGEVDLNSCEPRDLPGKANMGEKEWYFFVHKDLKYPTG

SRANRATKEGYWKATGKDREIFKPARELVGMKKTLVFYRGRAPRGAKSEWVMHEFRLE--NPK-DEWVVCKV

FNKK--GEQPPTT-TPNNATSTEQQLPSSASYDG-

>TraesCS5A02G143200.1

--ALPPGFRFHPTDEELIVHYLGRQAASMPSPPIIAEVNIYKCNPWDLPGKALFGENEWYFFSPRDRKYPNG

ARPNRAAGSGYWKATGTDKAILST-NESIGVKKALVFYRGKPPKGVKTDWIMHEYRL----MRLDDWVLCRI

HKKCNNLH---DSN-CDAGADA--KR-KR--SS--

>TraesCS5A02G220900.1

MS-LPPGFRFHPTDVEFVSYYLKRKIMGKKLFEAISEVELYKFAPWDLNKSCL--SKEWFFFCPHDKKNPKG

SWTNRTTPNGYWKTSGKDGTIDLN--RIVGLKKTLIFHEGKAPKGNRTDWVMYEYKMEDTGFSKDAYVL---

---SGLGPFAPEDQ---------------------

>TraesCS5A02G228000.1

MDELLPGFRFHPTDEELVGFYLKRKIQQKPLSELIRQLDIYKYDPWDLPKLASSGEKEWYFYCPRDRKYRNS

ARPNRVTGAGFWKATGTDRPIYS-GTKCIGLKKSLVFYKGRAAKGIKTDWMMHEFRL--P-KVIDAWAICRI

FKKPSSMA-IPQQQSP-EFE--DE---PKILGL-C

>TraesCS5A02G245900.2

--ELPSGFRFHPTDEEIITSYLVPKVLNPTFTIAIAEVDLNKNDPYELPKKAKMGEKEWYFYCQKDRKYPTG

IQTNRATKAGYWKATGKDKEIFHPLPTLIGMKKTLVFYKGRAPRGEKTNWVMHEYRLEISSSK-KERVVCKI

FHKNI-GVTFPDYGS-AVLPHEDQSESSSMLGN--

>TraesCS5A02G271500.2

---WPPGFRFSPTDEELVLFFLKRRVAAGR-PPYIADVDVYKSHPSHLPERSALGDKQWFFCSRLDRKYPNG

SRASRTTADGYWKATGKDRSICN-AGRAVGNKKTLVYHHGRAPRGERTDWVMHEYTILAP-ARRESYALYKL

FEKSGVGP--PDDG-PGFVPISEETA----VPA-W

>TraesCS5A02G275900.1

MESLPPGFRFHPTDEELITYYLRGKIADG-FTRAITEVDLNKCEPWDLPEKAKMGEKEWYFFSLRDRKYPTG

VRTNRATNAGYWKTTGKDKEIFTGAQELVGMKKTLVFYKGRAPRGEKSNWVMHEYRLH--SNK-DEWVVCRI

FAKSP-GVFLPGHDPPHLNPGGGQANGQAMLG-DW

>TraesCS5A02G291200.1

MEQLPPGFRFYPTDVELVLQYLRRMALDRPLPAVIPVHAAAMPDPWDLP-----G-SEAYFFSQRQ------

GGRRRRAAGGYWKATGKEKPVFVQKRLLVGVKTALAFHRGK----SRTDWVMHEYRLA--NKG-QEWVVCRV

SLK-----ET-DH--P--SP----SSTSSCVSS--

>TraesCS5A02G339600.1

MEEIMPGFRFHPTDEELVSFYLKKKIQQKPISELIRQLDIYKFDPWDLPKLASTGETDWYFYCPRDRKYRNS

ARPNRVTAAGFWKATGTDRPIYS-DTRCIGLKKSLVFYRGRAARGIKTDWMMHEFRL--PK-RIDSWTICKI

FKKTSSMA-APQQQSP-QFG-QNQ---GRLLGA-W

>TraesCS5A02G411700.1

MEDVLPGYRFHPTDEELVTFYLRRKVARKSLREVIREMDIYKHDPWDLPKASTVGEKEWYFFCLRGRKYRNS

IRPNRVTGSGFWKATGIDRPIYSASSVSIGLKKSLVYYRGSAGKGTKTDWMMHEFRL--PSMQAEVWTICRI

FRRTITYRAAPQYSTL---PLS--EEIARMVTDDY

>TraesCS5A02G411800.1

MADVLPGYRFHPTDEELVTFYLRRKVARKSLREVIREMDIYKHDPWDLPKASTVGEKEWYFFCLRGRKYRNS

IRPNRVTGSGFWKATGIDRPIYSASSVSIGLKKSLVYYRGSAGKGTKTDWMMHEFRL--PSMQAEVWTICRI

FRRTITYRAAPQYSNP---PLS--EEIARMVTDDY

>TraesCS5A02G411900.1

LEDVLPGYRFHPTDEELVTFYLRRKVARKPLREVIREMDIYKHDPWDLPKASTVGEKEWYFFCLRGRKYRNS

IRPNRVTGSGFWKATGIDRPIYSASGVSIGLKKSLVYYRGSAGKGTKTDWMMHEFRL--PSMQAEVWTICRI

FRRTITYRAAPQYST----PLS--EEIARMVTDDY

>TraesCS5A02G467300.1

MGN-IPGVRFKPTDAE-IIWYLERKYHGHPLPNFIKEFDVFEDHPDTVQEK--YGEG-WYVFSSRDRKYKNG

TRPVRSGTVGFWKSSGKEEDVLK--NVKIGRVNTLTFKLGHQPKGTSTPWRLKEYRMEKPSMLLDPWVICKL

FRTKNPPA---DEA-P-NGPCY--FSLDDF-----

>TraesCS5A02G468300.1

MPNLPPGFRFHPTDDELVEHYLCRKAAGQRLPPIIAEVDLYRFDPWALPDRALFGTREWYFFTPRDRKYPNG

SRPNRAAGNGYWKATGADKPVAPR-GRTMGIKKALVFYAGKAPKGVKTDWIMHEYRLADASLRLDDWVLCRL

YNKKNEWEMEPNTSSPQTAAQSEAS-YDDILDMDF

>TraesCS5A02G491700.1

MDCVPPGFRFHPTDEELVGYYLRKKVASQKIDDVIRDIDLYRIEPWDLTEHCGIGEEEWYFFSFKDRKYPTG

TRTNRATMAGFWKATGRDKAVHE-R--LIGMRKTLVFYKGRAPNGQKTDWIMHEYRLETP--QEEGWVVCRA

FKKRTAY-SNYNAS---ELPPSEDRALDKFE--DF

>TraesCS5A02G500400.1

MADDLPGFRFHPTEEELLGFYLSRVALGKKLHDIIGTLNIYRHDPWDLPGMAKIGEREWYFFVPRDRKAGSG

GRPNRTTERGFWKATGSDRAVRSTPKRVIGLKKTLVFYQGRAPRGSKTDWVMNEYRL--P---ED--VLCKV

YRKATPLKAC---SHP----LANPSNMCSLLPADW

>TraesCS5A02G500500.1

--DDLPGFRFHPTEEELLGFYLSRVALGKKLHDIIGTLNIYRHDPWDLPGMAKIGEREWYFFVPRDRKAGSG

GRPNRTTERGFWKATGSDRAIRSSPKRVFGLKKTLVFYQGRAPRGTKTDWVMNEYRL--P---ED--VLCKV

YRKATPLKAC---THP----VANPSNMSSLLPADW

>TraesCS5A02G500600.1

MADDLPGFRFHPTEEELLGFYLSRVALGKKLHDIIGTLNIYRHDPWDLPGMAKIGEREWYFFVPRDRKAGSG

GRPNRTTERGFWKATGSDRAIRSTPKRVIGLKKTLVFYQGRAPRGTKMDWVMNEYRL--P---ED--VLCKV

YRKATPLKAC--SSHP----VANPSNMSNLVPADW

>TraesCS5A02G500700.1

MADDLPGFRFHPTEEELLGFYLSRVALGKKLHDIIGTLNIYRHDPWDLPGMAKIGEREWYFFVPRDRKAGSG

GRPNRTTERGFWKATGSDRAIRSTPKRVIGLKKTLVFYQGRAPRGTKMDWVMNEYRL--P---ED--VLCKV

YRKATPLKAC-SSSHP----VANPSNMSSLLPADW

>TraesCS5A02G519300.1

--SLPPGFRFHPTDEELIIYYLKSKINGRQIEEIIPEVDLYKCEPWDLPEKSFLPSKEWYFFSPRDRKYPNG

SRTNRATKAGYWKATGKDRKVNSQ--RAVGMKKTLVYYRGRAPHGSRTDWVMHEYRLDEIGLQ-DAYALCRI

FKKTAPGPRSPDDESDQ-FPDTQESQPDDFPNAPV

>TraesCS5B02G104200.1

ME-MAAAFKFDPTDADIVASYLLPRAVG-PHGRAVIDDDPMSLPPWDL---EK-NGTDAFFFGPP------G

GRVKRVVGGGMWQGQNGTVTLF-DA----GRRYDLTY-RGK-----PSGWVMSEYQITSP--------LTRI

GGER------P----AG--P--A-------SPPD-

>TraesCS5B02G126300.1

L--STPGFRFYPTEDELLGFYLRHRLAGTR-SRVIPVVDVYSHHPSHLRSMA--GVQEWFFFYPRAERELRG

GRPARTTPSGYWKATGSPSGVFSSNSKVIGVKRTMVFYQGRAPTGAKTTWKMNEYKAVAPRLR--EFSVCRL

YISTGTLR--P---G--HVPNT--GQADNY---T-

>TraesCS5B02G141900.1

MVSLPPGFRFHPTDEEIITSYLLRKFLDS-FVRAVGEVDLNSCEPRDLPGKANMGEKEWYFFVHKDLKYPTG

SRANRATKEGYWKATGKDREIFKPARELVGMKKTLVFYTGRAPRGAKSEWVMHEFRLE--NPK-DEWVVCKV

FNKK--GEQLPTT-TPNNVPSTEQQTPSSRSYDG-

>TraesCS5B02G142100.1

--ALPPGFRFHPTDEELIVHYLRRQAASMPSPPIIAEVNIYKCNPWDLPGKALFGENEWYFFSPRDRKYPNG

ARPNRAAGSGYWKATGTDKAILST-NESIGVKKALVFYRGKPPKGVKTDWIMHEYRL----MRLDDWVLCRI

HKKCGNLP---DSN-CD-GADA--KR-KR--SS--

>TraesCS5B02G226800.1

MDELLPGFRFHPTDEELVGFYLKRKIQQKPLSELIRQLDIYKYDPWDLPKLASSGEKEWYFYCPRDRKYRNS

ARPNRVTGAGFWKATGTDRPIYS-GTKCIGLKKSLVFYKGRAAKGIKTDWMMHEFRL--P-KVIDAWAICRI

FKKPSSMA-IPQQQSP-EFE--DE---PKILGV-C

>TraesCS5B02G243300.1

MDKLPPGLRFHPTDEQIITSYL---------------------KPWELPKKAKMGENKWYFYCQKDHEDPSG

IQTNRATKVGYWKATGKDKEILDS-PALIGKKKTLVFYKGRAPTGEETKWVMHEYRLEISSSKGDLCSLCYI

DSSHGLGVMAPDSY--S---KS--HQKDIMVARDW

>TraesCS5B02G275200.1

MESLPPGFRFHPTDEELITYYLRGKIADS-FTRAITEVDLNKCEPWDLPEKAKMGEKEWYFFSLRDRKYPTG

VRTNRATNAGYWKTTGKDKEIFTGAQELVGMKKTLVFYKGRAPRGEKSNWVMHEYRLH--SNK-DEWVVCRI

FAKSP-GVFLPGHDPPHLNPGGG-ANGQAMLG-DW

>TraesCS5B02G290200.1

ME-LPPGFRFHPTDVELLLQYLRRMALDRPLPAVIPVHAAAMPDPWDLP-----G-SEAYFFSQRQ------

GGRRRRATGGYWKATGKEKPVFVQKRLLVGVKTALAFHRGK----SRTDWVMHEYRLA--NKR-QEWVVCRV

SMK-----ET-DH--P--SP----SSTSSCVSS--

>TraesCS5B02G338400.1

MEEIMPGFRFHPTDEELVSFYLKKKIQQKPISELIRQLDIYKFDPWDLPKLASTGETDWYFYCPRDRKYRNS

ARPNRVTAAGFWKATGTDRPIYS-GTRCIGLKKSLVFYRGRAARGIKTDWMMHEFRL--PK-RIDSWTICKI

FKKTSSMA-APQQQSP-QFG-QNQ---GKLLGA-W

>TraesCS5B02G338700.1

MEEIMPGFRFHPTDEELVSFYLKKKIQQKPISELIRQLDIYKFDPWDLPKLASTGETDWYFYCPRDRKYRNS

ARPNRVTAAGFWKATGTDRPIYS-GTRCIGLKKSLVFYRGRAARGIKTDWMMHEFRL--PK-RIDSWTICKI

FKKTSLMA-APQQQSP-QFG-QNQ---GRLLGA-W

>TraesCS5B02G356100.1

--GLPPGFRFHPTDEELVNYYLKRKVHGQSIEDIIPEVDLYKCEPWELAEKSFLPSREWYFFGPRDRKYPNG

CRTNRATRAGYWKSTGKDRSINYQ--RSIGMKKTLVFYQGRAPQGIRSNWVMHEYRIEENGVQ-DSYALCRV

FKKNVPAGGNQ----DQ------------------

>TraesCS5B02G415400.1

MEDVLPGYRFHPTDEELVTFYLRRKVAKKSLREVIREMDIYKHDPWDLPKASTVGEKEWYFFCLRGRKYRNS

IRPNRVTGSGFWKATGIDRPIYSASGMSIGLKKSLVYYRGSAGKGTKTDWMMHEFRL--PSMQAEVWTICRI

FRRTITYRAAPQYSTP---PLS--EEIARMVTDDY

>TraesCS5B02G415600.1

MEDVLPGYRFHPTDEELVTFYLRRKVARKSLSEVIREMDIYKHDPWDLPKASTVGEKEWYFFCLRGRKYRNS

IRPNRVTGSGFWKATGIDRPIYSASGVSIGLKKSLVYYRGSTGKGTKTDWMMHEFRL--SSMQAEVWTICRI

FRRAITYRGAPQYSTP---PLS--EEIARMVTDDY

>TraesCS5B02G415700.1

MEDVLPGYRFHPTDEELVTFYLRRKVARKPLREVIREMDIYKHDPWDLPKASTVGEKEWYFFCLRGRKYRNS

IRPNRVTGSGFWKATGIDRPIYSASGVSIGLKKSLVYYRGSAGKGTKTDWMMHEFRL--PSMQAEVWTICRI

FRRTITYRSAPQYSTP---PLS--EEIARMVTDDY

>TraesCS5B02G480900.2

MPNLPPGFRFHPTDDELVEHYLCRKAAGQRLPPIIAEVDLYRFDPWALPDRALFGTREWYFFTPRDRKYPNG

SRPNRAAGNGYWKATGADKPVAPR-GRTMGIKKALVFYAGKAPKGVKTDWIMHEYRLADASLRLDDWVLCRL

YNKKNEWEVEPNTSSPQ-AAQSEAS-YDDILDMDF

>TraesCS5D02G059700.2

MSELPPGFRFHPRDDELICDYLAPKVTG----PPMVDVDLNKVEPWDLPVTASVGGKEWYFYSLKDRKYATG

QRTNRATVSGYWKATGKDRVVARR--ALVGMRKTLVFYQGRAPKGRKTEWVMHEYRLE--SKQ-EDWVLCRV

ICKKKSGVPLPD-TA-NYLPSNDKVVPSMSVSS-W

>TraesCS5D02G111300.1

ME-MAAAFKFDPTDADIVASYLLPRAVG-PHGHAVIDDDPMSIPPWDL---EK-NGTDAFFFGPP------G

GRVTRVVGGGTWQGQNGTVTLF-DA----GRRYDLTY-RGK-----PSGWVMSEYQITSP--------LTRI

GRER------P----AG--P--T-------SPAD-

>TraesCS5D02G134800.1

L--STPGFRFYPTEDELLGFYLRHRLAGTR-PRVIPVVDVYSHHPSHLRSMA--GVQEWFFFCPRAERELRG

GRPARTTPSGYWKATGSPSCVFSSNSKVIGVKRTMVFYQGRAPTGAKTTWKMNEYKAVAPRLR--EFSVCRL

YISTGTLR--P---G--HAPNT--GQADNY---T-

>TraesCS5D02G148800.1

--ALPPGFRFHPTDEELIVHYLGRQAASMPSPPIIAEVNIYKCNPWDLPGKALFGENEWYFFSPRDRKYPNG

ARPNRAAGSGYWKATGTDKAILST-NESIGVKKALVFYRGKPPKGVKTDWIMHEYRL----MRLDDWVLCRI

HKKCGNLP---DSN-CD-GADA--KR-KR--SS--

>TraesCS5D02G148900.1

MVSLPPGFRFHPTDEEIITSYLLRKFLDS-FVRAVGEVDLNSCEPRDLPGKANMGEKEWYFFVHKDLKYPTG

SRANRATKEGYWKATGKDREIFKPARELVGMKKTLVFYTGRAPRGAKSEWVMHEFRLE--NPK-DEWVVCKV

FNKK--GEQLPTT-TPNNAPSTEQQLPSPASYDG-

>TraesCS5D02G239600.1

MDELLPGFRFHPTDEELVGFYLKRKIQQKPLSELIRQLDIYKYDPWDLPKLASSGEKEWYFYCPRDRKYRNS

ARPNRVTGAGFWKATGTDRPIYS-GTKCIGLKKSLVFYKGRAAKGIKTDWMMHEFRL--P-KVIDAWAICRI

FKKPSSMA-IPQQQSP-EFE--DE---PKVLGV-C

>TraesCS5D02G279100.2

---WPPGFRFSPTDEELVLFFLKRRVAAGR-PPYIADVDVYKSHPSHLPERSALGDKQWFFCSRLDRKYPNG

SRASRTTADGYWKATGKDRSICN-AGRAVGNKKTLVYHHGRAPRGERTDWVMHEYTILAP-ARRESYALYKL

FEKSGVGP--PDDG-PGFIPISEETA----VPA-W

>TraesCS5D02G283200.1

MESLPPGFRFHPTDEELITYYLRGKIADG-FTRAITEVDLNKCEPWDLPEKAKMGEKEWYFFSLRDRKYPTG

VRTNRATNAGYWKTTGKDKEIFTGTQELVGMKKTLVFYKGRAPRGEKSNWVMHEYRLH--SNK-DEWVVCRI

FAKSP-GVFLPGHDPPHLNPGGG-ANGQAMLG-DW

>TraesCS5D02G298600.1

ME-LPPGFRFHPTDVELVLQYLRRMALDRPLPAVIPVHAAAMPDPWDLP-----G-SEAYFFSPRQ------

GGRRRKAASGYWKATGKEKPVFVQKQLLVGVKTVLTFHRGK----SRTDWVMHEYRLA--NKR-QEWVVCRV

SLK-----ET-DQ--P--SP----SSTSSCVSS--

>TraesCS5D02G344100.1

MEEIMPGFRFHPTDEELVSFYLKKKIQQKPISELIRQLDIYKFDPWDLPKLASTGETDWYFYCPRDRKYRNS

ARPNRVTAAGFWKATGTDRPIYS-GTRCIGLKKSLVFYRGRAARGIKTDWMMHEFRL--PK-RIDSWTICKI

FKKTSSMA-APQQQSP-QFG-QNQ---GRLLGA-W

>TraesCS5D02G360800.1

--GLPPGFRFHPTDEELVNYYLKRKVHGQSIEDIIPEVDLYKCEPWELAEKSFLPSREWYFFGPRDRKYPNG

CRTNRATRAGYWKSTGKDRSINYQ--RSIGMKKTLVFYQGRAPQGIRSNWVMHEYRIEENGVQ-DSYALCRV

FKKNVPAGANQ----DQ------------------

>TraesCS5D02G420800.1

MEDVLPGYRFHPTDEELVTFYLRRKVAKKSLREVIREMDIYKHDPWDLPKASTVGEKEWY-FCLRGRKYRNS

IRPNRVTGSGFWKATGIDRPIYSASGVSIGLKKSLVYYRGSAGKGTKTDWMMHEFRL--PSMQAEVWTICRI

FRRTITYRAAPQYSTP---PLS--EEIARMVTDDY

>TraesCS5D02G421000.1

MEDVLPGYRFHPTDEELVAFYLRRKMARKSLREVIREMDIYKHDPWDLPEASTVGEKEWYFFCLRGRKYRNS

IRPNRVTGSGFWKATGIDRPIYSASGVSIGLKKSLVYYRGSAGKGTKTDWMMHEFRL--PSMQAEVWTICRI

FRRAITYRAAPQYSNP---PLS--GEIARMVADDY

>TraesCS5D02G421100.1

LEDVLPGYRFHPTDEELVTFYLRRKVARKSLREVIREMDIYKHDPWDLPKASTVGEKEWYFFCLRGRKYRNS

IRPNRVTGSGFWKATGIDRPIYSASGVSIGLKKSLVYYRGSAGKGTKTDWMMHEFRL--PSMQAEVWTICRI

FRRTITYRAAPQYSNP---PLS--EEIARMVTDDY

>TraesCS5D02G472600.1

MEEIMSGFRFHPTDEELVSFYLKKKIQQKSISELIRQLDIYKFDPWDLPKLASTGETDWYFYCPRDRKYRNS

ARPNGVTAAGFWKATGTDRPIYS-GTRCIGLKKSLVFYRGRAARGIKTDWMMHEFRL--PK-RIDSWTICKI

FKKTSSMA-APQQQSP-QFG-QNQ---GRLLGA-W

>TraesCS5D02G481200.1

MPNLPPGFRFHPTDDELVEHYLCRKAAGQRLPPIIAEVDLYRFDPWALPDRALFGTREWYFFTPRDRKYPNG

SRPNRAAGNGYWKATGADKPVAPR-GRTMGIKKALVFYAGKAPKGVKTDWIMHEYRLADASLRLDDWVLCRL

YNKKNEWEVEPHASSPQ-AAQSEAS-YDDILDMDF

>TraesCS5D02G537600.1

MS-MVGGGTWHPE-------------AGKP---------ILGADGESL-KRT-FS------YVKKSRRTGTG

DKSKKKTAAAAEAAPGCDRDIRKPGSTKIAAEKAVSYH-E------KVE--IKEFRLRT-IDQLDRPIISGS

LRKTTINKECPEKK-PTPVPPYDENWMDSDYPTGF

>TraesCS6A02G003800.1

ME-LPPGYRFQPTDAELILYYLKRKILGKKLRNPVTEIDIYQFAPWDLPKSSM--TGQWYFFCTCGRKYPTG

SRTNRSNQAGHWKATGKDRKVVCN--RTVGMKRTLVFHAGKGRKEKRTDWVMHEYRLVEAGVRLDDFVLCKV

YQKSGPGPLSH----PDVVPCD--LELNDL---VV

>TraesCS6A02G051700.1

MA---PGCRFDPHDADLISAYLRPMIAGERLPSFLHSADVYAADPATLPVSPR-D--RWYFFGSA-KRSGRD

RRRSRVVGDGQWHEKG-RKVVSDEQGRIGGYKQEFTYKT---NADGTEVWLMVEFGV---D-QETIPTLCKI

YRKRSS-T------PPSRMPSDNY-ISDQAESQDW

>TraesCS6A02G057400.1

VMNLPPGFRFHPTDEELVADYLCARAAGRAPPPIIAELDLYRFDPWELPERALFGAREWYFFTPRDRKYPNG

SRPSRAAGGGYWKATGADRPVARA-GRTLGIKKALVFYHGRPAAGVKTDWIMHEYRLAG-SLRLDDWVLCRL

YNKKNQWE---EEAAPESLPMGEVS-LDDL--LDL

>TraesCS6A02G065600.1

MA-M--GCRFSPSDADLISFYLRPMIAGEPLPRFLHTADAYGADPAALPLAPK-A--RWYFFCSA-KLSGHD

KRRSRAVGGGTWHEKG-RAAVLDGE--VVGYKQSFRYKI---HSDGEAVWLIVEFRVAH-DRQEKVPVLCKV

YRKRSA-S------SPS---SDNY-NSDQFQSQDW

>TraesCS6A02G065700.1

MA-M--GCRFSPSDADLISFYLRPMIASEPLPRFLHTADAYATDPAALPLAPK-V--RWYLFGPA-KLSGHD

KRRSRAVGGGTWHEKG-RAAVLDGEGRVVGYKQSFRYKI---HSDGEAVWLMVEFRMAH-E-QEKVPVLCKV

YRKRSA-S------SPSLMPSDNY-NSDQFQSQDP

>TraesCS6A02G146100.1

MEDVFHHYRLSPTEVEAVTYYLPRLLSGETLQKLIHRVEISGCEPKDLP-QAV-G---RFFFTTC-KKNGSK

LQSVRGAGAGTWSQKTT--EISH-AGVKVGEVRNLSFKKG--KS---TGWVMEEYQCL--LPESDVKVFCKM

HHADAA-R-QQTHQAPSQAPQSEE---EELANTDW

>TraesCS6A02G208900.3

LA-LPAGVKFDPSDQELLE-HLEEKIGGKPHMEFIPTVEICYSHPENLP-----GNKSAHFFHS--NAYGCG

QRKRRRNSDERWHKTGKSKPVY--G--VMGWKKILVLYTGG--KPDKTDWVMHQYHLGV----ENEFVVSKI

FRPKSEMAKIPSPE--AEVTESDVTSLDESLNGEW

>TraesCS6A02G237900.1

MDHVPPGFRFHPTDEELVDYYLRKKVASKKIDDVIKDVDLYKIEPWDLQDKCKIGEEDWYFFSHKDKKYPTG

TRTNRATSAGFWKATGRDKPIYT-C--LVGMRKTLVFYKGRAPNGQKSNWIMHEYRLETA---EEGWVVCRV

FKKRVA--PC-DHA-QQQLPPKSNRYLDKFTNPSW

>TraesCS6A02G299500.1

MADIPPGFRFRPSDDELIRYYL-PKLQGGHAPAIIE--NVYQCHPDEL-GK-R-GE-SFYFLSPRVRRYDNG

DRPRRDTGRGRWKSTGKK------EKKVAGCMSVLNYFES--PRGGKSEWLMREYKLPKP---LDRYVMCKI

YDKEEAGPKRPNGQMPQVRPPAQQQQQQQYLPTD-

>TraesCS6A02G299600.1

AAHRPPGFRFMPTEDEMIRYYL-PKLQGAYVPAIIE--TVYQCHPDGL-SK-R-GQASFYFLSPRERKYKNG

ARPRRDTRLGRWKSTGKTDE--RGEERVAGCKSGLAYFYF---RGRKSGWLMQEYKLPEP---LDRYVMYKI

YKKDEAGPKRPDGQ-PQVRPPA-QQQQQTYPPAN-

>TraesCS6A02G307200.1

MEEMLPGFRFHPTDEELVRFYLKRKIQQKSLPELIRQLDIYKFDPWDLPKLASTGEKEWYFYCPRDRKYRNS

TRPNRVTGAGFWKATGTDRPIYS-GSKCIGLKKSLVFYKGRAAKGVKTDWMMHEFRL--PQKKLESWAICRI

FKKTNATA------AP-EFA-------RSMLQG-W

>TraesCS6A02G378500.1

--TLPPGFRFYPSDEELVCHYLLSKVAN-RLSGTMVEVDLHVHEPWELPDVAKLTTNEWYFFSFRDRKYTTG

LRTNRATRSGYWKATGKDRVIRSLRDAIVGMRKTLVFYRGRAPNGIKTCWVMHEFRIE-P----ADWVLCRV

FHQKKADTYDP---S-DLHS--DF----SALQVN-

>TraesCS6A02G378600.1

--TLPPGFRFYPSDEELVCHYLHGKVAN-RFAGTMVEVDLHVHEPWELPDVAKLSTNEWYFFSFRDRKYATG

LRTNRATRSGYWKATGKDRVIRSPRAAIVGMRKTLVFYRGRAPNGSKTCWVMHEFRIE-P----EDWVLCRV

FHKKKADTHDP---S-HSLA--DF------LQVN-

>TraesCS6A02G392900.1

ME-MAPAFKFDPTDADLVAHYLLPRALGPPFA-AVIDDDPAGLPPADL---AK-GGGQAFFLHTAD----TP

ESSKRAVGGGRWRGQKASVTLV-PG----GRRSELTYEDGGAGAAAATGWVMHEYQIVSP--------LSRI

-RE-------P---A-------N------------

>TraesCS6B02G033000.1

ME-LPPGYRFQPTDVELILYYLKRKILGKKLRNAVTEIDIYKFAPWDLPKSSM--TGQWYFFCTCGREYPTG

SRTNRSNQAGHWKATGKDRKVVYN--RTVGMKRTLVFHAGKGRKEKRTDWVMHEYRLVEAGVRLDDFVLCKV

YQKSGPGPLSH----PDVVPCD--LELNDLLSSD-

>TraesCS6B02G075200.1

VMNLPPGFRFHPTDEELVADYLCARAAGRAPPPIIAELDLYRFDPWELPERALFGAREWYFFTPRDRKYPNG

SRPNRAAGGGYWKATGADRPVARA-GRTVGIKKALVFYHGRPSGGVKTDWIMHEYRLAG-SLRLDDWVLCRL

YNKKNQWE---DEAATESLP--EVS-LDDL--LDL

>TraesCS6B02G088700.1

MA-M--GCRFSPSDADLISFYLRPMIASEPLPRFLHTADAYAADPSSLPLAPR-A--RWYFFGPA-KLSGHD

KRRSRAVAGGTWHEKG-RVAVLDGEGRVVGYKQSFRYKS---DADGEAVWLMVEFRVAH-D-QEKVPVLCKV

YRKRSA-S------SPSRTPSDNY-------TQDW

>TraesCS6B02G237900.3

MA-LPAGVKFDPSDLELLE-HLEQKIGGKPHMEFIPTVEICYSHPENLP-----GNKSVHFFHS--NAYGCG

QRKRRRNSDERWHKTGKSKPVY--G--VMGWKKILVLYTGG--KPDKTDWVMHQYHLGV----ENEFVVSKI

FRQKSEMAKIPSPE--AEVTESDVTSLDESLNGEW

>TraesCS6B02G286200.1

MDHVPPGFRFHPTDEELVDYYLRKKVASKKIDDVIKDVDLYKIEPWDLQDKCKIGEEDWYFFSHKDKKYPTG

TRTNRATSAGFWKATGRDKPIYT-C--LVGMRKTLVFYKGRAPNGQKSDWIMHEYRLETAILQEEGWVVCRV

FKKRVA--PC-DHA-PQQLPPKSNRLLDKFTNPSW

>TraesCS6B02G323600.1

ME-MPPAFKFDPTDGDIVAYYLLPRALG-PHA-AIIEEDPGSAPPWEL---RR-GAGEAFFFGPP------G

GRRRRTVGGGVWQGQKLTVTML-PG----AKRYDLTFKKGG----SSTGYVMHEYEITSP--------LTRV

NKKKA-GAELP---SAG--P--KS------APFE-

>TraesCS6B02G323700.1

ME-MPPAFKFDPTDGDIVACYLLPRALG-PHA-AIIEEDPGSAPPWEL---RR-GGEEAFFFGPP------G

GRRRRTVGGGVWQGQKLTVTLL-PG----AKRYDLTFKKGG----SSTGYVMHEYEITSP--------LTRV

NKKKA-GAELP---SAG--P--TS------APWE-

>TraesCS6B02G329200.1

MAGIPPGFRFRPSDDELIRYYL-PKLQGGHAPAIIE--NVYQCHPDDL-GK-R-GE-SFYFLSPRVRRYDNG

DRPRRDTGRGRWKSTGKR------EKKVAGCMSVLNYFES--PRGGKSEWLMREYKLPKP---LDRYVMCKI

YDKEEAGPKRPNGQMPQVRPPAQQQQQQQHLPTD-

>TraesCS6B02G335700.1

MEEMLPGFRFHPTDEELVRFYLKRKIQQKSLPELIRQLDIYKFDPWDLPKLASTGEKEWYFYCPRDRKYRNS

TRPNRVTGAGFWKATGTDRPIYS-GSKCIGLKKSLVFYKGRAAKGVKTDWMMHEFRL--PQKKLESWAICRI

FKKTNATA------AP-EFA-------RSMMQG-W

>TraesCS6B02G416300.1

--TLPPGFRFYPSDEELVCHYLHGKVAN-RFAGTMVEVDLHVHEPWELPDVAKLSTNEWYFFSFRDRKYATG

LRTNRATRSGYWKATGKDRVIRSPRAAIVGMRKTLVFYRGRAPNGSKTCWVMHEFRIE-P----EDWVLCRV

FHKKKADTHDP---S-HSLT--DF-----ALQVN-

>TraesCS6B02G416400.1

--TLPPGFRFYPNDEELVCHYLQSKVAN-HFAGTMVEVDLHVHEPWELPDVAKLSANEWYFFSFRDRKYATG

LRTNRATRSGYWKATGKDRVIRSPRATIVGMRKTLVFYRGRAPNGTKTCWVMHEFRIE-P----EDWVLCRV

FHKKKADTHDP---S-HSVS--DF----SALQVN-

>TraesCS6B02G416500.1

M--LPPGFRFYPSDEELVCHYLHGKVAN-CFTGTMVEVDLHVHEPWELPDVAKLSTNEWYFFSFRDRKYATG

LRTNRATRSGYWKATGKDRVIHSPHAAIVGMRKTLVFHRGRAPNGTKTCWVMHELRTL-P----RTGC-CAE

FSTRRKPT--P---S--------------------

>TraesCS6B02G433100.1

ME-TAPAFKFDPTDADLVAHYLLPRAVGPPFA-AIIENDLAGLPPADL---AR-GGGHAFFMHTA-----AP

EIRERGVGGGRWRGQKATVTLV-PG----SRRSELSYEDGGAGAAAATGWVMHEYQIVSP--------LSRI

-RE-------P---A-------N------------

>TraesCS6B02G451300.1

--PMAPGFRFHPTDEELVSYYLRRRVLGRRLRDAIAEVDLYRLEPWDLPPLSRIRSRQWYFFARLDRKVAGA

GRTNRATPRGYWKTTGKDREVFHR-GXXXXXXXXXXXXXXXXXXXXXXXXXXXXXXXXX----QDMHVVCRI

FQKVGSGPNTPDAS-PESYPANSDD--DDIASSHL

>TraesCS6D02G059300.1

VMNLPPGFRFHPTDEELVADYLCARAAGRAPPPIIAELDLYRFDPWELPERALFGAREWYFFTPRDRKYPNG

SRPNRAAGGGYWKATGADRPVARA-GRTVGIKKALVFYHGRPSAGVKTDWIMHEYRLAGRTLRLDDWVLCRL

YNKKNQWE---EEAAPESLP--EVS-LDDL--LDL

>TraesCS6D02G096300.1

MRELPPGFRFHPTDEELVVHYLKKKAAKVPLPTIIAEVDLYKFDPWELPEKATFGEQEWYFFSPRDRKYPNG

ARPNRAATSGYWKATGTDKPILASGREKLGVKKALVFYRGKPPKGLKTNWIMHEYRLTD-SLRLDDWVLCRI

YKKINKAA---DATM-GAGALASSNR-SR-LSSDL

>TraesCS6D02G135400.1

MEDVFHHYRLSPTEVEAVTYYLPRLLSGETLHKLIHRVDITGCEPKDLP-QAM-G---RFFFTTC-KKNGSK

LQSVRSAGGGTWSQKTS--EISH-AGVKVGEVKNLSFKKG--KS---TGWVMEEYRCL--LPESDVKVFSKM

HHADAA-R-QQTHQVPSQAPQLEE---EELANTDW

>TraesCS6D02G140200.1

MC-LGPTENYSWSDEELVRF-LERK-ADDSLPNVVG--DLTLIHPRD-P-----G--NWYLNQSDDQPYGNG

EDIRKA-KGGYWKPTSK----------STGVKFSLEFYEGEAPSGKRTQWLMHEYQVEQ-DANQEYKSLCTI

FKESTNAPNALDGAAP-SPPAMDQSHVEQSSSG-Y

>TraesCS6D02G192400.1

QC-LPAGVKFDPSDLELLE-HLEQKIGGKPHMEFIPTVEICYSHPENLP-----GNKSAHFFHS--NAYGCG

QRKRRRNSDERWHKTGKSKPVY--G--VMGWKKILVLYTGG--KPDKTDWVMHQYHLGV----ENEFVVSKI

FRPKSEMAKIPSPE--AEVTESDVTSLDESLNGEW

>TraesCS6D02G220600.1

MDHVPPGFRFHPTDEELVDYYLRKKVASKKIDDVIKDVDLYKIEPWDLQDKCKIGEEDWYFFSHKDKKYPTG

TRTNRATSAGFWKATGRDKPIYT-C--LVGMRKTLVFYKGRAPNGQKSDWIMHEYRLETA---EEGWVVCRV

FKKRVA--PC-DHA-QQQLPPKSNRYLDKFTNPSW

>TraesCS6D02G279400.1

MADIPPGFRFRPSDDELIRYYL-PKLQGGHAPAIIE--NVYQCHPDEL-GQ-R-GE-SFYFLSPRVRRYDNG

DRPRRDTGRGRWKSTGKK------EKKVAGCMSVLNYFES--PRGGKSEWLMREYKLPKP---LDRYVMCKI

YDKEEAGPKRPNGQMPQVRPPAQQQQQQQYLQTD-

>TraesCS6D02G279500.1

AAHRPPGFRFMPTEDEMIRYYL-PKLQGAYVPAIIE--SVYQCHPDGL-SK-R-GQASFYFLSPRERKYKNG

VRPRRDTRLGRWKSTGKTDE--RGEERVAGCKSGLAYFYF---RGRKSGWLMQEYKLPEP---LDRYVMYKI

YKKDEAGPKRPDGQ-PQMRPPAQQ-QQQPYLPAN-

>TraesCS6D02G286300.1

MEEMLPGFRFHPTDEELVRFYLKRKIQQKSLPELIRQLDIYKFDPWDLPKLASTGEKEWYFYCPRDRKYRNS

TRPNRVTGAGFWKATGTDRPIYS-GSKCIGLKKSLVFYKGRAAKGVKTDWMMHEFRL--PQKKLESWAICRI

FKKTNATA------AP-EFA-------RSILQV-W

>TraesCS6D02G362800.1

--TLPPGFRFYPSDEELVCHYLHGKVAN-RFTGTMIEVDLHVHEPWELPDVAKLSTNEWYFFSFRDRKYATG

LRTNCATRSGYWKATGKDRVIRSPRATIVGMRKTLVFYRGRAPNGSKTCWVMHEFRIE-P----EDWVLCRV

FDQKKADTYDP---S-DSLS--DF----SALQVN-

>TraesCS6D02G362900.1

--TLPPGFRFYPSDEELVCHYLHGKVAN-RLSGTMVEVDLHVHEPWELPDVAKLSTNEWYFFSFRDRKYATG

LRTNRATRSGYWKATGKDRVIRSPRTAIVGMRKTLVFYRGRAPNGSKTCWVMHEFRIE-P----EDWVLCRV

FHKKKADTHDP---S-HSLT--DF-----ALQVN-

>TraesCS6D02G390200.1

--PMAPGFRFHPTDEELVSYYLRRRVLGRRLRDAIAEVDLYRLEPWDLPPLSRIRSRQWYFFARLDRKVAGA

GRTNRATPRGYWKTTGKDREVFHR-GRAVGMKKTLVFHAGRAPKGDRTNWVMHEYRLLD----QDMHVVCRI

FQKVGSGPNTPDTS-PEYYPAYSDD--DDIASSSL

>TraesCS7A02G000300.1

M-ALPVGFRFRPTDEELVRHYLKAKIAGRAHPLAIPDVDLAAVEPWDLPARS-I-SDEWFFFARRDRKYPGS

SRSCRSTAAGYWKATGKDRLIRAPKGALIGVKKTLVFHRGRAPRGARTPWIMHEYTATDP--QQNSFVLYRL

FNKQ---DLTPDHSIPMWTP--ES-EEE--ITPDW

>TraesCS7A02G008800.1

GSQVPPGFRFHPTDEELVDYYLRKKVASRRIDNVIKDVDLYKIEPWDLQEKCRIGEEDWYFFSHKDKKYPTG

TRTNRATAAGFWKATGRDKPIYA-C--LVGMRKTLVYYKGRAPNGQKSDWIMHEYRLETP--QEEGWVVCRV

FKKRLP--PC-DDP-HMTIPPSDDRVLDKFAKSQW

>TraesCS7A02G068000.1

MV-VPPGFRFHPTEEELLTYYLAKKVASQRIDDVIPDVDLNKLEPWDIQERCRIGGPDWYLFSHKDKKYPTG

TRTNRATAAGFWKATGRDKAIYSAG--RIGMRKTLVFYKGRAPHGHKSDWIMHEYRLDDPSAQEDGWVICRV

FKKKNIVVNCSNHK-AHKLPVAGNDSLDRLVASDW

>TraesCS7A02G068200.1

MV-VPPGFRFHPMEEELLTYYLTKKVAPQRMDDVIRDVDLTKLEPWDIQELCRIGGPDWYLFSHKDKKYPTG

TRTNRATAAGFWKATGRDKAIYSAS--RIGMRKTLVFYKGRAPHGHKSDWIMHEYRLDDPSAQEDGWVICRL

FKKK-------------------------------

>TraesCS7A02G068300.1

MV-VPPGFRFHPTEEELLTYYLAKKVASQRIDDVIRDVDLNKLEPWDIQERCRIGGPDWYLFSHKDKKYPTG

TRTNRATAAGFWKATGRDKAIYSAG--RIGMRKTLVFYKGRAPHGHKSDWIMHEYRLDDPSAPEDGWVICRV

FKKKNIVVNCSDRK-GHKLPVV--DDLDRL-PTDW

>TraesCS7A02G106300.1

M-SLPPGYHFAPTDMELIVHYLRRKMDG--HPPIFKDVPITDYRPEQI-EV-MCGER-WYFFTKRTRKYATG

NRPDRTPGRGYWKATGPQRLIRGP-EALVGRRRTLVFYTG--PDEAMTAWTMYEYNLTS-NDKADEWVLCTI

QRQKG-----G-EE-PTMEPSTNSAGTNKLSTYNL

>TraesCS7A02G152500.1

--KFPQGFRFHPTDVEIITSYLVPKVLNKAFDIAVGEVDLNKCEPWELPEKAKMGEKEWYFFSQKDRKYPTG

IRTNRATTAGYWKATGKDKEIFHHTTSLIGMKKTLVFYKGRAPRGEKTNWVMHEYRLESPSSK-EEYVVCRI

FHKST-GLTLPGYGS-SVLLYQDESGPSSILSSDW

>TraesCS7A02G189200.1

--NLPPGFRFQPTDMEIITFYLVPKVLKKVFDTVVKEVDLNKCEPWDLLNKVNMGEKGRYFFSQKGLKYSTG

IRTNRATKAGYWKATGKDKEIIHPTMSIIGMKKTLVFYKGRAPKGEKTNWIMHEYRLKSPSSK-GEYVVCRI

FHKST-GLTLIDYDS-FVLPQQDQYGSSSILSNDW

>TraesCS7A02G194700.1

--NLPAGFRFHPTDMEIITFYLVRKVLKKPFDTVIEEVDLNKCEPWDLQNNVNMGEKDQYFFSKKDLKYPTG

VRTNRATNAGYWKATGKDKEIVHPTMSLIGMKKTLVFYKGRAPRGEKTNWIMHEYRLEMPSSK-EEYVICRI

FHKST-GLTLADYDS-FVLPHQEQSGTSSITSNDW

>TraesCS7A02G204700.1

MED-FQHYRLNPTDVDAVTYYLPRLIAGQ-PHKFIHHVDIYSCEPKDLP-QAA-G---RFFFTTR-KKNGSK

TQSVRTAGGGTWTNATT--AVKH-AGVEVGERKNLSFRKG--KS---TGWVMEEYRCL--LPKADVKVFCKI

HHPDAA-R-EPTHQAPSQAPQSEE---EELASTDL

>TraesCS7A02G208200.1

TG-LPKGVKFDPSDQELLG-HLLAKHAGQPHPEFIPTVKICYTHPQKLP-----GKQSSHFFHR--KAYNTG

TRKRRKTS--RWHKTGKTKPVM--G--QHGCKKIMVLYKGG--KPKKTNWVMHQYHLGT----EDEYVVSKL

F--KSALT--PEYAADN---EN--GDGDE-YKDDI

>TraesCS7A02G209100.1

MEDVFHHYRLSPTDVDAVTYYLPRLLSGETLHKLIHRVNISGCEPKDLP-QAV-G---RFFFTTC-KKNGSK

LQSVRAAGGGTWTQKTT--EIFH-AGVKVGEVKNLSFKKG--KS---TGWVMEEYRCL--LPESDVKVFCRM

HHADAA-R-QETHQAPSQAPQSEE---EELANTDW

>TraesCS7A02G209300.1

MEDVFHHYRLSPTEVEVVTYYLPRLLSGETLHKLIHRVNISGCEPKDLP-QAI-G---RFFFTTC-KKNGSK

LQSVRGAGGSTWTQKTK--EICH-AGVKVGEVKNLSFKKG--KS---TGWVMEEYRCL--LPESDVKVFCKM

HHADAA-R-QPTHQAPSAAP--EE---LEF-ST-W

>TraesCS7A02G209400.1

MEDIFHHYRLSPTEVDAVTYYLPRLLSGETLHKLIHRVEISGCEPKDLP-QAV-G---RFFFTTC-KKNGSK

LQSVRGAGGGTWTQKTT--EICH-AGVKVGEVKNLSFKKG--KS---TGWVMEEYRCL--LPESDVKVFCKM

HHADAA-R-QQTHQAPSAAP--EE---LEFVST-W

>TraesCS7A02G209500.1

MEDVFHHYRLSPTEVDAVTYYLPRLLSGETLHKLIHRVEISGCEPKDLP-QAV-G---RFFFTTC-KKNGSK

LQSVRGAGTGTWTQKTT--EICH-AGVKVGEVKNLSFKKG--KS---TGWVMEEYRCL--LPESDVKVFCRM

HHADAA-R-QQTHQAPSQAPRSEEFDLEELANTDW

>TraesCS7A02G247600.1

PHGLPPGFRFHPTDEELVTFYLAAKVFNACCGVDIAEVDLNRCEPWDLPEAARMGEREWYFFSLRDRKYPTG

LRTNRATGAGYWKATGKDREVLNA-GSLLGMKKTLVFYRGRAPRGEKTKWVLHEYRLD--SCK-EEWVVCRI

FHKAVDPYQSSQ-SSPSPFP----GPPAQP----W

>TraesCS7A02G263100.1

MELLPPGFRFRPTDEELVVQYLRRKALALPLPAVIPVHNLYSLDPWDIP-----G-REKYYFAVRP---AAG

AGGRTTTASGCWKASARERPVVVSRSHLVGVKKSMVFVKGSKPAPAQTGWVMHEYRLALPHHHAGEWVVCRI

F-------QTPDA--P---P-S--SSSSSCVSS--

>TraesCS7A02G299600.3

MTKLPLGFRFHPTDEELVRHYLKGKITGQIKNEVIPEIDVCKCEPWDLPDKA-I-SEEWFFFAPKDRKYPNG

SRSNRATEAGYWKATGKDRVIKSKKQHMIGMKKTLVFHRGRAPKGERTGWIMHEYRTTEPEFEEQGYVLYRL

FQKQLE-KGTPDENSPDLLPMDEGDDAQSTLDADW

>TraesCS7A02G305200.1

MSRMPPGFRFHPRDEELVLDYLLHKLT--AY-VDIVDVDLNKCEPWDLPEAACVGGREWYFFSLRDRKYATG

QRTNRATRSGYWKATGKDRAILAHGEALVGMRKTLVFYQGRAPKGTRTEWVMHEFRLE--QLK-EDWVLCRV

FYKSRTSSPLAD-AA-L-----EKSKLELTLSQ-W

>TraesCS7A02G317100.1

M-CVPPGFRFHPTEEELLNYYLRKKVASEEIDDVIRDVDLNKLEPWDIQEKCKIGGPDWYFFSHKDKKYPTG

TRTNRATAAGFWKATGRDKAIYNAK--RIGMRKTLVFYKGRAPHGQKSDWIMHEYRLDDPAGQEDGWVVCRV

FKKKHHHK---KAR-AQKLPPSEDAMMDRL-ASDW

>TraesCS7A02G326000.1

--CVPPGFRFHPTEEELVGYYLARKVSSHKIDDIIQEVDLYRIEPWDLQERCGGGDPEYYFFSYKDRKYPSG

TRTNRATAAGFWKATGRDKPVLSSSAAVIGMRKTLVFYRGRAPNGRKTDWIIHEYRLQSPT-QEEGWVVCRA

FVKPVPSQSASDNN---DLPPTSSNLWSSL---S-

>TraesCS7A02G334800.1

--DLPPGFRFHPTDEELITYYLLRKVVDG-FSRAIAEIDLNKCEPWELPDKATTTEKEWYFYSLRDRKYPTG

LRTNRATGAGYWKATGKDREIRSANGALVGMKKTLVFYRGRAPKGQKTQWVMHEFRLE-PTTR-DEWVIAKI

FVKP--GAPRQDTSS-TYEP------APRLLQSDW

>TraesCS7A02G349500.1

--SVPPGFRFHPTDEELLYYYLRKKVAYEAIDDVIREIDLNKLEPWDLKDRCRIGGPEWYFFSHKDKKYPTG

TRTNRATTAGFWKATGRDKAIFLGR--RIGMRKTLVFYVGRAPHGKKTDWIMHEYRLDEV--QEDGWVVCRV

FTKKS------DE--AQQLPAEHESILDKLV-AAF

>TraesCS7A02G357500.1

GG-LPAGVKFDPTDQELIE-HLEAKVVAPSHPEFIPTIEICYTHPEKLP-----GSKLRHFFHR--KAYTTG

TRKRRKPPRSRWHKTGKTRPVA--G--RQGCKKILVLYKHR--KPEKTNWVMHQYHLGE----EEELVVSKI

FRQASSTF---RAHGPESTPGSD-SGLEEV--GPW

>TraesCS7A02G375300.1

MAPVPHGYHFVPDDAELLRLLM---IAGRALPSIFHGV-IRNYHPAEL-ELAK-G---IYFYNQ--REFS-G

SRPGRTAKDGWWKASGGGLPLVRR---VVGYKLTLVFYK-R-PGDKKTDWIIKEYIAGP-NRKASEMALYRL

YKKEKATQAAPDAG-PT-LPSD----AQQVVAAD-

>TraesCS7A02G464100.1

MNRLPPGFRFHPRDDELVLDYLAKKL-GSMY-PTMVDVDLNKIEPWDLPEIACIGGKEWYFYSLRDRKYATG

QRTNRATESGYWKATGKDRSISRK--LLVGMRKTLVFYQGRAPKGKKTEWVMHEFRKE--PLK-EDWVLCRV

FYKTRATVSLPD-NN-S----AEQVKREAPFSQ-W

>TraesCS7A02G464800.1

MSRLPPGFRFHPRDDELVLDYLSRKLGGSIY-PAMVDVDLNKIEPWDLPEIACIGGKEWYFYSLRDKKYATG

QRTNRATESGYWKATGKDRAISRK--LLVGMRKTLVFYEGRAPKGKKTEWVMHEFRKE--PLK-EDWVLCRV

FYKTRTTISLPD-NN-S----AEQVKREAPFSQ-W

>TraesCS7A02G549000.1

ME--LPGFRFHPTEEELLEFYLKHHVTRQQLRDIIPTVHLYRHDPWDLPGLAAISEREWYFFVPRDRKHASG

GRPSRTTERGFWKATGSDRAVRCAPKRLVGLKKTLVYYQGRAPRGTKTDWVMNEYRL--P---QD--VLCKV

YRKAVSLKTR---SHP----VS--KKEE--VAVEW

>TraesCS7A02G569100.1

ASVLPPGFRFHPTDEEIIKFYVVPKVLDEAFVAAIEDVNLNKYEPWELPEKAKMGEKEWYFYSRKDRKYPTG

IRTNRATETGYWKATGKDKEIFQP-FKLIGMKKTLVFYKGRAPRGEKTNWIMHEYRLESSASK-EQWVVCRI

FHKST-GLTLPEYDT-SALPHQDQGGLASMLRNDW

>TraesCS7A02G569300.1

ASELPPGFRFHPTDEEIIKFYVVPKVLDEAFVAAIEDVNLNKYEPWELPEKAKMGEKEWYFYCRKDRKYPTG

IRTNQAKNAGYWKATGKDKEIFHP-LTLIGMKKTLVFYKGRAPRGEKTNWIMHEYRLESSSSK-EQWVVCRI

FHKSA-GLTLPGYES-SALPQQDRCGPSSMLSNDW

>TraesCS7B02G004900.1

M-SLPPGYHFAPTDVELIVHYLRRKMDG--HPPVFKDVPITDYRPEQI-EV-MCGER-WYFFTKRTRKYATG

NRPDRTPGRGYWKATGPQRLIRGP-KALVGRRRTLVFYTG--PDEAKTAWTMYEYNLTS-NDKADEWVLCTI

QRQKG-----G-EE-PTMAPSTNSAGTNKLSTYNL

>TraesCS7B02G056300.1

--KFPQGFRFHPTDVEIITSYLVPKVLNKAFDIAIGEVDLNKCEPWELPEKAKMGEKEWYFFSQKDRKYPTG

IRTNRATTSGYWKATGKDKEIFHHTTSLIGMKKTLVFYKGRAPRGEKTNWVMHEYRLECPSSK-EEYVVCRI

FHKST-GLTLPGYGS-SVLPYQDESGPSSIFSSDW

>TraesCS7B02G077700.1

MTTLPPGV-FNPTNEEIVRTYL-NGIAGTS-GAVVIEDDVYGDQPDVLPASTR-S--SWWFRCH--KQATRG

GRGDRGVATGFWKADGE----------HLGFKRTFGFYEH--EGEKKT-WLMEEY-----NVDPDLPALYRI

YRDRKTGRGGP----PAAPPRGDDDDSPELMPA-W

>TraesCS7B02G094000.1

--NLPPGFRFQPTDMEIITFYLVPKVLKKVFNMVVGEVDLNKCEPWDLLKKVSMGDKGRYFFSQKDLKYSTG

IRTNRATKAGYWKATGKDKEIVHPTMSIIGMKKTLVFYKGRAPKGEKTNWIMHEYRLESPSSK-GEYVICRI

FLKSI-GLTLDDYDS-FVLPQQ-------------

>TraesCS7B02G100300.1

--NLPAGFRFHPTDLEIITFYLVRKVLKKPFDIVIEEVDLNKCEPWDLPNNVNMGEKDQYFFSKKDLKYPTG

VRTNWATNAGYWKATGKDKEILHPTMSLIGMKKTLVFYKGRAPRGEKTNWIMHEYRLEMPSSK-EEYVVCRI

FHKST-GLTLADYDS-SVLPHQEQSGTSSITSN--

>TraesCS7B02G111400.1

MED-YQHYRLNPTDVDAVTYYLPRLIACQTLHKFIHHVDIYSCEPKDLP-QAT-G---RFFFTTR-KKNGSK

TQSVRTAGGGTWTNATT--AVKH-AGVEVGERKNLSFRKG--KS---TGWVMEEYKCL--LPEADVKVFCKI

HHPDAA-R-EPMHQAPSQATQSEE---EELANTDW

>TraesCS7B02G111500.1

MED-YQHYRLNPTDVDAVTYYLPRLIAGQTLHKFIHHVDIYSCEPKDLP-QAA-G---RFFFTTR-KKNGSK

TQSVRTAGGGTWTNATT--AVKH-AGVEVGERKNLSFRKG--KS---TGWVMEEYKCL--LPEADVKVFCKI

HHPDAA-R-EPMHQAPSQATQSEE---EELANTEW

>TraesCS7B02G111900.1

MED-YQHYRLNPTDVDAVTYYLPRLIAGQTLHKFIHHVDIYSCEPKDLP-QAA-G---RFFFTTR-KKNGSK

TQSVRTAGGGTWTNATT--AVKH-AGVEVGERKNLSFRKG--KS---TGWVMEEYKCL--LPEADVKVFCKI

HHPDAA-R-EPMHQAPSQATQSEE---EELANTDW

>TraesCS7B02G115400.1

TG-LPKGVKFDPSDQELLG-HLLAKHAGQPHPEFIPTVEICYTHPQKLP-----GKQSSHFFHR--KAYNTG

TRKRRKTS--RWHKTGKTKPVM--G--QHGCKKIMVLYKGG--KPKKTNWVIHQYHLGT----EDEYVVSKL

F--KSALT--PEYAADN---EN--GDGDE-CKDDI

>TraesCS7B02G116200.1

MEDVFHHFRLNPTEVEAVTYYLPRLLSGETLHKLIHHVNISGCEPKDLP-QAV-G---RFFFTTC-KKNGSK

LQSVRGAGGGTWSQKTT--EICH-AGCKVGEVKNLSFKKG--KS---TGWVMEEYRCL--LPEVDVKVFCKM

HHADAA-R-QQTHQAPSQAPQSEE---EELANTDW

>TraesCS7B02G116300.1

MEDIFHHYRLNPTEVEAVTYYLPRLLSGETLHKLIHSVEISGCEPKDLP-QAV-G---RFFFTTC-KKNGSK

LQSVRGAGGGTWSQKTT--EICH-AGCKVGEVKNLSFKQG--KS---TGWVMEEYRCL--LPESDVKVFCKM

HHADAA-R-QQAHQAASAAP--EE---LEFVSTEW

>TraesCS7B02G116400.1

MEDVFHHYRLSPTEVEAVTYYLPRLLSGETLHKLINRVEISGCESKDLP-QAV-G---RFFFTTC-KKNGSK

LQSVRGAGGGTWSQKTT--EIIH-AGCKVGEVKNLSFKKG--KS---TGWVMEEYRCL--LPESDVKVFCKM

HHADAA-R-EATHQAPSQAPQSEE---EELANTDW

>TraesCS7B02G160900.1

MELLPPGFRFRPTDEELVVQYLRRKALALPLPAVISVHNLYSLDPWDIP-----G-REKYYFAVRP---AAG

AGGRTTTASGCWKASARERPVVVSRSHLVGVKKFLAFVKGSKPAPAQIGWVMHEYRLALPHHHAEEWVVCRI

F-------QTPDA--P---PPS--SSSSSCVSS--

>TraesCS7B02G205600.1

MSRMPPGFRFHPRDEELVLDYLLHKLT--AY-VDIVDVDLNKCEPWDLPEAACVGGREWYFFSLRDRKYATG

QRTNRATRSGYWKATGKDRAILAHGEALVGMRKTLVFYQGRAPKGTRTEWVMHEFRLE--QLK-EDWVLCRV

FYKSRTTSPLAD-AA-L-----EKSKLELTLSQ-W

>TraesCS7B02G219400.1

MTEVMPGFRFHPTEEELIEFYLRRKVDGKRFNDLIASVDLYRYDPWDLPALASIGDKEWFFYVPRDRKYRNG

DRPNRVTPSGYWKATGADRMVK-VGNRSIGLKKTLVFYVGKAPKGLRSSWIMNEYRL--PRYQ-E--SLCRV

YKRP----AT-STAS--TPPASNNEELSRANPNDW

>TraesCS7B02G233300.1

--SVPPGFRFHPTDEELLYYYLRKKVAYEAIDDVIREIDLNKLEPWDLKDRCRIGGPEWYFFSHKDKKYPTG

TRTNRATTAGFWKATGRDKAIFLGR--RIGMRKTLVFYVGRAPHGKKTDWIMHEYRLDEV--QEDGWVVCRV

FTKKS------DE--IQQLPAEHESILDKLVAAAF

>TraesCS7B02G246300.1

--ELPPGFRFHPTDEELITYYLLRKVVDG-FSRAIAEIDLNKCEPWELPDKATTAEKEWYFYSLRDRKYPTG

LRTNRATGAGYWKATGKDREIRSANGALVGMKKTLVFYRGRAPKGQKTQWVMHEFRLE-PTTR-DEWVIAKI

FVKP--GAPRQDTSS-TYEP------GSRLLQSDW

>TraesCS7B02G252100.1

MEQVPQGYHFVPSDLELIRLLI---IAGRPLPTIFRNV-IRDYHPAEL-ERAK-G---IYFFSE--REFP-G

GRPRRFTKDGWWKASGGGESLRRR---TVGSKLTLVFYK-K-PGQKKTDWAIKEYKIVD-KKKAEEMALYRL

YNKKHPTQVEP--G-PHLLPFV--QGSQQVVAVD-

>TraesCS7B02G270300.1

GG-LPAGVKFDPTDQELIE-HLEAKVVAPSHPEFIPTIEICYTHPEKLP-----GSKLRHFFHR--KAYTTG

TRKRRKPPRSRWHKTGKTRPVA--G--RQGCKKILVLYKHR--KPEKTNWVMHQYHLGE----EEELVVSKI

FRQ-SSRS---PARGQESTPGSD-SGLEEV--GPW

>TraesCS7B02G364600.1

MNRLPPGFRFHPRDDELVLDYLTRKL-GSIY-PTMVDVDLNKIEPWDLPEIACIGGKEWYFYSLRDRKYATG

QRTNRATESGYWKATGKDRSISRK--LLVGMRKTLVFYQGRAPKGKKTEWVMHEFRKE--PLK-EDWVLCRV

FYKTRATVSLPD-NN-S----AEQVKREAPFSQ-W

>TraesCS7B02G365300.1

MSRLPPGFRFHPRDDELVLDYLSRKL-GSIY-PAMVDVDLNKIEPWDLPEIACIGGKEWYFYSLRDRKYATG

QRTNRATESGYWKATGKDRAISRK--LLVGMRKTLVFYEGRAPKGKKTEWVMHEFRKE--PLK-EDWVLCRV

FYKTRTTISLPD-NN-S----AEQVKREAPFSQ-W

>TraesCS7B02G461700.1

MPQL----Q-DPGDDRLVS--LRRRVAREPFASFIQEADVYLAAPAEL-DHA-MGGKAWYFFSPANHETP-G

ARRKRKVAAGCWHEAGK-VPILGP-KKPVGYKRKLSY---KKPPGSKPNWIMVE-GLEQ-----DQLVLCKL

YRRTGAAPQKPGSSSPNPLPTSDKQSQQQVVHVDY

>TraesCS7B02G472200.1

ME-L-PGFRFHPTEEELLEFYLKHHVTSVR--DIIPTVHLYRHDPWDLPGLAAISEREWYFFVPRDRKHAAG

GRPSRTTERGFWKATGSDRAVRCAPKRLVGLKKTLVYYQGRAPRGTKTDWVMNEYRL--P---QD--VLCKV

YRKAVSLKAR---SHP----VS--KKEE--VAVEW

>TraesCS7B02G472600.1

ME-L-PGFRFHPTEEELLEFYLKHHVTSQHLRDIIPTVHLYRHDPWDLPGLAAISEREWYFLVPRDRKHAAG

GRPSRTTERGFWKATGSDRAVRCAPKRLVGLKKTLVYYQGRAPRGTKTDWVMNEYRL--P---QD--VLCKV

YRKAVSLKAR---SHP----VS--KKEEAVVAVEW

>TraesCS7B02G481400.1

ME-L-------PGEHTLVAV-LRRHAETGELP--VHEVNVYAASPVLLI-----G--TWFFLYTP-AKEGSS

SRCSRKAGGATWIERT-R-PVEKADGGVIGHASTFTYGRK--GPDKRTGWILVEVRLPG-E----TMCCAKL

YRKSAA-A-AP---PPVSPPHSDK------SDK--

>TraesCS7B02G489500.2

--VLPPGFRFHPTDEEIIKFYVVPKVLDEAFVAAIEDVNLNKYEPWELPEKVKMGEKEWYFYSRKDRKYPTG

IRTNRATEAGYWKATGKDKEIFHP-FTLIGMKKTLVFYKGRAPRGEKTNWIMHEYRLESSASK-EQWVVCRI

FHKST-RLTLPEYDT-SALPHQDQSGLASMLRNDW

>TraesCS7B02G499400.1

---FPAGYHFRPTDEELLDVYLRAKIDGEPPLDVFMDVDILDWDPAEL-RKA-YG--R-YFFTKRTRK----

-KVK-----ASWKATGC------PTGEKIGTKRILTYY-G------GVDWSMNEY---M-TGRLDQWILCTI

QKKTGATKEHPDQY-PTFLPASDF--LD---PADF

>TraesCS7D02G000200.1

MTALPVGFRFRPTDEELVRHYLKAKIAGRAHPLAIPDVDLAAVEPWDLPARS-I-SDEWFFFARRDRKYPGS

SRSCRSTAAGYWKATGKDRLIRAPKGALIGVKKTLVFHRGRAPRGARTPWIMHEYTATDPQSQQNSFVLYRL

FNKQ---DLTPDHSTPMWTP--ES-EE---MAPDW

>TraesCS7D02G008500.1

SNQVPPGFRFHPTDEELVDYYLRKKVASRRIDNVIKDVDLYKIEPWDLQEKCRIGEEDWYFFSHKDKKYPTG

TRTNRATAAGFWKATGRDKPIYA-C--LVGMRKTLVYYKGRAPNGQKSDWIMHEYRLETP--QEEGWVVCRV

FKKRLP--PC-DDP-HMTIPPSDDRVLDKFAKGQW

>TraesCS7D02G034000.1

MET---GFVFSPADHELTDLYLGGQIAGHPVFTFIHHADVYSAAPAEL--VRD-G----------------K

KSKLRTIDDDYWHESG-KKPVEGS---VGGYVQDFVYAK---KDGHRLGWRMKEYGLST-EHG---------

-----------------------------------

>TraesCS7D02G062100.1

MV-VPPGFRFHPTEEELLTYYLAKKVASQRIDDVIPDVDLNKLEPWDIQERCRIGGPDWYLFSHKDKKYPTG

TRTNRATAAGFWKATGRDKAIYSAG--RIGMRKTLVFYKGRAPHGHKSDWIMHEYRLDDPSAQEDGWVICRV

FKKKNILVNCSNHK-AHKLPVVGSDSLDRLVASDW

>TraesCS7D02G062300.1

MV-VPPGFRFHPTEEELLTYYLAKKVASQRIDDVIRDVDLNKLEPWDIQERCRIGGPDWYLFSHKDKKYPTG

TRTNRATAAGFWKATGRDKAIYSAG--RIGMRKTLVFYKGRAPHGHKSDWIMHEYRLDDPSAQEDGWVICRL

FKKKNIAVNCSDHK-GHKLPVV--DALDQL-APDW

>TraesCS7D02G062400.1

MV-VPPGFRFHPTEEELLTYYLAKKVASQRIDDVIPDVDLNKLEPWDIQERCRIGGPDWYLFSHRDKKYPTG

TRTNRATAAGFWKATGRDKAIYSAG--RIGMRKTLVFYKGRAPHGHKSDWIMHEYRLDDPSTQEDGWVICRV

FKKKNIVVNCSDNK-AHKLPVV--DALDRL-PADW

>TraesCS7D02G062600.1

MV-VPPGFRFHPTEEELLTYYLAKKVASQRIDDVIPDVDLNKLEPWDIQARCRIGGPDWYLFSHKDKKYPTG

TRTNRATAAGFWKATGRDKPIYSAG--RIGMRKTLVFYKGRAPHGHKSDWIMHEYRLDDTLAQEDGWVICRV

F-KKNIVANCSDPK-GNKLP----DALDRL-AADW

>TraesCS7D02G063300.1

MV-VPPGFRFHPTEEELLTYYLVKKVASQRIAGVIPDIDLNKLEPWDIQARCRIGGPDWYLFSHKDKKYPTG

TRTNRATAAGFWKATGRDKPIYSAG--RIGMRKTLVFYKGRAPRGHKSDWIMHEYRLDDPSAQEDGWVICRV

FKKKNIIVNCSDHK-GHKLPVV--DALDRL-SADW

>TraesCS7D02G100400.1

M-NLPPGYHFAPTDMELIVHYLRRKMDG--HPPIFKDVPITDYRPEQI-EV-MCGER-WYFFTKRTRKYATG

NRPDRTPGRGYWKATGPQRLIRGP-KALVGRRRTLVFYTG--PDEAKTAWTMYEYNLTS-NDKADEWVLCTI

QRQKG-----G-EE-PTMAPSTNSAGTNKLSTYNL

>TraesCS7D02G154200.1

--KFPQGFRFHPTDVEIITSYLVPKVLNKAFDIAVGEVDLNKCEPWELPEKAKMGEKEWYFFSQKDRKYPTG

IRTNRATTAGYWKATGKDKEIFHHTTSLIGMKKTLVFYKGRAPRGDKTNWVMHEYRLESPSSK-EEYVVCRI

FHKST-GLTLPGYGS-SVLPYQDESGPSSILSSDW

>TraesCS7D02G174400.1

MTTLPPGV-FNPMNEEIVRTYL-NGIAGTS-GAVVIEEDVYGDKPDVLPASTR-S--SWWFRCH--KQATRG

GRGDRSVATGFWKADGE----------HLGFKRTFGFYEH--EGEKKT-WLMEEY-----NVDPDLPALYRI

YRDRKQGRGGP----PAA-PRGDDDDSPEMIPA-W

>TraesCS7D02G189900.1

--NLPPGFRFQPTDMEIITFYLVPKVLKKVFNTVVEEVDLNKCEPWDLLKKVNMGDKGRYFFSQKDLKYSIG

IRTNGATKAGYWKATGKDKEIIHPTMSIIGMKKTLVFYKGRAPKGEKTNWIMHEYRLESPSSK-GEYVVCRI

FHKST-GLTLDDCDS-FVLPQQDQSVSSSILSN--

>TraesCS7D02G196300.1

--NLPAGFRFHPTDVEIITFYLVHKVLKKPFDIVIEEVDLNKCEPWDLPNNVNMGEKDQYFFSKKDLKYPTG

VRTNRATNAGYWKATGKDKEIVHPTMSLVGMKKTLVFYKGRAPRGEKTNWIMHEYRLEMPTSK-EEYVVCRI

FHKST-GLTLADYDS-SVLPHQEQSGTSSITSNDW

>TraesCS7D02G205200.1

MENVFQHFRLNPTEVEAVTYYLPRLIAGET-HKLIHDAHVYDCEPKDLP-QAV-G---RFFFTTC-KAKGRT

TRCARKAGGGTWTNTTT--VVTH-AGVDVGERKNLSFKQG--KS---TGWVMEEYRLLP-PPEADEKVFCKI

HHADEA-RAEPEHGAPSAAP--EE---EMLVST-W

>TraesCS7D02G207400.1

MED-FQHYRLNPTDVEAVTYYLPRLIAGQ-LHKFIHHVDIYSCEPKDLP-QAA-G---RFFLTTR-KKNGSK

TQSVRTAAGGTWTNATT--TVRH-AGVEVGERKNLSFRKG--KS---TGWVMEEYKCL--LPESDVKVFCKI

HHPDAA-R-EPTHQAPSQAPQSEE---EELANTDW

>TraesCS7D02G210600.1

TG-LPKGVKFDPSDQELLG-HLLAKHAGQPHPEFIPTVEICYTHPQKLP-----GKQSSHFFHR--KAYNTG

TRKRRKTS--RWHKTGKTKPVM--G--QHGCKKIMVLYKGG--KPKKTNWVMHQYHLGT----EDEYVVSKL

F--KSALT--PEYAADN---EN--GDGDE-YKDDI

>TraesCS7D02G210900.1

MEDVFHHYRLCPTEVEAVTYYLPRLLSGETLHKLIHRVEISGCEPKDLP-QAV-G---RFFFTTC-KKSGSK

LQSVRGAGAGTWSQKTT--EICH-AGVKVGEVKNLSFKKG--KS---TGWVMEEYRCL--LPESDVKVFCKM

HHADAA-R-QQTHQAPSQAPQSEE---EELANTDW

>TraesCS7D02G211300.1

MEDVFHHYRLSPTEVEAVTYYLPRLLSGETLHKLIHRVEISGCEPKDLP-QAV-G---RFFFTTC-KKNGSK

LQSVRGAGSGTWSQKTT--EICH-AGCKVGEIKNLSFKKG--KS---TGWVMEEYRCL--LPESDVKVFCKM

HHADAA-R-QQ-----------EE---EELTNTDW

>TraesCS7D02G211400.1

MEDVFHHYRLSPTEVEAVTYYLPRLISGQTLHKLIHRVEISGCEPKDLP-QAV-G---RFFFTTC-KKNGSR

LQSVRGAGAGTWTQKTT--EICH-AGVKVGEVKNLSFKKG--KS---TGWVMEEYRCL--LPESDVKVFCKM

HHADAA-R-QQTHQAPSAAP--EE---LEFVST-W

>TraesCS7D02G246100.1

QHGLPPGFRFHPTDEELVTFYLAAKVFNACCGVDIAEVDLNRCEPWDLPEAARMGEREWYFFSLRDRKYPTG

LRTNRATGAGYWKATGKDREVLNA-GSLLGMKKTLVFYRGRAPRGEKTKWVLHEYRLD--SCK-EEWVVCRI

FHKAVDPYQSSQ-SSPSPFP----GPPAQP----W

>TraesCS7D02G263800.1

MELLPPGFRFRPTDEELVVQYLRRKALALPLPAVISVHNLYSLDPWDIP-----G-REKYYFAVRP---AAG

AGGRTTTASGCWKASARERPVVVSRSHLVGVKKSLAFVKGSKPASAQTGWVMHEYRLALPHHHAEEWVVCRI

F-------QTPDA--P---PSS--SSSSSCVSS--

>TraesCS7D02G283800.1

MTSLAPGFRFHPTDEELVSYYLKRKVLGRPLKDAIAEVDLYKVEPWDLPARSRLRSRQWYFFSRLDRKHANR

ARTNRATAGGYWKTTGKDREVRHG-ARVVGMKKTLVFHAGRAPKGERTNWVMHEYRLE--GI-QDSFVVCRI

FQKAGPGPPMPDTN-PEFQPLGEDETLDDGAPSKF

>TraesCS7D02G295100.1

MTKLPLGFRFHPTDEELVRHYLKGKITGQIKNEVIPEIDVCKCEPWDLPDKA-I-SEEWFFFAPKDRKYPNG

SRSNRATEAGYWKATGKDRVIKSKKQHMIGMKKTLVFHRGRAPKGERTGWIMHEYRTTEPEFEEQGYVLYRL

FQKQLE-KGTPDENSPDLLPMDEDDDAQSTLDADW

>TraesCS7D02G302000.1

MSRMPPGFRFHPRDEELVLDYLLHKLT--AY-VDIVDVDLNKCEPWDLPEAACVGGREWYFFSLRDRKYATG

QRTNRATRSGYWKATGKDRAILAHGDALVGMRKTLVFYQGRAPKGTRTEWVMHEFRLE--QLK-EDWVLCRV

FYKSRTTSPLAN-AA-L-----EKSKLELTLSQ-W

>TraesCS7D02G314000.2

MC-VPPGFRFHPTEEELLNYYLRKKVASEEIDDVIRDVDLNKLEPWDIQEKCKIGGPDWYFFSHKDKKYPTG

TRTNRATAAGFWKATGRDKAIYNAK--RIGMRKTLVFYKGRAPHGQKSDWIMHEYRLDDPAGQEDGWVVCRV

FKKKHHHK---KAR-AQKLPPSEDAMMDRL-ASDW

>TraesCS7D02G315100.1

MTEVMPGFRFHPTEEELIEFYLRRKVDGKRFNDLIASVDLYRYDPWDLPALASIGDKEWFFYVPRDRKYRNG

DRPNRVTPSGYWKATGADRMVK-VGNRSIGLKKTLVFYVGKAPKGLRSSWIMNEYRL--PRYQ-E--SLCRV

YKRP----AT-STAS--PPPASNNDELSRANPNDW

>TraesCS7D02G322700.1

--CVPPGFRFHPTEEELVGYYLARKVSSHKIDDIIQEVDLYRIEPWDLQERCGGGDPEYYFFSYKDRKYPSG

TRTNRATAAGFWKATGRDKPVLSSSASVIGMRKTLVFYRGRAPNGRKTDWIIHEYRLQSPT-QEEGWVVCRA

FVKPVPNQSASDNN---DLPPTSSNLWSSL---S-

>TraesCS7D02G329200.1

--SVPPGFRFHPTDEELLYYYLRKKVAYEAIDDVIREIDLNKLEPWDLKDRCRIGGPEWYFFSHKDKKYPTG

TRTNRATTAGFWKATGRDKAIFLGR--RIGMRKTLVFYVGRAPHGKKTDWIMHEYRLDEV--QEDGWVVCRV

FTKKS------DE--IQQLPAEHESILDKLV-AAF

>TraesCS7D02G342300.1

--DLPPGFRFHPTDEELITYYLLRKVVDG-FSRAIAEIDLNKCEPWELPDKATTAEKEWYFYSLRDRKYPTG

LRTNRATGAGYWKATGKDREIRSANGALVGMKKTLVFYRGRAPKGQKTQWVMHEFRLE-PTTR-DEWVIAKI

FVKP--GAPRQDTSS-TYEP------GSRLLQSDW

>TraesCS7D02G348100.1

MEQVPQGYHFVPSDLELIRLLI---IAGRPLPTIFRNV-IREYHPAEL-ERAK-G---IYFFSE--REFP-G

GRPRRFTKDGWWKASGGGEALKRG---TVGSKLTLVFYK-K-PGQKKTDWAIKEYKIVD-KKKAEEMALYRL

YNKKQATQAEP--G-PHLLPFA--QGSQQVVAAD-

>TraesCS7D02G365200.1

-GGLPAGVKFDPTDQELIE-HLEAKVAAPSHPEFIPTIEICYTHPEKLP-----GSKLRHFFHR--KAYTTG

TRKRRKPPRSRWHKTGKTRPVA--G--RQGCKKILVLYKHR--KPEKTNWVMHQYHLGE----EEELVVSKI

FRQ-SSRS---PVRGHESTPGSD-SGLEEV--GPW

>TraesCS7D02G371800.1

MAPVPHGYHFVPDDPELLRLLM---IAGRALPSIFHGV-IRNYHPAEL-ELAK-G---IYFFNE--REFS-G

SRPGRTAKDGWWKASGGGLPLVRR---VVGYKLTLVFYK-R-PGDKKTDWIVKEYIVGP-NKKVSEMALYRL

YKKEKATHAAPDAG-PT-LPFG----TQQVVAAD-

>TraesCS7D02G451700.1

MNRLPPGFRFHPRDDELVLDYLAKKLGGSIY-PTMVDVDLNKIEPWDLPEIAFIGGKEWYFYSLRDRKYATG

QRTNRATESGYWKATGKDRSISRK--LLVGMRKTLVFYQGRAPKGKKTEWVMHEFRKE--PLK-EDWVLCRV

FYKTRATVSLPD-NN-S----AEQVKREAPFSQ-W

>TraesCS7D02G452500.1

MSRLPPGFRFHPLDDELVLDYLSRKL-GSIY-PAMVDVDLNKIEPWDLPEIACIGGKEWYFYSLRDRKYATG

QRTNRATESGYWKATGKDRAISRK--LLVGMRKTLVFYEGRAPKGKKTEWVMHEFRKE--PLK-EDWVLCRV

FYKTRTTISLPDNNN-S----AEQVKREAPFSQ-W

>TraesCS7D02G535300.1

ME--LPGFRFHPTEEELLEFYLKHHVTRQHLKDIIPTVHLYRHDPWDLPGLAAISEREWYFFVPRDRKHASG

GRPSRTTERGFWKATGSDRAVRCAPKRLVGLKKTLVYYQGRAPRGTKTDWVMNEYRL--P---QD--VLCKV

YRKAVSLKAR---SHP----VS--KKEE--VAVEW

>TraesCS7D02G543400.1

--ELPLGFRFHPTDEEIINSYVIPKVLDEAFVAAIEDVNLNKYEPWELPEKAKMGEKEWYFYSRKDRKYPTG

IRTNRATGVGYWKATGKDKEIFHP-LTLIGMKKTLIFYKGRAPTGEKTNWIMHEYRLESSASK-EQWVVCRI

FHKST-GLTFPDYDS-SALPQQDQCGPSSMLSND-

>TraesCS7D02G543500.2

ASVLPPGFRFHPTDEEIIKFYVVPKVLDEAFVAAIEDVNLNKYEPWELPEKAKMGEKEWYFYSRKDRKYPTG

IRTNRATEAGYWKATGKDKEIFQP-FTLIGMKKTLVFYKGRAPRGEKTNWIMHEYRLESSASK-EQWVVCRI

FHKST-GLTLPEYDT-SALPHQDQSGLASMLRNDW

>TraesCSU02G119900.1

MA---MGCRFSPSDADLICAYLRPMIASEPLPRFLHTADAYAADPAALPLAPK-A--RWYFFGSA-KLSGHN

KHRSRAVGGGTWHEKG-RKAMLDGEGRVVGYKQSFRYKI---HADGEAVWLMVEFRMAH-D-QGKVPVLCKV

YRKRSA-S------SPSLMPSDNC-NSDQFQNQDW

>TraesCSU02G120000.1

MAQ---GCRFSPSDADLISFYLRPMIASEPLPRFLHSADAYAADPAALPLAPK-E--RWYFFGSA-KLSGHD

KRRSRAVAGGTWHEKG-RAAVLDGEGRVVGYKQSFRYKV---HADGEAVWLMVEFRMAH-D-QEKVPVLCKV

YRKRSA-S------SPS---SDNY-NSDQFQSQDW

>TraesCSU02G135000.1

MTSLAPGFRFHPTDEELVSYYLKRKVLGRPLKDAIAEVDLYKVEPWDLPARSRLRSRQWYFFSRLDRKHANR

ARTNRATAGGYWKTTGKDREVRHG-ARVVGMKKTLVFHAGRAPKGERTNWVMHEYRLE--GI-QDSFVVCRI

FQKAGPGPPLPDTN-PEFQPLGEDETLDDGAPSKF

>TraesCSU02G137200.2

ME-LPPGYRFHPTDVELTLYYLKRKLLGKKLLNAVAEVDIYKHAPWDLPKSSM--TGQWYFFCTRGRKYSVG

QRANRSTEGGYWKATGKDRQVVYE--RTVGMKRTLVFHAGKAPKGTRTDWVMYEYRLVQAGVRLDDSVLCKV

HKKSGPGPLSPAAH-LDIVPCD--VELNDLVSNDV

>TraesCSU02G174800.1

MEDIFHHYRLSPTEVDAVTYYLPRLLSGETLHKLIHRVEISGCEPKDLP-QAV-G---RFFFTTC-KKNGSK

LQSVRGAGGGTWTQKTT--EICH-AGVKVGEVKNLSFKKG--KS---TGWVMEEYRCL--LPESDVKVFCKM

HHADAA-R-QQTHQAPSQAPQSEK---EELANSDW

>TraesCSU02G215800.1

MSQEP-GFRFHPTGQELVTYDLLRKVHGHHA--FIPEVHVYKHEPWELPDKS-S-STEWYFFAVRARKYPNG

LRMGRATVAGFWKSTGKDRPVMHN-V-IVGMKKTLVFHTGRAPGGTRTDWVMHEYRLQG-RNHQDAYALCRV

FKKNTVA-SVPGGE-PD-------KGKKPFQPVDW

>TraesCSU02G230600.1

MEDVFHHYRLSPTEVDAVTYYLPRLLSGETLHKLIHRANISGCEPKDLP-QAV-G---RFFFTTC-KKNGSK

HQSLRGAGTGTWTQKTT--EICH-AGVKVGEVKNLSFKKG--KS---TGWVMEEYRCL--LPESDVKVFCKM

HHADAA-R-QPTHQAPSAAP--EE---LEF-ST-W

>HORVU0Hr1G000630.1

MA-LPPGFRFHPTDVELVSYYLKRKIMGKKLFQAISEVELYKFAPWDLPKSCL--SKEWFFFCPRDKKYPKG

SRTNRATPNGYWKTSGKDRTIELN--RIVGLKKTLIFHEGKAPKGNRTDWVMYEYKMEDAGFSKDAYVLCKI

FKKSGLGPFAPEDQ-PM------------------

>HORVU0Hr1G005130.1

LKDLPPNS-FDPAPDAIVGRYLRRQIAGKLYPQVVQAADVFAAHPAALP--A--ADREWFFAVHRQHKLKDK

ARPRRA-GPGYVLREGS----------VVGIGYRRVFY--RARRVSRTEWWMEEYGFGKP---XXXXXXXXX

XXX--------------------------------

>HORVU0Hr1G017490.7

--TLPPGFRFYPSDEELVCHYLHGKVAN-RLPGTMVEVDLHVHEPWELPDVAKLSTNEWYFFSFRDRKYATG

LRTNRATRSGYWKATGKDRVIRSPRAAIVGMRKTLVFYRGRAPNGSKTCWVMHEFRIE-P----EDWVLCRV

FHKKKADTHDP---S-LSLS--DF-----ALEVN-

>HORVU0Hr1G026590.1

MEDVFQHCRLNPTEVDAVTYYLPRLLSGETLHKFIHSVEISGCEPKDLP-QAV-G---RFFFTTC-KKNGSK

LQSVRSAGGGTWTQKTT--EISH-AGGKVGEVKNLSFKKG--KS---TGWVMEEYRCL--LPEAEVKVFCRM

HHAAAA-R-ESMQQ--SAAP--DE---DELAKT--

>HORVU0Hr1G028970.1

MATLPPGFRFHPTDEELILHYLRNRAADAPCPTSTSTSSTHRTSPLQ-PRR------QVVLLQPAGQRHAEG

HRHRQA--------------------------------RRRHPGRRR-----QEARLLQ-PAQQD-----RL

DHA------------P-RPPHQ--QRLHEV-----

>HORVU0Hr1G038150.2

---LPAGVKFDPTDLELLE-HLEGKVAA--HVGFIPTIEICYTHPENLP-----GKLISHFFHK--NAYDVG

KRKRRKHSDERWHKTGKSRSIL--G--VIGWKKILVLYKGG-GKTVKTNWRMHQYHLGV----QDELVVSKV

FNNEN---YLPSPTS-E-----EDSGLDEDPQG--

>HORVU1Hr1G020380.1

MAR---GLNFAPGDADLITIYLHRKISGSPLPWYIHDADVYAAEPAALP-SAR-G--RWYIFTSV-RQSSRD

VRRCRAVAGGTWHEKA-RCGVLDGGGALLGYRQPFTYEK---NG-----WLMLEFS------QRRMPVLCKI

YRARSA-S------S-S--PSDNA------TTSSW

>HORVU1Hr1G021800.3

--SLPPEFRFHPTDEELILHYLCNRAAAAPCPPIIADLDIYNFDPWDLPSQAVYGDNEWYFFSPRDRKYPNG

IRPNRAAGSGYWKATGTDKPIYDATGQGVGVKKALVFYKGRLPKGTKTAWIMHEYRLAIVR-----------

-KKEG-AK-DP------------------------

>HORVU1Hr1G025010.2

MEDILQHYRLNPTEVEAVTYYLPRLISGETLHKLIHRVYIYDWEPKNLP-QAV-G---RFFFITC-KKNGSK

LQSVRSAGGGTWAQKTT--EITH-AGGKVGEIKNLSFKKG--KS---TGWVMEEYRCL--LREAEVKVFCRM

HHADAA-R-RPPH-AC-------------------

>HORVU1Hr1G045120.1

---LPVGVKFDPTDLELLE-HLEGKVAA--HVGFIPTIEICHTHPENLP-----GKLISHFFHK--NAYDVG

KRKHRKHSDERWHKAGKSRSIL--G--VIGWKKILVLYKGG-GKAEKTNWRMHQYHLGV----QDELVVSKV

FICK-------------------------------

>HORVU1Hr1G049840.3

--TLPPGFRFHPTDDELVGYYLKRRVDNLKIEEVIPVIDLYKCEPWELPEKSFLPKREWFFFVPRDRKYPNG

SRTNRATTTGYWKATGKDRKVSCD-GAVCGVRKTLVFYKGRAPGGERTDWVMHEYRLCQGNFI-GAYALCRV

IKRTEAGLSSSQQGADFTFSGREQTQEKDG-----

>HORVU1Hr1G063740.1

MSQLPPGFRFHPTDEELVMHYLCRRCAGLPISPIIAEVDLYKFDPWQLPRMALYGEKEWYFFSPRDRKYPNG

SRPNRSAGTGYWKATGADKPVGS--PKPLAIKKALVFYAGKAPKGDKTNWIMHEYRLADSSLRLDDWVLCRI

YNKKGASE--PGKS-PDSMPADSC--ISDWVNPDL

>HORVU1Hr1G064300.1

MSDVMPGFRFHPTEEELIEFYLRRKVEGRRFNELITFLDLYRFDPWELPAMAVIGEKEWFFYVPRDRKYRNG

DRPNRVTASGYWKATGADRMIR--NSRPIGLKKTLVFYSGKAPKGVRSSWIMNEYRL--PLFYSE--SLCRV

YKRS----ARPVSPAP--PPSA--DELSRLLPSDW

>HORVU1Hr1G065570.2

MA-LPPGFRFHPTDVELVSYYLKRKIMGKKLFEAISEVELYKFAPWDLPKSCL--SKEWFFFCPRDKKYPKG

SRTNRATPNGYWKTSGKDRTIELN--RIVGLKKTLIFHEGKAPKGNRTDWVMYEYKMEDAGFSKDAYVLCKI

FKKSGLGPFAPEDQ-PLELYGD--IELDDLLTGDF

>HORVU1Hr1G073900.1

MA-LPPGYRFYPTEEELVRFYLRHMLDGRR-DRVIPVADVCSLDPWQLPEVH--RAGEWFYFCARQEREARG

GRPSRTTPSGYWKAAGTPGLVYSA-GHPVGTKKTMVFYRGRAPAGAKTKWKMNEYRAF-PEVR--EFSLCRL

YTRSGSLR--P---A--PASAANDRSVVQQ---DW

>HORVU2Hr1G004130.1

--SLPSGFRFHPTDEELVGDYLCARAAGRTLPHVIAEVDMYRHDPWELPERALSGAREWYFFTPRDRKYPKG

SHPSRAVGGGYWKATGADRPVART-GRTVGVKKSLVFYHGRPGAAVKTDWIMHEYRLAGGSLRLDDWVLCRL

YNKKNQWE----EAAAESLSMGEVS-LDDL--LDL

>HORVU2Hr1G011370.1

MEELLPGFRFHPTDEELVSFYLKKKIQQKPISELIRQLDIYKFDPWDLPKLASTGETEWYFYCPRDRKYRNS

VRPNRVTAAGFWKATGTDRPIYS-GTRCIGLKKSLVFYKGRAARGMKTDWMMHEFKL--PKSRIDSWTICRI

FKKTSSMAVAPQQQSP-QFG--DQ--LGRLLEA-W

>HORVU2Hr1G011390.1

MEELLPGFRFHPTDEELVSFYLKKKIQQKPISELIRQLDIYKFDPWDLPKLASTGETDWYFYCPRDRKYRNS

VRPNRVTAAGFWKATGTDRPIYS-DTRCIGLKKSLVFYKGRAARGIKTDWMMHEFRL--PKSRIDSWTICRI

FKKTSSTVVAP---SP-HFG--DQ---GRLLVA-W

>HORVU2Hr1G017350.1

MSSLPPGFRFPPTDEEVVTDYLTRKVLHS-FSQVITDVDLNKNEPWDLPGLAKMGEKEWFFFVHKSRKYPTG

TRTNRATERGHWKATGKDKEIYRG--VLVGMKKTLVFYTGRAPSGGKTPWVMHEYRLE-PTAK-DDWAVCRV

FNKD---LDLPDSPSPQFMPGGDQASSSALLFDSW

>HORVU2Hr1G017360.1

MSSLPPGFRFHPTDEEVVTDYLTRKVLHS-FSQVITDVDLNKNEPWDLPGLAKMGEKEWFFFVHKSRKYPTG

TRTNRATERGHWKATGKDKEIYRG--VLVG------------------------------------------

-----------------------------------

>HORVU2Hr1G017370.1

MSSLPPGFRFHPTDEEVVTDYLTRKVLHS-FSQAITDVDLNKNEPWDLPGLAKMGEKDWFFFVHKGRKYPTG

TRTNRATERGYWKATGKDKEIYRG--VLVGMKKTLVFYTGRAPSGGKTPWVMHEYRLE-PTAKQDDWAVCRV

FNKD---MDLPDSPSPQFMPGGDQASSSALFFDSW

>HORVU2Hr1G017380.1

MSVLPRGFRFHPTDEEVVTYYLTPKISNY-SSLVVPDVNINNTEPWDLPSLAKMGEKEWFFFVHKDRKYPTG

TRTNRATRSGYWKATGKDKEIYRGGAVLIGMKKTLVFYLGRAPSGEKTSWVMHEYRLE-PSAK-DDWAVCRL

FNKD---S--PDSP--Q------------------

>HORVU2Hr1G017400.3

MSSLPPGFRFHPTDEEVVTHYLTPKAVNS-FSLVIADVDLNKTEPWDLPGKAKMGEKEWYFFVHKDRKYPTG

TRTNRATERGYWKATGKDKEIFRGDAVLVGMKKTLVFYTGRAPRGDKTPYVMHEYRLE-HSAK-NDWAVCRV

FDKD---LDLPDSPSPQFMPGGDQASSSALFFDSW

>HORVU2Hr1G017460.4

LLSTPREQRVHPLDPRLIVCAVRRRTRSSSCTSATAHLQVRPIGP-PLPG----GDCRWYFFSPRDHKYRNG

MRPNRAASSGYWKATGTDKPIHDATSQGVGVKKALVFYKGRPPKGTKTAWIMQEYRLAAASMRLDDWVLCRI

YNKTGLGS-VPDACMPEHVVYAEE-------SADL

>HORVU2Hr1G017470.1

--SLPPGFRFHPTDEELILHYLRNRAAAAPCPPIIADVDIYKFDPWDLPSQAVYGDCEWYFFSPRDRKYPNG

IRPNRAAGSGYWKATGTDKPIHDATGQGVGVKKALVFYKGRPPKGTKTAWIMHEYRLAAASMRLDDWVLCRI

YKKSGLAS-VPDACMPEHGVYALR--------VDI

>HORVU2Hr1G039640.2

MRELPPGFRFHPTDEELVVHYLKKKAAKVPLPTIIAEVDLYKFDPWELPEKATFGEQEWYFFSPRDRKYPNG

ARPNRAATSGYWKATGTDKPILASGREKVGVKKALVFYRGKPPKGLKTNWIMHEYRLTD-SLRLDDWVLCRI

YKKINKAA---DATM-GAGALASSNR-SR-LSSDL

>HORVU2Hr1G045580.1

MA-LPPGFRFHPTDVELVSYYLKRKIMGKKLFEAISEVELYKFAPWDLPKSCL--SKEWFFFCPRDKKYPKG

SRTNRATPNGYWKTSGKDRTIKLN--RIVGLKKTLIFHEGKAPKGNMTDWVMYEYKMEDAGFSKDAYVLCNI

FKKSGLGPFAPEDQ-PMELYGD--------LRV--

>HORVU2Hr1G077320.1

MA-VPPGFRFHPTDEELLLYYLKKKIGFEKFDEVIREVDLNKIEPWDLQERCRIGAPEWYFFSHKDRKYPTG

SRTNRATTAGFWKATGRDKCIRTS--RKIGMRKTLVFYRGRAPHGQKSDWIMHEYRLEET--SEDGWVVCRV

FKKKCFFKGAGDHD--QGLPPGAEQAMDGF---EW

>HORVU2Hr1G080460.8

ATELPPGFRFHPTDEELITHYLAKKVADR-FALAVSVADLNKCEPWDLPALARMGEKEWYFFCLKDRKYPTG

LRTNRATESGYWKATGKDKDIFRG--ALVGMKKTLVFYTGRAPKGEKSGWVMHEYRLN-SSSK-NEWVLCRV

FKKS---L-LPDMST--FNPGLDMGERERLLTSEW

>HORVU2Hr1G082320.2

MANLIAGCVFQPTGRELVNHYL-PRLGG--VP--IEGVDVLSLPPRKLPHK-R-G--EWFFFAAQPTPPP-G

A-------GGYWVQYGPEKAY-YGG-EAVAFRRRFAYRKG-----EST-WLMKEYRINRP-----VFVVSKV

YRK-----PPPD--S-------EE------VDS--

>HORVU2Hr1G082340.1

MANLGAGYVFQPTGRELVDHYL-PRLGG--FP--IVGVDVLSLRPRELPHK-R-G--EWFFFAAKITPTP-G

A-------GGCWVRYGPEKAY-Y-GREVVAFRRRFAYHNG-----EST-WRMKEYRLNRP-----VFVVHKV

YRK-----PQPD--S-------EEPRLDE------

>HORVU2Hr1G082640.1

FK-LPAGVKFDPTDLELLE-HLEGKVAA--HEDFITTIEICYTHPENLP-----GKVSNHFFNK--NAFDVG

KRKRRKNSDGRWHKTGKSKSIL--G--VIGWKKILVLHKGG-DEDEIFNWRMHQYHLGV----QDELVASKV

FKKQSVTVYPPSPRAPS-----EVSGLDERIQEGW

>HORVU2Hr1G082650.1

FF-LPAGVKFDPTDLELLE-HLEGKVAA--HVGFIPTIEICYTHPENLP-----GKLISHFFHK--NAYDVG

KRKRRKHSDERWHKTGKSRSIL--G--VIGWKKILVLYKGG-GKTVKTNWRMHQYHLGV----QDELVVSKV

FNNEN---YLPSPTS-E-----EDSGLDEDPQGDW

>HORVU2Hr1G085080.1

M------------ENASSSMYL----GG--------DSD------WRL---TK----D-KFFGTLD-ACPNG

ATSNNTSSLG-----GSNDPTSSA-EQ--GMNSSIITH--RVPNEQETSWLIPGFR-------TDDIVLHYL

K-K--------------LRP----RRIEEL---D-

>HORVU2Hr1G087260.6

ISDLSVCFRFRPMENASSSMYL----GG--------DSD------WRL---TK----D-KFFGTLD-ACPNG

ATSNNTSSLG-----GSNDPTSSA-EQ--GMNSSIITH--RVPNEQETSWLIPGFR-------TDDIVLHYL

K-K--------------LRP----RRIEEL---D-

>HORVU2Hr1G089210.2

--TLPPGFRFYPSDQELVCHYLYKKVTN-RASGTLVEVDLHAREPWELPGQSKLTASEWYFFSFRDRKYATG

SRTNRATKTGYWKATGKDREVRSPTRAVVGMRKTLVFYQGRAPNGTKTSWVMHEFRLD-P----EDWVLCRV

FQKQK---SSP---S--APP--DQGGSPALF--GW

>HORVU2Hr1G092030.1

MNHVPPGFRFHPTDEELVDYYLRKKVQLKRIDDIIKDVDLYKIEPWDLQERCKIGEDDWFFFSHKDKKYPTG

TRTNRATTAGFWKATGRDKPIYV-C--LVGMRKTLVFYKGRAPNGQKLDWIMHEYRLETA---DEGWVVCKV

FKKRVA--PF-EHA-H-QLPPRQPRDLDKFATPDW

>HORVU2Hr1G103930.1

SSRLPPGFRFHPRDHELVLDYLCRKLSG----VDMVDVDLNKCEPWELPDAACVGGREWYFFSRHDRKYATG

QRTNRATHTGYWKATGKDRVITGDAAAVVGMRKTLVFYRGRAPRGTKTEWVMHEFRVD--LPE-EDWVLCRV

FYKSTAAAPIAD-S--A-------PKPE--MPQ-W

>HORVU2Hr1G126040.14

MECVPPGFRFHPTEEELVGYYLARKVAAQKIDDIIQEVDLYRIEPWDLQKRC-GGQTEWYFFSFKDRKYPSG

TRTNRATAAGFWKATGRDKPVTSSG--VIGMRKTLVFYRGRAPSGQKTDWIIHEYRLQTPT-QINS-LVCRA

FQKPTPNQAAPDTP-G-SIPPPGDNFLDSL---H-

>HORVU2Hr1G126080.3

MECVPPGFRFHPTEEELVGYYLARKVAAQKIDDIIQEVDLYRIEPWDLQERC-GGQTEWYFFSFKDRKYPTG

TRTNRATAAGFWKATGRDKPVTSSG--VIGMRKTLVFYTGRAPNGRKTDWIIHEYRLQTPT-QEEGWVVCRA

FQKPTPNQAAPDTP-G-SIPPPGDNFLDSL---H-

>HORVU2Hr1G127550.1

MD-LPPGYRFHPTDVELTLYYLKRKLLGKKLHNTVAEVDIYKFPPWELPKSSM--TGQWYFFCTRGRKYSVG

YRTNRSTEGGYWKATGKDRQVVYE--RTVGMKRTLVFHAGKAPKGTRTDWVMYEYRLAQAGARLDDSVLCKV

HKKSGLGPLSPAAQ-PDVVPCD--VELNDLLSNDV

>HORVU3Hr1G014090.1

--ELPPGFRFHPTDEEIITSYLAPKVLNPAFETAIGEVDMNKNEPWELPKKAKMGQNEWYFYCQKDRKYPTG

IRTNRATKVGYWKATGKDKEIFNPTSMLIGMKKTLVFYKGRAPSGEKTNWVMHEYRLEIPSS--KEYVVCRI

FQKNT-------------------SGPSSMPSNDL

>HORVU3Hr1G014100.1

--HLPPGFRFHPTDVEIITSYLVPKVLKKPFDRVVTEVDLNKHEPWELPNMAIMGEKEWYFFSEKDHKYPTG

IRTNRAMTTGYWKATGKDKEIFQPTMSLIGMKKTLVFYMGTTPKGEKTNWIMHEYRLKSPSSK-EDYVVCRI

FHKST-GLTLPDYNS-LLLPHQDQSEPSSILKNDW

>HORVU3Hr1G014140.3

MTGLPPGFRFYPTDEELIVHYLRRRAAAAPCPAVIAEVDIYKLDPWELPSRAVFEDNEWYFFSPRDRKYPNG

VRPNRAAGSGYWKATGTDKPITAGGGEMVGVKKALVFYQGRPPKGHKTNWIMHEYRLADASMRLDDWVLCRI

YKKPN---PSP----PD-PPIASN------MNGD-

>HORVU3Hr1G019530.1

---LPAGVKFDPTDQELIE-HLEAKVEGRSHPEFIPTIDICYTHPEKLP-----GTMLKHFFHR--KAYTTG

TRKRRKTE-GRWHKTGKTRPVM--G--RQGCKKILVLYKHR--KPEKTNWVMHQYHLGD----EEELVVCKI

FRQ-AAVQ---AAEG-DSRPTSD-AGLEEL--G-L

>HORVU3Hr1G032680.1

MEELPPGFRFQPTDQEIIVCYLKKVASAASAVSIIADVDIYKFDPWELPDKAQFGEGEWFFFSPRDRKYPNG

ARPNRTAGSGYWKATGTDKPILAAGARCLGVKKALVFYQGRSPRGTKTEWVMHEYRLLHASMRLDDWVLCRV

RKKGVAVA-------PR------------------

>HORVU3Hr1G036380.1

--ALPPGFGFHPKDTELVAHYLKKKILGQKIEDIIPEVDIYKHEPWDLPAKC-V--QKWHFFAARDRKYPNG

ARSNRATVAGYWKSTGKDRAI---KKRTIGTKKTLVFHEGRPPTGKRTEWIMHEYYIDEPDIQFDNTVVDSV

--SAQLSPMAPDFS-PDQLPGGSEKFVDNNITSNL

>HORVU3Hr1G061560.1

NNQLPAGVKFDPTDQELLE-HLEGKADTKLHPEFIPAIEICYTHPERLP-----GGKLRHFFHR--KAYTTG

TRKRRKTDGGRWHKTGKTRPVF--G--KLGYKKILVLYKQR--KPEKTNWVMHQYHLGS----EEELVVSKV

FRQ-GGAA---GAT------ASSP--AAAL--GAM

>HORVU3Hr1G062150.1

TASSAQRPDFHPSDQVLIKSYLTPRVASGEHPHFTHDADVYTASPGALP-MA--G--KWYFFTVL-PKSAHG

QRRPRTVGTGCWHEAG-VKPVLDG-DHPIGWRQFFSFMK---EEGQRSGWIMVEIGLDH--GQPSEPVLCKV

YRAPVV-E-KRTLHAR-CGVSHSE-VEDDSMTG--

>HORVU3Hr1G080100.7

MMNLPPGFRFHPTDDELVVHYLCRKVAGQPQPPIIAEVDLYKFNPWDLPERALFGSREWYFFTPRDRKYPNG

SRPNRSAGTGYWKATGADKPVAPRGGRTVGIKKALVFYSGRAPRGVKTDWIMHEYRIAQTSL----------

--KAGRMG---GQGGQ-AAPHGEYSAVDEL---DL

>HORVU3Hr1G083330.1

---FPAGVKFDPTDQELLE-HLESKV--NAHTDFIPTIEICYTHPEKLP-----GTKQKHFFHR--KAYTTG

TRKRRKAEGARWHKTGKTRPLM--G--RHGCKKILVLYKTR--KAEKTNWVMHQYHLGD----EDELIVSKV

FRQSAATS---AAQN-------D-AGLEGLIKG--

>HORVU3Hr1G083820.9

MA-LPPGYRFYPTEEELICFYLRNKLDGR--GRVIPVVDVYSVDPLQLSEIH--EGGEWFYFCARQEREARG

GRPSRTTPSGYWKAAGTPGVVYSA-RRPIGLRKTMVFYRGRAPSGNKTKWKMNEYRAFQPQLR--EFSLCRL

YTKSGTLR--P---A--PVPAT--GSMQTL---DL

>HORVU3Hr1G088800.2

LASSAQRPDFHPSDQVLIKSYLTPRVASGEHPHFTHDADVYTASPGALP-MA--G--KWYFFTVL-PKSAHG

QRRPRTVGTGCWHEAG-VKPVLDG-DHPIGWRQFFSFMK---EEGQRSGWIMVEIGLDH--GQPSEPVLCKV

YRAPVV-E-KRTLHAR-CGVSHSE-VEDDSMTG--

>HORVU3Hr1G090440.4

SSDVMPGFRFHPTEEELIEFYLRRKVEGKRFNELITFLDLYRYDPWELPALAAIGEKEWFFYVPRDRKYRNG

DRPNRVTASGYWKATGADRMIR--SSRPIGLKKTLVFYSGKAPKGVRSSWIMNEYRL--PRYHTE--SLCRV

YKRT----AEAGSSPPQVKP--N-DELSTLLPTDW

>HORVU3Hr1G091720.2

MA--APGFAFCPSDSELVSFYLRPRISGKPLPQFLHEADVYATDPASLP-PAK-G--KWYFFSLV-KRSAQD

SRKRRIVGEGSWKERG-N-DVADAEGRTVGRVEKFTYTH---PKEDPPEWLMREFSVEV-D-QCQVLCLCKI

YRFKSA-S------AP---PI-NW---EET----W

>HORVU3Hr1G094170.1

M---------------------------------------YRHDPWELPERALSGAREWYFFMLRDRKYPNG

SRPSRAAGGGYWKATGADRPVART-GRTVGVKKSLVFYHGRPGAAVKTDWIMHEYRLAGGSLRLNDWVLCRL

YNKKNQWE---------------------------

>HORVU3Hr1G095880.1

MEGLPIGFRFRPTDEELLLHYLRRKALSCPLPDIIPVADLARLHPWDLP-----GEAERYFF-----HLPTG

GGAGRAGGSGVWRASGKERLVVAP--RPIGAKRTLVFC----RGGARTGWAMHEYRLL-PSLHKD-WVVCRV

FKKAT-----P----A-DLP----ESRSGG-AS--

>HORVU3Hr1G106070.1

---VKEEAEFDPTEDELVLHFLRPQLRG-RVA-AVVEADPCASPPWEL---ERL-LRGGYFFAARRR---K-

--VRRTPGGGAWMHSGNRRSVT--L----GTMTRYCFYR-GAGAGRSTGWVMSEYEI---------------

-RCRRA-----DQ-I--------------------

>HORVU4Hr1G012030.1

--GLPPGFRFHPTDEELITYYLSRKVSDA-FSRAIADVDLNKCEPWDLPSKASMGEKEWYFFSMRDRKYPTG

IRTNRATESGYWKTTGKDKEIFHG--RLVGMKKTLVFYGGRAPKGEKTSWVMHEYRIQ--PNK-EEWVVCRV

FKKSQ--ISPCDA-S--FAP--NQAAPGLLCS-HW

>HORVU4Hr1G017170.2

VL-LPVGVKFDPTDLELLE-HLEGKAVA--HVGFIPTIEIYHTHPENLP-----GKLISHFFHK--NAYDVG

KRKRRKHSDERWHKTGKSRSIL--G--VIGWKKILVLYKGG-GKAEKTNWRMHQYHLVV----QDELVVSKV

FNDQ--------PI---------------------

>HORVU4Hr1G017430.7

MDQLPPGFRFHPTDEELVMYYLCRKCGGLPIAPVIAEVDLYKLEPWRLPEKAAGGSKEWYFFSPRDRKYPNG

SRPNRAAGTGYWKATGADKPVGS--PRPVAIKKALVFYAGKPPKGVKTNWIMHEYRLADAALRLDDWVLCRI

YNKKGVIEVKPPAKGPESMPTDSPP-GDDWIDGDL

>HORVU4Hr1G051290.1

M--LPPGFRFHPTDEELILHYLRNRAAQSPCPSIIADVDIYKFDPWALPSKASYGDREWYFFTPRDRKYPNG

VRPNRAAGSGYWKATGTDKPIRCSTGESVGVKKALVFYKGRPPKGIKTNWIMHEYRLAAASMRLDDWVLCRI

YKKTSQVS-VP-GYMPENMSMDED------VTN-L

>HORVU4Hr1G051360.3

MSDLPPGFRFHPTDEEIISHYLTPKALDR-FCGVIGEVDLNKCEPWHLPGKAKMGEKEWYFFCHKDRKYPTG

TRTNRATMSGYWKATGKDKEIFRG--ILVGMKKTLVFYLGRAPRGEKTGWVMHEFRLE-PSAK-DEWAVSKV

FNKE---LELPDPPSQHSLP--DLGQTSALLSTAW

>HORVU4Hr1G067270.5

IDSLPRGVQFNPSDSDLLWHLAAEMGNGHPFIEFIKSGDPRDMP----------GRQNLYFFHRK-----SN

NENDKDI---SWQKS-------GPG----G-----AF-NPR------TDWELHRYGIKNT------LVLSKV

FKSKGHALMYPDK-ASGEGPKAPKDSSEKVAPGDW

>HORVU4Hr1G081430.1

MTDDLPGFRFHPTEEELLGFYLSGVALGKKLHDIIGTLNIYRHDPWDLPGLAKIGEREWYFFVPRDRKAGSG

GRPNRTTERGFWKATGSDRAIRNTPKRVIGLKKTLVFYQGRAPRGTKTDWVMNEYRL--P---ED--VLCKV

YRKATPLKACP--SHP----MANPSNMSSLLPADW

>HORVU4Hr1G081440.1

MADDLPGFRFHPTEEELLGFYLSRVALGKKLHDIIGTLNIYRHDPWDLPGLAKIGEREWYFFVPRDRKGGSG

GRPNRTTERGFWKATGSDRAIRSTPKRVIGLKKTLVFYEGRAPRGTKTDWVMNEYRL--P---ED--VLCKV

YRKATPLKACP--SHP----MANPSNM---LPADW

>HORVU4Hr1G081450.1

MAEDLPGFRFHPTEEELLGFYLSRVALGKKLHDIIGTLNIYRHDPWDLPGMAKIGEREWYFFVPRDRKAGCG

GRPSRTTERGFWKATGSDRAIRSTPKRVIGIKKTLVFYKGRAPRGTKTDWVMNEYRL--P---ED--VLCKV

YRKATPLKAC--SSHP----LGNPSNLSS-LPADW

>HORVU4Hr1G083050.3

MDCVPPGFRFHPTDEELVGYYLRKKVASQKIDDVIRDIDLYRIEPWDLTEHCGIGEEEWYFFSFKDRKYPTG

TRTNRATMAGFWKATGRDKAVHE-R--LIGMRKTLVFYKGRAPNGQKTDWIMHEYRLETP--QAEGWVVCRA

FKKRTAY-SNYHAG---ELPPSEDRALDKFE--DF

>HORVU4Hr1G085320.1

--SLPPGFRFHPTDEELIIYYLKSKINGRQIEEIIPEVDLYKCEPWDLPEKSFLPSKEWYFFSPRDRKYPNG

SRTNRATKAGYWKATGKDRKVNSQ--RAVGMKKTLVYYRGRAPHGSRTDWVMHEYRLDEIGLQ-DAYALCRI

FKKTAPGPRSPDDESDQ-FPDTQESQPDDFPDAPV

>HORVU4Hr1G089450.1

--NLPPGFRFHPTDMEIIIFYLVPKVLKKAFDTVVGEVDLKKYEPWNLPNEVNMGEKDRYFFSQKDLKYPTG

IRTNRATNAGYWKATGKDKAILLPTTTLIGMKKTLVFYKGRAPRGEKTNWIMHEYRIEIPSSK-EEYVVCKI

FHKST-GPTLADYDS-SVFPHQNRSSSSCILSNDW

>HORVU4Hr1G089660.1

--SLPPGFRFHPTDEELVGDYLCARAAGRALPHVIAEVDMYRHDPWELPERALSGEREWYFFTPRDRKYPNG

SRPSRAAGGGYWKAIGADRPVARA-GRTVGVKKSLVFYHGRPGAAVKTDWIMHEYRLAGGSIRLDDWVLCRL

YNKKNQWE----EAA---MSRS-------------

>HORVU5Hr1G022190.2

---LPVGVKFDPTDLELLE-HLEGKVAA--HVGFIPTIEICHTHPENLP-----GKLISHFFHK--NAYDVG

KRKRHKHSDETWHKTGKSRSIL--D--VIGWKKILVLYKGG-GKAEKTNWRMHQYHLGV----QDKLVVSKV

F----------------------------------

>HORVU5Hr1G040700.1

----TPGFRFYPTEDELLGFYLRHRLAGTR-PRVIPVVDVYSYHPSHLRAMA--GVQEWFFFCPRAERELRG

GRPARTTPSGYWKATGSPSCVFSSNSKVIGVKRTMVFYQGRAPTGAKTTWKMNEYKAVAPRLS--EFSVCRL

YISTGTLR--P---S----PTV---QHRNY---A-

>HORVU5Hr1G045640.7

MVSLPPGFRFHPTDEEIITCYLLRKFLDS-FVRAVGEVDLNSCEPRDLPGKANMGEKEWYFFVHKDLKYPTG

SRANRATKEGYWKATGKDREIFKPARELVGMKKTLVFYTGRAPRGAKSEWVMHEFRLQ--NPK-DEWVVCKV

FNKK--GEQLPTT-TPNNAPATEQQLPSSASYDG-

>HORVU5Hr1G045650.7

FRALPPGFRFHPTDEELIVHYLGRQAASMPSPPIIAEVNIYKCNPWDLPGKALFGENEWYFFSPRDRKYPNG

ARPNRAAGSGYWKATGTDKAILST-NESIGVKKALVFYRGKPPKGVKTDWIMHEYRL----MRLDDWVLCRI

HKKCGNLP---DSN-CDAGADA--KR-KR--SS--

>HORVU5Hr1G074230.30

-STWPPGFRFSPTDEELVLFFLKRRIAAGR-PPYIADVDVYRSHPSHLPERSALGDKQWFFCSRLDRKYPNG

SRASRTTADGYWKATGKDRSICN-AGRAVGNKKTLVYHHGRAPRGERTDWVMHEYTILAP-ARRESYALYKL

FEKSGVGP--PDDG-PGFAPISEETA----VPA-W

>HORVU5Hr1G074810.1

MESLPPGFRFHPTDEELITYYLRGKIADG-FTRAITEVDLNKCEPWDLPEKAKMGEKEWYFFSLRDRKYPTG

VRTNRATNAGYWKTTGKDKEIFTGAQELVGMKKTLVFYKGRAPRGEKSNWVMHEYRLH--SNK-DEWVVCRI

FAKSP-GVFLPGHDSPHLN-GGG-AGGQAMLAGDW

>HORVU5Hr1G077010.4

LEQLPPGFRFHPTDLELLLQYLRRMALDRPLPAVIPVHAAALPDPWDAA-----G-SEAYFFSLRQ---ASS

GGRRRRAASGYWKATGKEKPVFVQKRLLVGVKTALAFHRGK----SRTDWVMHEYRLA--NKRPQEWVVCRV

SLK-----ET-DH------P----SSTSSCVSS--

>HORVU5Hr1G085220.20

MEEIMPGFRFHPTDEELVSFYLKKKIQQKPISELIRQLDIYKFDPWDLPKLASTGETDWYFYCPRDRKYRNS

ARPNRVTAAGFWKATGTDRPIYS-GTKCIGLKKSLVFYRGRAARGIKTDWMMHEFRL--PK-RIDSWTICRI

FKKTSSMA-APQQQSP-QFG-QNQ---GRLLGA-W

>HORVU5Hr1G087840.5

--GLPPGFRFHPTDEELVNYYLKRKIHGLHIEDIIPEVDLYKCEPWDLAEKSFLPSREWYFFGPRDRKYPNG

FRTNRATRAGYWKSTGKDRRVMTQGARAIGMKKTLVYYRGRAPQGVRTDWVMHEYRLDDDAIQ-DTYALCRV

FKKNAICAGACEYQSDQ------------------

>HORVU5Hr1G087850.4

LDGLPPGFRFHPTDEELVNYYLKRKVHGQSIEDIIPEVDLYKCEPWELAEKSFLPSREWYFFGPRDRKYPNG

CRTNRATRAGYWKSTGKDRSINYQ--RSIGMKKTLVFYQGRAPQGIRSNWVMHEYRIEENGVQ-DSYALCRV

FKKNVPAGGNQ----DQ------------------

>HORVU5Hr1G099390.2

MEDVLPGYRFHPTDEELVTFYLRRKVARKSLREVIREMDIYKHDPWDLPKASTVGEKEWYFFCLRGRKYRNS

IRPNRVTGSGFWKATGIDRPIYG-SGVSIGLKKSLVYYRGSAGKGTKTDWMMHEFRL--PSMQA-----VSA

GRRVHSIS---ELAPP---P----ERCTRRAGG--

>HORVU5Hr1G099460.1

MEDVLPGYRFHPTDEELVTFYLRRKVARKSLREVIREMDIYKHDPWDLPKASTVGEKEWYFFCLRGRKYRNS

IRPNRVTGSGFWKATGIDRPIYG-SGVSIGLKKSLVYYRGSAGKGTKTDWMMHEFRL--PSMQAVSRPRRRI

FRRTITYRAAPQYTTP---PPS--EEIARLVTDDF

>HORVU5Hr1G099470.2

MEDVLPGYRFHPTDEELVTFYLRRKVARKSLREVIREIDIYKHDPWDLPKASTVGEKEWYFFCLRGRKYRNS

IRPNRVTGSGFWKATGIDRPIYSASGVSIGLKKSLVYYRGSAGKGTKTDWMMHEFRL--PSMQAEVWTICRI

FRRTITYRAAPQYSTP---PLS--EEIARMVTDDC

>HORVU5Hr1G111440.2

MGN-IPGVRFVPTDVE-IIWYLRRKYRGQKMPDFIQEFDVFEDHPDKIQEK--YGEG-WYVFSSRNRRYKNG

TRPGRSGKVGFWKSTSIEKDVKEN-NAKIGRTNELTFMLGHQPKGTSTPWRIKEYRMEEPSMLLDPWVLCKL

YRTKDKPA---DEV-P-NGPCY--FSLDDF-----

>HORVU5Hr1G111590.2

MPNLPPGFRFHPTDDELVEHYLCRKAAGQRLPPIIAEVDLYRFDPWALPDRALFGTREWYFFTPRDRKYPNG

SRPNRAAGNGYWKATGADKPVAPR-GRTMGIKKALVFYAGKAPKGVKTDWIMHEYRLADASLRLDDWVLCRL

YNKKNEWEMEPNTSSPQQATQSEAS-YDDILDMDF

>HORVU5Hr1G121660.1

M--L------APGDDLLVR--LRHRVTGEPFASSIREADVCSAPPADL-DHG-MGGKAYFFCSPANTVHP-G

VRRKRKVAAGFWHEAGQ-VPILDP-KKSVGHKRKLSY---KKPPGSKANWIMVE-GLEQ------QLVLCKV

YRSTEAAPRKPGNSSPN--------------HT--

>HORVU5Hr1G121710.2

MTQL-----AAQLDSLLAS--LRRRV-GEPFHSFIHETDAYSASPADL-RHA-TGGKAWYFCSP---VAP-G

-RRRRQVAAG-WHETGK-KTILGR-KKSVGYKRTLSY---KKPRGAKPNWCMVE-GLEQ----CDQLLLCKV

YRTASSAP--P----P---P----------VGR-W

>HORVU6Hr1G019380.2

MRELPPGFRFHPTDEELVVHYLKKKAAKAPLPTIIAEVDLYKFDPWELPEKATFGEHEWYFFSPRDRKYANG

ARPNRAATSGYWKATGTDKPILASGREKVGVKKALVFYRGKPPRGLKTNWIMHEYRLTG-SLRLDDWVLCRI

YKKTSKAA---DATM-GAGALASSNR-SR-LSSDL

>HORVU6Hr1G038560.1

---LPAGVKFDPTDLDLLE-HLEGKVAA--HVGFIPTIEICHTHLENLP-----GKLISHFFHK--NAYDVG

KRKRRKHSDERWHKIGKSRSIL--G--VIGWKKILVLYKGG-GKAEKTNWRMHQYHLGV----QGELVVSKV

F----------------------------------

>HORVU6Hr1G053540.1

MA-LPAGVKFDPSDLELLE-HLEQKIGGEPHMEFIPTVEICYSHPENLP-----GTKRAHFFHI--NAYGCG

QRKRRRNSDERWHKTGKSKAIY--G--VMGWKKILVLYTGG--KPDKADWVMHQYHLGL----EKEFVVCKI

FNQKYEMAKTPSPEDPD-------TSLEEPYDGSC

>HORVU6Hr1G060800.3

MDHVPPGFRFHPTDEELVDYYLRKKVASKKIDDVIKDVDLYKIEPWDLQEKCKIGEEDWYFFSHKDKKYPTG

TRTNRATSAGFWKATGRDKPIYT-C--LVGMRKTLVFYKGRAPNGQKSDWIMHEYRLETA---EEGWVVCRV

FKKRVA--PC-DHT-PQQLPPKSNRLLDKFTNPSW

>HORVU6Hr1G073200.1

ME-LPPAFKFDPTDDDIVAYYLLPRALG-PHA-AIIEDDPGSAPPWEL---RR-GGGDALFYGPP------G

RRRKRTVGGGVWQGQKVIATLL-PG----AKRYDLTFKK-------SMGYVMHEYEITSP--------LSRV

KK----GKELP---SAG--P--NS------APW--

>HORVU6Hr1G075830.1

MEEMLPGFRFHPTDEELVRFYLKRKIQQKSLPELIRQLDIYKFDPWDLPKLASTGEKEWYFYCPRDRKYRNS

TRPNRVTGAGFWKATGTDRPIYS-GSKCIGLKKSLVFYKGRAAKGVKTDWMMHEFRL--PQKKLESWAICRI

FKKTNATA------A--------------------

>HORVU6Hr1G086870.1

------G--------------------------FIPTIEICHTHPENLP-----GKLIIHFFHK--NAYDVG

KRKRRKHNDERWHKTGKSKSIL--G--VIGWKKILVLYKGG-GKAEKTNWRMHQYHLGI----QDELVVSKV

FNGK-------------------------------

>HORVU6Hr1G091230.1

ME-TAPAFKFDPTDADLVAHYLLPRAVGPPFA-AIIDDDPASLPPADL---AK-GGGHAFFLQTT-----DP

ESRERGVGGGRWRGQKATVTLV-PG----GRRSELTYEDGGAGAAATTGWVMHEYQIVSP--------LSRI

-RE-------P---A-------N------------

>HORVU6Hr1G093050.4

--PMAPGFRFHPTDEELVSYYLRRRVLGRRLRDAIAEVDLYRLEPWDLPPLSRIRSRQWYFFARLDRKVAGA

GRTNRATPRGYWKTTGKDREVSHR-GRLVGMKKTLVFHAGRAPKGDRTNWVMHEYRLLD----QDMHVVCRI

FQKVGSGPNTPDTS-SEYYPANSDD--DDIASSNL

>HORVU7Hr1G000140.6

GSQVPPGFRFHPTDEELVDYYLRKKVASRRIDNVIKDVDLYKIEPWDLQEKCRIGEEDWYFFSHKDKKYPTG

TRTNRATAAGFWKATGRDKPIYA-C--LVGMRKTLVYYKGRAPNGQKSDWIMHEYRLETP--QEEGWVVCRV

FKKRLP--PCXXXX-HMTIPPSDDRVLDKFAKGQW

>HORVU7Hr1G000910.1

MTALPVGFRFRPTDEELVRHYLKAKIAGRAHPLAIPDVDLAAVEPWDLPARS-I-SDEWFFFARRDRKYPGS

SRSCRSTAAGYWKATGKDRLIRGPKGALIGVKKTLVFHRGRAPRGARTPWIMHEYTAVSAGSQQNSFVLYRL

FNKQ---DLTPDHSTPMWTP--ESDEEE--IPPD-

>HORVU7Hr1G012730.2

-----------------LTYYLAKKVASQRIDDVIPNVDLNKLEPWDIQERCRIGGPDWYLFSHKDKKYPTG

TRTNRATTVGFWKATGRDKAIYSGG--RIGMRKTLVFYKGRAPHGHKSDWIMHEYRLDAPSAQEDGWVICRV

FKKKNIVVNCSDHR-AH------------------

>HORVU7Hr1G012760.6

IMVVPPGFRFHPTEEELLTYYLAKKVASQRIDDVIPDVDLNKLEPWDIQECCRIGGPDWYLFSHKDKKYPTG

TRTNRATTVGFWKATGRDKAIYPAG--HIGMRKTLVFYQGRAPHGHKSDWIMHEYRLEDRSAQEDGWVICRV

FKKKNIVVNCSDHK-AHKLPVV--DALDRL-AADW

>HORVU7Hr1G012780.1

IMVVPPGFRFHPTEEELLTYYLAKKVASQRIDDVIPDVDLNKLEPWDIQERCRIGGPDWYLFSHKDKKYPTG

TRTNRATAAGFWKATGRDKAIYSAG--RIGMRKTLVFYKGRAPHGHKSDWIMHEYRLDDPSAQEDGWVICRV

FKKKNIVVNCSSHK-AHKLPVVGNDTLDRLVASDW

>HORVU7Hr1G022310.2

M-NLPLGYHFAPTEEELIVHYLRRKMDG--HPPIFKDVPVIDYRPEQI-EE-RSGDR-WYFFTKRTRKYATG

SRPDRTPAGGYWKATGPQKDIFGG-KQ-VGRRRALVFYYG--PDD-KTDWSMYEYNITS-KDKVDEWVLCTI

QRQKGGGR--P-EE-PTMTPGMNNAGTNKLSTNNL

>HORVU7Hr1G031260.1

--KFPTGFRFHPTDVEIITSYLVPKVLNKAFNIAVGEVDLNKCEPWELPEKANMGEKEWYFFSQKDRKYPTG

IRTNRATAAGYWKATGKDKEVFHQTTSLIGMKKTLVFYMGRAPRGEKTNWVMHEYRLESPSSK-EEYVVCRI

FHKST-GLALPGYGS-SMLPYQDESGPSSILSSDL

>HORVU7Hr1G033990.1

MA-LPPGFRFHPTDVELVSYYLKRKILGKKLFEAISEVELYKFAPWDLPKSCL--SKEWFFFCPRDKKYPKG

SRTNRATPNGYWKTSGKDRTIKLN--RIVGLKKTLIFHEGKAPKGNMTDWVMYEYKMEDAGFSKDAYVLCNI

FKKSGLGPFAPEDQ-PMELYGD--------LRV--

>HORVU7Hr1G039700.1

--NLPQGFRFHPTDMEIITFYLVPKVLKKPFDMVIEEVDLNKCEPWDLPNQVNMGDMAQYFFSRKDLKYPTG

VRTNRATNAGYWKATGKDKEIFHPTRSLIGMKKTLVFYKGRAPRGEKTNWIMHEYRLETPSSK-EEYVVCRI

FHKST-GLTFANYSS-SVLPHQDQSGTSSIVNNDW

>HORVU7Hr1G040560.1

MEDIFQHYRLNPTEVEAVTYYLPRLLSSDTLHKLIHDVEISGCEPKDLP-QAA-G---RFFFTTS-KKNGSK

LQSVRTAGGGTWTQKTT--QISH-AGSKVGEVKNLSFKKG--KS---TGWVMEEYRCL--LPEAEVKVFCRM

HHAAAV-R-EL-----SAAP--DE---EEL---EW

>HORVU7Hr1G041340.1

MENVFQHYRLNPTDVEAVTYYLPRLLAGETLHKLIHRADVYDCEPKDLP-QAV-G---RFFFTPC-KKKGRT

TRCARTAGGGTWTNTTT--VIRH-AGVEVGERKNLSFKKG--KS---TGWVMEEYRLL--PPKADEKVFCKI

HHPDEA-RPEPEHGATSAAP--EE---QTLVSTDW

>HORVU7Hr1G042420.1

-TGLPKGVKFDPSDQELLG-HLLAKHAGQPHPEFIPTVEICYTHPQKLP-----GKQSSHFFHR--KAYNTG

TRKRRKTG--RWHKTGKTKPVI--G--QHGCKKIMVLYKGG--KPKKTNWVMHQYHLGT----EDEYVVSKL

FKPKSALT--PEYAADN---EN--GDGDE-YKDDI

>HORVU7Hr1G042560.1

MEDVFQHCRLNPTEVDAVTYYLPRLLSGETLHKFIHSVEISGCEPKDLP-QAV-G---RFFFTTC-KKNGSK

LQSVRSAGGGTWTQKTT--EISH-AGGKVGEVKNLSFKKG--KS---TGWVMEEYRCL--LPEADVKVFCRM

HHAAAA-R-EPTHQA-SAAP--DE---DELAKIEW

>HORVU7Hr1G042600.3

MEDVFQHCRLNPTEVDAVTYYLPRLLSGETLHKFIHSVEISGCEPKDLP-QAV-G---RFFFTTC-KKNGSK

LQSVRSAGGGTWTQKTT--EISH-AGGKVGEVKNLSFKKG--KS---TGWVMEEYRCL--LPEADVKVFCRM

HHAAAA-R-ESMQQA-SAAP--DE---DELAKTEW

>HORVU7Hr1G049450.1

MEDIFQHYRLNPTEVEAVTYYLPRLLSGETLHKLIHDVEISGCEPKDLP-QAA-G---RFFFTTC-KKNGSK

LQSVRSAGGGTWTQKTT--EISH-AGAKVGEVKNLSFKKG--KS---TGWVMEEYRCL--LPEAEVKVFCKM

HHAAPA-R-EPR--SPSAAP-------EEL---EW

>HORVU7Hr1G055080.1

MELLPPGFRFRPTDEELVVQYLRRKALGLPLPAVISVHNLHSLDPWDIS-----G-REKYYFAVRP---AAG

AGGRTPTASGCWKASARERPVVVSRNHLVGVKKSMAFVKGSKPALAQTGWVMHEYRLALPHHHGEEWVVCRI

F-------QTPDG--P---PAS--SSSSSCVSS--

>HORVU7Hr1G060310.1

MTSLAPGFRFHPTDEELVSYYLKRKVLGRPLKDAIAEVDLYKLEPWDLPARSRLRSRQWYFFSRLDRKHANR

ARTNRATAGGYWKTTGKDREVRHG-ARVVGMKKTLVFHAGRAPKGERTNWVMHEYRLE--GI-QDSFVVCRI

FQKAGPGPPLPDTN-PEFQPLGEDETLDDGAPSKF

>HORVU7Hr1G070780.5

MTKLPLGFRFHPTDEELVRHYLKGKITGQIKNEVIPEIDVCKCEPWDLPDKA-I-SEEWFFFAPKDRKYPNG

SRSNRATEAGYWKATGKDRVIKSKKQHMIGMKKTLVFHRGRAPKGERTGWIMHEYRTTEPEFEEQGYVLYRL

FQKQVE-KGAPDENSPDLLPMDEDDDAESTLDADW

>HORVU7Hr1G072670.3

MSKMPPGFRFHPRDEELVLDYLLHKLT--AY-VDIVDVDLNKCEPWDLPEAACVGGREWYFFSLRDRKYATG

QRTNRATRSGYWKATGKDRAILAHGEALVGMRKTLVFYQGRAPKGTRTEWVMHEFRLE-KQLK-EDWVLCRV

FYKSRTTSPLAD-AT-L-----EKSKLELPLPQ-W

>HORVU7Hr1G075550.1

MC-VPPGFRFHPTEEELLNYYLRKKVASEEIDDVIRDIDLNKLEPWDIQEKCKIGGPDWYFFSHKDKKYPTG

TRTNRATAAGFWKATGRDKAIYNAK--RIGMRKTLVFYKGRAPHGQKSDWIMHEYRLDDPSGQEDGWVVCRV

FKKKHHHK---KTR-AQKLPPSEDAMMDRL-ASDW

>HORVU7Hr1G076250.1

MTEVMPGFRFHPTEEELIEFYLRRKVDGKRFNDLIASVDLYRYDPWDLPALASIGDKEWFFYVPRDRKYRNG

DRPNRVTPSGYWKATGADRMVK-VGNRSIGLKKTLVFYVGKAPKGLRSSWIMNEYRL--PRYQ-E--SLCRV

YKRP----AT-STAS--TPPASNNDELSRANPNDW

>HORVU7Hr1G079500.1

--SVPPGFRFHPTDEELLYYYLRKKVAYEAIDDVIREIDLNKLEPWDLKDRCRIGGPEWYFFSHKDKKYPTG

TRTNRATTAGFWKATGRDKAIFLGR--RIGMRKTLVFYVGRAPHGKKTDWIMHEYRLDEV--QEDGWVVCRV

FTKKS------DE--TQQLPAELDSILDKLV-AAF

>HORVU7Hr1G082420.2

ITXLPPGFRFHPTDEELITYYLLRKVVDG-FSRAIAEIDLNKCEPWELQDKAATAEKEWYFYSLRDRKYPTG

LRTNRATGAGYWKATGKDREIRSANGALVGMKKTLVFYRGRAPKGQKTQWVMHEFRLE-PTTR-DEWVIAKI

FVKP--GAPRQDTSS-TYEP------GPRLLQSDW

>HORVU7Hr1G106480.1

MNRLPPGFRFHPRDDELVLDYLSRKL-SSIY-PAMVDVDLNKIEPWDLPEIACIGGKEWYFYSLRDRKYATG

QRTNRATESGYWKATGKDRSISRK--LLVGMRKTLVFYQGRAPKGKKTEWVMHEFRRE--PLK-EDWVLCRV

FYKTRATISLPD-NN-S----AEQVKREAPFSQ-W

>HORVU7Hr1G106660.9

MSRLPPGFRFHPRDDELVLDYLSRKL-GXIY-PAMVDVDLNKTEPWDLPEIACIGGKEWYFYSLRDKKYATG

QRTNRATESGYWKATGKDRLISRK--LLVGMRKTLVFYEGRAPKGKKTEWVMHEFRKE--PLK-EDWVLCRV

FYKTRTTISLPD-NN-S----TEQVKREAPFSQ-W

>HORVU7Hr1G120780.3

MA-A-RPPLFIPSDLEAIELLRLWKGGAAAVP--IRPAEVYGHSPSELP-TA--G--VWFLVTTV-RVGPKD

PRICRVVKGGTWKEHS-H-PLVSLPDRLVGTVRSLSYTKG--DDGKRTGHLMREYRLSG-GAGGSEIALCVV

YRDEAA-A------KPAATPAASA------LMP-S

>HORVU7Hr1G122680.1

ASELPPGFRFHPTDEEIIKFYVVPKVLDEAFVAAIEVVNLNKYEPWELPEKAKMGENEWYFYSRKDRKYPTG

IRTNRATDVGYWKATGKDKEIFDP-LTLIGMKKTLVFYKGRAPRGEKTNWVMHEYRIESTASK-EQWVVCRI

FHKST-GLTLPDYDT-SALPHQDQSGMSSMLRNDW

>Os01t0104200-00

MTGLPPGFRFHPTDEELLLHYLGKRAAAAPCPPVIAEVDIYKYNPWELPAMAVFGESEWYFFSPRDRKYPNG

VRPNRAAGSGYWKATGTDKPISISQTVLLGVKKALVFYRGRPPKGTKTSWIMHEYRLANSSMRLDEWVLCRI

YKKK-------D--------QQPSTA----ISITF

>Os01t0104500-01

--DLPPGFRFHPTDEEIITFYLAPKVVDRGFCAAIGEVDLNKCEPWDLPGKAKMGEKEWYFYCQKDRKYPTG

MRTNRATEAGYWKATGKDKEIFRD-HMLIGMKKTLVFYKGRAPKGDKTNWVMHEYRLADPPRQ-DDWAVCRI

FHKSS-GIIIQQMQM-STLPQQGGSEPSSLQNNDW

>Os01t0191300-01

---LPAGVKFDPTDQELIE-HLEAKVEGRSHPEFIPTIEICYTHPEKLP-----GTRLKHFFHR--KAYTTG

TRKRRKTE-GRWHKTGKTRPVM--G--RQGCKKILVLYKHR--KPEKTNWVMHQYHLGD----EEELVVCKI

FRQTAAQD--CAAEG-NSRPASD-AGLEEL--GQW

>Os01t0261200-01

--VLPPGFGFHPKDTELISHYLKKKIHGQKIEEIIPEVDIYKHEPWDLPAKC-V--QKWHFFAARDRKYPNG

SRSNRATVAGYWKSTGKDRAI---KKQTIGTKKTLVFHEGRPPTGRRTEWIMHEYYIDEPNLDFDSLA----

--SADLSPLAPDFM-PDWSPMKSEKF-ENSVASNW

>Os01t0393100-01

--DLPPGFRFHPTDEEIITFYLAPKVVDRGFCAAIGEVDLNKCEPWDLPGKAKMGEKEWYFYCQKDRKYPTG

MRTNRATEAGYWKATGKDKEIFRN-HMLIGMKKTLVFYKGRAPKGDKTNWVMHEYRLADPPRQ-DDWAVCRI

FHKSS-GIIIQQMQM-STLPQQAGSEPSSL---DH

>Os01t0667000-00

---------------------LRPRVVSGDKPGFIHEADVYSADPADLP-VAR-G--EWYFFSAV-R-GLKG

GRKARTVDDGCWHEAG-AKPVLAASGRRLGHRQSFSFIKD--DDGQRSGWLMVELSLDV-DEE--QLVLSKV

YRAGAR-K------IP------SI------FTED-

>Os01t0672100-02

VEALPAGVKFDPTDQELLE-HLEGKADAKLHPEFIPTIEICYTHPERLP-----GRQPPALLPP--EG----

------DGAAAHRRAGRRDAVA---------------------DRQDAAGVHRRQAEGV----ED------A

LAQEDQDAVPP-------------------VQGG-

>Os01t0675800-01

-------ADFHPTDQELVTKYLRRHVDSGGNPRYVHEADVYAADPDDLP-VA--G--RWYFFTTV-RKSTGG

QRRARAVGDGCWHEAG-AKDVVGGSPRPIGRRQFFSFVK---EGPRRSGWIMVEIGLKY--AQSSELVLCKV

YRAP-----APDVPKR-CRASDS--IEDE--TA--

>Os01t0816100-01

MENLPPGFRFHPTDEELVVHYLCRKVARQPLPPIIAEVDLYKLDPWDLPEKALFGRKEWYFFTPRDRKYPNG

SRPNRAAGRGYWKATGADKPVAPK-ARTVGIKKALVFYSGKAPRGVKTDWIMHEYRLADASQKLDEWVLCRL

YNKKNNWEAAPGEMTSD-----EDN-FDELMNLDS

>Os01t0862800-01

MA-RAPGFRFYPTEEELICFYLRNKLDGR--DRVIPVFDVYSVDPLQLSEIH--HGEEWFYFCPRQEREARG

GRPSRTTPSGYWKAAGTPGVVYSA-RRPIGMKKTMVFYRGRAPSGTKTAWKMNEYRAF-PQLR--EFSLCRL

YTRSGGIR--P------PGPAA--SSMQQL---DL

>Os01t0884300-01

MSQLPPGFRFHPTDEELVMHYLCRRCAGLPIAPIIAEIDLYKFDPWQLPRMALYGEKEWYFFSPRDRKYPNG

SRPNRAAGSGYWKATGADKPVGS--PKPVAIKKALVFYAGKAPKGEKTNWIMHEYRLADSSLRLDDWVLCRI

YNKKGGLE--PAAMGPDSMPADEF--ISEWINPDL

>Os01t0888300-01

MNDVMPGFRFHPTEEELIEFYLRRKVEGKRFNELITFLDLYRYDPWELPAMAAIGEKEWFFYVPRDRKYRNG

DRPNRVTASGYWKATGADRMIR--NNRPIGLKKTLVFYSGKAPKGVRSSWIMNEYRL--PRYHTE--SLCRV

YKRT----AAPGSSPPQAKPSSS-DELSSLLPTDW

>Os01t0925400-01

MAGLPIGFRFRPTDEELLLHYLRRKVMSRPLPDVIPVADLARLHPWDLP-----GEGERYFF-----HLPTG

GGGSRAGGGGAWRASGKEKLVVAP-KRPVGAKRTLVFF----RGGARTDWAMHEYRLL-PDDHNDVWVVCRV

FKKTTTLASPP----P-DMP----DGGDAGVAS--

>Os01t0946200-01

M-GIREDEGFEPTEDELMLHFLRPQLRG-RVA-AVVEADPCGAAPWEL---AR---REGFFFSARAR---K-

PSVRRTVGGGAWMHSSTGQSVT--L----GCRINYCFYRGMGQQ--STGWMMAEYEI---------------

-RCRRA-----DD-S-A--P-----------PAD-

>Os02t0214500-01

MT-MPPGFRFQPTDEQLVVDYLQRRTAA----D-ITDIDVYNVDPWQLPAMA-M-GSDRYFFT---R---E-

-QARRTTPSGFWKPTGTKKTIFAG-EVPTAVKRRFVFYLGHQPSGSKTSWIMHEYRLMN-------------

--------AVPR----E--PLE-----------D-

>Os02t0252200-01

--AVPPGFRFHPTDEELLYYYLRKKVAYEAIDDVIREIDLNKLEPWDLKDRCRIGGAEWYFFSHKDKKYPTG

TRTNRATVAGFWKATGRDKAIFLGT--RIGLRKTLVFYTGRAPHGKKTDWIMHEYRLDDV--PEEGWVVCRV

FKKKSI-----DER-PQHLPADLDSILDKLV-AAF

>Os02t0285900-00

MA-LSPGFKFNPSDQMLVELFLLPYLIDGELPLVFVHLGGLPLPPWIL-----LGDEEAYFVAPMG---ADG

ARQVRSVGGGKWVRSEGKGEVVAP-----GENFSLNFHRD-DRRSGSTGWVMHEYRVPDPSNQQEAYTD-QI

QQQ----E-FPEESSPEILPSQEASWQEPF---NY

>Os02t0555300-01

--NLPPGFHFFPSDEELVVHFLRRKVSL----DIIPTLLPHRYNPWELNGKA-LG--QWYFFCHLT----Q-

---SRTSSNGHWSPIGVDETVRSG-N--VGLKKTLLFSIGEPSEGIRTNWIMHEYHLLDSSHR-ENWVLCRV

FESSCGSQ--------E-------DDYDEV-----

>Os02t0579000-01

RQDLPPGFRFHPTDEELITHYLLRKAADAGFARAVGEADLNKCEPWDLPSRATMGEKEWYFFCVKDRKYPTG

LRTNRATESGYWKATGKDREIFRG--ALVGMKKTLVFYTGRAPRGGKTGWVMHEYRIH-ASKD-QEWVLCRV

FKKS---LSMP---APQLLPAM--LSGA--LTSHW

>Os02t0594800-01

MA-LPAGVKFDPSDLELLE-HLEQKIGGKPHTEFIPTIDICYSHPENLP-----GKKTGHFFHR--NAYGCG

QRKRRKNCVERWHKTGKSKAIV--G--VTGWKKIMVLYRGA--KPDKANWVMHQYHLGA----EDELVVSKI

SKQKSENANTPSPES-A-----REPNLDESVYGEW

>Os02t0643600-00

MDHVPPGFRFHPTDEELVDYYLRKKVASKKIDDVIKDVDLYKIEPWDLQEKCKIGEEDWYFFSHKDKKYPTG

TRTNRATGAGFWKATGRDKPIYA-C--LVGMRKTLVFYKGRAPNGQKSDWIMHEYRLETT---EEGWVVCRV

FKKRVA--PC-DHA-HQHLPPKHDRLLDKFMS-EW

>Os02t0745250-00

MDEMLPGFRFHPTDEELVRFYLRRKIQQKSLPELIRQLDIYKYDPWDLPKLASTGEKEWYFYCPRDRKYRNS

TRPNRVTGAGFWKATGTDRPIYS-GSKCIGLKKSLVFYKGRAAKGVKTDWMMHEFRL--PQKKLES------

-----------------------------------

>Os02t0810900-01

--TLPPGFRFYPSDEELVCHYLHNKVVNHRFAGTMVEVDLHTHEPWELPDVAKLSTNEWYFFSFRDRKYATG

LRTNRATKSGYWKATGKDRVIHNPRASIVGMRKTLVFYRGRAPNGVKTNWVMHEFRME-P----EDWVLCRV

FYKKKAETHDP---HHHSLN--DFVAASASL--N-

>Os02t0822400-01

--PLAPGFRFHPTDEELVSYYLRRRILGRRLRDAIAEVDLYRLEPWDLPSLSRIRSRQWYFFARLDRKVTGA

GRTNRATPRGYWKTTGKDRDVHHR-GKLVGMKKTLVFHSGRAPKGQRTNWVMHEYRLLD----QDLHVVCRI

FQKNGSGPNMPDTD-SENWPVNSDDAGDDLASSHL

>Os03t0109000-01

ISRLPPGFRFHPSDEELVGYYLRNKQQQQQTASMLVEVDLHACEPWDLPEVAKVGSDEWYFFSWRERKYATG

WRRNRASKQGYWKATGKDKPILHP--TVAGARKTLVFYSGRAPNGRKTAWVMHEFRLL-PQEG-DDWVLCRV

FRKGN-----P---S-SQLPQHQQQAPTSFIA-D-

>Os03t0119966-01

--SLPPGFRFHPTDEELIIYYLKRKINGRQIEEIIPEVDLYKCEPWDLPEKSFLPSKEWYFFSPRDRKYPNG

SRTNRATKAGYWKATGKDRKVNSQ--RAVGMKKTLVYYRGRAPHGSRTDWVMHEYRLDETGLQ-DAYALCRV

FKKTAPGPRSPDDESDQ-FPDTEVSQPDDFPNA-V

>Os03t0133000-01

MAEDLPGFRFHPTEEELLDFYLSRVVLGKKLHNIIGTLNIYRHDPWDLPGMAKIGEREWYFFVPRDRKAGNG

GRPNRTTERGFWKATGSDRAIRSSPKRVIGLKKTLVFYQGRAPRGTKTDWVMNEYRL--P---ED--VLCKI

YRKATPLKAS---SNP----DV--------LPADW

>Os03t0327100-01

MSDLPPGFRFHPTDEEIVSHYLTPKALNR-FSGVIGDVDLNKCEPWHLPAMAKMGEKEWYFFCHKDRKYPTG

TRTNRATESGYWKATGKDKEIFRG--ILVGMKKTLVFYLGRAPRGEKTGWVMHEFRLE-PSAK-DQWAVCKV

FNKEL-ALELPDPSSERSLP--DLGQTSALLSTDW

>Os03t0327800-01

M--LPPGFRFHPTDEELIVHYLRNRAASSPCPSIIADVDIYKFDPWDLPSKENYGDREWYFFSPRDRKYPNG

IRPNRAAGSGYWKATGTDKPIHSSTNESVGVKKALVFYKGRPPKGTKTNWIMHEYRLAAASMRLDDWVLCRI

YKKSSHAS-VP-EAMPD-MVLD-D------AAA-L

>Os03t0624600-01

--GLPPGFRFHPTDEELVTYYLARKVSDG-FARAIADVDLNKCEPWDLPSKASMGEKEWYFFSMRDRKYPTG

IRTNRATDSGYWKTTGKDKEIFHG--ALAGMKKTLVFYRGRAPKGAKTSWVMHEYRLQ--PAK-DEWVVCRV

FKKLQCHLSPPDA-S--FAPGGNQTQAGLVCSQDW

>Os03t0777000-01

-QDVLPGFRFHPTDEELVTFYLRRKVARKSLSEIIKEMDIYKHDPWDLPNASTVGEKEWYFFCLRGRKYRNS

IRPNRVTGSGFWKATGIDRPIYSASGESIGLKKSLVYYRGSAGKGTKTDWMMHEFRL--PCMQAEVWTICRI

FKRSITYR---QMSGP--KPPA--EEIARMVADDC

>Os03t0815100-01

M-NLPPGFRFHPTDDELVEHYLCRKAAGQRLPPIIAEVDLYKFDPWDLPERALFGAREWYFFTPRDRKYPNG

SRPNRAAGNGYWKATGADKPVAPR-GRTLGIKKALVFYAGKAPRGVKTDWIMHEYRLADASLRLDDWVLCRL

YNKKNEWE---EESSPESFPAPESS-YDDILDMDF

>Os04t0437000-01

--NLPPGFHFFPSDEELIIHFLRRKASL----DIVPTLILNLYDPWELNGKA-LG--QWYFFSHAT----Q-

---TRTSPNGHWKPI-ADETVISG-N--VGLKKTLIFFIGEPFEAIKTNWVMHEYHLMDSSHK-ENWVICRV

FESSYDSQ--------E-------DDYDEV-----

>Os04t0460600-02

--DLPPGFRFHPTDEELITHYLAKKVADR-FALAVAEADLNKCEPWDLPSLAKMGEKEWYFFCLKDRKYPTG

LRTNRATESGYWKATGKDKDIFRR--ALVGMKKTLVFYTGRAPKGEKSGWVMHEYRLH-ASSK-NEWVLCRV

FKKS---LVLPDMST--FNPGGDMGERERLLTSEW

>Os04t0475400-00

MAVLPWGYRFRPSDRQIIANYL-PMIHG--LP--VVGVDVFATRPAAIPHV-F-D--ERYFFGDQPREVP-G

---------GAWLPCGGDKAY-SGGGEAVAYRRKYEFREARAEEAAAP-WRMKEYRLNKP-----DCVVREI

YTK-----TPPDEMS-EDQP--DEGDYSDE-DG--

>Os04t0508400-00

MRELPPPSRFDPTAEDIVNRYLRRQV----YPQVVQRADVFDAHPAVIP--A--ADREWFFAAVRPRGHAGG

ARPRKA-GPGYVPRECR----------VVGMGCRLVFY--RARRASRTEWWMDEYRFGPP---EDELVVYKV

YRR--------------------------------

>Os04t0515900-01

--TLPPGFRFYPSDEELVCHYLYKKVSN-RASGTLVEVDLHAREPWELPDVAKLTASEWYFFSFRDRKYATG

SRTNRATKTGYWKATGKDREVRSPTRAVVGMRKTLVFYQGRAPNGVKSGWVMHEFRLD-P----EDWVLCRV

FQKSK---PAA---S--A-P--DHNSSPMMF--GW

>Os04t0536500-01

MDHVPPGFRFHPTDEELVDYYLRKKVALKKIDDVIKDIDLYKIEPWDLQEQCKIGEEEWYFFSHKDKKYPTG

TRTNRATTAGFWKATGRDKPIYV-C--LVGMRKTLVFYRGRAPNGQKSDWIMHEYRLETA------------

--------AC-------MLP---------------

>Os04t0691300-00

MECVAPGFRFHPTEEELVGYYLARKVVGQQDDGIIQEVDLNSIEPWDLLQAQQ--DQYCYFFSYKDRKYPTG

TRTNRATAAGFWKATGRDKPVLSSSPAVIGMRKTLVFYRGRAPNGCKTDWIIHEYRLVAP--DGSCWVVCRA

FHKPTTTT--------QNIPPT--TTAPAA---D-

>Os05t0194500-01

M---PAGVRFDPTDQELIE-HLEAKVGGTSHPEFIHTIQICYTHPENLP-----GTRLKHFFHR--KAYPTG

TRKRRKADAARWHKTGKTREIT--G--QPGCKKILVLYKKR--KAEKTSWVMHQYHLGE----DDELILSKV

FRS-AAAP---AAEV--KVT--SP---EHL-VGA-

>Os05t0415400-01

MAVLPTGFRFHPTDEELVINYLQRRATGLSCPPIIADVEIYNFNPWELPSMALFGEHEWYFFTLRDHRYPNS

VRPSRSAASGFWKATGTDKPVQVA-STPVAMKKALVFYVGRPPMETKTTWIMHEYRLTNASVKLDEWVLCKI

FNKSPEPDCSP----PD-FTEG--SSLNGTDTSD-

>Os05t0418800-00

MSDVMPGFRFHPTEEELIEFYLRRKVEGRRFNELITFLDLYRFDPWELPAMAVIGEKEWFFYVPRDRKYRNG

DRPNRVTASGYWKATGADRMIR--NSRPIGLKKTLVFYSGKAPKGVRSSWIMNEYRL--PLFYSE--SLCRV

YKRS----ARPRPPAP--PPAA--DELSTLFPLDW

>Os05t0426200-02

MA-LPPGFRFHPTDVELVSYYLKRKIMGKKPLQAISDVELYKFAPWDLPQSCL--SREWFFFCPRDKKYPNG

SRTNRSTPNGYWKTSGKDRTIELN--RIVGSKKTLIFHEGKAPKGNRTDWVMYEYKMEDAGFSKDDFVLCKI

FKKSGLGPLLPDDRSPRDLYGV--VNMDDI---DL

>Os05t0442700-01

MG-LPPGYRFYPTEEELVCFYLRHKLDGRRVPRVIPVADVCSLDPWQLPEAH--QTGEWFYFCPRQEREARG

GRPSRTTPSGYWKAAGTPGWVYSS-GRPIGTKKTMVFYRGRAPAGAKTKWKMNEYRAF-PQTR--DFSLCRL

YTRSGCPR-PP---S--AAPAADDGSMLQQ---DW

>Os05t0563000-01

SSQLPAGVKFDPTDQELLE-HLEGKADSKLHPEFIPTIEICYTHPERLP-----GSKLRHFFHR--KAYTTG

TRKRRKSDGGRWHKTGKTRPVM--G--RPGYKKILVLYKQR--KPEKTNWVMHQYHLGS----EEELVVSKV

FRQ-GSAA---AVN------ANDP---AAT--GAA

>Os06t0101800-01

--ALPVGFRFRPTDEELVRHYLKGKIAGRSHPLLIPDVDLSTCEPWDLPAMS-I-SDEWFFFAPRDRKYPGG

HRSNRSTAAGYWKATGKDRLIRS-AGPLIGIKKTLVFHRGRAPRGLRTAWIMHEYRTTEPHFQKNSFVLYRL

FNKHEQDDLTEDATTPA---------------PD-

>Os06t0104200-01

MSQVPPGFRFHPTDEELVDYYLRKKVAARRIDNVIKDVDLYKIEPWDLQERCRINEEEWYFFSHKDKKYPTG

TRTNRATAAGFWKATGRDKPIYATL--LVGMRKTLVYYRGRAPNGHKSDWIMHEYRLETP--QEEGWVVCRV

FKKRLP--PCGDEA-HTTMPSTDHRVLDKFTNKQW

>Os06t0131700-01

MV-VPPGFRFHPTEEELLTYYLKKKVASERIDDVIRDVDLNKLEPWDIQERCRIGGPDWYFFSHKDKKYPTG

TRTNRATAAGFWKATGRDKAIYSSN--RIGMRKTLVFYKGRAPHGQKSDWIMHEYRLDDP-AQEEGWVICRV

FKKKNLVHNCSDHK-AHKLPVAADDTLDRL-VADW

>Os06t0344900-00

--ELPPGFRFHPTDEELITYYLLRKVVDS-FNRAIAEIDLNKCEPWELPEKAKMGEKEWYFYSLRDRKYPTG

LRTNRATGAGYWKATGKDREIRSATGALVGMKKTLVFYRGRAPKGQKTQWVMHEYRLD--STR-DEWVIARI

FTKP--GVLRAEALN-SYNP-------PSLMQADW

>Os06t0530400-01

--SVPPGFRFHPTDEELLYYYLRKKVAYEAIDDVIREIDLNKLEPWDLKDRCRIGGPEWYFFSHKDKKYPTG

TRTNRATTAGFWKATGRDKAIFL-C--RIGMRKTLVFYVGRAPHGKKTDWIMHEYRLDQV--QVYIYIYIYI

YLTSA----------II-----HE-----------

>Os06t0560300-00

AG-LPAGVKFDPTDQELIE-HLEAKV---AHPEFIPTIEICYTHPEKLP-----GSKLRHFFHR--KAYTTG

TRKRRKPPRSRWHKTGKTRAVV--G--RQGCKKILVLYKHR--KPEKTNWVMHQYHLGE----EEELVVSKI

FRQ-ATAS---AAP-------------------P-

>Os06t0675600-01

MNRLPPGFRFHPRDDELVLDYLERKLLDTIY-PVMVDVDLNKCEPWDLPEIACVGGKEWYFYSLRDRKYATG

QRTNRATESGYWKATGKDRPISRK--LLVGMRKTLVFYKGRAPKGKKTEWVMHEFRKE--PLK-EDWVLCRV

FYKSRTTISLPD-NN-Q-----EQVKREALFTQ-W

>Os06t0726300-01

MEEQLPGFRFHPTEEELLEFYLKQVVQGKKLKDIIPTVHLYRHDPRELPGLARIGEREWYFFVPRDRKQATG

GRPSRTTERGFWKATGSDRAIRCAPKRLIGLKKTLVYYEGRAPRGTKTDWVMNEYRL--PQMQDD--VLCKV

YRKAVSLKST---S-P----SS--DEASAAHELEW

>Os07t0225300-01

MANLPPGFRFHPTDEELVAHYLCPRAAGRAAPPIIAELDLYRHDPWDLPHRALFGRREWYFFTPRDRKYPNG

SRPNRAAASGYWKATGADKPVLHN-GRTAGIKKALVFYHGKPPRGVKTEWIMHEYRLAKAALRLDDWVLCRL

YNKKNEWE---EE-AAESLPAAEAS-LDDL--LDL

>Os07t0272700-00

MA-LPPGLRFDPTDGELVSRFLLRRLQG-PLN-VILEADPLSVPPWKL---AE--GRDGFFFAEARA---NG

SRQKRTVGSGLWQGQRVGEKLL-PG----GRKYLLSF-AGR-----SSGWVMHEYAVTSP-----PIRLYRV

RKKKREGRPQPDADAPGEFP----HDSSAVAPAD-

>Os07t0456900-01

MA-LPPGVRFDPADDELVSRYLLRRLRK-PLH-VIHEADPLGAPPWML---AA--GRDAFFFAEARA---NG

KRQKRTVGGGFWQGQRVGERLR-PG----GRKYMLSF-AGR-----SSGWVMHEYAITAP-----PIRLYRV

RKKKREGGMMPDA-TPGDFPGSDS---DPVAPAD-

>Os07t0566500-01

--ELPPGFRFHPTDEELVVHYLKKKAASVPLPTIIAEVDLYKFDPWDLPEKANFGEQEWYFFSPRDRKYPNG

ARPNRAATSGYWKATGTDKPIMSSTREKVGVKKALVFYRGKPPKGVKTNWIMHEYRLTDASLRLDDWVLCRI

YKKTNKAG---DAAM-GTGALA-SSRSSR-LSSGM

>Os07t0684800-01

ESELPPGFRFHPTDEEVVTHYLTRKAQDS-FSVVIADVNLNNCEPWDLPSKAKMGEKEWFFFCHKDRKYPTG

MRTNRATASGYWKATGKDKEIFRG--LLVGMKKTLVFYMGRAPRGEKTPWVMHEYRLD-PSAK-EEWAVCRV

FNKD---LDLPDDPSPNSLPSSDQ--------SSW

>Os08t0103900-01

MDCVPPGFRFHPTEEELVGYYLARKVASQKIDDIIQELDLYRIEPWDLQERCKYGQTEWYFFSYKDRKYPSG

TRTNRATAAGFWKATGRDKPVLSSR--VIGMRKTLVFYKGRAPNGRKTDWIIHEYRLQSPT-QEEGWVVCRA

FQKPMPNQAVPDNS-S-DMMPTVDNLLTSL---S-

>Os08t0115800-01

MC-VPPGFRFHPTEEELLNYYLRKKVASEQIDDVIRDVDLNKLEPWDIQERCKIGGPDWYFFSHKDKKYPTG

TRTNRATAAGFWKATGRDKAIYNAH--RIGMRKTLVFYKGRAPHGQKSDWIMHEYRLDDPGGQEDGWVVCRV

FKKKHHHK---AGK-AQ--PCS-------------

>Os08t0157900-01

MTKLPLGFRFHPTDEELVRHYLKGKITGQIRSDVIPEIDVCKCEPWDLPDKS-I-SDEWFFFAPKDRKYPNG

SRSNRATEAGYWKATGKDRVIRSKKQQVIGMKKTLVFHRGRAPKGERTGWIMHEYRTTEPEFEEQGYVLYRL

FRKQEE-KAAPNENGPELLPMDEDDDAESTLDADW

>Os08t0200600-01

MSRMPPGFRFHPRDDELVLDYLLHKLAA-VYGVAIVDVDLNKCEPWDLPDAACVGGKEWYFFSLRDRKYATG

HRTNRATRSGYWKATGKDRSITRRAAAAVGMRKTLVFYRGRAPKGRKTEWVMHEFRLE--HLK-EDWVLCRV

FYKTRQTISLPD-AM-L-----EKAKLELSLSQ-W

>Os08t0433500-01

ME-LPAGFRFFPTDEELVTYYLRKAMDAFT--AAIRDVDLYTSDPWHLPSSA--AGGGCYFFCRRSSKYPSG

ARVRRATAGGYWKSTGKDKGVYAAGGGLVGTKKTLVFYEGRAPRGEKTSWVMHEYSRAP-IIY--EWVICRV

---------PP---T--RLPAAEDSSSDDL---DF

>Os08t0436700-01

MEELLPGFRFHPTDEELVSFYLKRKIQQKPISELIRQLDIYKFDPWDLPKLASTGEKEWYFYCPRDRKYRNS

VRPNRVTTAGFWKATGTDRPIYS-GTKCIGLKKSLVFYKGRAARGIKTDWMMHEFRL--PK-KIDSWTICRI

FKKTSSMA-TAQQHSP-QFG--SQ---ARILGA-W

>Os08t0511200-00

MHGLPPGFRFHPTDEELVTFYLAAKVFNACCGVDIAEVDLNRCEPWELPEAARMGEKEWYFFSLRDRKYPTG

LRTNRATGAGYWKATGKDREVVAAGGALIGMKKTLVFYKGRAPRGEKTKWVLHEYRLD--STK-EEWVICRI

FHKVGDQYFHPQHSSPSAFP----GPPAQL----W

>Os08t0535800-01

ME-LPPGFRFHPTDEELVVQYLRRKAFGLPLPAVIPLHNLFKLDPWDIP-----GSSDKYFFAVRP---PAA

RRQHVTASGGCWKAGGRDKPVVVAGSHLVGVKKGMVFVQGRKAAAAAAAWVMHEYSLALPMHKAEEWVVCRI

F-------RSPDV--P-RSPSS--SSQSSCVSS--

>Os08t0562200-02

MSSLAPGFRFHPTDEELVSYYLKRKVHGRPLKDAIAEVDLYKVEPWDLPARSRLRSRQWYFFSRLDRKHANR

ARTNRATAGGYWKTTGKDREVRNG-PTTVGMKKTLVFHAGRAPKGERTNWVMHEYRL---TIPQDSFVVCRI

FQKAGPGPLLPDNN-PGFQPMGEEDMVDDGAPSKF

>Os09t0295000-01

MA-------FHPTEQELISSYLHPRVAAGGVPSFIHHADAYAADPADLP-RA--G--RWYFFSPV-RTTERG

TRRARAVESGCWHESG-VRAVVDAAGRRVGHRQFFSFVKE--EDGKRTGWLMVELGVDN-DAASSELVLCKI

YRMP-P-SRAPLMGTRSGAPGGST-VEDEQTTTR-

>Os09t0493700-01

---WPPGFRFSPTDEELVLYFLKRRIATGR-PPYIADVDVYKSHPSHLPERSALGDKQWFFFSRMDRKYPNG

SRASRTTGEGYWKATGKDRSICNGSGRAVGSKKTLVYHHGRAPRGERSDWVMHEYTLLAP-ARREAYALYKL

FHKSGAGPALPNDD-PQSVPVGEEAT----VPS-W

>Os09t0497900-01

MESLPPGFRFHPTDEELITYYLRQKIADG-FTRAIAEVDLNKCEPWDLPEKAKMGEKEWYFFSLRDRKYPTG

VRTNRATNAGYWKTTGKDKEIFTGTPELVGMKKTLVFYKGRAPRGEKTNWVMHEYRLH--SNK-DEWVVCRI

FAKTA-GVLLPQQDPPHINPGSN-TTNQVMLANEW

>Os09t0509100-01

---LPPGFRFHPTDEELVVQYLRRRALCRPLPAVIPVHDATVLDPWDLP-----G-GEAYFFSFRQ-AAASG

GWRRRRAGSGYWKATGAEKPVFLRGQHLVGVKTTLLFLRAKP--PSRTHWVMHEYRLAAATKRAQEWVVCRI

FLKNN---ETPGH--P--SP----SS-SSCVSS--

>Os09t0552800-00

MEGLPPGFRFHPTDEELVNYYLKRKIHGLKIEDIIPEVDLYKCEPWELAEKSFLPSREWYFFGPRDRKYPNG

FRTNRATRAGYWKSTGKDRRVVHQGGRAIGMKKTLVYYRGRAPQGVRTDWVMHEYRLDDDPIK-DTYALCRV

FKKNAICTGACEQESDQ------------------

>Os10t0359500-01

MS-VPLALNTFWSDEELVRF-LERK-EAHSLPNVVG--NISLIDPRN----------DWYMNFSDDQSPKNG

EAIIKS-KTGYWKPTST----------VIGMKVSLDHYEGEAPSGKRTGWVMDEYLIEQ-EANQDYKNLCTI

FDKNANVPHAPDSAAA-SPPATDESHVKRSSSS-W

>Os10t0413700-01

MAR---GFRFVPRDQELLDI-LDDKLRGAPLDAVFHDTRILDFHPAKL-MYAE-E--GIYFFSTI-EKAAKQ

KKWPRRAAQGRWKVLG-SSQMVEVGGVPVGRKLSMEFYK-------RTNWGMHEFVRII-GPN--DLAVYRL

HGD-AA-D--A---APKAAT--ST---DD------

>Os10t0413900-01

MA----GVRFLPKDLELLAI-LDAKLRGSPLGAIFHDTQILDFHPYKLPMYAE-E--GIYFFSTM-Q----C

RKIVERAAQGRWKNNC---ETLEVGGVAVGRKFTMNFYHG--GDNDWTNWGMQEFARII-GPN--DLALYRL

YEDAAA-S-PPDQNA--ATP----------APV--

>Os10t0414000-01

MA---IGFYFAPTDQELLAI-LEAKRLGRPLSAFFHDIRILDFHPAEL-KYAK-E--GFYFFSKR-EP---S

KKRPLRVAEGAWNSGAVVKSSKSGGGYDVGHKKTLVFHRP--GDKEKTNWAIQEFTRII-GPQ--DLAVYRL

YAD-AA-D--PDKNAPKAAPGPST-MADERAAA--

>Os10t0477600-01

--TLPPGFRFCPSDEELICFYLRNKVAN-RVAGTLVDVDLHAREPWELPEVAKLTAEEWYFFSFRDRKYATG

SRTNRATKTGYWKATGKDRIVHEGTRAVVGMRKTLVFYLGRAPNGQKTTWVMHEFRLE-P----EDWVLCRV

FDKKKPSTPDPD--S--HCS--EHVGAHFGF--GG

>Os10t0532000-01

MVCVPPGFRFHPTDEELVGYYLRKKVASQKIDDVIRDVDLYRIEPWDLQEHCRIGEEEWYFFSYKDRKYPTG

TRTNRATMTGFWKATGRDKAVRE-R--LIGMRKTLVFYKGRAPNGHKTDWIVHEYRLESP--QEEGWVVCRA

FKKRTMQ-ASY------ELPP-EDRAFDKFQHTTP

>Os10t0571600-01

------------------------------------------------------------FFCPRDRKYPNG

SRTNRATSTGYWKATGKDRKIACA--EVFGLRKTLVFYKGRAPGGERTDWVMHEYRLCQGNFI-GAYALCRV

IKRHEAGLSSSHQSSQP-----QA-AADDN-----

>Os11t0126900-01

--ALPPGFRFHPTDEELIVHYLMNQAASVKCPPIIAEVNIYKCNPWDLPGKALFGENEWYFFSPRDRKYPNG

ARPNRAAGSGYWKATGTDKSILST-SDNIGVKKALVFYKGKPPKGVKTDWIMHEYRLTG-TMR---------

-----------------------------------

>Os11t0127000-01

-I-------------------------------------------------ARMGNNEWY-FSRKDMKYPTG

MRTNRATKEGYWKATGKDREIFK-NKQLVGMKKTLVFYMGRAPKGTRTNWVMHEFRPH-PNP--NEWVVCKV

FHKKQ-GDQLDDPSSP-NTPGI--LLPSSNVSQEY

>Os11t0127600-01

MVFLPPGFRFHPTDAEVILSYLLQKFLNS-FTLPIGEVDLNKCEPWDLPSKAKMGEKEWYFFSHKDMKYPTG

MRTNRATKEGYWKATGKDREIFNLNKQLVGMKKTLVFYMGRAPKGTKTNWVMHEFRLH-PNLK-DEWVVCKV

FHKKG-DDQLDDPSAPSNMPGI-----------D-

>Os11t0154500-01

IS-ATPGFRFYPTEEELLGFYLRHRLAGRP--RVIPVVDVYGYHPSQLAALA--GAREWFFFCPRAERELHG

GRPARTTPSGYWKATGSPSCVISSTNRVIGVKRTMVFYQGRAPTGTKTRWKMNEYKAVAPRLR--ELGVCRV

YISTGTLR--P---N--VMPAASSGSEDDA----W

>Os11t0184900-02

MEQLPPGFRFHPTDDELVMYYLCRKCGGLPLAPVIAEVDLYKFNPWDLPERAMGGEKEWYFFSPRDRKYPNG

QRPNRAAGTGYWKATGADKPVGS--PRAVAIKKALVFYAGKPPKGVKTNWIMHEYRLADAALRLDDWVLCRI

YNKKGVIEVKPAAKAPESMPTDSPP-GVDWIDSDF

>Os11t0512000-01

MAHLLPGFRFRPTDDELVIKYLYPRAFHVPLPAIITDVDIHHHNPWDI-----VAERGKHFFTRKEVKYPGS

RRSNRVAGNGFWRAAGSEVPIYYKADMLVGMRRTLVFHYGKSRSAERTEWAMHEFQLAGPRAKVESWVICCI

YKKRQRAPVIPDAE--DSSP--DEEDKDGD---N-

>Os11t0512100-01

MARL-------PTNADLVVHYLHRRAIQEPVPDFITNVDILQHNPWDI-----VEEKGKYFFIHEENERLGN

HHSNRAAGDGFWRPVGSEVPIYHKGEALVGMKRTLVFHYGNSSSAKRTEWVMQEFRLAGP-AGVESLRICRI

YKKRQRTPIIPNTE-PD-----DVDDKDGY-----

>Os11t0512200-00

MAHLSVGCVFRPTEGELVVNYLYRRAMQEPLPDFITDVDIQCHNPWEI-----VGEKGKHFFTRKENSHPRD

YESNHAAGDGFWRLAGTEVPIYNKGEKLVGMKRTLVFHFRKSSSTERTGWVMPTCRCQ-P------------

---------------------------DEA-----

>Os11t0512600-00

--------------------------------------------------------RGKYFFMQKEIKCPSS

RRSNRITSKGFWRSAGSEKPVYYNGCMLVGMRRTLTFYFGNSRTAERTKWGMQEFRLAGPRNVVESWLICRI

YRTRQRALITPNAE--DSSP--DEDQKD-------

>Os12t0123700-01

--ALPPGFRFHPTDEELIVHYLMNQAASIKCPPIIAEVNIYKCNPWDLPGKALFGENEWYFFSPRDRKYPNG

ARPNRAAGSGYWKATGTDKSILST-SDNIGVKKALVFYKGKPPKGVKTDWIMHEYRLTG-TMRLDDWVLCRI

HKKSN------DID-CD---DA--RR-KRMSSSG-

>Os12t0123800-01

MVFLPPGFRFHPTDAEVILSYLLQKLLNS-FTLPIGEVDLNKCEPWDLPSKAKMGEKEWYFFSHKDMKYPTG

MRTNRATKEGYWKATGKDREIFR-KKQLVGMKKTLVFYMGRAPKGTKTNWVMHEFRLH-PNPK-DEWVVCKV

FHKKQ-GDQLDDPSSPSITPGI--LLPSSNLTQEY

>Os12t0135850-00

MES----FRFNPSAEDLITFYLPRLIAGKPMKKFICRADVYGSEPSDLP-RC--G---RFFFTSC-KHKGSS

TKKERTAGAGTWVQNSK--EVKNKAGVKVGETQNFRFKDG--SY---TDWLMEEHHCC--RQQGDEPVICRM

YRASAA-R-QP----P-----------------D-

>Os12t0137000-00

MA--PSGY-FKPE--QLIRDYL-NHITGRPIEDIVREADVYGSDPATLAHRA--G--KWYFLTV---KWKRG

TRLNRCVG-GTWHSQ--RRVIE-------GYGDRQAFY--RAPGNKKTNWLMEEANLPA----TDIMVICKV

YRKATA--VVPDAAT-AFSPGVDPSH-GHI-----

>Os12t0156100-01

VS-ATPGFRFYPTEEELIGFYLRHRLAGRA-DRVIPVVDVYGYHPSQLAAMA--GAGEWFFFCPRAERELHG

GRPARTTPSGYWKATGSPSFVFSSAARVIGVKRTMVFYQGRAPSGTKTRWKMNEYKAVAPRLR--ELSVCRV

YVSTGTLR--P---H--QLPAAHD-SEDAA---DW

>Os12t0477400-01

ME-LPPGFRFRPTDEELVVHYLRRRALGSPLPPAVDDVRLLAHDPSDLP-----GEQERYFFTCKEAKYVKG

RRANRATGAGYWKATGKEKPVAVSAAAVVGMKRSLVFYRGKPPTGKKTDWVMHEYRLAGPPARAEGWVLCRV

FRKKGSAAASPDDT--DASPSSEHETTSRN---D-

>Os12t0610600-01

MSELPPGFRFHPRDDELICDYLAPKVAG----PPMVDVDLNKVEPWDLPEVASVGGKEWYFFSLRDRKYATG

QRTNRATVSGYWKATGKDRVVARR--ALVGMRKTLVFYQGRAPKGRKTEWVMHEYRME--SSK-EDWVLCRV

ICKRKSGGPLPD-TA-SYLPNNDKVLPGLPVSS-W

>Os12t0630800-00

MEDVFPGFRFHPTDQELVGFYLTRKVEKKPFSDIIKEIDIYKHDPWDLPSRG--GEG---FLLPSREQHPEG

HRHRQA----------------------------LLFLRRRR--RRRCRW-PQESRLL--------------

-------------------P---------------

>AT5G39610.1

MDDLPPGFRFHPTDEELITHYLKPKVFNF-FSTAIGEVDLNKIEPWDLPWKAKMGEKEWYFFCVRDRKYPTG

LRTNRATEAGYWKATGKDKEIFKG--SLVGMKKTLVFYKGRAPKGVKTNWVMHEYRLE-PTAK-NEWVICRV

FQKR----GLPDCSTD-SDP--DG------F--DW

>AT5G61430.1

MEDLPPGFRFHPTDEELITHYLHKKVLDS-FSKAIGEVDLNKSEPWELPWMAKMGEKEWYFFCVRDRKYPTG

LRTNRATEAGYWKATGKDKEIYRG--SLVGMKKTLVFYRGRAPKGQKTNWVMHEYRLE-PTAK-NEWVICRV

FQKS----LLPDSSPP-RIPSMENQSLKTMVSTEW

>AT5G53950.1

--YLPPGFRFHPTDEELITHYLLRKVLDC-FSRAIAEVDLNKCEPWQLPGRAKMGEKEWYFFSLRDRKYPTG

LRTNRATEAGYWKATGKDREIFSSTCALVGMKKTLVFYKGRAPKGEKSNWVMHEYRLE--SSK-DEWVISRV

FQKT--TLSLPDPTSPN-LP----STQSNFASTDW

>AT5G18270.1

VVDLPPGFRFHPTDEEIITCYLKEKVLNR-FTVAMGEADLNKCEPWDLPKRAKMGEKEFYFFCQRDRKYPTG

MRTNRATESGYWKATGKDKEIFKG--CLVGMKKTLVFYRGRAPKGEKTNWVMHEYRLE-PSAR-DEWVVCRV

FHKN----SLPDPS-PQ----QNNMEQSLVLSSGW

>AT3G18400.1

--NLPPGFRFHPTDEELITHYLCRKVSDG-FTKAVVDVDLNKCEPWDLPAKASMGEKEWYFFSQRDRKYPTG

LRTNRATEAGYWKTTGKDKEIYRS--VLVGMKKTLVFYKGRAPKGEKSNWVMHEYRLE--TNK-EEWVVCRV

FEKST-AAELPSNSEPQMIPSS--SSSSQV---DW

>AT5G07680.1

MEDLPPGFRFHPTDEELITHYLHKKVLDG-FSKAIGEVDLNKAEPWELPYKAKIGEKEWYFFCVRDRKYPTG

LRTNRATQAGYWKATGKDKEIFRG--SLVGMKKTLVFYRGRAPKGQKTNWVMHEYRLD-PTAK-NEWVICRV

FHKT----SLPDSSPP-MIPSEENQNFKTLVS-EW

>AT3G29035.1

MDDLPPGFRFHPTDEELITHYLRPKVVNF-FSIAIGEVDLNKVEPWDLPWKAKLGEKEWYFFCVRDRKYPTG

LRTNRATKAGYWKATGKDKEIFKG--SLVGMKKTLVFYKGRAPKGVKTNWVMHEYRLE-STAK-NECVISRV

FHTR----GLPDSSTP-FDP--DN------FKKDW

>AT3G15170.1

--LMPPGFRFHPTDEELITYYLLKKVLDN-FSAAISQVDLNKSEPWELPEKAKMGEKEWYFFTLRDRKYPTG

LRTNRATEAGYWKATGKDREIKSSTKSLLGMKKTLVFYKGRAPKGEKSCWVMHEYRLD--SAK-DEWVLCKV

CLKS--GV------S-SFLP----------SSGPW

>AT2G24430.1

MEALPPGFRFHPTDEELISYYLVNKIADN-FTKAIADVDLNKSEPWELPEKAKMGGKEWYFFSLRDRKYPTG

VRTNRATNTGYWKTTGKDKEIFNSTSELVGMKKTLVFYRGRAPRGEKTCWVMHEYRLH--TSKQDEWVVCRV

FKKTE-ATLPPPH-STQ---GGN----------SW

>AT1G54330.1

--SLPPGFRFHPTDEELVAYYLDRKVNGQAIEEIIPEVDLYKCEPWDLPEKSFLPGNEWYFYSTRDKKYPNG

SRTNRATRAGYWKATGKDRTVESK--MKMGMKKTLVYYRGRAPHGLRTNWVMHEYRLTH-SLK-ESYALCRV

FKKNIQIPEEEESKSDN-FP--QQSA---------

>AT5G17260.1

--SLPPGFRFHPTDEELITYYLKRKINGQEIEEIIPEVDLYKCEPWDLPGKSLIPSKEWFFFSPRDRKYPNG

SRTNRATKGGYWKATGKDRRVSWR--RAIGTKKTLVYYRGRAPHGIRTGWVMHEYRLDEPGMQ-DAYALCRV

FKKIVIEA------TDQDFSNSDENQAQDNNSADA

>AT3G15510.1

MENLPPGFRFHPTDEELVVHYLKRKAASAPLPAIIAEVDLYKFDPWELPAKASFGEQEWYFFSPRDRKYPNG

ARPNRAATSGYWKATGTDKPVLASGNQKVGVKKALVFYSGKPPKGVKSDWIMHEYRLIEGSLRLDDWVLCRI

YKKNN--AKIP---MPDADVASEEG-----MSSD-

>AT3G03200.1

--SLPPGFRFHPTDEELITYYLKRKINGLEIEEVIAEVDLYKCEPWDLPGKSLLPSKEWYFFSPRDRKYPNG

SRTNRATKGGYWKATGKDRRVSWR--RAIGTKKTLVYYRGRAPHGIRTGWVMHEYRLDEPGMQ-DAYALCRV

FKKIVIEA-----CTEQDFFNNDENQIEDNDSSDT

>AT1G65910.1

--SMPPGFRFHPTDEELVIYYLKRKINGRTIEEIIPEIDLYKCEPWDLPGKSLLPSKEWFFFSPRDRKYPNG

SRTNRATKAGYWKATGKDRKVTSH--RMVGTKKTLVYYRGRAPHGSRTDWVMHEYRLEESGIQ-DAYALCRV

FKKSALANSTSENE-EQYFGDTQENEHNENSSSS-

>AT1G01720.1

MSQLPPGFRFHPTDEELVMHYLCRKCASQSIAPIIAEIDLYKYDPWELPGLALYGEKEWYFFSPRDRKYPNG

SRPNRSAGSGYWKATGADKPIGL--PKPVGIKKALVFYAGKAPKGEKTNWIMHEYRLADSSLRLDDWVLCRI

YNKKGATE-PPDEM-PESVPTDEF--WKDWLDFNF

>AT3G17730.1

--GLPPGFRFHPTDEELVNYYLKRKINGQEIEDIIPEVDLYKCEPWDLAEKSFLPSREWYFFGPRDRKYPNG

FRTNRATRGGYWKSTGKDRRVTSQ--RAIGMKKTLVYYKGRAPQGIRTDWVMHEYRLDDDSLQ-DSYALCRV

FKKNGICSASMNDESDQ------------------

>AT1G69490.1

METLPPGFRFHPTDEELIVYYLRNQTMSKPCPSIIPEVDIYKFDPWQLPEKTEFGENEWYFFSPRERKYPNG

VRPNRAAVSGYWKATGTDKAIHSG-SSNVGVKKALVFYKGRPPKGIKTDWIMHEYRLHD-SMRLDEWVLCRI

YKKRGASK---DEK-CH--------------SS--

>AT3G04060.1

VEDLPPGFRFHPTDEEIITHYLKEKVFNR-FTAAIGQADLNKNEPWDLPKIAKMGEKEFYFFCQRDRKYPTG

MRTNRATVSGYWKATGKDKEIFRG--CLVGMKKTLVFYTGRAPKGEKTNWVMHEYRLD-PTAR-DEWVVCRV

FHKN----SLPDPGPPQ---GSNNLEHSLVLSSDW

>AT3G04070.1

MISLPPGFRFHPTDEELILHYLRKKVSSSPVPSIIADVDIYKSDPWDLPAKAPFGEKEWYFFSPRDRKYPNG

ARPNRAAASGYWKATGTDKLIAVPGHENIGIKKALVFYRGKPPKGVKTNWIMHEYRLADSSMRLDDWVLCRI

YKKSHASL-SP---LSNETPSDTSGSKRE-INADL

>AT5G08790.1

--NLPAGFRFHPTDEELVKFYLCRKCASEQISPVIAEIDLYKFNPWELPEMSLYGEKEWYFFSPRDRKYPNG

SRPNRAAGTGYWKATGADKPIGK--PKTLGIKKALVFYAGKAPKGIKTNWIMHEYRLANANLRLDDWVLCRI

YNKKGTME-FP----SPTYPDDDV--LEDAMELDP

>AT1G77450.1

MKQFPPGFRFHPTDEELVLMYLCRKCASQPIPPIITELDLYRYDPWDLPDMALYGEKEWYFFSPRDRKYPNG

SRPNRAAGTGYWKATGADKPIGR--PKPVGIKKALVFYSGKPPNGEKTNWIMHEYRLADSSLRLDDWVLCRI

YNKKGVIE------D-DSVA--EF--LSN-LDFDF

>AT1G61110.1

MEHLPPGFRFHPTDEELVVHYLKKKADSVPLPSIIAEIDLYKFDPWELPSKASFGEHEWYFFSPRDRKYPNG

VRPNRAATSGYWKATGTDKPIFTC-SHKVGVKKALVFYGGKPPKGIKTDWIMHEYRLTDASLRLDDWVLCRI

YKK--------DLEM-G-GQEAS-HA----TTS--

>AT1G52880.1

MENLPPGFRFHPTDEELVIHYLKRKADSVPLPAIIADVDLYKFDPWELPAKASFGEQEWYFFSPRDRKYPNG

ARPNRAATSGYWKATGTDKPVISTGSKKVGVKKALVFYSGKPPKGVKSDWIMHEYRLTD-SLRLDDWVLCRI

YKKNNSTAHVP----PD---CF-NG-----MAA--

>AT5G63790.1

MDNLPAGFRFHPTDEELVKFYLCRRCASEPINPVIAEIDLYKFNPWELPEMALYGEKEWYFFSHRDRKYPNG

SRPNRAAGTGYWKATGADKPIGK--PKTLGIKKALVFYAGKAPKGIKTNWIMHEYRLANANLRLDDWVLCRI

YNKKGTME-LP----PK-----DV--LEDAIGLDF

>AT1G76420.1

VEGLPPGFRFHPTDEELITFYLASKIFHG-LSIHISEVDLNRCEPWELPEMAKMGEREWYFYSLRDRKYPTG

LRTNRATTAGYWKATGKDKEVFSG-GQLVGMKKTLVFYKGRAPRGLKTKWVMHEYRLE--TCK-EEWVICRV

FNKT--GDALPEPSNPSIFPEDDFTEQSSNFSSDL

>AT5G04410.1

--TLAPGFRFHPTDEELVRYYLKRKVCNKPFKDAISVTDIYKSEPWDLPDKS-L-SREWYFFSMLDKKYSNG

SKTNRATEKGYWKTTGKDREIRNG-R-VVGMKKTLVYHKGRAPRGERTNWVMHEYRLSD-DLKQEAYVLCRI

FQKSGTG-YVPNNESPSMDPLSNQAVEEKEIPTDW

>AT5G39820.1

MDDVLPGFRFHPTDEELVSFYLKRKVLHKSLPDLIKKVDIYKYDPWDLPKLAAMGEKEWYFYCPRDRKYRNS

TRPNRVTGGGFWKATGTDRPIYS-STRCIGLKKSLVFYRGRAAKGVKTDWMMHEFRL--PKKQLNAWAICRI

FKKTNAVS---DVSSS----ASNN------L---V

>AT1G12260.1

MNHVPPGFRFHPTDEELVDYYLRKKVASKRIEDFIKDIDLYKIEPWDLQELCKIGEEDWYFFSHKDKKYPTG

TRTNRATKAGFWKATGRDKAIYL-S--LIGMRKTLVFYKGRAPNGQKSDWIMHEYRLETT--QEEGWVVCRV

FKKRLA--SPSDQS-H-QLPPKQQRVLDKFAT-QW

>AT4G36160.1

MESVPPGFRFHPTDEELVGYYLRKKVASQKIDDVIRDIDLYRIEPWDLQESCRIGEEEWYFFSHKDKKYPTG

TRTNRATMAGFWKATGRDKAVYD-K--LIGMRKTLVFYKGRAPNGQKTDWIMHEYRLESP--QEEGWVVCRA

FKKKPMT-SS-DE--KVQLPPSEKRALDKF-GVDY

>AT1G33060.2

IKELPLGFRFRPTDEELINHYLRLKINGRDLERVIPEIDVCKWEPWDLPGLS-I-TDEWFFFCPRDRKYPSG

HRSNRATDIGYWKATGKDRTIKSK-M-IIGMKKTLVFYRGRAPRGERTNWIMHEYRATD-ELDQNPYVLCRL

FHK-----ALDNHERPDVLPVAEQRDLDSVEPAK-

>AT1G26870.1

MGDLLPGFRFHPTDEELVSFYLKRKVQHNPLSELIRQLDIYKYDPWDLPKFAMTGEKEWYFYCPRDRKYRNS

SRPNRVTGAGFWKATGTDRPIYS-GNKCIGLKKSLVFYKGRAAKGVKTDWMMHEFRL--PSKRFDSWAICRI

FKKTNTTT-KPENNSPNQETSNQEDHCDTL---DL

>AT4G35580.2

--ELPLGFRFRPTDEELVNHYLRLKINGRHSDRVIPDIDVCKWEPWDLPALS-I-TDEWFFFCPRDRKYPNG

HRSNRATDSGYWKATGKDRSIKSK-T-LIGMKKTLVFYRGRAPKGERTNWIMHEYRPTL-DLDQSPYVLCRL

FHK-----VEKGYH-PDVSPMAQQRQSTGLAAAGW

>AT5G46590.1

--CLPPGFRFHPTDEELIEYYLKRKVEGLEIEEVIPVIDLYSFDPWELPDKSFLPNREWYFFCSRDKKYPNG

FRTNRGTKAGYWKATGKDRKITSR-SSIIGYRKTLVFYKGRAPLGDRSNWIMHEYRLCDQNLK-GAFVLCRV

AMKNEIKTSRVETPNQQ-FP--NMTSLDQNTNY--

>AT2G18060.1

MESVPPGFRFHPTDEELVGYYLRKKIASQKIDDVIRDIDLYRIEPWDLQEQCRIGEEEWYFFSHKDKKYPTG

TRTNRATMAGFWKATGRDKAVYD-K--LIGMRKTLVFYKGRAPNGKKSDWIMHEYRLESP--QEEGWVVCRA

FKKR-AT-SS-DEA-QIQLPPSDNRELDKFNGTDF

>AT4G17980.1

--CLPPGFRFHPTDEELIGYYLSRKIEGLEIEEVIPVIDLYKFDPWELPGKSFLPNREWFFFCPRDKKYANG

SRTNRATKAGYWKATGKDRKITCK-SHVIGYRKTLVFYEGRAPLGDRTNWFMHEYRLCDQNFK-GAFALCRV

VKKNELKKSLAEAQSKE-FP--HVEMLVRS-----

>AT3G15500.1

MGSLPPGFRFYPTDEELMVEYLCRKAAGHDFSQLIAEIDLYKFDPWVLP-KALFGEKEWYFFSPRDRKYPNG

SRPNRVAGSGYWKATGTDKVISTE-GRRVGIKKALVFYIGKAPKGTKTNWIMHEYRLIEPSTKLDDWVLCRI

YKKQTSAQSGRG-S-QDALPKT--NSVPELTEGDF

>AT4G10350.1

MG-VPPGFRFHPTDEELLHYYLKKKISYQKFEEVIREVDLNKLEPWDLQERCKIGTPEWYFFSHKDRKYPTG

SRTNRATHAGFWKATGRDKCIRNS--KKIGMRKTLVFYKGRAPHGQKTDWIMHEYRLEDP---EDGWVVCRV

FMKKNLFKPYQNK----CEPGT--AMMDRLSSREW

>AT1G62700.1

MNQVPPGFRFHPTDEELVDYYLRKKVASKRIEDIIKDVDLYKIEPCDLQELCKIGEEEWYFFSHKDKKYPTG

TRTNRATKAGFWKATGRDKAIYI-S--LIGMRKTLVFYKGRAPNGQKSDWIMHEYRLETT--QEEGWVVCRV

FKKKLAA-SPSDQS-HQQLPP-QKRVLDKFAAAQ-

>AT1G33280.1

M--VPPGFRFHPTDEELLHYYLKKKISYEKFEEVIKEVDLNKIEPWDLQDRCKIGTPEWYFFSHKDRKYPTG

SRTNRATHSGFWKATGRDKCIRNS--KKIGMRKTLVFYKGRAPHGQKTDWIMHEYRIEDP---EDGWVVCRV

FKKKNLFKPYQNKE---CQPGT--G----ISSREL

>AT3G10500.1

--TLAPGFRFHPTDEELVRYYLKRKICNKPFKDAISVTDVYKSEPWDLPDKS-L-SREWYFFSMLDKKYRNG

SKTNRATEMGYWKTTGKDREILNG-K-VVGMKKTLVYHKGRAPRGERTNWVMHEYRLVD-DLDQDAFVLCRI

FQKSGSG-FVPNNESPSLDPLTDQVVEEKEIPADW

>AT1G52890.1

MGSLPPGFRFYPTDEELMVQYLCRKAAGYDFSQLIAEIDLYKFDPWVLP-KALFGEKEWYFFSPRDRKYPNG

SRPNRVAGSGYWKATGTDKIISTE-GQRVGIKKALVFYIGKAPKGTKTNWIMHEYRLIEPSTKLDDWVLCRI

YKKQSSAQNARGTS-HDRI-KT--NSVPELVESDF

>AT2G43000.1

--EPLPGFRFHPTDEELLGYYLRRKVENKTIKELIKQIDIYKYDPWDLPRVSSVGEKEWYFFCMRGRKYRNS

VRPNRVTGSGFWKATGIDKPVYS-NLDCVGLKKSLVYYLGSAGKGTKTDWMMHEFRL--PA-QAEVWTLCRI

FKRVTSQR---RT-SP---P-QN-------IGDEL

>AT2G02450.2

MSDVMPGFRFHPTEEELIEFYLRRKVEGKRFNELITFLDLYRYDPWELPAMAAIGEKEWYFYVPRDRKYRNG

DRPNRVTTSGYWKATGADRMIR--TSRPIGLKKTLVFYSGKAPKGTRTSWIMNEYRL--PKYQAE--SLCRV

YKRP-------NHSS-E---GS--DDLQRLLVADW

>AT1G32870.1

---LAPGFRFHPTDEELVVYYLKRKIRRKKLREAIGETDVYKFDPEELPEKALYRDRQWFFFSLRDRKH--G

SRSSRATERGYWKATGKDRVIHC-DSRPVGEKKTLVFHRGRAPNGERTNWVMHEYTLHKKGEDKDAYVLYKI

YKKSGSGP--PDQG-----PYNNNDSSEFLIPADW

>AT2G33480.1

ME-LPPGFRFHPTDEELVVQYLRRKVTGLPLPSVIPETDVCKSDPWDLP-----GESEMYFFSTREAKYPNG

NRSNRSTGSGYWKATGLDKQI--G-KLVVGMKKTLVFYKGKPPNGTRTNWVLHEYRLVD-SLYQNNWVLCRV

FLKKRSNSTCPDTK---LTPTSSSRQEISY----F

>AT3G10490.2

MGSLAPGFRFHPTDEELVSYYLKRKVLGQPVRDAIGEVDIYKHEPWDL---AVFSTREWYFYSALDKKYGNG

ARMNRATNRGYWKATGKDREIRRD-ILLLGMKKTLVFHSGRAPDGLRTNWVMHEYRLVE-NLVQDAYVLCRV

FHKNNIGPPPPDQD-PNEAPNNKENDLQKEMSAD-

>AT4G27410.3

MGSLPPGFRFYPTDEELLVQYLCRKVAGYHFSQVIGDIDLYKFDPWDLPGKALFGEKEWYFFSPRDRKYPNG

SRPNRVAGSGYWKATGTDKIITAD-GRRVGIKKALVFYAGKAPKGTKTNWIMHEYRLIEHSSKLDDWVLCRI

YKKTSGSQCREG-S-QDRM-----NPIPELAESSF

>AT5G62380.1

MEHIPPGYRFHPTDEELVDYYLKNKVAFPGMQDVIKDVDLYKIEPWDIQELCGRGGEEWYFFSHKDKKYPTG

TRTNRATGSGFWKATGRDKAIYS-E--LVGMRKTLVFYKGRAPNGQKSDWIMHEYRLETP--HEEGWVVCRA

FKKKLTTMSGQ----QVHLPPRHQQTLDKFEEVPW

>AT1G79580.1

SSSVPPGFRFHPTEEELLYYYLKKKVSYEPIDDVIREVDLNKLEPWELKEKCRIGGPEWYFFSHKDKKYPTG

TRTNRATAAGFWKATGRDKSIHLNK--KIGLRKTLVFYTGRAPHGQKTEWIMHEYRLDDI--QEDGWVVCRV

FKKKN---QDHDHH-PHHLPPDTDSFLDKLV---F

>AT3G10480.3

MGSLAPGFRFHPTDEELVSYYLKRKVLGKPVRDAIGEVDIYKHEPWDLEMFALFSTREWYFFSALDKKYGNG

ARMNRATNKGYWKATGKDREIRRD-IQLLGMKKTLVFHSGRAPDGLRTNWVMHEYRLVE-SLLQDAYVLCRV

FHKNNIGPALPNQDSPSEAPNNKENDLQKEMSAD-

>AT1G71930.1

--SMPPGFRFHPTEEELVGYYLDRKINSMKSADVIVEIDLYKMEPWDIQARCKLGEEEWYFFSHKDRKYPTG

TRTNRATAAGFWKATGRDKAVLS-S--VIGMRKTLVYYKGRAPNGRKSDWIMHEYRLQNP--QEEGWVVCRA

FRKPIPNQ---NYS-NQELPPSESRTLDTL---HF

>AT3G12910.1

MEAKLPGFRFHPTDEELVGYYLSKKVLLKKTSEIVSQIDIYKFDPWDLPRSRNT-EKESYFFCKRGRKYRNS

IRPNRVTGSGFWKATGIDKPVYS-NKAVIGLKKTLVYYLGSAGKGNKTDWMMHEFRL--PSLLAEVWTLCRI

FKRTVSSR---ESSSP--------------IHHDL

>AT5G66300.1

MMSIPPGFRFHPTDEELVGYYLKKKIASQRIDDVIREIDLYKIEPWDLQERCRIGEEEWYFFSHRDKKYPTG

TRTNRATVAGFWKATGRDKAVYL-K--LIGMRKTLVFYRGRAPNGQKSDWIIHEYSLESP--QEEGWVVCRA

FKKRTTI-NC-DDT---QLPPSDDRYLDKF---D-

>AT1G32510.1

MV-LPPGFRFYPTDEELVGYYLHRRNEGLEIEEIIPLMDLYKFDPWELPEKS-L-NREWFFFCHRDRKYQNG

SRINRATKSGYWKATGKDRKIVSSSSSSIGCRKTLVFYMGRAPFGGRTEWVMHEYRLFD-NFK--DFALCRV

IKRNEHTLSSPNSS-PPYHPNN--EVMSHH-----

>AT2G46770.1

MQ-VPPGFRFHPTEEELLQYYLRKKVNSIEIDDVIRDVDLNKLEPWDIQEMCKIGTPDWYFFSHKDKKYPTG

TRTNRATAAGFWKATGRDKIIYSNR--RIGMRKTLVFYKGRAPHGQKSDWIMHEYRLDDP--QDEGWVVCRI

FKKKNL-----DTK-TQKLPPNSNAALDRLITA-W

>AT1G32770.1

NK-VPPGFRFHPTEEELLHYYLRKKVNSQKIDDVIREVDLNKLEPWDIQEECRIGTPDWYFFSHKDKKYPTG

TRTNRATVAGFWKATGRDKIICSC--RRIGLRKTLVFYKGRAPHGQKSDWIMHEYRLDDPSYNEEGWVVCRV

FRKKNYQK---DDP--HQLPPKTEASLDRL---NW

>AT5G13180.1

MD-LPPGFRFHPTDEELVVQYLKRKVCSSPLPSIIPEFDVCRADPWDLP-----GEKERYFFSTREAKYPNG

NRSNRATGSGYWKATGIDKRVVTS-NQIVGLKKTLVFYKGKPPHGSRTDWIMHEYRLSS-SM-PTNWVLCRI

FLKKRAGNTKPE-----LLPDHSDSSCNSF-----

>AT1G34190.1

MA-FAPGFRFHPTDEELVMYYLKRKICRKRLRNVIGVVDVYKMDPEELPGQSMLGDRQWFYFTPRSRKYPNA

ARSNRGTENGYWKATGKDRVIEY-NSRSVGLKKTLVFYRGRAPSGERTDWVMHEYTMDEG-KNQEYYALYKL

FKKSGAGP--PNAG---FLPAENYVMPDSTIPS-W

>AT1G56010.2

ESKLPPGFRFHPKDDELVCDYLMRRSLHNNHRLVLIQVDLNKCEPWDIPKMACVGGKDWYFYSQRDRKYATG

LRTNRATATGYWKATGKDRTILRK--KLVGMRKTLVFYQGRAPRGRKTDWVMHEFRLQ--SPK-EDWVLCRV

FHKNTEGVSLPD-P--N--PSADQLGPKESIPS-W

>AT2G17040.1

MG-L-PGFRFHPTEEELLDFYLKNMVYGKRSSEVIGFLNIYRHDPWDLPGLSRIGEREWYFFVPRERKHGNG

GRPSRTTEKGYWKATGSDRKIISLPKRVIGLKKTLVFYRGRAPGGSKTDWVMNEFRM--P---KD--VLCKI

YRKATSLKTCP--SMP----AN-----SSLLPFEW

>AT5G24590.2

--ALPVGFRFSPTDEELVRYYLRLKINGHDNDRVIREIDICKWEPWDLPDFS-V-TTEWLFFCPLDRKYPSG

SRMNRATVAGYWKATGKDRKIKSG-TKIIGVKRTLVFYTGRAPKGTRTCWIMHEYRATE-DLDQNPFVVCKL

FKKNGAAEPVFSEREPE--PLS--GCAEQF---G-

>AT2G27300.1

--AFP-GFRFSPTDVELISYYLRRKIDGDENSAVIAEVEIYKFEPWDLPEES-L-S-EWFYFCARGRKYPHG

SQSRRATQLGYWKATGKERSVKSG-Q-VVGTKRTLVFHIGRAPRGERTEWIMHEYCIHG-PQ--DALVVCRL

RKN-----KMEGGE--DLKA----THQETI--SDW

>AT3G61910.1

MQ-VPPGFRFHPTEEELLKYYLRKKISNIKIDDVIPDIDLNKLEPWDIQEMCKIGTPDWYFYSHKDKKYPTG

TRTNRATTVGFWKATGRDKTIYTND--RIGMRKTLVFYKGRAPHGQKSDWIMHEYRLDE---SDEGWVVCRV

FKKNNL---CKSSK-TQKLP--SKAALDRLIPA-W

>AT3G12977.1

KGNLPPGFRFHPRDDELVCDYLMRRTVRSLYQVVLIDVDLNKCEPWDIPQTARVGGKEWYFYSQKDRKYATG

YRTNRATATGYWKATGKDRAIQRN--GLVGMRKTLVFYRGRSPKGRKTDWVMHEFRLQ--SLE-EEWVLCRV

FHKNSNGA---D-S--N--P--DQ-KVEESIPS-W

>AT5G22290.1

MDTFP-GFKFSPTDVELISYYLKRKMDGLERSEVIPDLEIYNFEPWDLPDKS-V-S-EWFFFCARGKKYPHG

SQNRRATKMGYWKATGKERDVKSG-E-VIGTKRTLVFHIGRAPKGERTDWIMHEYCVKG-SLD-DAMVVCRV

RRN-----KAPNGTD-EMTG----NEVPTLRPSKW

>AT1G34180.2

MV-FAPGFRFHPTDEELVVYYLKRKICCKKLRNAIGVVDVYKVDPSELPGNSMLGDRQWFFFTPRNRKYPNA

ARSSRGTATGYWKATGKDRVIEY-NSRSVGLKKTLVFYRGRAPNGERTDWVMHEYTMDEG-KNKEYYALYKL

YKKSGAGP--PDAG-I-FLPFENNVMPESMIPS-W

>AT4G28530.1

--KLPPGFRFHPSDEELVCHYLCNKIRAEALKTDLVEIDLHICEPWELPDVAKLNAKEWYFFSFRDRKYATG

YRTNRATVSGYWKATGKDRTVMDPTRQLVGMRKTLVFYRNRAPNGIKTTWIMHEFRL--PPPK-EDWVLCRV

FNKGR---QLP---S-Q--PHHEQSSPSS-ISGD-

>AT5G09330.1

MG-LAPGFRFHPTDVELVRYYLKRKILGKKLLDAIAEVDIYKFEPPDLPDMS-IG--KWHFFCPREKKYASG

VRANRATECGYWKTTGKERPVLCN-SEVVGKIKTLVYHFGKSPRGERTDWVMHEYRLDD----QDTYVVCVL

FKKDGPGPE-PD-NSPSSAPGIDLLELQDLLTAD-

>AT3G49530.1

--DLPVGLRFRPTDEELIRYYLRRKINGHDDDKAIREIDICKWEPWDLPDFS-I-TKEWLYFCPLDRKYPSG

SRQNRATVAGYWKATGKDRKIKSG-TNIIGVKRTLVFHAGRAPRGTRTNWIIHEYRATE-DLSQSPFVICKL

FKKLGE--EVIGDHEPNVAPGS--GYTNDY---K-

>AT3G44290.1

--TFP-GFKFSPTDIELISYYLKRKMDGLERSEIIPEVEIYNFEPWDLPDKS-V-S-EWFFFCARGKKYPHG

SQNRRATKIGYWKATGKERNVKSG-E-VIGTKRTLVFHIGRAPKGGRTEWLMHEYCMIG-SL--DALVICRL

RRN-----KPPNEIG-EVSG----INVPRLLPLRW

>AT5G64060.1

MG-LAPGFRFHPTDVELVRYYLKRKVMGKKFQDAIAEVDIYKFEPPDLPDKS-LG--KWYFFCPREKKYPKG

GKANRSTECGYWKTTGRDRDVSYN-DEVTGKIRTLIYHYGKIPRGDRTDWVIHEYRLED----QDTYVLCVL

FKKNGLGPAGPSYPSPSEAPGNDQVELQDLIQSD-

>AT5G04400.1

MG-LPPGFRFHPSDEELISYYLKKKVQGKPMR--YDEVDICKLEPWDL-AVA-LVTREWFFFSALD-KKTTG

TSMSRAT-QGYW-VTGTDGKIRGG-----GTMKTLVFHRGRSPNGLGTDWVMNEYRVA-PSSQQDSFT----

--ESTASA-IAEQPQPK-EPDEKASFLE-------

>AT1G02230.1

MM--PVGFRFRPNDEEIVDHYLRKNLDSH-VDEVISTVDICSFEPWDLPSKSMI-SRVWYFFSVKEMKYNRG

DQQRRRTNSGFWKKTGKTMTVMRKGREKIGEKRVLVF---KNRDGSKTDWVMHEYHATSP-----TYTVCKV

EFKSSSTGLIPNSGSPTYTP--DDSTCASFLTQE-

>AT1G02250.1

MA--PVGFRFRPTDGEIVDIYLRKNLESH-VDEVISTVDICSFDPWDLPSHSRM-TRVWYFFGRKENKYGKG

DRQIRKTKSGFWKKTGVTMDIMRKGREKIGEKRVLVF---KNHGGSKSDWAMHEYHATFP-----TYTLCKV

KFKVATG-LIPNNGSIGHTP--DGSTCPSFLAQE-

>AT4G01550.1

MV----GYRFYPTGEELINHYLKNKILG----EAISEINICSYDPIYLPSLSKI-SDVWYFFCPKEK---K-

-VTKRTTSSGYWKATGVDRKI-KR-RGEIGIKKTLVYYEGRVPKGVWTPWVMHEYHITC-DQR--NYVICQV

MYK-----SEPDST-PDVHPVN--SNSDAEVGSDY

>AT3G04420.1

ME--PVGLRFRPTDKEIVVDYLRKNSDRH-VDRVISTVTIRSFDPWELPCQSRI-LKSWCFFSPKENKYGRG

DQQIRKTKSGYWKITGKPKPILR--RQEIGEKKVLMFYMSKELGGSKSDWVMHEYHAFSP-----TYTICKV

MFKSSSASLIPND--PTHRP--DDSTCDSFLTQE-

>AT5G22380.1

MA-VTIGFRFYPTEEELVSFYLRNQLEGSD-DRVIPVLDVFEVEPSHLPNVA--GRGEWFFFVPRQEREARG

GRPSRTTGSGYWKATGSPGPVFSK-NKMIGAKKTMVFYTGKAPTGRKTKWKMNEYHAVDPKLR--EFSLCRV

YITTGSSR--P------MLTAVES-HIDVS---EW

>AT5G64530.1

--NLPPGFRFFPTDEELVVHFLHRKASL----DVIPDLDLYHYDPWDLPGKA-LG--QWYFYSRKT----Q-

---ERVTSNGYWGSMGMDEPIYTS-HKKVGIKKYLTFYLGDS----QTNWIMQEYSLPDSSSRPDKWVICRV

YEQNCSEE--------E-------DDLDEV-----

>AT4G01520.1

MM----GYRFSPTGEEVINHYLKNKILG----EAISEINILNHKPSKLPKLARI-SEEWYFFSPIEN---K-

-KMKRTTGSGFWKPSGVDRKI-KR-GVVIGIKKTLVYHEGKSPHGVRTPWVMHEYHITC-HKR--KYVVCQV

KYK-----YEPDST-PE--PVDNN-----------

>AT1G01010.1

MEQ--VGFGFRPNDEELVGHYLRK-IEGRDVEVAISEVNICSYDPWNLRFQSKY-SRMWYFFSRREN--NKG

NRQSRTTVSGKWKLTGESVEVKDQGRGKIGHKRVLVFLDGRYPDKTKSDWVIHEFHYDLP-----TYVICRL

EYKSA---FVPSSGSPHHRP--DT-STDSVISSDW

>AT4G01540.1

MM----GYRFSPTGEEVINHYLKNKLLG----EAISEINILSHKPSKLPKLARI-SEEWYFFSPIEN---K-

-KMKRTTGSGFWKPTGVDREI-KR-GVVIGIKKTLVYHEGKSPHGVRTPWVMHEYHITC-HKR--KYVVCQV

KYK-----YEPDST-PE--PVDNNSGSDAGVGSNY

>AT1G02220.1

ME--PVGLRFCPTDEEIVVDYLWKNSDRH-VDRFINTVPVCRLDPWELPCQSRI-LKAWCFFRPKENKYGRG

DQQMRKTKSGFWKSTGRPKPIMR--RQQIGEKKILMFYTSKE---SKSDWVIHEYHGFSH-----TYTLCKV

MFNSSSPSLIPNN-SPTYRP--DDSTCDSFLTQE-

>AT3G44350.2

MG-LSVGFRFYPTEVELLTYYLRIQL-GGN-ASLIPILDVFSVEPTQLPNLA--GRGEWIFFVPRQEREARG

GRPSRTTGSGYWKATGSPGPVFSP-NRVIGVKKTMVFYTGKAPTGRKTKWKMNEYKAV-PKLR--EFSICRI

YIKSGSSR--P------NLP-GST-QVDLP---DW

>AT1G60350.1

ME---AAFKLNAEDDAIISRYL-KRVVNDSWP--IEDADVFNKNPNVFP-------S--FVIVK--R---TG

K-----TGTGCWR--GRDKPIKST-VKIQGFKKILKF-R-K-PRGYKS-WVMEEYRLTN-NWK--DHVICKI

REKTTS--LLPDKL-P-LHPGTDRLHRDRL---DW

>AT1G60280.1

AE---ATYKLKAEDDVIISRYL-KRIVNDSWP--IEDVDVFNKNPNEFP-------R--FVIVK--R---TG

R-----TGSGCWR--GRDKLIKSK-GKILGFKKILKF-K-K-PREYKS-WVMEEYRLNN-NCK--DHVICKI

REKSTR--LLPDSA-P-LHPGTDEVEGHRV---DW

>AT4G29230.1

---LPAGVKFDPTDQELIE-HLEAKVKDKSHPEFIPTIEICYTHPEKLP-----GTRLRHFFHR--KAYTTG

TRKRRKTESGRWHKTGKTRPVM--G--KQGCKKILVLYKNR--KPEKTNWVMHQYHLGT----EEELVVSKI

FRQSGGGE-SCAAAARHSQPASDGSGLEEL-DGSW

>AT3G56530.1

RSQFPPGYRFVPKDQELIFHYLKKCS------LNVPRVNIYESNPQHL-EK-K-GDKDWFYISERT-K---G

KSNKRV-NGGYWSATVAAQKINAG---IVGYKTSLEYYVGKQSNSVKGDWLMQEYWFES-SDDNEDHALCKI

YKKAEEAE-------PEMIP----EEFNNYFFD--

>AT3G04430.1

--------TFNPPEEELINYYLNNKI------DLVGKQIILHHEPADLP-----GAKESHTWYF--PVEKFG

KLNRTKRS--HWKITGNSRTIK--G--NPGLKKFLVFQSSSSTQQQKTNWIIHEFHLTE----KNELL----

-LQ-------------------EN-----------

>AT1G60300.1

ME---AAYKLYSEDEVIISRYL-KGVVNDSWP--IEDANVFTKNPDKFP-------R--FVIVK--R---TG

K-----TGSGCWR--GRDKLIKSE-GKILGFKKILKF-R-K-PIDYKS-WVMEEYRLTN-NWK--DHVICKI

REKTTS--LLPNTE-P-LHPGTDKLHGDKL---EW

>AT5G14000.1

ME-LPIGYRFHPTEQELILHYLLP--KA-PLP-SII-------PVFDL---FSLGQKKRYFFCKKRE---S-

SNEHRIKGDGYWKPIGK-RPII--C----GSMTEYCL--AFA-SAKSGEWVYNVYERK--------------

-RKRKS-----EE-S-G--P---------------

>AT1G28470.1

KDDLPAGVKFDPSDKEILM-HLEAKVDKKLHPEFIPTLEICYTHPEKLP-----GSKQRHFFHR--KAYTTG

TRKRRKTDGHRWHKTGKTRPVL--S--GEGFKKILVLYRQK--KPEKTNWVMHQYHLGS----EDEPVLSKV

FRQ--------GSE------LV--DGSCSF-----

>AT4G28500.1

RIHLPAGVKFDPTDQEVLE-HLEGKVDAKLHPEFIRTIDICYTHPEKLP-----GNKTRHFFHR--KAYTTG

TRKRRKTDGGRWHKTGKTRPVL--G--RVGYKKILVLYKQK--KPEKTNWVMHQYHLGT----EEELVVSKV

FRQ-GGAA---GGH------TAHN---PAI--SQI

>AT5G56620.1

---LPAGVKFDPTDQELIE-HLEAKVKWSSHPEFIPTIDICYTHPQKLP-----GTRLKHFFHK--RAYTTG

TRKRRKTD-SRWHKTGKTRPVM--G--QQGCKKILVLYKNR--RPEKTNWVMHQYHLGI----EEELVVSKI

FRQSVALQ---SRNT-----VSD-FHMSSS---G-

>AT1G60380.1

MA-------LNAEDEVIISRYL-KPIVNVSWP--IEDADVFNKDPYVFP-------S--FVIVK--R---TG

K-----TGSGCWR--GRDKLIKSE-GKILGFKKILKF-W-K-PREYKS-LVMEEYRLTN-NWK--DHVICKI

REKTTS--LLPDKE-P-LHPGIDKKHREKL---EW

>AT5G50820.1

MDRLPPGFRFDPDDEDLVFEYLAKKVLHRPMD----ELRSCNVDPWDL-----LGEKEVYYFVKKER---E-

-RKGRETLSGYWEAGGRD-----------GRRKTFAFFIGKKPRGTITPWIMYEFRLLSP---GEKWRAVKV

VVK-------------E---ES--DESDGEIQS--

>AT3G55210.1

MS-LPPGFKFVPNDEEVIHCYLKTNV------LHVPLVNIYESNPQTL-EE-K-GDKEWFIITERN-KVDTG

YRQKRVTNGGYWHATVAAQKINAG---VVRNKRPLAYYVGKPSEGVKTDWLMQEYSLDH-S-SNDDYTLCKI

YNKEVGEEQLPDSQ-P---P----------LKKD-

>AT1G60340.1

ME---AAYKLNSEDEVIISRYL-KPVVNDSWP--IEDANVFTKNPNEFP-------R--FVIVK--R---TG

K-----TGSGCWR--GRDKLIKSE-GKILGFKKILKF-W-K-PIEYKS-WVMEEYRLTN-NWK--DHVICKI

REKTTS--LLPNTE-P-LHPGTDKLHGDKL---E-

>AT5G14490.1

LH-LPKGVKFEPTDEEVIE-HLEAKCDGKPHLDFICSVTINYTHPQNLP-----GSKTVFFFNK--HAYQNG

QRKRRRPTDDRWHKTGQTKPVM--G--IQGCKKIMVLYKGF--KPEKSNWVLHQYHLGT----EGEYVVSKI

TKK-----STISVEQ-------GGSVIEDNGSG--

>AT3G01600.1

MA-LPKGVKFEPTDEDIIE-FLEAKCGGEPHVEFIRPVTINYTHPQNLP-----GNKVVFFFHK--QAYGTG

QRKRRKPTDERWHKTGRTKPVM--G--VQGCKKIMVLYKGT--KPEKSNWVLHQYHLGT----GKDYVVSKI

TKN-----NTPGVGP-------DENLSEDNFDGG-

>AT1G25580.1

AG-LPRGVKFDPSDPEIIW-HLLAKSSGSSHPEFIPTVNICYTHPKNLP-----GKSTSHFFHK--KAYSTG

TRKRRKDD--RWHKTGRTKPVV--G--VQGCKKIMVLY-GG--KAVKTNWVMHQYHLGI----EDDYVVSKI

FQRKAEVSATPDYTSDT---EA---DREENCQKDW

>AT5G18300.1

MPELGC-FRFNPSEEELILDYLLPKLFHNTI-YLLEDRNIYAKEPWRLD----F---EWFYFVKRTRK--KG

W------ATGE----LKD----SK-GEVIG-KRNLRFYEGES----KTSWTMREY------SSGNNQRLC--

--H----------------P---------------

>AT1G19040.1

--SLTRGFRFRPADEE-VTDYLMRK--TPEFSFI--KTDLYDKDPWVLP----Y---EWYYFVKK-NEYLNG

F------PRLKWDKQIHD----S--GQNVPFTYGFRFHPTQE----LLSYFIRERYMPW-SHGLNLDLLFTR

TTT--------------------------------

>AT5G41090.1

MG-LPVGMRFRPSDLELAVYFLIKKALGLPMKLTVPECDIFSTHPRDLP-----GSEEWYFYCKKPK---N-

-QVTRTKSYNLWIPTGEKTDVLDPGGELVGIKHSFTFIEEESN-GDQCNWFLDE-SLPL----VDDWTLCHI

FEK-----VKPESS-PSVLP---------------

>AT3G56520.1

ME-LPVGFRFRSTNCEISNYFLKKKALGQPMKRTIPECDIFSRHPHDLP-----GSEEWYYFCRKRD---N-

-QVT-SNSHNLWTPIGEETNVLDPDA-LVGIKRRFTLIEEESCLSDKYNWFMDE-SLPQ----ANDWVLCHI

FKK-----TKPDSS---------------------

>AT1G64105.1

--EIC-----RVSDEEIIENYLRPKI-GSSIPYVVELAELYTVEPWLLP----L---EWFYFGKR-RKY-EG

V------CEGSW--ILEDGCIASK-GEEIG-TTRFRYYKGKE----KMSWFMREYYYK--SRRFNRQVFCII

TEH--------------------------------

>AT1G03490.1

MK-LPVGSRFCPTDLGLVRLYLRNKVQS----SFITTMDIHQDYPWLLPNN--L---EWYYFVPTER---GG

KSVHRKAGGGTWRSNGK-KEIKDGG----GLLQKVVLYK-KAEFGLQTDFMMLEFSLETPSLEEDELTVTQQ

QQQQQQ---------P----LA--TRVDK------

>AT3G56560.1

RSALPPGFKFMPNDKEVINCYLKTNV------LNVPRVNIYESNPQTL--------------SGRN-V--TG

TLPKRF----------------AG---VVGNKRPLAYYVGKPSEGVKTDWLMQEYSLDH-S-STT-------

-----------------------------------

>AT1G60240.1

--------KRQSIEEAIVANYL-KMIDNNVWP--LRSEDVYCKNPWTFP----L-GR--YFFVN--R---SG

L-----TGYGCWR--GRDRVIKSV-GKILGLKKVYKF-R-K-PKSVRI-WAMEEYRFAS-TWK--DYVICKI

RPQ--------------------------------

>AT5G39690.1

MA------------EQVISMGGISS-----------LIKVDEALLKQQ-RE-K-GDKEWFIITERN-KVDTG

NRQKRVTNGGYWHATVAAQKINAG---VVGNKRPLAYYDRKPSEDVKTDWLMQEYSLD-----ND----VRL

HNKEVGEEQQPDSQ-PEMIP----EESNNYLFD—
