# Supplementary material for: Comprehensive analysis of pathogen-responsive wheat NAC transcription factors: new candidates for crop improvement
Source: G3 (Bethesda). 2022 Sep 21;12(11):jkac247. doi: 10.1093/g3journal/jkac247 (PMC9635653; doi:10.1093/g3journal/jkac247)
Supplement: jkac247_Supplemental_File_S6 [file jkac247_supplemental_file_s6.docx]

**File S6** Masked multiple sequence alignment of unique 446 TaNAC protein sequences.

>TraesCS1A01G190100.1

M--GTMTLPPGFRFHPTDDELVGYYLKRRVDNLKIELEVIPVIDLYKCEPWELPEKSFLP

DLEWFFFVPRDRKYPNGSRTNRATTTGYWKATGKDRKVSCDGAVCGVRKTLVFYKGRAPG

GERTDWVMHEYRLCQDLLASNFIGAYALCRVIKRTEAGLLHDAAAKASSQQGSLTPGNAF

PSPQDSHDMGVSDHDTLPPCA----------T------

>TraesCS1A01G261800.1

MSHENDLVMPGFRFHPTEEELIEFYLRRKVEGRRFNVELITFLDLYRFDPWELPAMAVIG

EKEWFFYVPRDRKYRNGDRPNRVTASGYWKATGADRMIRGENRPIGLKKTLVFYSGKAPK

GVRSSWIMNEYRLPPPTTDLFYKSEISLCRVYKRSGIDDGHGRPSSSNAQAASARPSSPS

PSPIMLHAMS----SVAPGARDAPMMGSSST-PG----

>TraesCS1A01G263700.1

MSQQDLQLPPGFRFHPTDEELVMHYLCRRCAGLPISVPIIAEVDLYKFDPWQLPRMALYG

EKEWYFFSPRDRKYPNGSRPNRSAGTGYWKATGADKPVGT--KPLAIKKALVFYAGKAPK

GDKTNWIMHEYRLADVDRNSLRLDDWVLCRIYNKKGASERPGAGDR---TASASPGGSPP

QKPLLPQYYERPSD-SMPRA-ASCERPEVQS-------

>TraesCS1A01G266300.2

M--AQTCLPPGFRFHPTDVELVSYYLKRKIMGKKLFVEAISEVELYKFAPWDLPDKSCLQ

DLEWFFFCPRDKKYPKGSRTNRATPNGYWKTSGKDRTIELNSRIVGLKKTLIFHEGKAPK

GNRTDWVMYEYKMEDETLAGFSKDAYVLCKIFKKSGLGPRI--EQYADENEWENLDSSIF

TAPAPEHFLNSPNHSGLPPMSVLAQSGDMPASNGMDDD

>TraesCS1A01G275900.1

M----GELPPGYRFYPTEEELVRFYLRHMLDGRRGDIEVIPVADVCSLDPWQLPEVHRGA

GEPWFYFCARQDREARGGRPSRTTPSGYWKAAGTPGLVYSAGCPVGTKKTMVFYRGRAPA

GAKTKWKMNEYRAFDDDGRLQVRSEFSLCRLYTRSGSLRQFDRTVAA--EDPASPSASAS

PS---RS------------------QGGADEELVTDWA

>TraesCS1A01G276000.1

M----AALPPGYRFYPTEEELVRFYLRHKLDGSRADIEVIPVADVCSLDPWQLPEVHRGA

GEPWFYFCARQDREARGGRPSRTTPSGYWKAAGTPGLVYSAGHLVGTKKTMVFYRGRAPA

GAKTKWKMNEYRALEEDGVFQVRSEFSLCRLYTSSGNMRQFDRTAVA-----ASSSASPN

TS---RH------------------QEGANEELATDWA

>TraesCS1B01G113000.1

MA-RTPDSPRGLIFAPGDADLITIYLQRKISGLPLAAPYIHNADVYAAEPAALPASASNG

GREWYFFTSVRAQSSRGTRRCRAVGVGTWHSEKARCDVLDTGAVVGYRQPFTYE------

-PKNGWLMLEFSQEDPRPG---EAMPALCKIYKKRRAGRSASKPISSGSKRKAAAAGSSA

EAPRRNPDSPTPA-LVSPASSLTSSNGTTSLLPGQDWA

>TraesCS1B01G113200.1

MA-RTPDSPRGLIFAPGDADLITIYLQRKISGLPLAAPYIHNADVYAAEPAAVPASASNG

GREWYFFTSVRAQSSRDTRRCRAVGVGTWHSEKARCDVLDAGAVVGYRQPFTYE------

-PKNGWLMLEFSQEDPRPG---EAMPALCKIYKKRRAGRSASKPISSGSKRKAAAAGSSA

EAPRRNPDSPTPA-LVSPASSLTSSNGTTSLLPGQDWA

>TraesCS1B01G192200.1

M--GTMTLPPGFRFHPTDDELVGYYLKRRVDNLKIELEVIPVIDLYKCEPWELPEKSFLP

DLEWFFFVPRDRKYPNGSRTNRATTTGYWKATGKDRKVSCDGAVCGVRKTLVFYKGRAPG

GERTDWVMHEYRLCQDLLASNFIGAYALCRVIKRTEAGLLHDAVAKASSQQSSLTPGNAF

PSPQDAHDMGVSDHDTLPLCT----------TAG----

>TraesCS1B01G272600.1

MSHENDLVMPGFRFHPTEEELIEFYLRRKVEGRRFNVELITFLDLYRFDPWELPAMAVIG

EKEWFFYVPRDRKYRNGDRPNRVTASGYWKATGADRMIRGENRPIGLKKTLVFYSGKAPK

GVRSSWIMNEYRLPPPTTDLFYKSEISLCRVYKRSGIDDGHGRPSSSNVQASSARPSSPS

PSPIMLHAMS----PVAPAARDAPMMGSSST-PG----

>TraesCS1B01G274300.1

MSQQDLQLPPGFRFHPTDEELVMHYLCRRCAGLPISVPIIAEVDLYKFDPWQLPRMALYG

EKEWYFFSPRDRKYPNGSRPNRSAGTGYWKATGADKPVGT--KPLAIKKALVFYAGKAPK

GDKTNWIMHEYRLADVDRNSLRLDDWVLCRIYNKKGASERPSAGDR---TASASPGGSPP

QKLLLPQYYERPSD-SMPRA-ASCERPEVQS-AGLD--

>TraesCS1B01G277300.1

M--AQACLPPGFRFHPTDVELVSYYLKRKIMGKKLFVEAISEVELYKFAPWDLPDKSCLQ

DLEWFFFCPRDKKYPKGSRTNRATPNGYWKTSGKDRTIELNSRIVGLKKTLIFHEGKAPK

GNRTDWVMYEYKMEDETLAGFSKDAYVLCKIFKKSGLGPRI--EQYADENEWENLDSSIF

TAPAPEHFLDSPNHSGLPPMSGLTQSGDMSASNGMDDD

>TraesCS1B01G285100.1

M----GELPPGYRFYPTEEELVCFYLRHMLDGRRGDIEVIPVVDVCSLDPWQLPEVHRGA

GEPWFYFCARQDREARGGRPSRTTPSGYWKAAGTPGLVYSACRPVGTKKTMVFYRGRAPA

GAKTKWKMNEYRAFDDDGRLEVRSEFSLCRLYTRSGSLRQFDRTAAA--EDPASPSASAS

PS---RS------------------QGGADEELATDWA

>TraesCS1B01G285200.1

M----AELPPGYRFYPTEEELVRFYLRHKLDGSRADIEVIPVVDVCSLDPWQLPEVHRGA

GEPWFYFCARQDREARGGRPSRTTPSGYWKAAGTPGLVYSAARPVGTKKTMVFYRGRAPA

GAKTKWKMNEYRALEEDGVFQVRSEFSLCRLYTSSGNMRQFDRTTFA-----SSSSAWPN

TS---RP-----------------MQGGADEVLATDWA

>TraesCS1D01G004800.1

MKEGESSLPAGLNFEPTDVELVQDYLRPAIQRLPLP-P-LVNPDLPFMGPWELPGMI--A

-RNKYYLTDHDSCFELGGR--LHTPYGIWKVKTSDMAIRVQHGLVGVKRTMKFH---TYT

GINTNWTMNVYSL-N---GFTMDDGLVLCHVFETDSAPEYD--DSPKGA-------APSN

ASP--HHAGNSSARGVPPTADTASSKGGTTHAAGKD--

>TraesCS1D01G094300.1

MA-RTPDSPRGLIFAPCDADLVTIYLQRKISGSPLAARYIHDADVYAAEPAALPASASSG

GREWYFFTSVRAQSSRGTRRCRAVGVGTWHSEKARCDVLDAGAAVGYRQPFTYE------

-PKNGWLMLEFSQEDPRPG---EAMPALCKIYQKRRAGRSASKPISSGSKRKAAAA----

EAPRR--DSPTPA-FVSPASSLTSSNGTTSLLPGQDWA

>TraesCS1D01G194200.2

M--GTMTLPPGFRFHPTDDELVGYYLKRRVDNLKIELEVIPVIDLYKCEPWELPEKSFLP

DLEWFFFVPRDRKYPNGSRTNRATTTGYWKATGKDRKVSCDGAVCGVRKTLVFYKGRAPG

GERTDWVMHEYRLCQDLLASNFIGAYALCRVIKRTEAGLLHDAAAKASSQQSSLTPGNAF

PSPQDAHDMGVSDHDTLPLCT----------TAG----

>TraesCS1D01G261800.1

MSHENDLVMPGFRFHPTEEELIEFYLRRKVEGRRFNVELITFLDLYRFDPWELPAMAVIG

EKEWFFYVPRDRKYRNGDRPNRVTASGYWKATGADRMIRGENRPIGLKKTLVFYSGKAPK

GVRSSWIMNEYRLPPPTTDLFYKSEISLCRVYKRSGIDDGHGRPSSSNVQASSARPSSPL

PSPIMLHAMS----SVAPAARDAPMMGSSST-PG----

>TraesCS1D01G263800.1

MSQQDLQLPPGFRFHPTDEELVMHYLCRRCAGLPISVPIIAEVDLYKFDPWQLPRMALYG

EKEWYFFSPRDRKYPNGSRPNRSAGTGYWKATGADKPVGT--KPLAIKKALVFYAGKAPK

GDKTNWIMHEYRLADVDRNSLRLDDWVLCRIYNKKGASERPSAGDR---TASASPGGSPP

QKPLLPQYYERPSD-SMPRA-ASCERPEVQS-AGLD--

>TraesCS1D01G266500.1

M--AQACLPPGFRFHPTDVELVSYYLKRKIMGKKLFVEAISEVELYKFAPWDLPDKSCLQ

DLEWFFFCPRDKKYPKGSRTNRATPNGYWKTSGKDRTIELNSRIVGLKKTLIFHEGKAPK

GNRTDWVMYEYKMEDETLAGFSKDAYVLCKIFKKSGLGPRI--EQYADENEWENLDSSIF

-------FLDSPNDSGLPPMSGLSQSGDMSTSNGMDDD

>TraesCS1D01G275500.1

M----GELPAGYRFYPTEEELVRFYLRHMLDGRRGDIEVIPVADVCSLDPWQLPEVHRGA

GEPWFYFCARQDREARGGRPSRTTPSGYWKAAGTPGLVYSADRPVGTKKTMVFYRGRAPA

GAKTKWKMNEYRAFDDDGRLQVRSEFSLCRLYTRSGSLRQFDRTAAA--EDPASSSASPN

PS---RS------------------QGGADEELATDWA

>TraesCS2A01G053500.1

MC------PPGMDYSWSDEELVRF-LAERKADDSLPENVLVGMDLSLIHPQDSP-----G

-NIWYLNQSDDQPYGNGESDIRKAKGGYWKCIDVLRI--TSKSTAGVKFSLEFYEGEAPS

GKRTQWLMHEYQVEQNDE----QEYKSLCTILMQGSKTEDESLSTNAADEQNVAVNSSRE

SSP-----------HHPPASSSSSSHGSTTSKLG----

>TraesCS2A01G063200.1

M-KSDEILMPGFRFHPTDEELVSFYLKKKIQQKPISIELIRQLDIYKFDPWDLPKLASTS

ETESYFYCPRDRKYRNSARPNRVTAAGFWKATGTDRPIYSSERCVGLKKSLVFYKGRAAR

GVKTDWMMHEFRLPSLADNIPLNDSWTICRIFKKTSSMAAQRALSHTEQDIFSAL-ALHF

SNPNTSFQTQPPSQHAAPPL-----------TTGADVN

>TraesCS2A01G063300.1

M-KSEEILMPGFRFHPTDEELVSFYLKKKIQQKPISIELIRQLDIYKFDPWDLPKLANTG

ETEWYFYCPRDRKYRNSARPNRVTAAGFWKATGTDRPIYSSKRCVGLKKSLVFYKGRAAR

GIKTDWMMHEFRLPSLADTIPLNDSWTICRIFKKTSSMAAQRTLTHTKQDLFSAM-ASHF

SSPNTSFQTQPPSQHTAPPF-----------ITGEDVN

>TraesCS2A01G063400.1

M-KSDEILLPGFRFHPTDEELVSFYLKKKIQQKPISIELIRQQDIYKFDPWDLPKLASTS

ETEWYFYCPRDRKYRKSTRPNRVTATGFWKATGTDRPIYSSE------------------

-----------------------------RIFKKTSSMAAQRALSHAEQHLLSAM-ASHF

SN---SFQTQAPSGRTVPPPL--------------DVN

>TraesCS2A01G101000.1

MSELKLALPPGFRFHPTDEEVVTHYLTRKVLRESFSCQVIADVDLNKTEPWDLPGKAKMG

EKEWFFFVHKGRKYPTGTRTNRATEKGYWKATGKDKEIFRGKVLVGMKKTLVFYTGRAPS

GGKTPWVMHEYRLEGE--PRTAKDDWAVCRVFNKDLAAKNAPQMAPAMEDPLAFLDSPSG

TSPEPEHAVVDQQAYCKPKA-EVASSSA----LGLDMG

>TraesCS2A01G101100.1

MS---LALPPGFRFHPTDEEVVTHYLTRKVLRESFSCQVITDVDLNKNEPWELPGKAKMG

EKEWFFFVHKGRKYPTGTRTNRATKKGYWKATGKDKEIFRGKVLVGMKKTLVFYTGRAPS

GGKTPWVMHEYRLEGQ--PHTAKDDWAVCRVFNKDLAAKNAPQMAPAMEDPLAFLDSPSG

TSPEPEHAVVDQQAYCKPKA-EVASSSA----LGLDMG

>TraesCS2A01G101200.1

MSEPWLALPPGFRFHPTDEEVVTHYLTPKIRNPDFSCLMVAYVNLNNTEPWDLPKKAKMG

ETEWFFFVHKDRKYPTGTRTNRATKSGYWKATGKDKEIFRGTVLVGMKKTLVFYRGRAPG

GQKTSWVMHEYRLEGE--PRSAKDDWAVCRLFNKELAAQNAPQMAPAMEDPFAFLDYPSG

TSPELEH-------------------------------

>TraesCS2A01G101400.1

MSEPRLSLPPGFRFHPTDEEVVTHYLTPKAVNNAFSCLVIADVDLNKTEPWDLPGKAKMG

EKEWYFFVHKDRKYPTGTRTNRATEKGYWKATGKDKEVFRGKVLVGMKKTLVFYTGRAPR

GDKTPYVMHEYRLEGQ--PRSARNDWAVCRVFDKDLAAKNAPQTASAMEDPYAFLDSPSG

TSPEPEHAMGEQQSHCKP---EAASSSALLSSLGFDTG

>TraesCS2A01G101900.1

MAGAATSLPPGFRFHPTDEELILHYLRNRAAAAPCPVPIIADVDIYKFDPWDLPSQAVYG

DCEWYFFSPRDRKYPNGIRPNRAAGSGYWKATGTDKPIHDAAQGVGVKKALVFYKGRPPK

GTKTAWIMHEYRLAADPLVSMRLDDWVLCRIYKKTGLASPM--PPLADYDHMADHDSSGG

AGPISEHATH----AVHPSLNGSARKRKAVSERG----

>TraesCS2A01G102000.1

MAGAATSLPPGFRFHPTDEELILHYLRNRAAAAPCPVSIIADVDIYKFDPWDLPSQAVYG

DCEWYFFSPRDRKYPNGIRPNRAAGSGYWKATGTDKPIHDPAQGVGVKKALVFYKGRPPK

GTKTAWIMHEYRLAADPLVSMRLDDWVLCRIYKKTGLASPM--PPLSDYDHMADHDSGGG

AGPFSEHATH----AVHPSLNGSAGKRKAASESS----

>TraesCS2A01G201800.2

MSGSAPDLPPGFRFHPTDEELVVHYLKKKAAKVPLPVTIIAEVDLYKFDPWELPEKATFG

EQEWYFFSPRDRKYPNGARPNRAATSGYWKATGTDKPILASGEKVGVKKALVFYRGKPPK

GLKTNWIMHEYRLTDASSASLRLDDWVLCRIYKKINKAAAGDQQRSSVEDA-----ATAG

DS---HHATSLSHLRASPAPTDASTVGILPQNVGADMA

>TraesCS2A01G306800.1

MAGGSTNLPPGFHFFPSDEELVIHFLRRKAALLPCRPDIVPTLPQNRYDPWELN--ALQA

GNQWYFFSQAT--------QSRASRNGCWNPVGADEAVSSGGSHVGLKKTLVFSIGEPFQ

ATKTNWVMHEYHLLDGNVPDKECSNWVVCRVFESSYDSQVT------EEDM---------

SC--------------------------------DDYD

>TraesCS2A01G311500.1

M-SSSNGVPPGFRFHPTDEELLLYYLKKKIGFEKFDLEVIREVDLNKIEPWDLQERCRIG

QNEWYFFSHKDRKYPTGSRTNRATTAGFWKATGRDKCIRTSYRKIGMRKTLVFYRGRAPH

GQKSDWIMHEYRLEEIDEGGTSEDGWVVCRVFKKKCFFKIAGGGEGSSQSADAGGGSPLG

AAPYHQHHVQVQDLNHRPAADVGSSDGVAADTNGQQWQ

>TraesCS2A01G325900.2

IV-SQPGLPAGVKFDPTGLELLEH-LEGKVRAHDL-IDFIPTTGICYTHPENLPGIKMDG

-RNNHFFHKVSNAYDVGQRKRRKISNSRWHKTGKSKKILNNGVIKGWKKILVLMMRSGTS

RTKTNWTMHQYHLGVDQD---KHGELVVSKVFYQSK--KDE--QSVMNEESDPFAGSSPS

MA---PH--GTPSN---PQ----------------DLQ

>TraesCS2A01G326300.2

--DSQPGLPIGVKFDPTDLELLGH-LEGKIRAHVL-IDFIPTIGICYTHPENLPGVKLDG

-IASHFFHKICNAYDVGTRKRRKISSNRWHKTGKSRHILNNGDIKGWKKIMVLR---GAK

AEKTNWTMHQYHLGVDEN---KDGELVVCKVFFQLPSEKAG--QSAMNEESDSFAGGSPS

AA---SR--GASPHFSSLAVS----------FRGQDMQ

>TraesCS2A01G326400.2

IADSQPGLPIGVKFDPTDLELLGH-LEGKIRAHVL-IDFIPTIGICYTHPENLPGVRLDG

-VASHFFHKICNAYDVGTRKRRKISNSRWHKTGKSRHILNNGDIKGWKKIMVLR---GYK

AEKTNWTMHQYHLGVDEN---KDGELVVCKVFFQLPSEKAG--QSVMDEESDSFAVGSPS

AT---SR--GTSPHFSSLA----------------DMQ

>TraesCS2A01G326500.1

IADSQPGLPAGVKFDPTDLELLEH-LEGKVRAHVL-IDFIPTIGICYTHPENLPGIKMDG

-STGHFFHKVSNAYVVGKRKRRKISNSRWHKTGKSRSILNNGVIKGWKKILVLYIGYGGK

TEKTNWRMHQYHLGVDQD---KQEELVVSKVFYQVQSMNAG--QSLVSEEFDSFAGGSPS

AV---SS--GAPPDIPLPP----------------DMN

>TraesCS2A01G326600.1

IV-SQPGLPAGVKFDPTGLELLEH-LEGKVRAHNL-IDFIPTIGICYTHPENLPGTKMDG

-RKSHFFHKISNAYDVGQRKRRKISNSRWQKTGKSKEISKNGVIKGWKKILVLMMRSGTS

RTKTNWTMHQYHLGVDQD---KHGEPVVSKVFYQSK--KDE--QPVMNEESDPFAGSSPS

MA---PH--GMPPN---PQ----------------DLQ

>TraesCS2A01G328100.1

MA-AGLALRAGAVFRPSGRQIIVEYLGPKATTGKAP-GFVAGVDIFAASPDALPHRKQDG

-EAWYFFAAKP----ASSMP-----GGWWMQYGDAKGYEF--EVYARHRRFEF---RAAD

DEQTRWLMKEYALHKGADGAGA-NDLVVYKVFTKPVVTPPPPPALSSEAENQARKG----

--P--------------------------------N--

>TraesCS2A01G328200.1

MA---PNLTAGYVFQPTGRELIDHYLVPRALGGDFP-GFIEGVDVLSLPPRELPHIRDYG

-EVWFFFAAKS----AGETPG---AGGCWVQYGTEKPYYGSEEAVAFRRRFAYR---MKG

GSPARWLMKEYRLNRDAADEAR-DVFVVHKVYRKPVLPPPADSSSSDSEEEGSERS----

--------------------------------------

>TraesCS2A01G328300.1

MA---PNLTAGYVFQPTGLELIDHYLVPRALGGDFP-GFINGVDVLSLPPRELPHIRDYG

-EVWFFFAAKL----AGETPG---AGGCWVQYGTEKPYYGSEEAVAFRRRFAYR---TKG

GSPTRWLMKEYRLNRDAADEAR-DVFVVHKVYRKPVLPPPTDSSSSDSEEEDFERS----

--------------------------------------

>TraesCS2A01G328400.1

MA---L-AAGGYVFQPTGCELVGYYLMPRALGGFLP-GVIADVDVLSLRPRVLSHRRDYG

-EVWFFFAAKP----AGETPG---AGGCWEQYGQEKAYYDGEEAVALRCRFAYR---SRD

GSQTRWRMKEYWLNRNAAGVPP-DVFVVHKVYRKPLIPPPLPPDSNSSEDEGSESY----

--------------------------------------

>TraesCS2A01G328500.1

MAQASPSLPPGMRFDPNGRCLVRTYLAPKARG--LPLEVIPDVDVYGTRPEALPNRGASG

-KVWHFFTTRPAAAASGGSYVRVVPGGLWVRYDEEKAYADSDDVLGYRCRFAFH---GED

GKLTAWRMKEFRLNEGAAHVA--KNLVAWKVYNDVEEPLSDDE----DEDEDEDDEAAAC

----------------------------------DDLT

>TraesCS2A01G338300.1

M-QHAMDLPPGFRFHPTDEELITHYLAKKVADARFAALAVSVADLNKCEPWDLPSLARMG

EKEWYFFCLKDRKYPTGLRTNRATESGYWKATGKDKDIFRGKALVGMKKTLVFYTGRAPK

GDKSGWVMHEYRINAKLHAASSRNEWVLCRVFKKSLLVSSAPASSAAMEEISSVAASGSG

EAPATDHSSGYHRGDMAPCGNPAACKGERERDTGSDVN

>TraesCS2A01G354300.1

MA---RSLPPGHAFEPSGHQIITQYLAPKALRGDTP-GHVAGVDVFAASPPALPSRRRHG

-EAWYFYGAQA----SGDAP-----GGCWARYGREKGYVHGAEAVAFRSRFAFHVPRGDG

GAPTPWLMKEYRLNKGAAGKA--NDCVVRKIFTKPVVAAPPPPACSSDEEDSSGPDGEED

----------GPPRRARPS-------------------

>TraesCS2A01G363700.1

TADEEDGLPPGEAFDPALDAIVARYLRRAL--DGLP-RQIHDADVYGAHPGFLPA----G

RPEWFFFVCRAQ--CQGGR--RRAGPGAYRLGSEARLLGGAA-----CHAFRYYEDEAEV

GRETEWRMDEY---DSAA---AA-DMVVCKLYPARGT----HQASPA--HQDMNKPAVPD

AAPANAH-------KARPRRAVTVRRVSRTEEYGKDFP

>TraesCS2A01G382400.1

M-EIESTLPPGFRFYPSDQELVCHYLCKKVTNERASQGTLVEVDLHAREPWELPDVAKLT

ASEWYFFSFRDRKYATGSRTNRATKTGYWKATGKDREVRSPAAVVGMRKTLVFYQGRAPN

GSKTSWVMHEFRLDSPHLDAILQEDWVLCRVFQKQKVDGEQDNARSSAGSSQAAQEASSD

SAPMNQYLDQYPQESVSPTMGIGARGGAGDENSGEDMA

>TraesCS2A01G386300.1

M-NAFSHVPPGFRFHPTDEELVDYYLRKKVQLKRIDLDIIKDVDLYKIEPWDLQERCKIG

QNDWFFFSHKDKKYPTGTRTNRATTAGFWKATGRDKPIYVKHCLVGMRKTLVFYKGRAPN

GQKLDWIMHEYRLETNENGAPHDEGWVVCKVFKKRVATVQRMAATDSNNDHVAFMASPRQ

NA---EYFLQLPQL-EMPGLPNQTHDGQASRASAGEWR

>TraesCS2A01G409900.1

M-K----YPVGFRFAPTDEELVEYYLLPRLQGRQVP-NAIIQDNVYQCHPDDL-GKYKDG

DNNWFFLTSRTRKYVNGGRPARTTGRGRWKASTGTTEVV---ATVTYESGLAYHEGPIKT

ERKTKWLMHELTVPEDKSKTDTLDEYVMCRIYVTPRKRKRNDEAGPSEEAASPELGVEPE

TSPAF-----RPQMQKQPETEGDAAHAAQQPYFGQNWQ

>TraesCS2A01G459700.1

MDVFPLNLPPGYHFAPSGDELIVHYLRRKLDGLPPHLPIFNDVPIIDYRPEQITDEFDFG

GARWYFFTKRTRKYATGSRPDRTTGNGFWKATGPVREIPICPKLVGRARTLVFYTGPE--

-KPTHWTMYEYENRTSEANVDKLGEWVLCTIQKSQR-DQKKEAKSKAKGKARNGADAWPE

TGPIE--YNNMMYQ---PANS----------YAG----

>TraesCS2A01G462500.1

M--VEARLPPGFRFHPRDHELVLDYLCHKLSGGGGGVD-MVDVDLNKCEPWELPDAACVG

GKEWYFFSRHDRKYATGQRTNRATHTGYWKATGKDRVITGDGAVVGMRKTLVFYLGRAPR

GTKTEWVMHEFRVEGRPPPFLLEEDWVLCRVFYKSTTAAPTPASDESSGSLSSDLGEITD

TGPTS--MVEGSRGDPKPEWS-----------DG----

>TraesCS2A01G561700.1

MAGAFSHLRPGFRFHPTDQELVSFYLRRKVLGHG---GFIPEVDLYKLQPHHLPGKSFSS

KVEWYLFAPRGRKYPTGLRMERATPRGFWKSTGKDRPVMHKGIVVGMKKTLVFHIGKAPS

GTRTDWVMHEYRLHG----HHIQDTYALCRVFNKNMASPSTNASGDVDEDLMQFVPADSG

QDP--KHVQDVMQSGKDPIQVASMENDFMQSALGVDTD

>TraesCS2A01G561800.1

MAAAAAQLRPGIRFYPTDQELVGWLLRSKVLGHALHIDFIPVVNAYKFEPQELPDKSFSS

KVESYFFAPRGRKYPTGLRMKRATENGFWKSTGKDHPIMHNGTIIGMKKTLVFHAGRAPK

GTRTDWVMHEYRLHGHCN----QDTYALCRVFNKNTVTPSTDVSGGADEDPMHFLPANSD

TS---KFNNDLMHFEKDPIPVAVADNGGVQLSGGGDKG

>TraesCS2A01G565900.1

M-QEESCVPPGFRFHPTEVELVGYYLARKVAAQKIELDIIQEVDLYRIESWDLQGRCGTG

SSEWYFFSFKDRKYPSGTRTNRATAAGFWKAIGRDKRVTSSRGVMGMRKTLVLFRTPAPN

GRKTDWIIHEYRRQNSLHAPTQEEGWVVCRAFQKPIPNQRP-----SAP-----------

--------------------------------------

>TraesCS2A01G566000.1

M-QEESCVPPGFRFHPTEEELVGYYLARKVAAQTIDLDIIQEVDLYRIEPWDLQDRCVGG

SSEWYFFSFKDRKYPSGTRTNRATAAGFWKATGRDKPVTSSRGVIGMRKTLVFYQGRAPN

GRKTDWIIHEYRLQTSEHAPTQEEGWVVCRAFQKPTPNQRP-----S--DA-----IQGG

SGPQDNYFGNIPQLIESPPA-LGCGDDAVLQAAGIDWN

>TraesCS2A01G566100.1

M-QEESCVPPGFRFHPTEEELVGYYLARKVAAQKIDLDIIQEVDLYRIEPWDLQERCGGG

SSEWYFFSFKDRKYPSGTRTNRATAAGFWKATGRDKPVTSSRGVIGMRKTLVFYRGRAPN

GRKTDWIIHEYRLQTNEHAPTQEEGWVVCRAFQKPTPNQRP-----STPDA-----GR-G

SAPQGQYFNNIPQLIESPPTTAGGGDAGYDIAAGMDWN

>TraesCS2A01G566200.1

M-QEESCVPPGFRFHPTEEELVGYYLARKVAAQKIDLDIIQEVDLYRIEPWDLQEKCGGG

SSEWYFFSFKDRKYPSGTRTNRATAAGFWKATGRDKPVMSSRGVTGMRKTLVFYRGRAPN

GRKTDWIIHEYRLQTNEHAPTQEEGWVVCRAFQKPAPNQRP-----SAPNA-----GQ-G

STPQGQYFGNIPQLIESPPTTAGCGDAGYDIAAGIDWN

>TraesCS2A01G566300.1

M-QEESCVPPGFRFHPTEEELVGYYLARKVAAQNIDLGIIQEVDLYRTEPWDLQEKCGGG

SSEWYFFSFKDRKYPSGTRTNRATAAGFWKATGRDKPVTSSRSVIGMRKTLVFYRGRAPN

GRKTDWIIHEYRLQTSEHGPTQEDGWVVCRAFQKPTPNQRQ-----SALDA-----GQ-G

SAPQSQYFNNIPQLIQSPPPAAGCSEAGYDLATGIDWN

>TraesCS2A01G566400.1

M-QEVSCVPPGFRFHPTEEELVGYYLAQKVAAQKIDLDIIPEVDLYRIEPWDLQERCGGG

SSDWYFFSFKDRKYPSGTRTNRATAAGFWKATGRDKPVMSSRGVIGMRKTLVFYRGRAPN

GRKTDWIIHEYRLQTSEHAPTQEEGWVVCRAFQKPTPNQRP-----SAPDA-----DQ-G

--PQGQHFSNIPQLIESPPTTAGCGDAGYDVATGIDWN

>TraesCS2B01G067400.1

MC------PPGMNYSWSDEELVRF-LAERKAEDSLPENVLVGMDLTLIHPLDSP-----G

-NIWYLNQSDDQPYGNGESDIRKAKGGYWKCIDVLRI--TSKSTAGVKFSLEFYEGEAPS

GKRTQWLMHEYQVEQNDE----QEYKSLCTIFMQGSKTEDESLSTNAADEQNVAVNCSRE

SSP-----------PHPPASSSSSSHGSTTSKLG----

>TraesCS2B01G075900.1

M-KSDEILMPGFRFHPTDEELVSFYLKKKIQQKPISIELIRQLDIYKFDPWDLPKLASTG

ETEWYFYCPRDRKYRNSARPNRVTAAGFWKATGTDRPIYSSERCVGLKKSLVFYKGRAAR

GIKTDWMMHEFRFPSLADNIPLNDSWTICRIFKKTSSMAAQRALSHTEQDLFSAL-ALHF

SNPNTSFQTQPPSQHVAPPL-----------ITGADVN

>TraesCS2B01G076000.1

M-KSDEILMPGFRFHPTDEELVSFYLKKKIQQKPISIELIRQLDIYKFDPWDLPKLANTG

ETEWYFYCPRDRKYRNSARPNRVTAAGFWKATGTDRPIYSSKRCVGLKKSLVFYKGRAAR

GIKTEWMMHEFRLPSLADTIPLNDSWTICRIFKKTSSMAAQRALTHTKQDLFSAM-ASHF

SSPSTSF----------------------------DID

>TraesCS2B01G076100.1

M-KSDEILLPGFRFHPTDEELVSFYLKKKIQQKPISIELIKQQDIYKFDPWDLPKLASTS

ETEWYFYCPRDRKYHNSARPNRVTAAGFWKATGTDRPIYSSERCVGLKKSLVFYKGRAAR

GIKTDWMMHEFRLPSLADNIPLNDSWTICRIFKKPGSMAAQRALSHAEQ-------ASHF

SS---SFQTQAPSHHTAPPPL----------SAGADVN

>TraesCS2B01G100600.1

MV-ADLGLTRGYKLEPHDDEAVEYYLLRRLLGQELPLEIILEDDPLSAPPWELLNKH---

EDNAFFFAHGQTIDSKGSRNKRTCGGGCWEVLVDDKEIVDSGMEITWKYQLNFH---DGE

KGSTGWVMHEYSVTAPPD------SLRLYRILFSGHGKKRKREGARARQDEESELHRSSE

AGPAAEF-------GTLPSSG----------VCGLDGQ

>TraesCS2B01G118200.1

MSEPQLALPPGFRFHPTDEEVVTHYLTRKVLRESFSCQVITDVDLNKNEPWELPGLAKMG

EKEWFFFVHKGRKYPTGTRTNRATKKGYWKATGKDKEIFRGKVLVGMKKTLVFYTGRAPS

GGKTPWVMHEYRLEGE--PRTAKDDWAVCRVFNKDLAARNAPQMAPAMEDPLAFLDSPSG

TCPEPELAMGDQQAYCKPKA-EVASSSALLSSLGLDTA

>TraesCS2B01G118300.1

MSEPQLALPPGFRFHPTDEE---------VLRESFSCQVITDVDLNKNEPWELPGLAKMG

EKEWFFFVHKSRKYPTGTRTNRATKNGYWKATGKDKEIFRGKVLVGMKKTLVFYTGRAPS

GGKTPWVMHEYRLEGQ--PRTAKDDWAVCRVINKDLAARNAPQMAPVMDDPLAFLDSQSC

SSPEPEHVMGDQQAYCKPKA-EVASSSALLSSLGLDTA

>TraesCS2B01G118400.1

MSERQLALPPGFRFHPTDEEVVTHYLTRKVLRESFSCQVITDVDLNKNEPWELPGLAKMG

EKEWFFFVHRGRKYPTGTRTNRATKNGYWKATGKDKEIFRGKVLVGMKKTLVFYTGRAPS

GGKTPWVMHEYRLEGQ--PRTAKDDWAVCRVINKDLGARNAPR----MEDPLAFLDSPSG

TSPEAEHAMGDQQVYSKPKA-EVASSSALLSSLGLDTA

>TraesCS2B01G118500.1

MSEPRLSLPPGFRFHPTDEEVVTHYLTPKAVNNAFSCLVIADVDLNKTEPWDLPGKAKMG

EKEWYFFVHKDRKYPTGTRTNRATEKGYWKATGKDKEIFRGKVLVGMKKTLVFYTGRAPR

GDKTPYVMHEYRLEGQ--PRSAKNDWAVCRVFDKDLAAKNAPPMAPAMEDPYAFLDSPSG

TSPEPEHAMGDQQTHCKP---EAASSSALLSSLGFDAG

>TraesCS2B01G119000.1

MAGAATSLPPGFRFHPTDEELILHYLRNRAAAAPCPVPIIADVDIYKFDPWDLPSQAVYG

DCEWYFFSPRDRKYPNGIRPNRAAGSGYWKATGTDKPIHDAAQGVGVKKALVFYTGRPPK

GTKTAWIMHEYRLAADPLVSMRLDDWVLCRIYKKTGLASPM--PPLADYDHMADHDSSGG

AGPFSEHATH----AVHPSLNGSAGKRKAASEPG----

>TraesCS2B01G119100.1

MAGAATSLPPGFRFHPTDEELILHYLRNRAAAAPCPVPIIADVDIYKFDPWDLPSQAVYG

DCEWYFFSPRDRKYPNGIRPNRAAGSGYWKATGTDKPIHDAAQGVGVKKALVFYKGRPPK

GTKTAWIMHEYRLAADPLVSMRLDDWVLCRIYKKTGLASPM--PPLADYDHMADHDSGGG

AGPFSEHATH----AVHPSLNGSAGKRKAASESS----

>TraesCS2B01G228900.1

MSGSAPELPPGFRFHPTDEELVVHYLKKKAAKVPLPVTIITEVDLYKFDPWELPEKATFG

EQEWYFFSPRDRKYPNGARPNRAATSGYWKATGTDKPILASGEKVGVKKALVFYRGKPPK

GLKTNWIMHEYRLTDASSASLRLDDWVLCRIYKKINKAAAGDQQRSSVEDA-----ATAG

DS---HHPTSLSHLRASPAPTDASTVGILPQNVGADMA

>TraesCS2B01G323500.1

MTGGSTNLPPGFHFFPSDEELVIHFLRRKAALLPCRPDIVPTLPQNRYDPWELN--ALQA

GNQWYFFSQAT--------QSRTSRNGCWNPIGADEAVSSGGSHVGLKKTLVFSIGEPFQ

ATKTNWVMHEYHLLDGNGPDKECSNWVVCRVFESSYDSQVS------EEDM---------

SC--------------------------------DDYD

>TraesCS2B01G328300.1

M-SSSNGVPPGFRFHPTDEELLLYYLKKKIGFEKFDLEVIREVDLNKIEPWDLQERCRIG

QNEWYFFSHKDRKYPTGSRTNRATTAGFWKATGRDKCIRTSYRKIGMRKTLVFYRGRAPH

GQKSDWIMHEYRLEEIDEGGTSEDGWVVCRVFKKKCFFKIAGGGEGSSQSADAGAGSPLG

AAPYHQHHVQVQDLNHRPAADVGSSDGVAADTNGQQWQ

>TraesCS2B01G343600.1

M-QHAMDLPPGFRFHPTDEELITHYLAKKVADARFAALAVSVADLNKCEPWDLPSLARMG

EKEWYFFCLKDRKYPTGLRTNRATESGYWKATGKDKDIFRGKALVGMKKTLVFYTGRAPK

GDKSGWVMHEYRLNAKLHAASSKNEWVLCRVFKKSLVVSSAPASSAAIEEISSVADSGSG

EAPATDHSSGYHRGDMAPCGNPAACKGERERDTGSDVN

>TraesCS2B01G353800.1

MA---PNLPLGYVFRPKARELIQHYLAPKALGGYTP-GLVAGVDVFSAAPDALPHRRENG

-EVWYFFAAHP----AGERP-----GGCWITYGPEKAYRGGTEAVAFRRNLAYY---ARG

GARTPWLMAEYRLNKGGAGE-A-NDCVVRKVFMKPAVPPPPAR---SSDDESAGSSGEE-

--------------------------------------

>TraesCS2B01G353900.1

MA---PNLTAGYVFQPTGRELIDHYLVPRALGGDFP-GFIEGVDVLSLPPSELPHIRDYG

-EVWFFFAAKP----AGERPG---AVGCWVQYGTEKPYYGGEEAVAFRRRFAYR---TKG

GSPTRWLMKEYRLNRDAADEAR-DVFVVHKVYRKPVLPPPADSSS--SEEEGSERS----

--------------------------------------

>TraesCS2B01G355300.2

IV-SQPGLPVGVKFDPTGLELLEH-LEGKVRAHDL-IDFIPTIGICYTHPENLPGTKMDG

-TKGHFFHQISNAYDVGQRKRRKISNSRWHKTGKSKQISNNGVIKGWKKILVLRMRSGTS

HKNTNWTMHQYHLGVDQH---KHGELVVSKVFHQSE--KDE--QPVMNEESDPFAGSSQS

MA---PH--VTPPD---P-----------------DLQ

>TraesCS2B01G355400.1

IADSQPGLPAGVKFDPTDLELLEH-LEGKVRVHVL-IDFIPTIGICYTHPENLPGIKMDG

-STGHFFHKVSNAYVVGKRKRRKISNSRWHKTGKSRSILNNGVIKGWKKILVLYIGYGGK

TEKTNWRMHQYHLGVDQD---KQEELVVSKVFYQVQSMNAG--QSLVNEEFDSFAGGSPS

AG---SH--GMPPDVSLLT----------------DMQ

>TraesCS2B01G355500.1

IADSQPGLPAGVKFDPTDLELLEH-LEGKVGAHVL-IDFIPTIGICYTHPENLPGIKMDG

-STGHFFHKVSNAYVVGKRKRRKISNSRWHKTGKSRSILNNGVIKGWKKILVLYIGYGGK

TEKTNWRMHQYHLGVDQD---KQEELVVSKVFYQVQSMNAG--PSA-----DSL--GSPS

AV---SS--GAPPDVSLPP----------------HTQ

>TraesCS2B01G355600.1

IADSQPGLPTGVKFDPTDLQLLGH-LEGKIRAHVL-IDFIPTIGICYTHPENLPGVKSDG

-TASHFFHKICNAYDVGTRKRRKISNSRWHKTGKSRHILNNGDIKGWKKIMVLR---GAK

AEKTNWTMHQYHLGVDEN---KDGELVVCKVFFQLPSEKAG--QSVMNEESDSFAVSSPF

AA---SR--GTPPDFSALA----------------DLP

>TraesCS2B01G355700.1

IGDSQSGLPIGVKFDSTDLELLGH-LEGKIRAHVL-IDFIPTIGICYTHPENLPGVKLDG

-IASHFFHKICNAYDVGTRKRRKIRSNRWHKTGKSRHILNNGDIKGWKKIMVLR---GAK

AEKTNWTMHQYHLGVDEN------EMYVC-------------------------------

GA------------------------------------

>TraesCS2B01G359200.1

MA---L-AEAGYVFQPTGRELVGHYLMPRALGGFLP-GVIEGVDVLSLRPRALSHRRDYG

-EVWFFFAAKP----AGETP------GCWVQYGQEKAYYGGEEAVAFRRRFAYR---SRD

GSPTRWRMKEYRLNRNAAGVPA-DVFVVHKVYRKPLIPRPPPPDSSSSEDEGSESY----

--------------------------------------

>TraesCS2B01G375000.1

MA---RNLPPGHAFEPSGHQIITQYLAPKALRGDTP-GHVAGVDVFSASPAALPSRRRHG

-EAWYFYGAQA----AGDAP-----GGCWARYGREKGYVHGAEAVAFRRRFAFHVPRGDG

RAPTPWLMKEYRLNKGAAGKA--DDCVVRKIFTKAVAVAPPTPPCSSDEEDSSGPDGEED

----------GPPRRARPS-------------------

>TraesCS2B01G376900.1

KG-LEIGLHKYLRFNPTDQELMER-LEAKVDGDPL-IKFIPTIG--YPHPQQLPGVTMDG

-TIKHFFCRP-RAFESGRRKHRKIKAEAWHKTGKSMALKANGRLTGWKNILVLYTN----

TRKTNWVMHEYRLSDVED---EGGELVLCKIFYQTEPKRRS--V---QENKCPT------

--------------------------------------

>TraesCS2B01G381700.1

MADEEDGLPPGEAFDPTPDVIVGRYLRRAL--DGLP-RQIHDADVYGAHPGFLPA----D

RSEWFFFVCRAQ--CQGGR--RRAGPGAYRLGSEARLLGGAA-----CHAFRYYEDEAEV

GKETEWRMDEY---DSAA---AA-DMVVCKLYTARGT----HEASPS--RPDMNKPATAD

AAPANVH-------KARPRRAATVRRVSRTEEYGKDFP

>TraesCS2B01G399700.1

M-EIESTLPPGFRFYPSDQELVCHYLYKKVTNERASQGTLVEVDLHAREPWELPDVAKLT

ASEWYFFSFRDRKYATGSRTNRATKTGYWKATGKDREVRSPAAVVGMRKTLVFYQGRAPN

GSKTSWVMHEFRLDSPHLDAILQEDWVLCRVFQKQKLDGEQDNARSSARSSQVAQEASGD

AAPMNQYLDQYPQEGSSPMM---ATGGAGDEDSGEDTA

>TraesCS2B01G403300.1

M-NVFSHVPPGFRFHPTDEELVDYYLRKKVQLKRIDLDIIKDVDLYKIEPWDLQERCKIG

QNDWFFFSHKDKKYPTGTRTNRATTAGFWKATGRDKPIYVKHCLVGMRKTLVFYKGRAPN

GQKLDWIMHEYRLETNENGAPHDEGWVVCKVFKKRVATVQRMATVDSNHDHAAFMASPRQ

NA---EYFLQLPQL-EMPGLPNQTHDGQASRASAGEWR

>TraesCS2B01G481400.1

M-QFALNLPPGYHFTPTEAELIVHYLRRKLDGLPPHLPIFNDVPITDYRPEQITDEFDFG

GGRWYFFTKRTRKYATGSRPDRTTGKGFWKGTGPVREIPINPKLVGHARTLVFYTGPD--

-EPTYWTMYEYENHTSEANIDKLGEWVLCTIQKKKA-------GGKAKGKAQNRADAWPE

TGPMQ--YNSMMYQ---PATS----------YAG----

>TraesCS2B01G484100.1

M--VEARLPPGFRFHPRDHELVLDYLCHKLSGGGAGVD-MVDVDLNKCEPWELPDAACVG

GKEWYFFSRHDRKYATGQRTNRATHTGYWKATGKDRVITGDGAVVGMRKTLVFYLGRAPR

GTKTEWVMHEFRAEGRPPPFLLEEDWVLCRVFYKSTTAAPTPASDESSGSQSSEVGAITD

TSPRS--MVESSSGDPKPEWS-----------DG----

>TraesCS2B01G616900.1

M--EMPPLPPGYRFHPTDVELTLYYLKRKLLGKKLLCNAVAEVDIYKHAPWDLPAKSSMG

DLQWYFFCTRGRKYSVGQRANRSTEGGYWKATGKDRQVVYENRTVGMKRTLVFHAGKAPK

GTRTDWVMYEYRLVQGEIAGVRLDDSVLCKVHKKSGPGPKIG-EQYAEEEE-----GDTS

PGPRAEQ---VPSSLLEPAAGGHAPDGSSSQSNGLDLE

>TraesCS2B01G627000.1

M-QEESCVPPGFRFHPTEEELVGYYLARKVAAQTIDLDIIQEVDLYRIEPWDLQERCSGA

SSEWYFFSFKDRKYPSGTRTNRATAAGFWKATGRDKPVTSSRGVIGMRKTLVFYRGRAPN

GRKTDWIIHEYRLQTNEHAPTQEEGWVVCRAFQKPAPNQRP-----SAPDA-----GQ-G

STPQGQYFGNIPQLIQSPPTTAGGGDAGYDIAAGIDWN

>TraesCS2B01G627100.1

M-QEESCVPPGFRFHPTEEELVGYYLARKVAAQKIDLDIIQEVDLYRIEPWDLQERCGGG

LSEWYFFSFKDRKYPSGTRTNRATAAGFWKATGRDKPVISSRGVIGMRKTLVFYRGRAPN

GRKTDWIIHEYRLQTSEHAPTQDEGWVVCRAFQKPAPNQRP-----SAPDA-----GQGG

SA--DKYFDNIPQLIQSPQT-LGCGDDAILQVLGIDWN

>TraesCS2B01G627200.1

M-EEESCVPPGFRFHPTEEELVGYYLARKVAAQKIDLDIIQEVDLYRIEPWDLQERCGGG

SSEWYFFSFKDRKYPSGTRTNRATAAGFWKATGRDKPVMSSRGVIGMRKTLVFYRGRAPN

GRKTDWIIHEYRLQTNEHAPTQEEGWVVCRAFQKPTPNQRA-----SAPDP-----GQGG

SAPQGQYFGNIPQLIESPPTTAGCGDTGYGIAAGIDWN

>TraesCS2D01G061500.1

M-KSDEILMPGFRFHPTDEELVSFYLKKKIQQKPISIELIRQLDIYKFDPWDLPKLASTG

ETESYFYCPRDRKYRNSARPNRVTAAGFWKATGTDRPIYSSERCVGLKKSLVFYKGRAAR

GIKTDWMMHEFRLPSLADNIPLNDSWTICRIFKKTSSMAAQRALSHTEQDLFSAL-ALHF

SYPNTSFQTQPPSQHTSPPI-----------ITGEDVN

>TraesCS2D01G061600.1

M-KSDEILLPGFRFHPTDEELVSFYLKKKIQQKPISIELIMQQDIYKFDPWDLPKLASTS

ETEWYFYCPRDRKYRNSARPNRVTAAGFWKATGTDRPIYSSERCVGLKKSLVFYKGRAAR

GIKTDWMMHEFRLPSLTDNIPLNDSWTICRIFKKTGSMAAQRALSHAEQHLLSAM-ASHF

SN---SFQTQAPSHRTAPPSL----------IAGADVN

>TraesCS2D01G083700.1

MV-ADLGLTRGYKLEPLDEEAVEYYLLRRLLGQELPLEIILEDDPLSAPPWELLNKH---

EDDAFFFAYGQTIDSKESRNKRTCGGGCWEVVVDDKEIVDSGMEITWKYQLNFH---DGE

KGSTGWVMHEYSVTAPPD------SLRLYRILFSGHGKKRKREGERARQAEESD--VSPQ

AAPASEF-------GTLPSSS----------VCGLDGQ

>TraesCS2D01G100600.1

MSEPKLVLPPGFRFHPTDEEVVTHYLTRKVLRESFSCQVIADVDLNKTEPWDLPGKAKMG

EKEWFFFVHKGRKYPTGTRTNRATEKGYWKATGKDKEIFRGKVLVGMKKTLVFYTGRAPS

GGKTPWVMHEYRLEGE--PRTAKDDWAVCRVINKDLAARNALQMAPAMEDPLAFLDSPSG

SSPEPVHAMGDQQAYSKPKA-QVASSSALLNSLGLDTV

>TraesCS2D01G100700.1

MSEPQLALPPGFRFHPTDEEVVTHYLTRKVLRESFSCQVIADVDLNKTEPWDLPGKAKMG

EKEWFFFVHKGRKYPTGTRTNRATEKGYWKATGKDKEIFRGKVLVGMKKTLVFYTGRAPS

GGKTPWVMHEYRLEGE--PRTAKDDWAVCRVINKDLAARNALQMAPAMEDPLAFLHSPSG

TSPEPEHAMGDQQAYCRPKA-EVASSSALLSSLGLDTA

>TraesCS2D01G100800.1

MSEPWLALPPGFRFHPTDEEVVTHYLTPKIRNPDFSCLMVSYVNLNNTEPWDLPKKAKMG

QSEWFFFVHKDRKYPTGTRTNRATKSGYWKATGKDKEIFRGTVLVGMKKTLVFYRGRAPG

GQKTPWVMHEYHLEGE--PRSAKDDWAVCRLLNKDMAAKNAPQMAPAMEDPFAFLDSPSG

TSPGPEH-------------------------------

>TraesCS2D01G100900.1

MSEPRLSLPPGFRFHPTDEEVVTHYLTPKAVNNAFSCLVIADVDLNKTEPWDLPGKAKMG

EKEWYFFVHKDRKYPTGMRTNRATEKGYWKATGKDKEIFRGKVLVGMKKTLVFYTGRAPR

GDKTPYVMHEYRLEGQ--PRSAKNDWAVCRVFDKDLAAKNAPQTAPAMEDPYAFLDSPSG

TSPEPEHAMGDQQTHCKP---EAASSSALLSSLGFDAG

>TraesCS2D01G101300.1

MAGAATSLPAGFRFHPTDEELILHYLRNRAAAAPCPVPIIADVDIYKFDPWDLPSQAVYG

DCEWYFFSPRDRKA------------------------------SAVKKALVFYTGRPPK

GTKTAWIMHEYRLAADPLVSMRLDDWVLCRIYKKTGLASPM--PPLADYDHMADHDSSGG

AGPFSEHATH----AVHPALNGSSGKRKAASERA----

>TraesCS2D01G101400.1

MAGAATSLPPGFRFHPTDEELILHYLRNRAAAAPCPVPIIADVDIYKFDPWDLPSQAVYG

DCEWYFFSPRDRKYPNGIRPNRAAGSGYWKATGTDKPIHDAAQGVGVKKALVFYKGRPPK

GTKTAWIMHEYRLAADPLVSMRLDDWVLCRIYKKTGLASPM--PPLSDYDHMADHDSGGG

AGPFSEHATH----AVHPSLNGSAGKRKAASESS----

>TraesCS2D01G214100.1

MSGSAPELPPGFRFHPTDEELVVHYLKKKAAKVPLPVTIIAEVDLYKFDPWELPEKATFG

EQEWYFFSPRDRKYPNGARPNRAATSGYWKATGTDKPIMASGEKVGVKKALVFYRGKPPK

GLKTNWIMHEYRLTDASSASLRLDDWVLCRIYKKINKAAAGDQQRSSVEDA-----ATAG

DS---HHATSLSHLRASPAPTDASTVGILPQNVGADMA

>TraesCS2D01G305300.1

M-GGSTNLPPGFHFFPSDEELVIHFLRRKAALLPCRPDIVPTLPQNRYDPWELNGKALQA

GNQWYFFSQAT--------QSRTSRNGCWNPIGADEAVSSGGSHVGLKKTLVFSIGEPFQ

ATKTNWVMHEYHLLDGNGPDKECSNWVVCRVFESSYDSQVS------EEDM---------

SC--------------------------------DDYD

>TraesCS2D01G324700.1

M-QHAMDLPPGFRFHPTDEELITHYLAKKVADARFAALAVSVADLNKCEPWDLPSLARMG

EKEWYFFCLKDRKYPTGLRTNRATESGYWKATGKDKDIFRGKALVGMKKTLVFYTGRAPK

GDKSGWVMHEYRLNAKLHAASSRNEWVLCRVFKKSLVVSSAPASSAAIEEISSVAASGSG

EAPVTDNSSGYHRGDMAPCGNPAACKGERERDTGSDVN

>TraesCS2D01G334200.1

MA---PNLPPGYVFRPKARELIQHYLAPKALGGYTP-GLVAGVDVFSAAPDALPHRRESG

-EVWYFFAAHP----AGERP-----GGCWIPYGPEKAYRGGGEAVAFRRRLAYY---ARG

GARTPWLMAEYRLNKGGAGE-A-NDCVVRKVFTKPAVPPPPAS---SSDDESAGSSGEEA

RA------------------------------------

>TraesCS2D01G334300.1

MA---PNLTAGYVFQPTGRELIHHYLVPRALGGVFP-GFIEGVDVLSLPPRELPHIRDYG

-EVWFFFAVKP----AGETPG---AGGCWVQYGTEKPYYGSEEAVAFRRRFAYR---TKG

GSPTRWLMKEYRLNRDAADEAS-DVFVVHKVYRKPVLPPPADSSSSDSEQEGSERS----

--------------------------------------

>TraesCS2D01G334400.1

MA---L-AAAGCVFQPTGCELIGHYLIPRALGGVFP-GIIEGVDVLSLCPRALSHRRDYD

-EVWFFFAAKP----AGETPD---AGGCWEQYGQEKAYYGGEEAVAFRRRFAYR---TRD

GSQTRWRMKEYRLNRNAAGVPP-DVFVVHKVYRKPLITPPPPPDSSSSEDEGSESY----

--------------------------------------

>TraesCS2D01G334500.1

MA---PNLTAGYVFQPTGRELIHHYLVPRALGGGFP-GFIEGVDVLSLPPRELPHVRDYG

-EVWFFFAAKP----AGETPG---AGGCWVQYGTEKAYYGGEEAVAFRR-----------

--------------RDAADEAA-DVFVVHKVYRKPVLPPPADSSSSDSEEEGSERS----

--------------------------------------

>TraesCS2D01G334600.1

MA---L-AAAGCVFQPTGRELIGHYLIPRALGGVFP-GIIEGVDVLSLRPRALSHRRDYG

-EVWFFFAAKP----AGETPD---AGGCWEQYGQEKAYYGGEEAVAFRRRFAYR---TRD

GSQTRWRMKEYRLNRNAAGVPP-DVFVVHKVYRKPLIPPPPPPDSSSSEDEGSESY----

--------------------------------------

>TraesCS2D01G334800.1

MA-AGLTLRPGVVFRPSGRQIIVEYLGPKATTGKAP-GSVAGVDIFAASPDALPHRKQGG

-EAWYFFAAKP----ASSMP-----GGWWMQYGDAKGYEF--EVYARHRRFAF---RAAD

DEQTRWLMKEYALHKGASGAGA-NDLVVYKVFTKPVVT-PPPPARSSEAENQARKG----

--P--------------------------------N--

>TraesCS2D01G336300.1

IV-SQPGLPAGVKFDPTGLELLEH-LEGKVRAHEL-IDFIPTIGICYTHPENLPGTKMDG

-RNLHFFHKISNAYDVGQRKRRKISNSRWHKTGKSKEILNNGVIKGWKKILVLIMRSGTS

RTKTNWTMHQYHLGVDQD---KHGELVVSKVFYQSN--KDE--QPVMNEESGPFARSSPS

MA---PH--GTPPD---PQ----------------DLQ

>TraesCS2D01G336400.1

IADSQPGLPAGVKFDPTDLELLEH-LEGKVRAHVL-IDFIPTIGICYTHPENLPGIKNDG

-STCHFFHKVSNAYVVGKRKRRKISNSRWHKTGKSRSILNNGDIKGWKKILVLYIGYGGK

TEKTNWRMHQYHLGVDQD---KQEELVVSKVFYQVQSMNAG--QSLVNEEFDSFAGGSPS

AG---SH--GTPQDISLPT----------------DMQ

>TraesCS2D01G336500.1

IADSQPGLPIGVKFDPTDLELLGH-LEGKIRAHVL-IDFIPTIGICYTHPENLPGVKLDG

-IASHFFHKICNAYDVGTRKRRKISNSRWHKTGKSRHILNNGDIKGWKKIMVLR---GYK

AEKTNWTMHQYHLGVDEN---KDGELVVCKVFFQLPSEKAG--QCVMDEESDSFAVGSPF

AA---SR--GTPPDFSSLA----------------DLP

>TraesCS2D01G354700.1

MA---RSLPPGHAFEPSGHQIITQYLAPKALRGDTP-GHVAGVDVFAASPAALPTRRRHG

-EAWYFYGAQA----SGDAP-----GGCWARYGREKGYVHGAEAVAFRSRFAFHVPRGDG

GAPTPWLMKEYRLNKGAAGKA--DDCVVRKIFTKPVVAPPPPPACSSDEEDSSGLGSEED

----------GPPRRARPS-------------------

>TraesCS2D01G361500.1

MADEEDGLPPGEAFDPTPDAIVGRYLRRAL--DGLP-RQIHDADVYGAHPGFLPA----G

RPEWFFFVCRAQ--CQGGR--RRAGPGAYRLGSEARLLGGAA-----CHAFRYYEDEAEV

GRETEWRMDEY---DSAA---AA-DMVVCKLYPARGT----HEASPS--RPDMNKPATPD

AAPANVH-------KARPRRAATVRRVSRTEEYGKDFP

>TraesCS2D01G378800.1

M-EIESTLPPGFRFYPSDQELVCHYLYKKVTNERASQGTLVEVDLHAREPWELPDVAKLT

ASEWYFFSFRDRKYATGSRTNRATKTGYWKATGKDREVRSPAAVVGMRKTLVFYQGRAPN

GSKTSWVMHEFRLDSPHLDAILQEDWVLCRVFQKQKLDGEQDNARSSAGSSQAAQEASSD

SAPMNQYLGQYPQEGSSPMM---ATGGAGDEDSGEDTA

>TraesCS2D01G382800.1

M-NAFSHVPPGFRFHPTDEELVDYYLRKKVQLKRIGLDIIKDVDLYKIEPWDLQERCKIG

QNDWFFFSHKDKKYPTGTRTNRATTAGFWKATGRDKPIYVKHCLVGMRKTLVFYKGRAPN

GQKLDWIMHEYRLETNENGAPHDEGWVVCKVFKKRVATVQRMATADSNHDHAAFMASQRQ

NA---EYFMQLPQL-GMPGLPNQTHDGQTSRASAGEWR

>TraesCS2D01G460000.1

M-RVLLNLPPGYHFAPSEDELIVHYLRPKLDGAPAHLQAHHRLPGKDHR--------DFG

GGRWYFFTKRTRKYATGSRPDRTTGKGFWKATGPVREIPIRPKLVGRARTLVFYTGPD--

-EPTHWTMYEYENENAEANIDKLGEWVLCTIQKSQR-DVKKKAKGKAKGKAQSGADAWPE

TGPMQ--YNSMMYQ---PATS----------YAG----

>TraesCS2D01G462900.1

M--VEARLPPGFRFHPRDHELVLDYLCHKLSGGGGGVD-MVDVDLNKCEPWELPDAACVG

GREWYFFSRHDRKYATGQRTNRATHTGYWKATGKDRVITGDGAVVGMRKTLVFYLGRAPR

GTKTEWVMHEFRVEGRPPPSLLEEDWVLCRVFYKSTTASPTPASDESSGSLSSDLGEITD

TGPTS--MVEGSRGDPKPEWS-----------DG----

>TraesCS2D01G567000.1

M--EMPPLPPGYRFHPTDVELTLYYLKRKLLGKKLLCNAVAEVDIYKHAPWDLPAKSSMG

DLQWYFFCTRGRKYSVGHRANRSTEGGYWKATGKDRQVVYENRTVGMKRTLVFHSGKAPK

GTRTDWVMYEYRLVQGEIAGVRLDDSVLCKVHKKSGPGPKIG-EQYAEEQE-----GDAS

PGPRAEQ---VPSSLLEPAAGGHAPDGSSSQSNGLDLE

>TraesCS2D01G568000.1

MARAFSHLRPGFRFHPTDQELVGFFLRRKVLGHG---GFIPEVDLYKFEPHHLPAISFSS

KVEWYFFAPRGRKYPTGFRMVRATVKGFWKSTGKDRPVMHNGIVVGMKKTLVFHMGQAPG

GTRTDWVMHEYRLHGHRN-NHIEDTYALCRVFNKNMASPSMNASGDADEDLMQFMPTGLH

KDP--KHVQDLMQSDKDPIQVADAENDFMQSGMGVDID

>TraesCS2D01G576200.1

M-QEESCVPPGFRFHPTEEELVGYYLARKVAAQTIDLDIIQEVDLYRIEPWDLQERCGGA

SSEWYFFSFKDRKYPSGTRTNRATAAGFWKATGRDKPVMSSRGVIGMRKTLVFYRGRAPN

GRKTDWIIHEYRLQTSEHAPTQEEGWVVCRAFQKPAPNQKP-----STPDA-----SQVG

SAPQDKYFGNIPQLIESPPP-LGCGDDAVLQVTGIDWN

>TraesCS2D01G576300.1

M-EEESCVPPGFRFHPTEEELVGYYLARKVAAQKIDLDIIQEVDLYRIEPWDLQERCGGG

SSEWYFFSFKDRKYPSGTRTNRATAAGFWKATGRDKPVMSSRGVIGMRKTLVFYRGRAPN

GRKTDWIIHEYRLQTSEHAPTQEEGWVVCRAFQKPAPNQRP-----SAPDT-----GQVG

SAPQGQYFGNIPQLIESPPTTAGCGDTGYGIAAGIDWN

>TraesCS2D01G576400.1

M-QEESCVPPGFRFHPTEEELVGYYLARKVAAQKIDLDIIQEVDLYRIEPWDLQERCGGG

SSEWYFFSFKDRKYPSGTRTNRATAAGFWKATGRDKPVTSSSGVIGMRKTLVFYRGRAPN

GRKTDWIIHEYRLQTSEHAPTQEEGWVVCRAFQKPTPNQRP-----SAPEA-----GQ-G

SAPQGQYFGNIPQLIESPPTTAGGGDAGYDVAAGIDWN

>TraesCS2D01G576500.2

M-QEESCVPPGFRFHPTEEELVGYYLARKVAAHKIDLDIIQEVDLYRIEPWDLQERCGGG

SSEWYFFSFKDRKYPSGTRTNRATAAGFWKATGRDKPVTSSKGVIGMRKTLVFYRGRAPN

GRKTDWIIHEYRLQTSEHGPTQEEGWVVCRAFQKPTPNQRS-----SAPNA-----DQ-G

--PQGQYFSNIPQLIEKPPMTAGCGDARYDFAADIDWN

>TraesCS3A01G077900.1

M-QQQLELPMGFRFHPTDEEIITSYLAPKILNPAFDATAIGEVDLNKNEPWELPKKAKMG

ENEWYFYCQKDRKYPTGIRTNRATKAGYWKTTGKDKEIVNPHMLIGMKKTLVFYKGRAPS

GEKTNWVMHEYRLKINKQNMSSK-EYVVCRIFHKNAGSRLSSMVSHE-------------

---------------------GATPTDKIWSSMGTDGA

>TraesCS3A01G078400.1

MTRAGTGLPPGFRFYPTDEELIVHYLRRRAAAAPCPAAVIAEVDIYKLDPWELPSRAVFG

NDEWYFFSPRDRKYPNGVRPNRAAGSGYWKATGTDKPITAGGEVVGVKKALVFYQGRPPK

GLKTNWIMHEYRLADAHASSMRLDDWVLCRIYKKPNPQQLSPFPSPS---------ASLG

SSPVSDYAVS----DSMPPPPAGASDGEASTNDG----

>TraesCS3A01G107400.1

ISPKDLGLPAGVKFDPTDQELIEH-LEAKVEEHPL-IEFIPTIGICYTHPEKLPGVTMDG

-LSKHFFHRPSKAYTTGTRKRRKIQTERWHKTGKTRPVMANGRQKGCKKILVLYTNFHRK

PEKTNWVMHQYHLGDLEE---KEGELVVCKIFYQTQPRQCS--SASSDRGGAGGAVGSGS

SF-----LVSRPTNTVPPPLQSTCTKGETSI---QETE

>TraesCS3A01G113300.1

MA---PDTPPAFKFDPTDADIVAGYLLPRALGLP-PAHAIIEDDPASAPPWELLRRH--G

VDHAFFFGPPA----NGRRKSRTIGAGVWQGQKGSQGVTRPGLDVTYRYDLTFC---AKH

GGSTGYVMHEYEIISPPL-----PGTVLSRVNINKHPKKKR--AAAG--ER-----PSDS

GGP-----------CCLPATD----------FAGVDTD

>TraesCS3A01G157600.1

MA-RAPDWPRGLIFAPGDADLITIHLQRKSSGSSLDARYIHNADVYAAEPAALPASARSG

GREWYFFTSVRAQSSRDTRRCRAVGAGTWHSEKARCVVLDGGAAVGYRQSFTYE------

-PKNGWLMLEFSQQEPRRG---EAMPVLCKIYQKRRAGRSASKTISSGSKRKAAAAGSSA

EAPRIDHDWPMPG-FVSPATSLTSSYDTTFLLPGQDWA

>TraesCS3A01G162900.1

M-ERGCELPPGFRFQPTDQEIIVCYLKRKVASAASAVSIIADVDIYKFDPWELPDKAQFG

EGEWFFFSPRDRKYPNGARPNRTAGSGYWKATGTDKPILAAGRCLGVKKALVFYQGRSPR

GTKTEWVMHEYRLLHADGDSMRLDDWVLCRVRKKGVAVAPDMDGNPATEQA-----STTG

AA---QYAAGVHDGESAPEVQRDLSFHAMDDCMGTDDD

>TraesCS3A01G176500.1

M-ERDMALPPGFGFHPKDTELVAHYLKKKILGQKIEYDIIPEVDIYKHEPWDLPAKCNVP

DNKWHFFAARDRKYPNGARSNRATVAGYWKSTGKDRAIKVDKRTIGTKKTLVFHEGRPPT

GKRTEWIMHEYYIDENECCPDMKDAFVLCKVTKRIDWTSENPQPQQA--NVAAILAAASS

TSPEQDY---MEEFNNGPNLYVTSSSESINQNNGLDLM

>TraesCS3A01G245900.2

SAQQDPGLPAGVKFDPTDQELLEH-LEGKAPDHPL-IEFIPAIGICYTHPERLPGVGKDG

-LIRHFFHRPSKAYTTGTRKRRKVHTDRWHKTGKTRPVFTDGKLKGYKKILVLYTNYQRK

PEKTNWVMHQYHLGSDEE---KDGELVVSKVFYQTQPRQCG---------------TAGN

-----HHGYSSQA---APPNN-----------------

>TraesCS3A01G247500.1

MARSR---APGLDAHPCEQELIAAYLGPRVTDGDMSCKFIHEGDVYAAHPEDLAAVSSNG

DEAWYFFTAVRAKG--GGRRGRTVQEGCWHSEAGSKPVVSAHGLLGHRQNFSFVTKEDGV

RVRSGWLMVELGLHGD--G---QDEVTLCKVYFSPRAAKNNKEPAASARKRKADADAGEQ

ESPAA-----APPSPVVPNED-------------NDLP

>TraesCS3A01G269900.1

MA-RRAA-RPDFASHPSDLELISTYLIPWVTGER-PWKFVHEADVYAATPQDLPATASDG

QESWYFFTSLRAKSRRGQRKARTVGHGCWHSERAAKPLF-AGRQIGYRQAFSFATKEDGR

LVRSGWLMAEIGLND-ASA---EEELVLCKVYRSPRVGTGQRSTAPAASEEDSTSSTSPS

PSPALQRPVEPPAATLGPAAGTSGRNKNTSAEDGTDLD

>TraesCS3A01G336300.1

MAQQQPGLVPGVRFVPTDQELILCYLRSKLRGDPPPTSLVRDDDVYAEHPEYLTTRL--G

EDYWYVFTQRSRKYAKGGRPSRSTGTGRWKSVGKNTPVTYGKATIGFRNSLAYEDSKKKR

IEKTEWKMTEFVDVDSNRNFMLLNDWVLCRITRKPEKKKGEEEASPADEEEEHEPSTSPD

GAPKTDHPAYHPQPTDLPSSSRASGDDAGKKSSGPDGQ

>TraesCS3A01G339600.1

MKEAELNLPPGFRFHPTDDELVVHYLCRKVAGQPQPVPIIAEVDLYKFNPWDLPERALFG

SREWYFFTPRDRKYPNGSRPNRSAGTGYWKATGADKPVAPRERTVGIKKALVFYSGRAPR

GVKTDWIMHEYRIAEADRGSLKLDEWVLCRLYNKKNNWEKLKVEQDAVE-------AGPN

DS---QH--------------QQQAR--MVTFTGLSMD

>TraesCS3A01G375400.1

MA---DGLPPGYRFYPTEEELICFYLRNKLDGSRGDIEVIPVVDVYSVDPLQLSEIHERG

GEPWFYFCARQEREARGGRPSRTTPSGYWKAAGTPGVVYSADRPIGLRKTMVFYRGRAPS

GTKTKWKMNEYRAFQHEHPPQLRSEFSLCRLYTKSGTLRQFDRAAAA--DIPGPSTASPD

DGP------------------EDLMEGAGGDPYGATLA

>TraesCS3A01G377700.2

PGPKDVGFPAGVKFDPTDQELIEH-LESKV--HSL-IDFIPTIGICYTHPEKLPGVTRNG

-QSKHFFHRPSKAYTTGTRKRRKIHAERWHKTGKTRPLMVGGQHKGCKKILVLYINFTRK

AEKTNWVMHQYHLGDLED---KEGELIVSKVFYQTQPRQ-----------------TNGN

---------------------SASTKEGTST--TEDEQ

>TraesCS3A01G387500.1

IA-PPPEPAPGFSFCPTDSELVSFYLRPRISGQPLTKQFFHEADVYATDPASLPGRA--Q

SKNWYFFSLVKPRSAQDVRKCRIVGKGTWKQERGNDVV-GAEHAVGRLEKFTYTPSPKED

KKPPEWLMMEFSVGQE--GGEPRPVLCLCKIYQSPRFLKFASKNSVSARKRKTPDDESLA

PNPPC---------------------------------

>TraesCS3A01G403300.1

MSHEDDLVMPGFRFHPTEEELIEFYLRRKVEGKRFNVELITFLDLYRYDPWELPALAAIG

EKEWFFYVPRDRKYRNGDRPNRVTASGYWKATGADRMIRAENRSIGLKKTLVFYSGKAPK

GVRSSWIMNEYRLPTADTDRYHKTEISLCRVYKRTGIDDGRGHPSSARSTMPSRRGSSST

PSPTDTHAGEHQQESKSTNTFNAASMGVASA---ID--

>TraesCS3A01G406000.1

MSGQELNLPPGFRFHPTDEELVTHYLCRRCAGAPIAVPIITEIDLYKFDPWQLPKMALYG

EKEWYFFSPRDRKYPNGSRPNRAAGSGYWKATGADKPVGT--KPLAIKKALVFYAGKAPK

GEKTNWIMHEYRLADVDRNSLRLDDWVLCRIYNKKGGMEKPAAVDR---KPAAMGGSSPP

QKPMGAQYYDRPSD-SMPRL-PEFPAGEVQSAAGLE--

>TraesCS3A01G438900.1

MAAGQGGLPVGFRFRPTDEELLLHYLRRKALSCPLPADIIPVADLARLHPSDLPGEA---

GERYFFHLPATGCWRKGGGAGRAGGSGVWRASGKERLVVAPRRPIGAKRTLVFC---RPG

GARTGWAMHEYRLLPAGLSLHAAKDWVVCRVFKKATPARHGTAGRRG--DADADMTASPS

--PSS---------------------------SGEDDD

>TraesCS3A01G471300.1

MA-AERGLTPGYKFMPTDEEVIAFYLIPRLRGQPLPLDVIIDEDPRSAPPWKLFERN--G

VEHAFFSAS--GEYKSAKRKVRAYAGGTWVKVGNKGKLR-GGETFAWVYRMNYQLGVGRR

TGSTGWVMLEYSIKAPADA-----SIKVCKISFSGYGQKRKRVSARASEDEDAGGETAQQ

SSPRNQF-------GTVPPLT----------EAGFDVE

>TraesCS3A01G485400.1

M-E----L--GESFEPTEDELVLHFLRPRLRGFA-PVGAVVEADPCAAPPWELLERH--G

RGQGYFFAARRR----GKR--RTPGGGAWMHSGNKEDRRS--TELGVMTRYCFYRGGAQQ

GRSTGWVMSEYEITDPRCDGEEDQYWVLCHVRRSIREPRSRRR-----------------

--------------------------------------

>TraesCS3A01G485500.1

M-Q----L--GESFEPTEDELVLHFLRPQLRGFA-PVGAVVEADPCAATPWELLARH--G

RGHGYFFAARRR----GKR--RTPGGGAWMHSGNREDRRS--TELGVMTRYCFYRDW-AQ

GRSTGWVMSEYEITDPRCDGEEDHYWVLCHVRRSVRKPRSRRP-----------------

--------------------------------------

>TraesCS3A01G486500.1

M-E----L--GESFEPTEDELVLHFLRPQLRGFA-PVGAVVEADPCAAPPWDLLERH--G

RGHGYFFHARRR----GKR--RTPGGGTWMHSGNREDRRS--TELGVMTRYCFYRDS-AQ

GRSTGWVMSEYEITDPRCDGEEEEYWVLCHVRRSTRKPRSRRR-----------------

--------------------------------------

>TraesCS3B01G092800.1

----MLELPPGFRFHPTDEEIITSYLAPKILNPAFNATAIGEVDLNKNEPWELPNKAKMG

ENEWYFYCQKDRKYPTGIRTNRATKAGYWKATGKDKEIVNPHMLMGMKKTLVFYKGRAPS

GEKTNWVMHEYRLEIGKQNVSSK-EYVVCRIFHKNTGSGVSSMVSHE-------------

--------------GTGPGN-GATTTDKILSSMRTGGA

>TraesCS3B01G092900.3

M-QQQLKLPSGFRFHPTDVEIITAYLVPKVLKKPFDTREVGEVDLNKHDPWELPEMANMG

EKEWYFFSQKDHKYPTGIRTNRATTAGYWKATGKDKEIFHPPSLIGTKKTLVFYKGRAHK

GEKTNWIMHEYRLERGKQNASSKEEYVVCRIFHKSIGLKKVVMSSFAVERQHGFLESSLA

GAPMNHHDMIVDQGRVEPRSETTTTSDEIMSNMGMDGM

>TraesCS3B01G093300.1

MTRAGTGLPPGFRFYPTDEELIVHYLRRRAAAAPCPAAVIAEVDIYKLDPWELPSRAVFG

NDEWYFFSPRDRKYPNGVRPNRAAGSGYWKATGTDKPITAGGEVVGVKKALVFYQGRPPK

GLKTNWIMHEYRLADAHASSMRLDDWVLCRIYKKPNPQQLSPFPSPS---------ASLG

SSPISDYAVS----DSMPPPPTGAGDGEASTNDG----

>TraesCS3B01G126200.3

ISPKDLGLPAGVKFDPTDQELIEH-LEAKVEEHPL-IEFIPTIGICYTHPEKLPGVTMDG

-LSKHFFHRPSKAYTTGTRKRRKIQTERWHKTGKTRPVMANGRQKGCKKILVLYTNFHRK

PEKTNWVMHQYHLGDLEE---KEGELVVCKIFYQTQPRQCS--SASSDRGGAGGAVGSGS

SF-----LVSRPTNTVPPPLQSTCTKGEASI---QETE

>TraesCS3B01G184300.1

MA-RAPDWPRGLIFAPGDANLITIHLQRKISGSSLDARYIHNADVYAAEPAALSASARSG

GREWYFFTSVRAQSSRDTRRCRAVGAGTWHSEKARCVVLDGDGIVGYRQSFTYE------

-PKNGWLMLEFSQQEPRRG---EAMPVLCKIYQKRRAGRSASKTISSGSKRKAAAAGSSA

EAPRIDHDWPMPMPFVSPATSLTSSYDTTFLLPGQDWA

>TraesCS3B01G184500.1

MA-RAPDWPRGLIFAPGDANLITIHLQRKISGSSLDARYIHNADVYAAEPAALSASARSG

GREWYFFTSVRAQSSRDTRRCRAVGAGTWHSEKARCVVLDGDGIVGYRQSFTYE------

-PKNGWLMLEFSQQEPRRG---EAMPVLCKIYQKRRACRSASKTISSGSKRKAAAAGSSA

EAPRIDHDWPMPMPFVSPATSLTSSYDTTFLLPGQDWA

>TraesCS3B01G194000.1

M-ERGCELPPGFRFQPTDQEIIVCYLKRKVASAASAVSIIADVDIYKFDPWELPDKAQFG

EGEWFFFSPRDRKYPNGARPNRTAGSGYWKATGTDKPILAAGRCLGVKKALVFYQGRSPR

GTKTEWVMHEYRLLHADADSMRLDDWVLCRVRKKGVAVAPDMDGNPAAEQA-----STTA

AA---QYAAGVHDGESAPEVHRDLSFHAMDDCMGTDGD

>TraesCS3B01G208300.2

M-ERDMALPPGFGFHPKDTELVAHYLKKKILGQKIEYDIIPEVDIYKHEPWDLPAKCNVP

DNKWHFFAARDRKYPNGARSNRATVAGYWKSTGKDRAIKVDKRTIGTKKTLVFHEGRPPT

GKRTEWIMHEYYIDENECCPDMKDAFVLCKVTKRIDWTSENPQPQQA--NVAAILAAASS

TSPEQDY---MDEFNNGPNLYVTSSSESINQNNGLDLM

>TraesCS3B01G271900.1

MARSR---APGLDAHPCEQELIAAYLGPRVTEGDMSCKFIHEGDVYAAHPEDLAAVASNG

DEAWYFFTAVRAKG--GGRRGRTVQEGCWHSEAGSKPEVSAHGLLGHRQNFSFVTKEDGV

RVRSGWLMVELGLHGD--G---QDEVTLCKVYFSPRAAKNNKKPAASARKRKADAGAGKQ

ESPAA-----APPSPVVPNED-------------HGLP

>TraesCS3B01G273600.1

SAQQDPGLPAGVKFDPTDQELLEH-LEGKALDHPL-IEFIPAIGICYTHPERLPGVGKDG

-LIRHFFHRPSKAYTTGTRKRRKVHTDRWHKTGKTRPVFTEGKLKGYKKILVLYTNYQRK

PEKTNWVMHQYHLGSDEE---KDGELVVSKVFYQTQPRQCG---------------TAGN

-----HHGYSSQA---APPNN-----------------

>TraesCS3B01G303800.1

MA-RRAA-RPDFASHPSDLELISTYLIPWVTGER-PWKFIHEADVYAATPQDLPATASDG

QESWYFFTTLRAKSRRGQRKSRTVGHGCWHSERAAKPLF-AGRQIGYRQAFSFATKEDGR

LVRSGWLMAEIGLND-ASA---EEELVLCKVYRSPRVGTGQRSTAPAASEEDSSSSTSPS

PSPALQRPVEPPAATLGPAPGTSGRKKSTSAEDGTDLD

>TraesCS3B01G367100.1

LAQQQSGLVPGVRFVPTDQELILCYLRSKLRGDPPPTTLVHDDDVYAEHPQILTERL--G

EDYWYVFTQRNRKYAKGGRPSRNTGTGRWKSVGKNMPVTYGKATIGFRNSLAYEDSKKKR

IEKTEWKMAEFVDVDSNMNFMLLNEWVLCRITRKPEKKKGEEEASPADEEEEHEPSTSPD

GAPQTGHPA-APQPTDLPSSTRASGDDAGKKSSGPDGQ

>TraesCS3B01G371200.1

MKEAELNLPPGFRFHPTDDELVVHYLCRKVAGQPQPVPIIAEVDLYKFNPWDLPERALFG

SREWYFFTPRDRKYPNGSRPNRSAGTGYWKATGADKPVAPKERTVGIKKALVFYSGRAPR

GVKTDWIMHEYRIAEADRGSLKLDEWVLCRLYNKKNNWEKVKVEQDAVE-------AGPN

DS---QR--------------QGQPRGGMVTFTGLSMD

>TraesCS3B01G407600.1

MA---DGLPPGYRFYPTEEELICFYLRNKLDGSRGDIEVIPVVDVYSVDPLQLSEIHERG

GEPWFYFCARQEREARGGRPSRTTPSGYWKAAGTPGVVYSADRPIGLRKTMVFYRGRAPS

GTKTKWKMNEYRAFQHEHPPQLRSEFSLCRLYTKSGTLRQFDRAAAA--DIPGPSTASPG

DGP------------------EELMEGADGDPYGATLA

>TraesCS3B01G410500.1

PGPKDVGFPAGVKFDPTDQELIEH-LESKV--HSL-IDFIPTIGICYTHPEKLPGVTRNG

-QSKHFFHRPSKAYTTGTRKRRKIHAERWHKTGKTRPLMVDGRHKGCKKILVLYSNFARK

PEKTNWVMHQYHLGDLED---KEGELIVSKVFYQTQPRQ-----------------TNGD

---------------------SASTKEGTST--TEDVQ

>TraesCS3B01G421300.1

IA-PPPEPAPGFAFCPTDSELVSFYLRPRISGQPLAKQFFHEADVYATDPASLPGRA--Q

SKNWYFFSLVKPKSAQNSRKSRTVGKGTWKQERGNDVV-DAEHAVGRFEKFTYTPNPKED

KKPPEWLMMEFSVGQE--GGQPRPVLCLCKIYQSPRFLKSASKNSASARKRKTPDDESSA

PNPPC---------------------------------

>TraesCS3B01G436900.1

MSHEDDLVMPGFRFHPTEEELIEFYLRRKVEGKRFNVELITFLDLYRYDPWELPALAAIG

EKEWFFYVPRDRKYRNGDRPNRVTASGYWKATGADRMIRAESRPIGLKKTLVFYSGKAPK

GVRSSWIMNEYRLPTADTDRYHKTEISLCRVYKRTGIDDGRGHPSSARSTMPSRRGSSST

PSPTDTHAGEHQQESKSTNTFNAASMGVASA---ID--

>TraesCS3B01G439600.1

MSGQELNLPPGFRFHPTDEELVTHYLCRRCAGAPIAVPIITEIDLYKFDPWQLPKMALYG

EKEWYFFSPRDRKYPNGSRPNRAAGSGYWKATGADKPVGT--KPLAIKKALVFYAGKAPK

GEKTNWIMHEYRLADVDRNSLRLDDWVLCRIYNKKGGLEKPASVDR---KPAAMGGSSPQ

QKPMGVQYYDRPSD-SMPRL-PDFPAGEVQSAAGLE--

>TraesCS3B01G446100.1

VA-PPPQPAPGFAFCPTDRELVSFYLRPRISGQPLAKHYFHEADVYATDPASLPGKA--Q

SKNWYFFSLVKPRSAQDSRKCRIVGKGTWKQERGNDVA-GAERAIGRLERFTYTPSPKED

KKPPEWLMTEFSVDQH--GGQPTPVLCLCKIYQSPRFLKSASKNSASARKRKSPADESSA

PNPPC---------------------------------

>TraesCS3B01G472800.1

MAAGQGGLPIGFRFRPTDEELLLHYLRRKALSCPLPADIIPVADLARLHPWDLPGEA---

GERYFFHLPATGCWRKGGGTGRAGGSGVWRASGKERLVVAPRRPIGAKRTLVFC---RPG

GARTGWAMHEYRLLPAGLSLHAAKDWVVCRVFKKATPARRDTAGRRS--RGDADMTASPS

--PSS--------------------------RSGEDDD

>TraesCS3B01G533100.1

M-E----L--GIEFEPTEDELVLHFLRPQLRGFA-PVGAVVEADPCASPPWELLARH--G

RGHGYFFAARRR----GKR--RTPGGGAWMHSGNKEDRRS--TELGVMTRYCFYRGGAQQ

GRSTGWVMSEYEITDPRCDGEEDQYWVLCHVRRSIREPRSRRR-----------------

--------------------------------------

>TraesCS3B01G533200.1

M---------GIEFEPTEDELVLHFLRPQLRGFA-PVGAVVEADPCAATPWELLARH--G

RGHGYFFAARRR----GKR--RTPGGGAWMHSSNREDRRS--TELGVMTRYCFYRDW-AQ

GRSTGWVMSEYEITDPRCDGEEDHYWVLCHVRRSVRKPRSRRP-----------------

--------------------------------------

>TraesCS3B01G534500.1

M-Q----L--GIEFEPTEDELVLHFXXXXXXXXX-XXXXXXXXXXXXXXPWELLARH--G

RGHGYFFAARRR----GKR--RTPGGGTWMHSGNREDRCS--TELGVMTRYCLYRDG-AQ

GRSTGWVMSEYEITDPRCDGDEDQYWVLCHVRRSIRKPRSRRR-----------------

--------------------------------------

>TraesCS3B01G599700.1

MN-----APPGFRFTPTREELIRYYLDPWVADKT-PVEIICVADIYGEDPAALTSRHRFG

DGNWYFLCVS-RKGRAGGRTSRTVGGGTWHGYGKRVAVPEA----GHRQAFEYL---DAR

GRKTAWLMEEFGTVLPAAG-----VRVLCRVHQTTRTVADDERSSKASSRRRITADAKDE

DSP--EMTKNLPSPKNLPK--GAANKEATPN----DAE

>TraesCS3B01G599900.1

MD-----APPGFRFTPTQEELILYYLDPWVADKT-PDEIVCVADVYGEDPAALTSRHRFG

DGNWYFLCVA-RKGRAGGRASRTVGGGTWHGSGKRVAVAGA----GH-------------

-----------------------------RVHQTTRTAEDEERSSKASSRRRGEHDAKDE

ESP--QMTKNLPSPKNLPK--GAANKEATPN----DAE

>TraesCS3B01G600000.1

MN-----APPGFRFTPTREELIGYYLNPWVADKT-PVEIVCVADIYGEDPAALTSRHRFG

DGNWYFLCVA-RKGAVGGRASRAVGGGTWHGYGKRVAVAEA----GHRQAFEYL---DAR

GRKTAWLMEEIGTVLPVAG-----VRVLCR-------------SSKASSRRRQ---AKDE

ESP--QMTKNLPSPKNLPK--GTANKEATPN----DAE

>TraesCS3D01G078500.1

M-QQQLELPPGYRFHPTDEEIITSYLAPKILNPAFDATAIGEVDVNKNEPWELPKKAKMG

ENEWYFYCQKDRKYPTGIRTNQATKAGYWKTTGKDKEIVNPHMLIGMKKTLVFYKGRAPS

GEKTNWVMHEYRLEIGKQNASSK-EYVVCRIFHKNTGSGLSSMVSHE-------------

--------------GTRPGN-SGTTTHKISSSMGTDGA

>TraesCS3D01G078900.1

MTRAGTGLPPGFRFYPTDEELIVHYLRRRAAAAPCPAAVIAEVDIYKLDPWELPSRAVFG

NDEWYFFSPRDRKYPNGVRPNRAAGSGYWKATGTDKPITAGGEVVGVKKALVFYQGRPPK

GLKTNWIMHEYRLADAHASSMRLDDWVLCRIYKKPNPQQLSPFPSPS---------ASLG

SSPVSDYAVS----DSMPPPPAGAGDGEASTNDG----

>TraesCS3D01G109400.2

ISPKDLGLPAGVKFDPTDQELIEH-LEAKVEEHPL-IEFIPTIGICYTHPEKLPGVTMDG

-LSKHFFHRPSKAYTTGTRKRRKIQTERWHKTGKTRPVMANGRQKGCKKILVLYTNFHRK

PEKTNWVMHQYHLGDLEE---KEGELVVCKIFYQTQPRQCS--SASSDRGGAGGAVGSGS

SF-----LISRPTNTVPPPLQSTCTKGETSI---QETE

>TraesCS3D01G165200.1

MA-RAPDWPRGLVFAPGDADLITIHLQRKISGSSLDARYIHNADVYGAEPAALSASARSG

GREWYFFTSVRAQSSRDTRRCRAVGVGTWHSEKARCVVLDGGAVVGYRQSFTYE------

-PKNGWLMLEFSQQEPRRG---EAMPILCKIYQKRRAGRSASKTISAESKRKAAAAGSSA

EAPRIDHDWPMPA-FVSPATSLTSSYDTTFLLPGQHWA

>TraesCS3D01G170000.1

M-ERGCELPPGFRFQPTDQEIIVCYLKRKVASAASAVSIIADVDIYKFDPWELPDKAQFG

EGEWFFFSPRDRKYPNGARPNRTAGSGYWKATGTDKPILAAGRCLGVKKALVFYQGRSPR

GTKTEWVMHEYRLLHADADSMRLDDWVLCRVRKKGVAVAPDMDGNPAAEQA-----STTA

AA---QYAAGVHDGESAPEVQRDLSFHAMDDCMGTDGD

>TraesCS3D01G183900.2

M-ERDMALPPGFGFHPKDTELVSHYLKKKILGQKIEYDIIPEVDIYKHEPWDLPAKCNVP

DNKWHFFAARDRKYPNGARSNRATVAGYWKSTGKDRAIKVDKRTIGTKKTLVFHEGRPPT

GKRTEWIMHEYYIDENECCPDMKDAFVLCKVTKRIDWTSENPQPQQA--NVAAILAAASS

TSPEQDY---MDEFNNGPNLYVTSSSESINQNNGLDLM

>TraesCS3D01G243900.1

MARSR---APGLDAHPCEQELIAAYLGPRVTDGDMSCKFIHEGDVYAAHPEDLAAVASNG

DEAWYFFTAVRAKG--GGRRGRTVQEGCWHSEAGSKPVVSAHGLLGHRQNFSFVTKEDGV

RVRSGWLMVELGLHGD--G---QDEVTLCKVYFSPRAAKNNKKPAASARKRKADAGAGKQ

ESPAA-----APPSPVVPSKD-------------NDLP

>TraesCS3D01G245500.1

SAQQDPGLPAGVKFDPTDQELLEH-LEGKAPDHPL-IEFIPAIGICYTHPERLPGVGKDG

-LIRHFFHRPSKAYTTGTRKRRKVHTDRWHKTGKTRPVFTDGKLKGYKKILVLYTNYQRK

PEKTNWVMHQYHLGSDEE---KDGELVVSKVFYQTQPRQCG---------------TAGN

-----HHGYGSQV---APPNN-----------------

>TraesCS3D01G269200.1

MA------PVATPRHPSEPELIKSYLLGRITK--LSWEFIHDADVYTADPASLPASD---

EKVWYFFSPVRTKNVRGQRKARTLGAGCWHSEAGTKAVEDGDHRVGCRQFFSFVYKHNGQ

RIRTGWLMVELRLDREQVS--DDPDLVLCKIYFTPRVSSSTAHATAADEKRKNDDQASSN

SSPES---------FVLPSSD-------------GDCS

>TraesCS3D01G269600.1

MA-RRAA-RPDFASHPSDLELISTYLIPWVTGER-PWKFVHEADVYAATPQDLPATASDG

QESWYFFTTLRAKSRRGQRKARTVGHGCWHSERAAKPLF-AGRQIGYRQAFSFATKEDGR

LVRSGWLMAEIGLND-ASA---EEELVLCKVYRSPRVGTGQRSTAPAASEEDSTSSTSPS

PSPALQRPVEPPAATLGPAPGTSGRKKNSSAEDGTDLD

>TraesCS3D01G329300.1

MAQQQPGLVPGVRFVPTDQELILFYLRSKLRGDPPPTSLVRDDDVYAEHPQILTTRL--G

EDYWYVFTQRSRKYAKGGRPSRSTGTGRWKSVGKNTPVTYGKATIGFRNSLAYEDSKKKR

IEKTEWKMMEFVDVDSNRNFMLLNDWVLCRITRKPEKKKGEEEASPADEEEEHEPSTSPD

GAPQTDHPA-APQPTDPPSSARASGDDAGKKSSGQDGQ

>TraesCS3D01G333100.1

MKEAELNLPPGFRFHPTDDELVVHYLCRKVAGQPQPVPIIAEVDLYKFNPWDLPERALFG

SREWYFFTPRDRKYPNGSRPNRSAGTGYWKATGADKPVAPKERTVGIKKALVFYSGRAPR

GVKTDWIMHEYRIAEADRGSLKLDEWVLCRLYNKKNNWEKLKVEQDAVE-------AGPN

DS---QH--------------QGQPRGGMVTFTGLSMD

>TraesCS3D01G370800.1

PGP-DVGFPAGVKFDPTDQELIEH-LESKM--HSL-IDFIPTIGICYTHPEKLPGVTRNG

-QSKHFFHRPSKAYTTGTRKRRKIHAERWHKTGKTRPLMVDGRHKGCKKILVLYSNFARK

PEKTNWVMHQYHLGDLED---KEGELIVSKVFYQTQPRQ-----------------TNGD

---------------------SASTKEGTST--TEDMQ

>TraesCS3D01G381900.1

IA-PPPEPAPGFAFCPTDSELVSFYLRPRISGQPLTSQFFHEADVYATDPASLPGRA--Q

SKNWYFFSLVKPKSAQNSRKSRIVGKGTWKQERGNDVV-DGEHAVGRFEKFTYTPNPKED

KKPPEWLMMEFSVGQE--GGQPGPVLCLCKIYQSPRFLKSASKNSASARKRKTPDDESPA

PNPPC---------------------------------

>TraesCS3D01G398200.1

MSHEDDLVMPGFRFHPTEEELIEFYLRRKVEGKRFNVELITFLDLYRYDPWELPALAAIG

EKEWFFYVPRDRKYRNGDRPNRVTASGYWKATGADRMIRAENRSIGLKKTLVFYSGKAPK

GVRSSWIMNEYRLPTADTDRYHKTEISLCRVYKRTGIDDGRGHPSSARSTMPSRRGSSST

PSPTDTHTGDHQQESKSTNTFNAASMGVASA---ID--

>TraesCS3D01G401200.1

MSGQELNLPPGFRFHPTDEELVTHYLCRRCAGAPIAVPIITEIDLYKFDPWQLPKMALYG

EKEWYFFSPRDRKYPNGSRPNRAAGSGYWKATGADKPVGT--KPLAIKKALVFYAGKAPK

GEKTNWIMHEYRLADVDRNSLRLDDWVLCRIYNKKGGMEKPASVDR---KPAAMGGSSPQ

QKPMGVQYYDRPSD-SMPRL-PEFPAGEVQSAAGLE--

>TraesCS3D01G406800.1

VA-PPPQPAPGFAFCPTDSELVSFYLRPRISGQPLAKRFFHEADVYATDPASLPGRA--Q

SKNWYFFSLVKPRSAQDSRKCRIVGKGTWKQERGNDVV-GAKRAVGRLERFTYTPNPKED

KKPPEWLMTEFSVDQH--GGQPRPVLCLCKIYQSPRFLKSASKNSASARKRKAAADESPA

PNPPC---------------------------------

>TraesCS3D01G431700.1

MAAGQGGLPIGFRFRPTDEELLLHYLRRKALSCPLPADIIPVADLARLHPWDLPGEA---

GERYFFHLPATGCWRKGGGAGRAGGSGVWRASGKERLVVAPRRPIGAKRTLVFC---RPG

GARTGWAMHEYRLLPAGLSLHAAKDWVVCRVFKKATPARHGTAGRRG--DADADMTASPS

--PSS--------------------------RSGEDED

>TraesCS3D01G467000.1

MA-AEWGLTPGYKFMPADEEVIEFYLIPRLRGQPLPLDVIIDEDPRSAPPWKLFERN--G

VEHAFFFTS--GEYKSAKRKVRACAGGTWVKVGNKGKLR-GGETFAWVYRMNYQWGAGRR

TGSTGWVMLEYSIVAPADA-----SIKVCKISFSGYGQKRQRVSGRASEDEDAGGEAAQQ

SSPRNQF-------GTVPPLT----------EAGFDVE

>TraesCS3D01G480700.1

M-E----L--GELFEPTEDELVLHFLRPQLRGFA-PVGVVVEADPCAAPPWELLARH--G

RGHGYFFAARRR----GKR--RTPGGGAWMHSGNKEDRRS--TELGVMTRYCFYRGGAQQ

GRSTGWVMSEYEITDPRCDGQEDQYWVLCHVRRSIREPRSRRR-----------------

--------------------------------------

>TraesCS3D01G480900.1

M-Q----L--GESFEPTEDELVLHFLRPQLRGFA-PVGAVVEADPCAATPWELLARH--G

RGHGYFFAARRR----GKR--RTPGGGAWMHSGNREDRRS--TELGVMTRYCFYRDW-AQ

GRSTGWVMSEYEITDPRCDGEEDHYWVLCHVRRSIRKPRSRRP-----------------

--------------------------------------

>TraesCS3D01G481900.1

M-E----L--GIEFEPTEDELVLHFLRPQLRGFA-PVGAVVEADPCAAPPWELLERH--G

RGHGYFFAARRR----GKR--RTPGGGTWMHSGNREDRRS--TELGVMTRYCFYRDG-AQ

GRSTGWVMSEYEITDPRCDGEEDHYWVLCHVRRSTRKPRSRRR-----------------

--------------------------------------

>TraesCS3D01G535500.1

MD-----APPGFRFTPTPEELINFYLDPWVADKT-PVEIVCVADVYGEDPAALTSRHRFG

DGNWYFLCMA-RKGAVGGRASRAVGGGTWHGYGKRVTVAGA----GH-------------

-----------------------------RVHQTTRTAADDERSSKVSSRRRREHDTKDE

ESP--EMTKNLPSPKNLPD--GAANKEAIPN----DAE

>TraesCS4A01G065000.5

MAEQQPGLPRGVEFNPSDSDLLWH-LAAEMNGHPF-IEFIKSGRFGCTHPRDMPGMRQDG

-HALYFFHKKINDN-N--ENDKDI---SWQKNGPSRSIILDGGLRGCKETFALYTFK-NS

PRRTDWKLHQYYIKNTME---NEGELVLSKVFYKSQKSHCE--AAKASAQDNSVTDATES

QAPSENHEDSLPSLSMEPIVKTDSIETALEEEWGRDLQ

>TraesCS4A01G130600.1

MSAAGLDLPPGFRFHPTDEEIISHYLTPKALDHRFCSGVIGEVDLNKCEPWHLPGKAKMG

EKEWYFFCHKDRKYPTGTRTNRATESGYWKATGKDKEIFRGRLLVGMKKTLVFYLGRAPR

GEKTGWVMHEFRLEGK--PRSAKDEWAVSKVFNKELTATNGAMAAADAGERVSSFGGDVD

TGPANRYSPSRETGSTDPNAAAATPSSQNQE---LDEF

>TraesCS4A01G131000.1

M-SDPAMLPPGFRFHPTDEELILHYLRNRAAESPCPVSIIADVDIYKFDPWALPSKASYG

DREWYFFTPRDRKYPNGVRPNRAAGSGYWKATGTDKPIRCSAESVGVKKALVFYKGRPPK

GIKTNWIMHEYRLAAADAASMRLDDWVLCRIYKKTSQASPMAVPPLSDHELDEPSGSSAG

AAPISRHQHA----LGHPIMSGAAGKRKRPSEDG-DRN

>TraesCS4A01G213100.1

M-ESPPRLPPGVYFSPTREESVAL-LDRWIAG---------------------------G

-----------KECP-GARAGRKVTGGYWREDRCKAE---GG---GFKSYFVFFLGPPSR

KEKTPWVAQEFTSAKDDGG--KKGVPALYMLYVSPRATDDELR---GGE-------VGPD

GSP--HH------------------------VLG----

>TraesCS4A01G219700.1

MDDGALQLPPGFRFHPTDEELVMYYLCRKCGGLPIAAPVIAEVDLYKFEPWRLPEKAAGG

AKEWYFFSPRDRKYPNGSRPNRAAGTGYWKATGADKPVGS--RPVAIKKALVFYAGKPPK

GVKTNWIMHEYRLADVDRNALRLDDWVLCRIYNKKGVIERYDTADS---DVADVKPPRPG

AGPMKQQSADRDSNHSMPRL-LSSPSPDFPSDDGIDGS

>TraesCS4A01G242700.1

---MEEGLPPGFRFHPTDEELITYYLSRKVSDFSFATRAIADADLNKCEPWDLPSKASMG

EKEWYFFSMRDRKYPTGIRTNRATESGYWKTTGKDKEIFHGGRLVGMKKTLVFYGGRAPK

GEKTSWVMHEYRIQNKFPYKPNKEEWVVCRVFKKSQIVKMRPHDSPE--EAHASLGSMLG

SGPAA--NPAMQKA-LAPFAGDALFGNAMAK---VDME

>TraesCS4A01G419900.1

MSNGQSVVPPGFRFHPTEEELLTYYLARKVASQRIDRDVIPNVDLNKLEPWDIQELCRVG

QNDWYLFSHKDKKYPTGTRTNRATAAGFWKATGRDKAIYSVAGRIGMRKTLVFYKARAPH

GHKSDWIMHEYRLHHAV-SSTQEDGWVICRVFKKKNIAVQHQDGQNDSSNKLVGAGSQTK

SS---KHTASCRQE-TKPTN--ACHNGTSTLEYNRDWD

>TraesCS4A01G420000.1

MCNGQSVVPPGFRFHPTEEELLTYYLAKKVASQRIDLDVIPDIDLNKLEPWDIQERCRIG

QNDWYLFSHKDKKYPTGTRTNRATAAGFWKATGRDKAIYSAAGRIGMRKTLVFYKGRAPR

GHKSDWIMHEYRLDDAV-SSAQEDGWVICRVFRKKNIVVQHQNGG--------GAGSQSN

SS-HQQHTTSCKQE-TKPTN--ACNNGTGTLEYDRDWD

>TraesCS4A01G420100.1

MSNGQSVVPPGFRFHPTEEELLTYYLAKKVASQRIDLDVIPDVDLNKLEPWDIQERCRIG

QNDWYLFSHKDKKYPTGTRTNRATTAGFWKATGRDKAIYSAAGRIGMRKTLVYYKGRAPH

GHKSDWIMHEYRLDDAVASSTQEDGWVICRVFKKKNIVVQHQAGQSGASNKLVGAGSQSN

SS---QHTASCRQE-TKPPN--ACRNGTSTLEYGRDWD

>TraesCS4A01G420200.1

MSNGQSVVPPGFRFHPTEEELLTYYLAKKVASQRIDLDVIPDVDLNKLEPWDIQERCRIG

QNDWYLFSHKDKKYPTGTRTNRATAAGFWKATGRDKGIYSAAGRIGMRKTLVFYKGRAPH

GHKSDWIMHEYRLDDAVASPAQEDGWVICRVFRKKNIVVQHQ----NASNKLVGAGSQSN

SS-HQQHTASCKQE-TKPTN--ACHSGSSTLEYGRDWD

>TraesCS4A01G420900.1

MSNGQSVVPPGFRFHPTEEELLTYYLAKKVASQRIDLDVIRDVDLNKLEPWDIQERCRIG

QNDWYLFSHKDKKYPMGTRTNRATAAGFWKATGRDKAIYSAAGRIGMRKTLVFYKGRAPH

GRKSDWIMHEYRLEDAV-SSTQEDGWVICRVFKKKNIVAQRQAGQNGASNKLVGAGSESN

SS---THTTSCRQE-TKPTN--VCHSGSSTLEYSGDWE

>TraesCS4A01G421000.1

MSNGHSVVPPGFRFHPTEEELLTYYLAKKVASQPIDLDVIPDVDLNKLEPWDIQERCRIG

QNDWYLFSHKDKKYSTGTRTNRATAAGFWKATGRDKAIYSAAGRIGMRKTLVFYKGRAPH

GHKSDWIMHEYRLDDAVASSAQEDGWVICRVFKKKNIVVQHQAGQNGASNKLVGAGSQSN

SS-HQQHIASCKQE-TKPAN--VCHNGTSTLEYSSDWE

>TraesCS4A01G421100.1

MSNGQSEVPPGFRFHPTEEELLTYYLAKKAASQRIDLDVIRDVDLNKLEPWDIQERCRIG

QNDWYLFSHKDKKYPTGTRTNRATGAGFWKATGRDKAIYSASGTIGTRKTLVFYKGRAPH

GHKSDWIMHEYRLDDAAT--AQEDGWVICRVFKKKNIVVQHQVGQNGASNKLVGVGGRSN

SS-MQQHTTPCKQE-TKPTN--ACHNRNSTLKYGSDWD

>TraesCS4A01G421200.1

MSNGQSVVPPGFRFHPTEEELLTYYLAKKVASQRIDLDVIPDVDLNKLEPWDIQERCRIG

QNDWYLFSHKDKKYPTGTRTNRATAAGFWKATGRDKAIYSAAGLIGMRKTLVFYKGRAPH

GHKSDWIMHEYRLDAAAASSAQEDGWVICRVFKKKNIVVQQQAGQNGASNKLVVAGSRSN

SP-MQQHMTACKQE-TKPTN--ACHNGNSTLKYGCDWY

>TraesCS4A01G421300.1

MSNGQSVVPPGFRFHPTEEELLTYYLAKKVASQRIDLDVIPDVDLNKLEPWDIQERCRIG

QNDWYLFSHKDKKYPTGTRTNRATAAGFWKATGRDKAIYSAAGRIGMRKTLVFYKGRAPH

GHKSDWIMHEYRLDDAVPSSGQEDGWVICRVFKKKNIVVNQ--GQNGASNKLAGAASQSN

SSQQQLHTTPCKQE-AKPA--TACPNG--TLEYGTEWD

>TraesCS4A01G486200.1

M-NTQPQVPPGFRFHPTDEELVDYYLRKKVASRRIDLNVIKDVDLYKIEPWDLQEKCRIG

QSDWYFFSHKDKKYPTGTRTNRATAAGFWKATGRDKPIYAKHCLVGMRKTLVYYKGRAPN

GQKSDWIMHEYRLETNENGPPQEEGWVVCRVFKKRLPTTRRDLDHDAVDDDGPFM-SPMR

SM---QYLNTIPHELESPSSFVSPDDHQINVQAATDWR

>TraesCS4A01G499900.1

MT-TPAVLPVGFRFRPTDEELVRHYLKAKIAGRAPDLLAIPDVDLAAVEPWDLPARSVIK

DPEWFFFARRDRKYPKSSRSCRSTAAGYWKATGKDRLIRGPGALIGVKKTLVFHRGRAPR

GARTPWIMHEYTATDPNPAGPQNDSFVLYRLFNKQDEEAPAPISEPPSTSPPA---ASMV

TAPDIQHQQGIAARASQPILAAGCSTESSSSMAGHNMA

>TraesCS4B01G069400.1

MA-HRRDLAPGWTFNPPDRELTDVYLRREIDGQPIAASFMHHVDVYSAAPEKLPGEA--G

KRVWYFFTTLRHAGKNGGRMSRTIADGWWHSEGSLKAVK-GSAAGGALQKLTYK---ESA

SGSGGWLMTEYSIH----G---AGDLVLCKVYKTPRRPRLARRSSPASSKRKASGDRQPD

SSPAF-----APPCAAAPPSN----------YDGDDLL

>TraesCS4B01G072400.1

---MEEGLPPGFRFHPTDEELITYYLSRKVSDFSFVTRAIADVDLNKCEPWDLPSKASMG

EKEWYFFSMRDRKYPTGIRTNRATESGYWKTTGKDKEIFHAGRLVGMKKTLVFYGGRAPK

GEKTSWVMHEYRIQNKFPYKPNKEEWVVCRVFKKSQIVKMRPQDSPD--DGHASLGSMLG

SGPAA--NPAMHKA-LAPFAGDALFGNAMAK---VDME

>TraesCS4B01G098200.1

MDDGALQLPPGFRFHPTDEELVMYYLCRKCGGLPIAAPVIAEVDLYKFEPWRLPEKAAGG

SKEWYFFSPRDRKYPNGSRPNRAAGTGYWKATGADKPVGS--RPVAIKKALVFYAGKPPK

GVKTNWIMHEYRLADVDRNALRLDDWVLCRIYNKKGVIERYDTPDS---DVADVKPPRQG

AGPMKQQSADRDSNHSMPRL-LSSPSPDFPSDDGIDGS

>TraesCS4B01G102700.1

MAENPPRLPPGVYFSPTREESVAL-LDRWIAGKEVPADFVSHADIYGDSPDALPGSARAG

QHTWWFLCE--RQSP-GARAGRKVTGGYWRVERSEAE---GG---AVKSYFGFFLGP-SR

KEKTPWLTQEFTSATDGGG--KKGVPALYMLYVSPRATDDELR---GGE-------VGPD

GSP--RH----------PAA-----------VLG----

>TraesCS4B01G132500.1

MT---RAL--GMRFNPKGEEAIAVYLVPWLLRQPLP-DIIHEAEVYKSEPKDLP-RL---

THHRFFFTTRRQKAGSGFRMKRTAGAGRWVTSDKTEVKNSADETIGYHEKLRYEKGQS--

-GKSEWLMDEYHCRD---G---KEERVLCRLYVSPNAKPGSQQSAAADHHQRSICRPQEQ

SSPARQQPSWLPPQAPLPQRQRTIQQKACNEEEGEDVE

>TraesCS4B01G173600.1

M-SDPAMLPPGFRFHPTDEELILHYLRNRAAESPCPVSIIADVDIYKFDPWALPSKASYG

DREWYFFTPRDRKYPNGVRPNRAAGSGYWKATGTDKPIRCSAESVGVKKALVFYKGRPPK

GIKTNWIMHEYRLAAADAASMRLDDWVLCRIYKKTSQVSPMAVPPLSDHELDEPSGSSAG

AAPISRHQHA----LGHPIMSGAAGKRKRPSEDG-DRN

>TraesCS4B01G174000.1

MSAAGLDLPPGFRFHPTDEEIISHYLTPKALDHRFCSGVIGEVDLNKCEPWHLPGKAKMG

EKEWYFFCHKDRKYPTGTRTNRATESGYWKATGKDKEIFRGRILVGMKKTLVFYLGRAPR

GEKTGWVMHEFRLEGK--PRSAKDEWAVSKVFNKELTATNGAMAAAEAGERVSSLGGDVD

TGPANRYSPSRETGSTDPNAAAATPSSQNQE---LDQF

>TraesCS4B01G242600.3

MAEQQPGLPRGAQFNPSDSDLLWH-LAAEMNGHPF-IEFIKYGRFGCTHPRDMPGMRQDG

-HALYFFHKKVNHN-N--ENDKDI---SWQKSGPSRSIILDGGLQGCKEIFALYAFK-NS

PQRTDWELHQYHIKNTME---NEGELVLSKIFHKSQKCHCE--ASKASAQDNSVTDATES

QAPSENHEDSLPSLSMEPIVKTDSIETALEEEWGRDLQ

>TraesCS4B01G320300.1

M-SMESCVPPGFRFHPTDEELVGYYLRKKVASQKIDLDVIRDIDLYRIEPWDLTEHCGIG

QNEWYFFSFKDRKYPTGTRTNRATMAGFWKATGRDKAVHERSRLIGMRKTLVFYKGRAPN

GQKTDWIMHEYRLETDENAPPQEEGWVVCRAFKKRTAYPNRGMMERSSYNEVNAMSASTY

AA---RYLVELPQL-ESPSAPDAASGGSRRRAATTDWR

>TraesCS4B01G328600.1

MA-EVDQ-LPGFRFHPTEEELLGFYLSRVALGKKLHFDIIGTLNIYRHDPWDLPGIAKIG

EREWYFFVPRDRKAGSGGRPNRTTERGFWKATGSDRAIRSTGRVIGLKKTLVFYQGRAPR

GTKTDWVMNEYRLPDTGA-APPKEDTVLCKVYRKATPLKELEQRAFEMEEMKQRPGPSDD

SS---SHTSSLPQAVNPPCGLLPAANHGMSNSQGLDLP

>TraesCS4B01G328700.1

MA-EVDQ-LPGFRFHPTEEELLGFYLSRVALGKKLHFDIIGTLNIYRHDPWDLPGMAKIG

EREWYFFVPRDRKAGSGGRPNRTTERGFWKATGSDRAIRSTGRVIGLKKTLVFYQGRAPR

GTKTDWVMNEYRLPETGA-APPSEDTVLCKVYRKATPFKELEQRAFEMEEMKQRSGPSDD

SS---SHTSSLSQAVNPPCSILPAANYGMPNSKGLDLP

>TraesCS4B01G328800.1

MA-EVDQ-LPGFRFHPTEEELLGFYLSRVALGKKLHFDIIGTLNIYRHDPWDLPGMAKIG

EREWYFFVPRDRKAGSGGRPNRTTERGFWKATGSDRAIRSTARVIGLKKTLVFYQGRAPR

GTKTDWVMNEYRLPDSGA-APPQEDTVLCKVYRKATPLKELEQRAFEMEEMKQRSGPSDD

SS---CHMSSLSKAVNPPCGLLPAANHG---SQGVDLP

>TraesCS4B01G328900.1

MA-EVDQ-LPGFRFHPTEEELLGFYLSRVALGKKLHFDIIGTLNIYRHDPWDLPGMAKIG

EREWYFFVPRDRKAGSGGRPNRTTERGFWKATGSDRAIRSTGRVIGLKKTLVFYQGRAPR

GTKTDWVMNEYRLPDSGA-APPQEDTVLCKVYRKATPLKELEQRAFEMEEMKQRSGPSDD

SS---SHTSSLSQAVNPPYGLLPAANHGMTNSQGLDLP

>TraesCS4B01G329100.1

MA-EVEQ-LPGFRFHPTEEELLGFYLSRVALGKKLHFDIIGTLNIYRHDPWDLPGLAKIG

EREWYFFLPRDRKAGSGGRPNRTTERGFWKATGSDRAIRSTARVIGLKKTLVFYQGRAPR

GTKTDWVMNEYRLPDTGA-PPPQEDTVLCKVYRKATPLKELEQRAFAMEEMKQRPCPSDD

SS-----TSSLSQAVNPPCGLLPAANHGMPNSQGLDLP

>TraesCS4B01G350600.1

M--APVSLPPGFRFHPTDEELIIYYLKSKINGRQIELEIIPEVDLYKCEPWDLPEKSFLP

DLEWYFFSPRDRKYPNGSRTNRATKAGYWKATGKDRKVNSQKRAVGMKKTLVYYRGRAPH

GSRTDWVMHEYRLDERECDNGLQDAYALCRIFKKTAPGPKIME---AAQEPPQWTPSSSF

ATPAAQHVASASQESSYPDMWAAKAKEEVDNEAGFDLY

>TraesCS4B01G384700.1

MAEEDDVVLPGYRFHPTDEELVTFYLRRKVAGKPLSIEVIREMDIYKHDPWDLPKGSTVG

EKEWYFFCLRGRKYRNSIRPNRVTGSGFWKATGIDRPIYSAAVLIGLKKSLVYYLGKFGK

CTKTDWMMHEFRLLPSAPSMQEAEVWTICRIFRRTITYRKQQTTAPTAADQSSNTGSSEV

IAPIPQY-------PAAPEPEHAVAENDFYETAGYD--

>TraesCS4D01G071200.1

---MEEGLPPGFRFHPTDEELITYYLSRKVSDFSFATRAIADVDLNKCEPWDLPSKASMG

EKEWYFFSMRDRKYPTGIRTNRATESGYWKTTGKDKEIFHGGRLVGMKKTLVFYGGRAPK

GEKTSWVMHEYRIQNKFPYKPNKEEWVVCRVFKKSQIVKMRPQDSPD--DAHASLGSILG

PGPAA--NPAMQKA-LAPFAGDALFGNAMAK---VDME

>TraesCS4D01G094400.1

MDDGALQLPPGFRFHPTDEELVMYYLCRKCGGLPIAAPVIAEVDLYKFEPWRLPEKAAGG

AKEWYFFSPRDRKYPNGSRPNRAAGTGYWKATGADKPVGS--RPVAIKKALVFYAGKPPK

GVKTNWIMHEYRLADVDRNALRLDDWVLCRIYNKKGVIERYDTADS---DAADVKPSRPG

AGPMKQQSADRDSNHSMPRL-LSSPSPDFPSDDGIDGS

>TraesCS4D01G099300.1

M--NPPRLPPGVYFSPMREESVAL-LDRWIAGKEVPADFVSRADIYGESPDALPASARAG

QHTWWFLCE--RQCP-GSRAGRKVTGGHWRAERSEAE---GG---GVESHFVFFLGP-SR

KDKTPWLVQEFTSANDDGG--KKGVPA---LYVSPRATDDELR---GGE-------VGPD

GSP--HH-------AASPAA-----------VLG----

>TraesCS4D01G099900.1

MAESPPRLPPGVYFSPTREESVAL-LDRWIAGKEVPADFVCHADIYGESPDALPASARAG

QHTWWFLCE--RQCP-GSRAGRKVTGGHWRAERSEAE---GG---GVESHFVFFLGP-SR

KEKTPWLVQEFTSANDDGG--KKGVPALYMLYVSPRATDDELR---GGE-------VGPD

GSP--HH-------AAVPAA-----------VLG----

>TraesCS4D01G127300.1

MA---RAL--GMRFNPTGEQAIARYLVPWLLDQPLP-DIIHEAEVYKSEPKDLP-RL---

THHRFFFTTIRQKAGTGFRMKRTAGAGKWVTSDKTEVKNSADETIGYHEKLRYAKGQS--

-GKSEWLMDEYHCRD---G---KEERVLCRLYVSPNAKPGSQQSAAADHHHRSICRPQEQ

SSPARQQPSWCPPQAPLPQRQRTIQQKACNEEEGEDVQ

>TraesCS4D01G175700.1

M-SDPAMLPPGFRFHPTDEELILHYLRNRAAESPCPVSIIADVDIYKFDPWALPSKASYG

DREWYFFTPRDRKYPNGVRPNRAAGSGYWKATGTDKPIRCSAESVGVKKALVFYKGRPPK

GIKTNWIMHEYRLAAADGASMRLDDWVLCRIYKKTSQVSPMAVPPLSDHELDEPSGSSAG

AAPISRHQHA----FGHPIMGGAAGKRKRPSEDG-DHN

>TraesCS4D01G176000.1

MSAAGLDLPPGFRFHPTDEEIISHYLTPKALDHRFCSGVIGEVDLNKCEPWHLPGKAKMG

EKEWYFFCHKDRKYPTGTRTNRATESGYWKATGKDKEIFRGRVLVGMKKTLVFYLGRAPR

GEKTGWVMHEFRLEGK--PRSAKDEWAVSKVFNKELTATNGAMAAAEAGERVSSFGGDVD

TGPANRYSPSRETGSTDPNAAAATPSSQNQD---LDQF

>TraesCS4D01G242000.3

MAEQQPGLPRGVEFNPSDSDLLWH-LAAEMNGHPF-IEFIKSGRFGCTHPRDMPGMRQDG

-HALYFFHKKVNDN-N--ENDKDI---SWQKSAPSRSIILDGGLRGCKETFALYAFK-NS

PRRTDWELHRYYIKNIME---NEGELVLSKVFYKSQKGHCE--AAKASAQDNSVTDATES

QAPSENHEDSLPSLSMEPIVKTDSIETALEEEWGRDLQ

>TraesCS4D01G316800.1

M-SMESCVPPGFRFHPTDEELVGYYLRKKVASQKIDLDVIRDIDLYRIEPWDLTEHCGIG

QNEWYFFSFKDRKYPTGTRTNRATMAGFWKATGRDKAVHERSRLIGMRKTLVFYKGRAPN

GQKTDWIMHEYRLETDENAPPQEEGWVVCRAFKKRTAYPNRGMMERSSYNEANAMSASTY

AA---RYLVELPQL-ESPSAPDAASGGSRRRAATTDWR

>TraesCS4D01G325700.1

MA-EVDQ-LPGFRFHPTEEELLGFYLSRVALGKKLHFDIIGTLNIYRHDPWDLPGMAKIG

EREWYFFVPRDRKAGSGGRPNRTTERGFWKATGSDRAIRSTGRVIGLKKTLVFYQGRAPR

GTKTDWVMNEYRLPDTGA-APPSEDTVLCKVYRKATPLKELEQRAFEMEEMKQRPGSSDD

SS---SCTSSLPHAVNPPCGLLPAANHEMPNSQGLDLP

>TraesCS4D01G325800.1

MA-EVDQ-LPGFRFHPTEEELLGFYLSRVALGKKLHFDIIGTLNIYRHDPWDLPGMAKIG

EREWYFFVPRDRKAGSGGRPNRTTERGFWKATGSDRAIRSTARVIGLKKTLVFYQGRAPR

GTKTDWVMNEYRLPDNGA-PPPQEDTVLCKVYRKATPLKELEQRAFEIEEMKQRSGPSDD

SS---SHTSSLPQAVNPPCGLLPAANHGMSNSQGVDLP

>TraesCS4D01G345300.1

M--APVSLPPGFRFHPTDEELIIYYLKSKINGRQIELEIIPEVDLYKCEPWDLPEKSFLP

DLEWYFFSPRDRKYPNGSRTNRATKAGYWKATGKDRKVNSQKRAVGMKKTLVYYRGRAPH

GSRTDWVMHEYRLDERECDNGLQDAYALCRIFKKTAPGPKIME---AAQEQPQWTPSSSF

ATPAAQHVASASQESSYPDMWAAKAKEEVDNEAGFDLY

>TraesCS5A01G049100.2

M--VETELPPGFRFHPRDDELICDYLAPKVTGKVFRRPPMVDVDLNKVEPWDLPVTASVG

GKEWYFYSLKDRKYATGQRTNRATVSGYWKATGKDRVVARRGALVGMRKTLVFYQGRAPK

GRKTEWVMHEYRLEGAHEQASKQEDWVLCRVICKKKSGVGAPRPRNSVHGTPTDTSTSAA

SNPVTDHSMG----SMSPQMA-----------------

>TraesCS5A01G099000.1

MA---RDMAAAFKFDPTDADIVASYLLPRAVGLD-PGHAVIDDDPMSLPPWDLMEKH--N

SHQAFFFGPPR----NGGPVKRVVGGGTWQGQNGSVGVTCDGVDISYRYDLTYK---AGD

KAPSGWVMSEYQITSPPL----L-STVLTRIGLTVAAREQRKRPEPA--QH-----AAEH

QAP----------------------------LNG----

>TraesCS5A01G127200.1

MT-GD--LTPGFRFYPTEDELLGFYLRHRLAGTTPHVDVIPVVDVYSHHPSHLRSMAGES

AEQWFFFCPRAERELRGGRPARTTPSGYWKATGSPSCVFSSSKVIGVKRTMVFYQGRAPT

GAKTTWKMNEYKAVAADDPLRLRNEFSVCRLYISTGTLRSFDRNTSGNGQAAARRHANTA

TS--------------------------------FDMN

>TraesCS5A01G143100.1

MEGGGLSLPPGFRFHPTDEEIITSYLLRKFLDPSFVSRAVGEVDLNSCEPRDLPGKANMG

EKEWYFFVHKDLKYPTGSRANRATKEGYWKATGKDREIFKPRELVGMKKTLVFYRGRAPR

GAKSEWVMHEFRLEGK---NNPKDEWVVCKVFNKKGEAKAARAADVDGEGAGDFTDTNSA

TSPTSQHALGGVVGFGEPAKGAAMSGRPAAANLGPRFY

>TraesCS5A01G143200.1

MPAAMPALPPGFRFHPTDEELIVHYLGRQAASMPSPVPIIAEVNIYKCNPWDLPGKALFG

ENEWYFFSPRDRKYPNGARPNRAAGSGYWKATGTDKAILSTPESIGVKKALVFYRGKPPK

GVKTDWIMHEYRLTAADNSSMRLDDWVLCRIHKKCNNLHNFSSSDQEQEEQESST-----

SSPSEQFYAALSHL-------DGAGAGASSSDAGADYQ

>TraesCS5A01G220900.1

M--SQTCLPPGFRFHPTDVEFVSYYLKRKIMGKKLFVEAISEVELYKFAPWDLNHKSCLQ

DLEWFFFCPHDKKNPKGSWTNRTTPNGYWKTSGKDGTIDLNSRIVGLKKTLIFHEGKAPK

GNRTDWVMYEYKMEDETLTGFSKDAYVLS------GLGPRI--EQYADGNEWENLDTYIL

TAPAPEH-------------------G-----------

>TraesCS5A01G228000.1

M-KSEEVLLPGFRFHPTDEELVGFYLKRKIQQKPLSIELIRQLDIYKYDPWDLPKLASSG

EKEWYFYCPRDRKYRNSARPNRVTGAGFWKATGTDRPIYSSEKCIGLKKSLVFYKGRAAK

GIKTDWMMHEFRLPSLTDNIPANDAWAICRIFKKPSSMAQR--LSHSEPDLLSAL-ASHF

VSPSSNFETQLQRSSIAPGII----------CNGIDTN

>TraesCS5A01G245900.2

M-QHQLELPSGFRFHPTDEEIITSYLVPKVLNPTFTAIAIAEVDLNKNDPYELPKKAKMG

EKEWYFYCQKDRKYPTGIQTNRATKAGYWKATGKDKEIFHPLTLIGMKKTLVFYKGRAPR

GEKTNWVMHEYRLEISKQNASSKKERVVCKIFHKNIGVKKVVTPSYAGEQQQGSLNLSLA

RVPVNPHQMGADQCGVEPGSEATTTIDS----------

>TraesCS5A01G271500.2

ME-PPPRWPPGFRFSPTDEELVLFFLKRRVAAGR-PSPYIADVDVYKSHPSHLPERSALG

DKQWFFCSRLDRKYPNGSRASRTTADGYWKATGKDRSICNAGRAVGNKKTLVYHHGRAPR

GERTDWVMHEYTILADALPARGRESYALYKLFEKSGVGPKNEQGAP-EEDDDCELPSEPQ

TSPLQEHFSTVPRHSVDPSSACTEETSAMRSASGADSQ

>TraesCS5A01G275900.1

MEGEEESLPPGFRFHPTDEELITYYLRGKIADAGFTARAITEVDLNKCEPWDLPEKAKMG

EKEWYFFSLRDRKYPTGVRTNRATNAGYWKTTGKDKEIFTGQELVGMKKTLVFYKGRAPR

GEKSNWVMHEYRLHLKPASKSNKDEWVVCRIFAKSPGVKKYPSSTNASHHHPYTLDGGRG

AAPPQHHEQHMAAGGGVPAAGHAAGGAGMRY---LDVD

>TraesCS5A01G291200.1

MEQQQQQLPPGFRFYPTDVELVLQYLRRMALDRPLPAAVIPVVHAAAMDPWDLPGAS---

GESAYFFSQRQR----GGRRRRAA-GGYWKATGKEKPVFVQLLLVGVKTALAFHRGK---

-SRTDWVMHEYRLAGAAEDGSQSSEWVVCRVSLKSRARRTAAGETTGDHQQEQPSPSTSS

SS---SH-------QQHPRR------------------

>TraesCS5A01G339600.1

M-KSEEIIMPGFRFHPTDEELVSFYLKKKIQQKPISIELIRQLDIYKFDPWDLPKLASTG

ETDWYFYCPRDRKYRNSARPNRVTAAGFWKATGTDRPIYSSERCIGLKKSLVFYRGRAAR

GIKTDWMMHEFRLPSLTDNIPLNDSWTICKIFKKTSSMAQQMVLSHTQQDLFSAM-ASHF

ASPNTSFQTQ-TSQHTAPPL-----------ITGADVN

>TraesCS5A01G353500.1

M--APVGLPPGFRFHPTDEELVNYYLKRKVHGQSIELDIIPEVDLYKCEPWELAEKSFLP

DPEWYFFGPRDRKYPNGCRTNRATRAGYWKSTGKDRSINYQKRSIGMKKTLVFYQGRAPQ

GIRSNWVMHEYRIEESECTMGVQDSYALCRVFKKNVPAGEFEKQGESSQNQEQVTDESST

SN------------------------------------

>TraesCS5A01G411700.1

MADDDDVVLPGYRFHPTDEELVTFYLRRKVARKSLRIEVIREMDIYKHDPWDLPKASTVG

EKEWYFFCLRGRKYRNSIRPNRVTGSGFWKATGIDRPIYSAAVSIGLKKSLVYYRGSAGK

GTKTDWMMHEFRLPPAAASMQEAEVWTICRIFRRTITYRKQQQPAPTTADSNSNTGSSEG

AAPIPQY-------LAGPIPELAVATNDFHKPAGYDYR

>TraesCS5A01G411800.1

MAEEDDVVLPGYRFHPTDEELVTFYLRRKVARKSLRIEVIREMDIYKHDPWDLPKASTVG

EKEWYFFCLRGRKYRNSIRPNRVTGSGFWKATGIDRPIYSAAVSIGLKKSLVYYRGSAGK

GTKTDWMMHEFRLPPAAASMQEAEVWTICRIFRRTITYRKQQQPAPAAADSSSNTGSSEA

AAPIPQY-------PAAPVPEHAVAANDFYKSAGYDYR

>TraesCS5A01G411900.1

MAEDDDVVLPGYRFHPTDEELVTFYLRRKVARKPLRIEVIREMDIYKHDPWDLPKASTVG

EKEWYFFCLRGRKYRNSIRPNRVTGSGFWKATGIDRPIYSAGVSIGLKKSLVYYRGSAGK

GTKTDWMMHEFRLPP--ASMQEAEVWTICRIFRRTITYRKQQQPAPAAADSNSNTGSSEA

AAPIPQY--------AAPVPEHAVAANDFYKPTGYDYR

>TraesCS5A01G467300.1

MNQSEAALPPGVRFKPTDAEII-WYLERKYHGHPLPVNFIKEFDVFEDHPDTVQEKY--G

EGVWYVFSSRDRKYKNGTRPVRSVGVGFWKSSGKEEDVLDKNVKIGRVNTLTFKLGHQPK

GTSTPWRLKEYRMEKYQESSMLLDPWVICKLFRTKNPPARQDLPVQS---------ASEA

NGP--------------PNGP----------ELGDDFT

>TraesCS5A01G468300.1

MAEAELNLPPGFRFHPTDDELVEHYLCRKAAGQRLPVPIIAEVDLYRFDPWALPDRALFG

TREWYFFTPRDRKYPNGSRPNRAAGNGYWKATGADKPVAPRGRTMGIKKALVFYAGKAPK

GVKTDWIMHEYRLADAGRGSLRLDDWVLCRLYNKKNEWEKMQLQQQGEEETMME--PKAE

EAPSEQQAAA----AQSPAAAMVPKKEAADEFVDLSYD

>TraesCS5A01G491700.1

M-SMESCVPPGFRFHPTDEELVGYYLRKKVASQKIDLDVIRDIDLYRIEPWDLTEHCGIG

QNEWYFFSFKDRKYPTGTRTNRATMAGFWKATGRDKAVHERSRLIGMRKTLVFYKGRAPN

GQKTDWIMHEYRLETDENAPPQEEGWVVCRAFKKRTAYPNRGMMERSSYNEVNAMSASTY

AA---RYLVELPQL-ESPSAPDAASGGSRRRETTKDWR

>TraesCS5A01G500400.1

MA-EVDQ-LPGFRFHPTEEELLGFYLSRVALGKKLHFDIIGTLNIYRHDPWDLPGMAKIG

EREWYFFVPRDRKAGSGGRPNRTTERGFWKATGSDRAVRSTGRVIGLKKTLVFYQGRAPR

GSKTDWVMNEYRLPDTGA-APPSEDTVLCKVYRKATPLKELEQRAFEMEEMKQRPRSSDD

SS---SHTSWLPHAVNPPCGFLPAANHGMSNSQGVDLP

>TraesCS5A01G500500.1

---EVDQ-LPGFRFHPTEEELLGFYLSRVALGKKLHFDIIGTLNIYRHDPWDLPGMAKIG

EREWYFFVPRDRKAGSGGRPNRTTERGFWKATGSDRAIRSSGRVFGLKKTLVFYQGRAPR

GTKTDWVMNEYRLPDTGA-PPPQEDTVLCKVYRKATPLKELEQRAFAMEEMKQRYGSSDD

TS---SHTSSLPEAANPPCGLLPPANHGMSNSHG----

>TraesCS5A01G500600.1

MA-EVDQ-LPGFRFHPTEEELLGFYLSRVALGKKLHFDIIGTLNIYRHDPWDLPGMAKIG

EREWYFFVPRDRKAGSGGRPNRTTERGFWKATGSDRAIRSTGRVIGLKKTLVFYQGRAPR

GTKMDWVMNEYRLPDNGA-PPPQEDTVLCKVYRKATPLKELEQRAFQMEEMKQRSGPSDD

SS---SHTSSLSQTVNPPCGLLPAANHGLSNNQGLDLP

>TraesCS5A01G500700.1

MA-EVDQ-LPGFRFHPTEEELLGFYLSRVALGKKLHFDIIGTLNIYRHDPWDLPGMAKIG

EREWYFFVPRDRKAGSGGRPNRTTERGFWKATGSDRAIRSTGRVIGLKKTLVFYQGRAPR

GTKMDWVMNEYRLPDNGA-PPPQEDTVLCKVYRKATPLKELEQRAFQMEEMKQRSGQSDD

SS---SHTSSLSQAVNPPCGLLPAANHGMSNSQGVDLP

>TraesCS5A01G519300.1

M--APVSLPPGFRFHPTDEELIIYYLKSKINGRQIELEIIPEVDLYKCEPWDLPEKSFLP

DLEWYFFSPRDRKYPNGSRTNRATKAGYWKATGKDRKVNSQKRAVGMKKTLVYYRGRAPH

GSRTDWVMHEYRLDERECDNGLQDAYALCRIFKKTAPGPKIME---AAQEQPQWTPSSSF

ATPAAQHVASASQESSYPDMWAAKAKEEVDNEAGLDLY

>TraesCS5B01G054200.2

M--VETELPPGFRFHPRDDELICDYLAPKVTGKVFRRPPMVDVDLNKVEPWDLPVTASVG

GKEWYFYSLKDRKYATGQRTNRATVSGYWKATGKDRVVARRGALVGMRKTLVFYQGRAPK

GRKTEWVMHEYRLEGAHEQASKQEDWVLCRVICKKKSGVGAPRPRNTVHGTSTDTSTSSA

SNPVTDHSMG----SMSPQMA-----------------

>TraesCS5B01G104200.1

MA---RDMAAAFKFDPTDADIVASYLLPRAVGLD-PGHAVIDDDPMSLPPWDLMEKH--N

SDQAFFFGPPR----NGGRVKRVVGGGMWQGQNGRVGVTCDGVDISYRYDLTYK---AGN

KDPSGWVMSEYQITSPPL----L-STVLTRIGLTVAAGEQRKRQEAA--QQ-----AAEH

QAP----------------------------LNG----

>TraesCS5B01G126300.1

MT-GD--LTPGFRFYPTEDELLGFYLRHRLAGTTSHVDVIPVVDVYSHHPSHLRSMAGES

AEQWFFFYPRAERELRGGRPARTTPSGYWKATGSPSGVFSSSKVIGVKRTMVFYQGRAPT

GAKTTWKMNEYKAVAADNPLRLRNEFSVCRLYISTGTLRSFDRNTSGNGQAAARRHANTA

TS--------------------------------FDMD

>TraesCS5B01G141900.1

MEGGGLSLPPGFRFHPTDEEIITSYLLRKFLDPSFVSRAVGEVDLNSCEPRDLPGKANMG

EKEWYFFVHKDLKYPTGSRANRATKEGYWKATGKDREIFKPRELVGMKKTLVFYTGRAPR

GAKSEWVMHEFRLEGK---NNPKDEWVVCKVFNKKGEVKATRAADVDGEGAGDFTDTNSA

PSPISQHALEGVVGFGEPAKGAAMTGRPAAANLGARFY

>TraesCS5B01G142100.1

MPAAMPALPPGFRFHPTDEELIVHYLRRQAASMPSPVPIIAEVNIYKCNPWDLPGKALFG

ENEWYFFSPRDRKYPNGARPNRAAGSGYWKATGTDKAILSTPESIGVKKALVFYRGKPPK

GVKTDWIMHEYRLTAADNSSMRLDDWVLCRIHKKCGNLPNFSSSDQEQEEQESS------

SSPSEQFYAALSHL-------LLDGAGASSSDAGADYQ

>TraesCS5B01G226800.1

M-KLEEALLPGFRFHPTDEELVGFYLKRKIQQKPLSIELIRQLDIYKYDPWDLPKLASSG

EKEWYFYCPRDRKYRNSARPNRVTGAGFWKATGTDRPIYSSEKCIGLKKSLVFYKGRAAK

GIKTDWMMHEFRLPSLTDNIPANDAWAICRIFKKPSSMAQR--LSHSEPDLLSAL-ASHF

VSPSSNFETQLQRSSIAPGII----------CNGIDTN

>TraesCS5B01G243300.1

M-QPKLVLPPGLRFHPTDEQIITSYLVPK--------------------PWELPKKAKMG

ENKWYFYCQKDHEDPSGIQTNRATKVGYWKATGKDKEILDSTALIGKKKTLVFYKGRAPT

GEETKWVMHEYRLEIGKQ-ST---DISKATIINAS--SKHRKESGDAGQNAHAHLHSSMA

SS---YH---VEQS-------------------GREWA

>TraesCS5B01G275200.1

MEGEEESLPPGFRFHPTDEELITYYLRGKIADGSFTARAITEVDLNKCEPWDLPEKAKMG

EKEWYFFSLRDRKYPTGVRTNRATNAGYWKTTGKDKEIFTGQELVGMKKTLVFYKGRAPR

GEKSNWVMHEYRLHLKPASKSNKDEWVVCRIFAKSPGVKKYPSSTNASHHHPYTLDGGRG

GAPPHHHEQHMAAGGGIPAAGHAAGGAGMRY---LDVD

>TraesCS5B01G290200.1

ME---QQLPPGFRFHPTDVELLLQYLRRMALDRPLPAAVIPVVHAAAMDPWDLPGAS---

GESAYFFSQRQR----GGRRRRAT-GGYWKATGKEKPVFVQLLLVGVKTALAFHRGK---

-SRTDWVMHEYRLAGAADGGSQSSEWVVCRVSMKSRARRSAAGETTGDHQQEQPSPSTSS

SS---SH-------QQHPTR------------------

>TraesCS5B01G338400.1

M-KSDEIIMPGFRFHPTDEELVSFYLKKKIQQKPISIELIRQLDIYKFDPWDLPKLASTG

ETDWYFYCPRDRKYRNSARPNRVTAAGFWKATGTDRPIYSSERCIGLKKSLVFYRGRAAR

GIKTDWMMHEFRLPSLTDNIPLNDSWTICKIFKKTSSMAQQMALSHTEQDLFSAM-ASHF

ASPNTSFQTQ-PSQHTAPPL-----------ITGADVN

>TraesCS5B01G338700.1

M-KSDEIIMPGFRFHPTDEELVSFYLKKKIQQKPISIELIRQLDIYKFDPWDLPKLASTG

ETDWYFYCPRDRKYRNSARPNRVTAAGFWKATGTDRPIYSSERCIGLKKSLVFYRGRAAR

GIKTDWMMHEFRLPSLTDNIPLNDSWTICKIFKKTSLMAQQMAPSHTEQDLFSAM-ASNF

ASPNTSFQTQ-PSQHTAPPL-----------ITGADVN

>TraesCS5B01G356000.1

M--APVGLPPGFRFHPTDEELVNYYLKRKIHGLHIELDIIPEVDLYKCEPWDLAEKSFLP

DPEWYFFGPRDRKYPNGFRTNRATRAGYWKSTGKDRRVMTQQRAIGMKKTLVYYRGRAPQ

GVRTDWVMHEYRLDDKDSTIAIQDTYALCRVFKKNAICAEVEELQEGSRQEYYQTPAGST

SS-AE---------------------------AG----

>TraesCS5B01G356100.1

M--APVGLPPGFRFHPTDEELVNYYLKRKVHGQSIELDIIPEVDLYKCEPWELAEKSFLP

DPEWYFFGPRDRKYPNGCRTNRATRAGYWKSTGKDRSINYQKRSIGMKKTLVFYQGRAPQ

GIRSNWVMHEYRIEESECTMGVQDSYALCRVFKKNVPAGEFEKQGESSQNQEQVTEESST

SN------------------------------------

>TraesCS5B01G415400.1

MAEEDDVVLPGYRFHPTDEELVTFYLRRKVAKKSLRIEVIREMDIYKHDPWDLPKASTVG

EKEWYFFCLRGRKYRNSIRPNRVTGSGFWKATGIDRPIYSATMSIGLKKSLVYYRGSAGK

GTKTDWMMHEFRLPPAAASMQEAEVWTICRIFRRTITYRKQQTPAPAAADSSSNTGSSEG

AAPISQY-------PAAPEPELTVAPNDFHKPAGYDYR

>TraesCS5B01G415600.1

LAGEEDVVLPGYRFHPTDEELVTFYLRRKVARKSLSIEVIREMDIYKHDPWDLPKASTVG

EKEWYFFCLRGRKYRNSIRPNRVTGSGFWKATGIDRPIYSAGVSIGLKKSLVYYRGSTGK

GTKTDWMMHEFRLSPAAASMQEAEVWTICRIFRRAITYRKQQQPPPAAADSNSNTGSSEA

GAPIPQY-------PAVPEPEHAVAANDFYKPAGYDYR

>TraesCS5B01G415700.1

MAEEEDVVLPGYRFHPTDEELVTFYLRRKVARKPLRIEVIREMDIYKHDPWDLPKASTVG

EKEWYFFCLRGRKYRNSIRPNRVTGSGFWKATGIDRPIYSAGVSIGLKKSLVYYRGSAGK

GTKTDWMMHEFRLPPAAASMQEAEVWTICRIFRRTITYRKQQQPAPAAADSNSNTGSSEA

SAPIPQY-------PAPPVPEHAVAASDFYKPAGYDYR

>TraesCS5B01G480900.2

MAEAELNLPPGFRFHPTDDELVEHYLCRKAAGQRLPVPIIAEVDLYRFDPWALPDRALFG

TREWYFFTPRDRKYPNGSRPNRAAGNGYWKATGADKPVAPRGRTMGIKKALVFYAGKAPK

GVKTDWIMHEYRLADAGRGSLRLDDWVLCRLYNKKNEWEKMQLQQQGGEEMMVE--PKAE

EAPSEQQAAA----AQSPAAAMVPKKEAAEEFVDLSYD

>TraesCS5D01G059700.2

M--VETELPPGFRFHPRDDELICDYLAPKVTGKVFRRPPMVDVDLNKVEPWDLPVTASVG

GKEWYFYSLKDRKYATGQRTNRATVSGYWKATGKDRVVARRGALVGMRKTLVFYQGRAPK

GRKTEWVMHEYRLEGAHEQASKQEDWVLCRVICKKKSGVGAPRPRNTVHGTSTDTSTSAA

SNPVTDHSMG----SMSPQMA-----------------

>TraesCS5D01G111300.1

MA---RDMAAAFKFDPTDADIVASYLLPRAVGLD-PGRAVIDDDPMSIPPWDLMEKH--N

SDQAFFFGPPR----NGGRVTRVVGGGTWQGQNGRVGVTCDGVDISYRYDLTYK---AGD

KAPSGWVMSEYQITSPPL----L-STVLTRIGLIVAAREQRKRPEAA--QQ-----AAEH

QAP----------------------------LNG----

>TraesCS5D01G134800.1

MT-GD--LTPGFRFYPTEDELLGFYLRHRLAGTTPHVDVIPVVDVYSHHPSHLRSMAGES

AEQWFFFCPRAERELRGGRPARTTPSGYWKATGSPSCVF-SSKVIGVKRTMVFYQGRAPT

GAKTTWKMNEYKAVAADDPLRLRNEFSVCRLYISTGTLRSFDRNTSGNGQAAARRHANTA

TS--------------------------------FDMD

>TraesCS5D01G148800.1

MPAAMPALPPGFRFHPTDEELIVHYLGRQAASMPSPVPIIAEVNIYKCNPWDLPGKALFG

ENEWYFFSPRDRKYPNGARPNRAAGSGYWKATGTDKAILSTPESIGVKKALVFYRGKPPK

GVKTDWIMHEYRLTAADNSSMRLDDWVLCRIHKKCGNLPNFSSSDQEQEEQESS------

SSPSEQFYAALSHL-------LLDGAGASSSDAGADYQ

>TraesCS5D01G148900.1

MEGGGLSLPPGFRFHPTDEEIITSYLLRKFLDPSFVSRAVGEVDLNSCEPRDLPGKANMG

EKEWYFFVHKDLKYPTGSRANRATKEGYWKATGKDREIFKPRELVGMKKTLVFYTGRAPR

GAKSEWVMHEFRLEGK---NNPKDEWVVCKVFNKKGEAKATRAADVDGEGAGDFTDTNSA

PSPTSQHALGGVVGFGEPEKGAAMNGRPAADNLGARFY

>TraesCS5D01G279100.2

ME-PPPRWPPGFRFSPTDEELVLFFLKRRVAAGR-PSPYIADVDVYKSHPSHLPERSALG

DKQWFFCSRLDRKYPNGSRASRTTADGYWKATGKDRSICNAGRAVGNKKTLVYHHGRAPR

GERTDWVMHEYTILADALPARCRESYALYKLFEKSGVGPKNEQGAP-EEDDDCELPSEPQ

ISPLQEHFSTVPRHSVDPSSACTEETSALRSASGADSQ

>TraesCS5D01G283200.1

MEGEEESLPPGFRFHPTDEELITYYLRGKIADAGFTARAITEVDLNKCEPWDLPEKAKMG

EKEWYFFSLRDRKYPTGVRTNRATNAGYWKTTGKDKEIFTGQELVGMKKTLVFYKGRAPR

GEKSNWVMHEYRLHLKPASKSNKDEWVVCRIFAKSPGVKKYPSSTNASHHHPYTLDGGRG

AAPPQHHEQHMAAGGGIPAAGHAAGGAGMRY---LDVD

>TraesCS5D01G298600.1

ME---QQLPPGFRFHPTDVELVLQYLRRMALDRPLPAAVIPVVHAAAMDPWDLPGAS---

GESAYFFSPRQR----GGRRRKAA-SGYWKATGKEKPVFVQLLLVGVKTVLTFHRGK---

-SRTDWVMHEYRLAGAADDGSQSSEWVVCRVSLKSRARRTAAGETTGDQQQEQPSPSTSS

SS---SH-------QQHPRR------------------

>TraesCS5D01G344100.1

M-KSDEIIMPGFRFHPTDEELVSFYLKKKIQQKPISIELIRQLDIYKFDPWDLPKLASTG

ETDWYFYCPRDRKYRNSARPNRVTAAGFWKATGTDRPIYSSERCIGLKKSLVFYRGRAAR

GIKTDWMMHEFRLPSLTNNIPLNDSWTICKIFKKTSSMAQQMALSHTEQDLFSAM-ASNF

ASPNTSFQTH-PSQHTAPPL-----------ITGADIN

>TraesCS5D01G360700.1

M--APVGLPPGFRFHPTDEELVNYYLKRKIHGLHIELDIIPEVDLYKCEPWDLAEKSFLP

DPEWYFFGPRDRKYPNGFRTNRATRAGYWKSTGKDRRVMTQQRAIGMKKTLVYYRGRAPQ

GVRTDWVMHEYRLDDKDSTIAIQDTYALCRVFKKNAICAEVEELQESSRQEYYQTPAGST

SS-AE---------------------------AG----

>TraesCS5D01G420800.1

MADDDDVVLPGYRFHPTDEELVTFYLRRKVAKKSLRIEVIREMDIYKHDPWDLPKASTVG

EKEWY-FCLRGRKYRNSIRPNRVTGSGFWKATGIDRPIYSAAVSIGLKKSLVYYRGSAGK

GTKTDWMMHEFRLPPAAASMQEAEVWTICRIFRRTITYRKQQTPAPAAADSSSNTGSSEG

AAPIPQY-------PAGPMPELAVATNDFHKPAGYDYR

>TraesCS5D01G421000.1

MAEEEDVVLPGYRFHPTDEELVAFYLRRKMARKSLRIEVIREMDIYKHDPWDLPEASTVG

EKEWYFFCLRGRKYRNSIRPNRVTGSGFWKATGIDRPIYSAGVSIGLKKSLVYYRGSAGK

GTKTDWMMHEFRLPPAAASMQEAEVWTICRIFRRAITYRNQQQPAPAFADSNSNTGSSEA

AAPIPQY-------PAAPVPEHAVAANDFYKPAGYDYR

>TraesCS5D01G421100.1

MAEEEDVVLPGYRFHPTDEELVTFYLRRKVARKSLRIEVIREMDIYKHDPWDLPKASTVG

EKEWYFFCLRGRKYRNSIRPNRVTGSGFWKATGIDRPIYSAGVSIGLKKSLVYYRGSAGK

GTKTDWMMHEFRLPP--ASMQEAEVWTICRIFRRTITYRKQQTPVPAVADSNSNTGSSEA

AAPIPQY-------PAAPVPEHAVAANDFYKPTGYDYR

>TraesCS5D01G472600.1

M-KSDEIIMSGFRFHPTDEELVSFYLKKKIQQKSISIELIRQLDIYKFDPWDLPKLASTG

ETDWYFYCPRDRKYRNSARPNGVTAAGFWKATGTDRPIYSSERCIGLKKSLVFYRGRAAR

GIKTDWMMHEFRLPSLTDNIPLNDSWTICKIFKKTSSMAQQMALSHTEQDLSSAM-ASNF

ASPNTSFQTQ-PSQHTAPPL-----------ITGADVN

>TraesCS5D01G481200.1

MAEAELNLPPGFRFHPTDDELVEHYLCRKAAGQRLPVPIIAEVDLYRFDPWALPDRALFG

TREWYFFTPRDRKYPNGSRPNRAAGNGYWKATGADKPVAPRGRTMGIKKALVFYAGKAPK

GVKTDWIMHEYRLADAGRGSLRLDDWVLCRLYNKKNEWEKMQLQQQGGEEMMVE--PKEE

EAPSEQQAAA----AQSPAAAMVPKKEAADEFVDLSYD

>TraesCS5D01G537600.1

MS--------G------------------------H-----------------------G

EGSCYFYSK-PAPVKEGKR--RMVGGGTWHPEAGAKPILGADSLVSNKRTFSYVKKS-PS

GQRTGWIMVEISLEK---QTGDADHMVLCVLYKSKKKYPDTSMAAAAAADAEPSVHAAEK

SAPPVEKEYPVPPTAVVPEVQPTRSDDSAMQNGG----

>TraesCS6A01G003800.1

M--EMAPLPPGYRFQPTDAELILYYLKRKILGKKLRPNPVTEIDIYQFAPWDLPGKSSMG

DLQWYFFCTCGRKYPTGSRTNRSNQAGHWKATGKDRKVVCNSRTVGMKRTLVFHAGKGRK

EKRTDWVMHEYRLVESEVAGVRLDDFVLCKVYQKSGPGPRI--EQYAEEEE-----GEAY

PGPRAQQ---VPSSFLEPVAGGHVPHGSSHEASG----

>TraesCS6A01G051700.1

MA-PSSALPPGCRFDPHDADLISAYLRPMIAGERLPASFLHSADVYAADPATLPAVSRTG

RRYWYFFGSAKARSGRDRRRSRVVGKGQWHSEKGRKVVSDEQNIGGYKQEFTYKPTNADG

SGTEVWLMVEFGVDQD--G---KSIPTLCKIYRSPRKPRSSTPSSTSMRKRKAGDTPLPA

QTPPEDH---MPFPP-VPESENMVLHGASVTNAGGDTT

>TraesCS6A01G057400.1

MAEAELNLPPGFRFHPTDEELVADYLCARAAGRAPPVPIIAELDLYRFDPWELPERALFG

AREWYFFTPRDRKYPNGSRPSRAAGGGYWKATGADRPVARAGRTLGIKKALVFYHGRPAA

GVKTDWIMHEYRLAGAA-ASLRLDDWVLCRLYNKKNQWEKMQRQRQEEEEAAAK--AAAS

ETPSDEL-------DSLPEFQAILPKEEVRELMGISLD

>TraesCS6A01G065600.1

MA-SSPVLPMGCRFSPSDADLISFYLRPMIAGEPLAARFLHTADAYGADPAALPALAKTG

RRCWYFFCSAKALSGHDKRRSRAVGEGTWHAEKGRAAVLDGE-VVGYKQSFRYKPIHSDG

SVEAVWLIVEFRVAHDQGG---ETVPVLCKVYQSPRKPRSASVTSPAGRKRKAGDDSAPV

EPPPEN--------P-VPEPV-TVLHGGSVTYAGGEST

>TraesCS6A01G065700.1

MA-SSSVLPMGCRFSPSDADLISFYLRPMIASEPLPARFLHTADAYATDPAALPALAKTG

RRCWYLFGPAKALSGHDKRRSRAVGEGTWHAEKGRAAVLDGE-VVGYKQSFRYKPIHSDG

SVEAVWLMVEFRMAHEQGG---ETVPVLCKVYQSPRKPRSASVTSPAGRKRKAGDDSAPV

EPPPENH---MPYHPVVPESE-MVLHGGSVTYAGGEST

>TraesCS6A01G108300.2

MSGSAPELPPGFRFHPTDEELVVHYLKKKAAKVPLPVTIIAEVDLYKFDPWELPEKATFG

EQEWYFFSPRDRKYPNGARPNRAATSGYWKATGTDKPILASGEKLGVKKALVFYRGKPPK

GLKTNWIMHEYRLTDASGASLRLDDWVLCRIYKKINKAAAGDQQRSSVEDA-----ATAG

PS---HHATSLSHLRASPAPTDASPAGILPQNVGADMA

>TraesCS6A01G146100.1

MEEHH------YRLSPTEVEAVTYYL-PRLSGETLQVKLIHRVEISGCEPKDLPVPQAVS

SGDRFFFTTCKSKNGSKLQSVRGAGAGTWSIQKTTEISH-AGVKVGEVRNLSFK----KK

GKSTGWVMEEYQCLLPEAG-----VKVFCKMHLAQHAPDAARQESAA-QQQQPE--STHA

-APKR-TRSDV---AQAPEISSSIARSTSEEEDG-DLE

>TraesCS6A01G208900.3

VADIQPGLPAGVKFDPSDQELLEH-LEEKIVGHMF-IEFIPTVGICYSHPENLPGTNKDG

-SSAHFFHSISNAYGCGQRKRRRISNSRWHKTGKSKPVYDNGVMKGWKKILVLYKGSGGK

PDKTDWVMHQYHLGVEEN---KVGEFVVSKIFYQLKTRPVD--KSEANEESTAFTANSPC

NP---RHLGTAPTDVTSQAGTDVAFHAGTSLSQGPDLQ

>TraesCS6A01G237900.1

M-DTFSHVPPGFRFHPTDEELVDYYLRKKVASKKIDLDVIKDVDLYKIEPWDLQDKCKIG

QNDWYFFSHKDKKYPTGTRTNRATSAGFWKATGRDKPIYTNHCLVGMRKTLVFYKGRAPN

GQKSNWIMHEYRLETNENGATPEEGWVVCRVFKKRVATVRRMADMNS--DHGAGGGSPRQ

NL---EYMQQLPHL-ESPKPTPLAHDGPSRFSSATDWR

>TraesCS6A01G299500.1

MAEEEAAIPPGFRFRPSDDELIRYYLLPKLQGRGAPNRAIIEHNVYQCHPDELTGKYRGG

GKSFYFLSPRVRRYDNGDRPRRDTGRGRWKVSTGKKDVAGDGTTRYCMSVLNYFESPRGG

GRKSEWLMRELTVPAPKDGGKTLDRYVMCKIYLKDDKGDDDEEAGPSSASPLLLHGAQDQ

SGPAY---------QKQPETDQQQQQLAMASYLGADYY

>TraesCS6A01G299600.1

MAEEEDGMPPGFRFMPTEDEMIRYYLLPKLQGRAVPNNAIIEDTVYQCHPDGLTSKYMDG

GRSFYFLSPRERKYKNGARPRRDTRLGRWKASTGKTDVAGDGTTKYCKSGLAYFRGRGEK

EKKSGWLMQELTVPEPEKGGPTLDRYVMYKIYPAPGKRNNDDEAGPSNAELVESDDSEGQ

SGPAY-----VPQ-QKQPETD----------YFGADCH

>TraesCS6A01G307200.1

M-KQDEVMLPGFRFHPTDEELVRFYLKRKIQQKSLPIELIRQLDIYKFDPWDLPKLASTG

EKEWYFYCPRDRKYRNSTRPNRVTGAGFWKATGTDRPIYSSDKCIGLKKSLVFYKGRAAK

GVKTDWMMHEFRLPSLTDTIPPNDSWAICRIFKKTNATAQR--LSHSGA-------RSRH

SSPIIDS---LPAGNASPITF----------NN-VDLP

>TraesCS6A01G378500.1

M-DIEMTLPPGFRFYPSDEELVCHYLLSKVANQRLAAGTMVEVDLHVHEPWELPDVAKLT

TNEWYFFSFRDRKYTTGLRTNRATRSGYWKATGKDRVIRSLRAIVGMRKTLVFYRGRAPN

GIKTCWVMHEFRIENP--HSPPKADWVLCRVFHQKKADTEYAMDGKQAHKAAAVSGSSSS

PSPLGHHHSN----AGMPQLLAGLRDGADDQDLGYNYN

>TraesCS6A01G378600.1

M-DIEMTLPPGFRFYPSDEELVCHYLHGKVANQRFAAGTMVEVDLHVHEPWELPDVAKLS

TNEWYFFSFRDRKYATGLRTNRATRSGYWKATGKDRVIRSPRAIVGMRKTLVFYRGRAPN

GSKTCWVMHEFRIENP--HAPPKEDWVLCRVFHKKKADTEYAMDGEQARSAAAVSGSSSA

PTSLGHHLAD----ADMPPLTAGLRDGAGDQDLGYNYN

>TraesCS6A01G392900.1

MA-----MAPAFKFDPTDADLVAHYLLPRALGVPPPAHAVIDDDPAGLPPADLFAKH--G

SQHAFFLHTADT---DAPESKRAVGGGRWRGQKASVEVTHPDLDMKYRSELTYE---AGN

GDATGWVMHEYQIVSPPL-----QATVLSRISKVRE-----DQAAA---QN---------

--------------------------------------

>TraesCS6A01G406700.1

MSAEEVPMAPGFRFHPTDEELVSYYLRRRVLGRRLRIDAIAEVDLYRLEPWDLPPLSRIR

DAQWYFFARLDRKVAGGNRTNRATPRGYWKTTGKDREVFHRGRAVGMKKTLVFHAGRAPK

GDRTNWVMHEYRLLDNDG---PQDMHVVCRIFQKVGSGPQN--AQYAMEEEWENEDTSTE

ESPQASYLSGCPNQFSDPSNGTNAYSGQQQAENGNDFS

>TraesCS6B01G033000.1

M--EVAPLPPGYRFQPTDVELILYYLKRKILGKKLRRNAVTEIDIYKFAPWDLPGKSSMG

DLQWYFFCTCGREYPTGSRTNRSNQAGHWKATGKDRKVVYNSRTVGMKRTLVFHAGKGRK

EKRTDWVMHEYRLVESEIAGVRLDDFVLCKVYQKSGPGPKI--EQYAEEEE-----GEAY

PGPRAQQ---VPSSLLEPVAGGHVPDGSSQASSGMD--

>TraesCS6B01G075200.1

MAEAELNLPPGFRFHPTDEELVADYLCARAAGRAPPVPIIAELDLYRFDPWELPERALFG

AREWYFFTPRDRKYPNGSRPNRAAGGGYWKATGADRPVARAGRTVGIKKALVFYHGRPSG

GVKTDWIMHEYRLAGAG-SSLRLDDWVLCRLYNKKNQWEKMQQQRQ-EDEAAAK--AAAS

ETPSDEL-------DSLPAFQAILPKEEVRELMGISLD

>TraesCS6B01G088700.1

MA-STPILPMGCRFSPSDADLISFYLRPMIASEPLPARFLHTADAYAADPSSLPALARTG

RRCWYFFGPAKALSGHDKRRSRAVGKGTWHAEKGRVAVLDGEGVVGYKQSFRYKPSDADG

SVEAVWLMVEFRVAHDQGG---ETVPVLCKVYQSPRKPRSASITSPAERKRKARDDSAPA

EPPPENH---TPFHPVVSESE----------YAGGDTT

>TraesCS6B01G237900.3

VADIQPGLPAGVKFDPSDLELLEH-LEQKIVGHMF-IEFIPTVGICYSHPENLPGTNKDG

-SSVHFFHSISNAYGCGQRKRRRISNSRWHKTGKSKPVYDNGVMKGWKKILVLYKGSGGK

PDKTDWVMHQYHLGVEEN---KVGEFVVSKIFYQLKPRQVD--KSEANEESNAFTSNSPC

NP---HHLGTAPTDVTSQAGTDVASHAGTSLSQGPDLQ

>TraesCS6B01G286200.1

M-DTFSHVPPGFRFHPTDEELVDYYLRKKVASKKIDLDVIKDVDLYKIEPWDLQDKCKIG

QNDWYFFSHKDKKYPTGTRTNRATSAGFWKATGRDKPIYTNNCLVGMRKTLVFYKGRAPN

GQKSDWIMHEYRLETNENGATPEEGWVVCRVFKKRVATVRRMADMNS--DHGAGGGSPRQ

NL---EYMQQLPLL-ESPKPTPLAHDGASRFSSATDWR

>TraesCS6B01G323600.1

MA---LGMPPAFKFDPTDGDIVAYYLLPRALGLP-PAHAIIEEDPGSAPPWELLRRH--G

IEHAFFFGPPT----DGGRRRRTVGGGVWQGQKLNEGVTGPGLDIAYRYDLTFKLK-AGR

GVSTGYVMHEYEITSPPL-----PGKVLTRVNISSNAKKEK--AATAGADQQVMVPASNG

GGP-----------CVLPDGE----------FTGVETN

>TraesCS6B01G323700.1

MA---PGMPPAFKFDPTDGDIVACYLLPRALGLP-PAHAIIEEDPGSAPPWELLRRH--G

IEHAFFFGPPT----GGGRRRRTVGGGVWQGQKLNEGVTGPGLDIAYRYDLTFKLK-AGR

GASTGYVMHEYEITSPPL-----PGKVLTRVNISSNAKKEK--AATAGADQQVMVSASNG

GGP-----------CVLPDGE----------FTGVGTN

>TraesCS6B01G329200.1

MAEEEAGIPPGFRFRPSDDELIRYYLLPKLQGRGAPNRAIIEHNVYQCHPDDLVGKYRGG

GKSFYFLSPRVRRYDNGDRPRRDTGRGRWKVSTGKRDVAGDGTTRYCMSVLNYFESPRGG

GRKSEWLMRELTVPAPKDGGRTLDRYVMCKIYLKDDKGDDDEEAGPSSASPLHLHGAEDQ

SGPAY---------QKQPETDQQQQQLAMASHLGANYY

>TraesCS6B01G335700.1

M-KQDEVMLPGFRFHPTDEELVRFYLKRKIQQKSLPIELIRQLDIYKFDPWDLPKLASTG

EKEWYFYCPRDRKYRNSTRPNRVTGAGFWKATGTDRPIYSSDKCIGLKKSLVFYKGRAAK

GVKTDWMMHEFRLPSLTDTIPPNDSWAICRIFKKTNATAQR--LSHSGA-------RSRH

SSPIIDS---LPAGNASPITL----------NSNVDLP

>TraesCS6B01G416300.1

M-DIEMTLPPGFRFYPSDEELVCHYLHGKVANQRFATGTMVEVDLHVHEPWELPDVAKLS

TNEWYFFSFRDRKYATGLRTNRATRSGYWKATGKDRVIRSPRAIVGMRKTLVFYRGRAPN

GSKTCWVMHEFRIENP--HSPPKEDWVLCRVFHKKKADTEYAMDGEQTRNATAVSGSSSS

PPPLGHHLTD----AGMPPLLAGLRDGAGDQDLGYNYN

>TraesCS6B01G416400.1

M-DIEMTLPPGFRFYPNDEELVCHYLQSKVANQHFAAGTMVEVDLHVHEPWELPDVAKLS

ANEWYFFSFRDRKYATGLRTNRATRSGYWKATGKDRVIRSPRTIVGMRKTLVFYRGRAPN

GTKTCWVMHEFRIENP--HSPPKEDWVLCRVFHKKKADTDYAMDGKA-----AVSGSSSS

PMPIGQHVSD----AVMPQLLACLRDGGDDQ---YKYN

>TraesCS6B01G416500.1

M-DIEMMLPPGFRFYPSDEELVCHYLHGKVANQCFATGTMVEVDLHVHEPWELPDVAKLS

TNEWYFFSFRDRKYATGLRTNRATRSGYWKATGKDRVIHSPRAIVGMRKTLVFHRGRAPN

GTKTCWVMHELRTLTP--H--PRRTGC-CAEFSTRRKPTLST-----SRSSAAVAW----

--TL------------LP--------------------

>TraesCS6B01G433100.1

MA-----TAPAFKFDPTDADLVAHYLLPRAVGVPPPAHAIIENDLAGLPPADLLARH--G

SHHAFFMHTAA----DAPERERGVGGGRWRGQKASVEVTHPDLDMKYRSELSYE---AGE

GDATGWVMHEYQIVSPPL-----QSTVLSRISKVRE-----DQAAAA--QN---------

--------------------------------------

>TraesCS6B01G451300.1

MSAEEVPMAPGFRFHPTDEELVSYYLRRRVLGRRLRIDAIAEVDLYRLEPWDLPPLSRIR

DAQWYFFARLDRKVAGGNRTNRATPRGYWKTTGKDREVFHRGXXXXXXXXXXXXXXXXXX

XXXXXXXXXXXXXXXXXA----QDMHVVCRIFQKVGSGPQN--AQYAMEEEWEDEDTSTE

ESPQVSYLSGCHNQWSDPSIGANAYSGQQQAEVGDDFS

>TraesCS6D01G059300.1

MAEAELNLPPGFRFHPTDEELVADYLCARAAGRAPPVPIIAELDLYRFDPWELPERALFG

AREWYFFTPRDRKYPNGSRPNRAAGGGYWKATGADRPVARAGRTVGIKKALVFYHGRPSA

GVKTDWIMHEYRLAGADGGTLRLDDWVLCRLYNKKNQWEKMQRQRQ-EEEAAAK--AAAS

ETPSDEL-------DSLPEFQSILPKEEVQELMGISLD

>TraesCS6D01G096300.1

MSGSAPELPPGFRFHPTDEELVVHYLKKKAAKVPLPVTIIAEVDLYKFDPWELPEKATFG

EQEWYFFSPRDRKYPNGARPNRAATSGYWKATGTDKPILASGEKLGVKKALVFYRGKPPK

GLKTNWIMHEYRLTDASGASLRLDDWVLCRIYKKINKAAAGDQQRSSVEDA-----ATAG

SS---HHATSLSHLRASPAPTEASAAGILPQNVGADMA

>TraesCS6D01G135400.1

MEDHH------YRLSPTEVEAVTYYL-PRLSGETLHVKLIHRVDITGCEPKDLPVPQAMS

SGDRFFFTTCKSKNGSKLQSVRSAGGGTWSIQKTSEISH-AGVKVGEVKNLSFK----KK

GKSTGWVMEEYRCLLPEAG-----VKVFSKMHLAQHAPDAARQESEA-QQQQPE--STHA

-APKR-THSDV---AQAPEISSSIARSTSEEEDG-DLE

>TraesCS6D01G140200.1

MC------PPGMNYSWSDEELVRF-LAERKADDSLPENVLVGMDLTLIHPRDSP-----G

-NIWYLNQSDDQPYGNGESDIRKAKGGYWKCIDVLRI--TSKSTAGVKFSLEFYEGEAPS

GKRTQWLMHEYQVEQNDD----QEYKSLCTIFMQGSKTEDESLSTNAADEQNVAVNSSRE

SSP-----------HHPPASSSGSSHGSTTSKLG----

>TraesCS6D01G192400.1

VADIQPGLPAGVKFDPSDLELLEH-LEQKIVGHMF-IEFIPTVGICYSHPENLPGTNKDG

-SSAHFFHSISNAYGCGQRKRRRISNSRWHKTGKSKPVYDNGVMKGWKKILVLYKGSGGK

PDKTDWVMHQYHLGVEEN---KVGEFVVSKIFYQLKTRPVD--KSEANEESTAFTANSPC

NP---SHLGTAPTDVTSPAGTDVASHAWTSLSQGPDLQ

>TraesCS6D01G220600.1

M-DTFSHVPPGFRFHPTDEELVDYYLRKKVASKKIDLDVIKDVDLYKIEPWDLQDKCKIG

QNDWYFFSHKDKKYPTGTRTNRATSAGFWKATGRDKPIYTNHCLVGMRKTLVFYKGRAPN

GQKSDWIMHEYRLETNENGATPEEGWVVCRVFKKRVATVRRMADMSS--DHGAGGGSPRQ

NL---EYMQQLPHL-ESPKPTPLAHDGPSRFSSATDWR

>TraesCS6D01G266000.1

--------------------------------HPL-ILFISTIGICYTHPEKLPGITLSG

-LSKHFFQRNSRAFKRGTWTRRKIQSEMWHKTGNTLPVVVNGRQMGSKKVLVLHTNKNFD

QQRTN-------------------------------------------------------

--------------------------------------

>TraesCS6D01G279400.1

MAEEEAGIPPGFRFRPSDDELIRYYLLPKLQGRGAPNRAIIEHNVYQCHPDELVGQYRGG

GKSFYFLSPRVRRYDNGDRPRRDTGRGRWKVSTGKKDVAGDGTTRYCMSVLNYFESPRGG

GRKSEWLMRELTVPAPKDGGKTLDRYVMCKIYLKDDKGDDDEEAGPSSASPLHLHGAEDQ

SGPAY---------QKQPETDTQQQQLAMASYLGANYY

>TraesCS6D01G279500.1

MAEEEDGMPPGFRFMPTEDEMIRYYLLPKLQGRAVPNNAIIEDSVYQCHPDGLTSKYMDG

GRSFYFLSPRERKYKNGVRPRRDTRLGRWKASTGKTDVAGDGTTKYCKSGLAYFRGRGEK

EKKSGWLMQELTVPEPEKGGPTLDRYVMYKIYPAPGKRNNDDEAGPSNAEAAESDDSEDQ

PGPAY-----VPL-QKQPETD----------YFGADCN

>TraesCS6D01G286300.1

M-KQDEVMLPGFRFHPTDEELVRFYLKRKIQQKSLPIELIRQLDIYKFDPWDLPKLASTG

EKEWYFYCPRDRKYRNSTRPNRVTGAGFWKATGTDRPIYSSDKCIGLKKSLVFYKGRAAK

GVKTDWMMHEFRLPSLTDTIPPNDSWAICRIFKKTNATAQR--LSHSGA-------RSRH

SSPIVDS---LPAGNASPITL----------NSNVDLP

>TraesCS6D01G362800.1

M-DIEMTLPPGFRFYPSDEELVCHYLHGKVANQRFATGTMIEVDLHVHEPWELPDVAKLS

TNEWYFFSFRDRKYATGLRTNCATRSGYWKATGKDRVIRSPRTIVGMRKTLVFYRGRAPN

GSKTCWVMHEFRIENP--HSPPKEDWVLCRVFDQKKADTEYAVDGKQAQKAAAVSGSSSS

PSPLGHHLSN----AGMPQLLAGLRDGADDQDLGYNYN

>TraesCS6D01G362900.1

M-DIEMTLPPGFRFYPSDEELVCHYLHGKVANRRLAAGTMVEVDLHVHEPWELPDVAKLS

TNEWYFFSFRDRKYATGLRTNRATRSGYWKATGKDRVIRSPRAIVGMRKTLVFYRGRAPN

GSKTCWVMHEFRIENP--HSPPKEDWVLCRVFHKKKADTEYTMDGEQARSAAAVSG---S

PPPLGHHLTD----VDMPQLLAGLRDGAGDQDLGYNYN

>TraesCS6D01G390200.1

MAAEEVPMAPGFRFHPTDEELVSYYLRRRVLGRRLRIDAIAEVDLYRLEPWDLPPLSRIR

DAQWYFFARLDRKVAGGNRTNRATPRGYWKTTGKDREVFHRGRAVGMKKTLVFHAGRAPK

GDRTNWVMHEYRLLDNDG---PQDMHVVCRIFQKVGSGPQN--AQYAMEEEWEDEDTSTE

ESPQASHLSGCPNQFSDPSNGTNAYSGQQQAENGNDFS

>TraesCS7A01G000300.1

MTTAAAVLPVGFRFRPTDEELVRHYLKAKIAGRAPDLLAIPDVDLAAVEPWDLPARSVIK

DPEWFFFARRDRKYPKSSRSCRSTAAGYWKATGKDRLIRAPGALIGVKKTLVFHRGRAPR

GARTPWIMHEYTATDPNPAGPQNDSFVLYRLFNKQDEETPAPVSEPPSTSPPA---ASMV

TAPAIQEQQGIAARASQPILAAGCSTESSSNMAGHNIT

>TraesCS7A01G008800.1

M-STQPQVPPGFRFHPTDEELVDYYLRKKVASRRIDLNVIKDVDLYKIEPWDLQEKCRIG

QSDWYFFSHKDKKYPTGTRTNRATAAGFWKATGRDKPIYAKHCLVGMRKTLVYYKGRAPN

GQKSDWIMHEYRLETNENGPPQEEGWVVCRVFKKRLPTTRRDLDHDAVDDDGPFM-SPMR

SM---QYLNTIPHELESPSSFVSPDDHQINVQAATDWR

>TraesCS7A01G068000.1

MSNGQSVVPPGFRFHPTEEELLTYYLAKKVASQRIDLDVIPDVDLNKLEPWDIQERCRIG

QNDWYLFSHKDKKYPTGTRTNRATAAGFWKATGRDKAIYSATGRIGMRKTLVFYKGRAPH

GHKSDWIMHEYRLDDAVPSSAQEDGWVICRVFKKKNIVVNQ--GQNGASNKLAGVASQSN

SS-QQLHTAPCKQE-TKPA--TACPNG--TLEYGTDWD

>TraesCS7A01G068200.1

MSNGQSVVPPGFRFHPMEEELLTYYLTKKVAPQRMDLDVIRDVDLTKLEPWDIQELCRIG

QNDWYLFSHKDKKYPTGTRTNRATAAGFWKATGRDKAIYSAASRIGMRKTLVFYKGRAPH

GHKSDWIMHEYRLDDATTSSAQEDGWVICRLFKKKT----------------------SP

ST------------------------------------

>TraesCS7A01G068300.1

MSNGQSVVPPGFRFHPTEEELLTYYLAKKVASQRIDLDVIRDVDLNKLEPWDIQERCRIG

QNDWYLFSHKDKKYPTGTRTNRATAAGFWKATGRDKAIYSAAGRIGMRKTLVFYKGRAPH

GHKSDWIMHEYRLDDAA-PSAPEDGWVICRVFKKKNIVVQRQAGQNGAFSKLVGAGSQSN

SS---MHTASCRQE-TKPTN--ACHSGSSTLEYGGDWD

>TraesCS7A01G106300.1

MERLVLSLPPGYHFAPTDMELIVHYLRRKMDGHPPHLPIFKDVPITDYRPEQITEVFGCG

EERWYFFTKRTRKYATGNRPDRTTGRGYWKATGPQRLIRTGPPLVGRRRTLVFYTGP-DE

AAMTAWTMYEYENLTSEENADKLGEWVLCTIQRQKSQRSADKGESKAKGKAQKGAD----

ASPMMKHYSTTPYQ---PATS----------YAG----

>TraesCS7A01G152400.1

M-RQQLKFPQGFRFHPTNVEIITSYLVPKVLNKAFDPIAVGEVDLNKCEPWELPEKAKMG

------------------------------------------------------------

------------------------------IFHKSTGLKKVVMSSYAAEEQHGFLESSLV

GAPMNHHQIGADEGGVEPGSGVATTADEISSNMGTDGM

>TraesCS7A01G152500.1

M-RQQLKFPQGFRFHPTDVEIITSYLVPKVLNKAFDPIAVGEVDLNKCEPWELPEKAKMG

EKEWYFFSQKDRKYPTGIRTNRATTAGYWKATGKDKEIFHHASLIGMKKTLVFYKGRAPR

GEKTNWVMHEYRLESGKQNASSKEEYVVCRIFHKSTGLKKVVMSSYAGEEQHGFLESSLV

GALMNHHQIGADEGGVEPGSGVATTAEEISSNMGTDGM

>TraesCS7A01G189200.1

M-QQQLNLPPGFRFQPTDMEIITFYLVPKVLKKVFDTTVVKEVDLNKCEPWDLLNKVNMG

EKGRYFFSQKGLKYSTGIRTNRATKAGYWKATGKDKEIIHPPSIIGMKKTLVFYKGRAPK

GEKTNWIMHEYRLKSGKQNASSKGEYVVCRIFHKSTGLKKVMMPSSDEEKQQSFLKSSLA

EAPMNHYEMSVDQGGVDPGYGGIATSGEILSNMGIDGM

>TraesCS7A01G194700.1

M-QQHLNLPAGFRFHPTDMEIITFYLVRKVLKKPFDVTVIEEVDLNKCEPWDLQNNVNMG

EKDQYFFSKKDLKYPTGVRTNRATNAGYWKATGKDKEIVHPPSLIGMKKTLVFYKGRAPR

GEKTNWIMHEYRLEMGKQNASSKEEYVICRIFHKSTGLRKVVMPSYSVEQQQGFLESSLA

GAPMNHHHMSAEQGGVDPGSGVATISSEISSNMGMDGM

>TraesCS7A01G204300.1

MEDQH------YRLNPTDVDAVTYYL-PRLAGQP-HAKFIHHVDIYSCEPKDLPVPQAAS

SGDRFFFTTRKSKNGSKTQSVRTAGGGTWTVNATTAVKH-AGVEVGERKNLSFR----KK

GKSTGWVMEEYRCLLPKAG-----VKVFCKIHLAQHPPDAARQESAA-QEPQRE--STHA

-APKR-MHSDVASSAQAPAISSSIARSTSEEADG-DLE

>TraesCS7A01G208200.1

IADHQPGLPKGVKFDPSDQELLGH-LLAKHKAHPF-IEFIPTVGICYTHPQKLPGVKQDG

-SISHFFHRTFKAYNTGTRKRRKINTSRWHKTGKTKPVMVDGQHLGCKKIMVLYMSTGGK

PKKTNWVMHQYHLGTGED---QNGEYVVSKLFFQQQNGEKS--AL--NTDVMQT--AEEE

-------GDNPPSQ--DPNPWAQLPVEDL-----KDLQ

>TraesCS7A01G209100.1

MEDHH------YRLSPTDVDAVTYYL-PRLSGETLHVKLIHRVNISGCEPKDLPVPQAVS

SGDRFFFTTCKSKNGSKLQSVRAAGGGTWTIQKTTEIFH-AGVKVGEVKNLSFK----KK

GKSTGWVMEEYRCLLPEAG-----VKVFCRMHLAQHAPDAARQESAA-QQEQPE--STHA

-APKR-MRSDV---AQAPEISSSIARSSSEEADG-DIE

>TraesCS7A01G209200.1

MEDHH------YRLSPTEVDAVTYYL-PRLSGETLHVKLIHRVEISGCEPKDLPVPQAVS

SGDRFFFTTCKSKNGSKLQSVRGAGGGTWTIQKTTEICH-AGVKVGEVKNLSFK----KK

GKSTGWVMEEYRCLLPEAG-----VKVFCKMHLAQHAPDAARQESEA-QQQQPE--STHA

-APKR-M-------AAAPD--DDMDRFCCTI----DEL

>TraesCS7A01G209300.1

MEDHH------YRLSPTEVEVVTYYL-PRLSGETLHVKLIHRVNISGCEPKDLPVPQAIS

SGDRFFFTTCKSKNGSKLQSVRGAGGSTWTIQKTKEICH-AGVKVGEVKNLSFK----KK

GKSTGWVMEEYRCLLPEAG-----VKVFCKMHLAQHAPDAARQESEA-QQPQPE--STHA

-A------------AAAPV--DDMARFCCTI----DEL

>TraesCS7A01G209500.1

MEDHH------YRLSPTEVDAVTYYL-PRLSGETLHAKLIHRVEISGCEPKDLPVPQAVS

SGDRFFFTTCKSKNGSKLQSVRGAGTGTWTIQKTTEICH-AGVKVGEVKNLSFK----KK

GKSTGWVMEEYRCLLPEAG-----VKVFCRMHLAQHAPDAARQESAA-QQQQPE--CTHA

-APKR-MHSDV---AQAPEISSSIARSSSEEADG-DLE

>TraesCS7A01G247600.1

M-AGEHGLPPGFRFHPTDEELVTFYLAAKVFNGACCGGDIAEVDLNRCEPWDLPEAARMG

EREWYFFSLRDRKYPTGLRTNRATGAGYWKATGKDREVLNAASLLGMKKTLVFYRGRAPR

GEKTKWVLHEYRLDGDFGRRSCKEEWVVCRIFHKAVDPYSKMMEMRNSPHHSFFQDQSQG

SSPAAAY-------PQEPPPT----------EMG----

>TraesCS7A01G263100.1

MEEVLRQLPPGFRFRPTDEELVVQYLRRKALALPLPAAVIPNVNLYSLDPWDIP-----G

EGEKYYFAVRPAGAKRGGR--TTTASGCWKASARERPVVVSRHLVGVKKSMVFVPRRAPA

PAQTGWVMHEYRLALPHHAGAGTEEWVVCRIFQRDRSSSSSNIQTPGGTDARRTM-SSSS

SS------------------------------------

>TraesCS7A01G299600.3

M--ELKALPLGFRFHPTDEELVRHYLKGKITGQINEVEVIPEIDVCKCEPWDLPDKALIR

DPEWFFFAPKDRKYPNGSRSNRATEAGYWKATGKDRVIKSKGHMIGMKKTLVFHRGRAPK

GERTGWIMHEYRTTEPEFESGEQGGYVLYRLFQKQLEKTERDRSGYSNEEAITLINGNSG

GSPNSQYSADVDDSRSVPSDCSSSTDSSFNTNEGVDTN

>TraesCS7A01G305200.1

M--MEARMPPGFRFHPRDEELVLDYLLHKLTGRRYGGVDIVDVDLNKCEPWDLPEAACVG

GREWYFFSLRDRKYATGQRTNRATRSGYWKATGKDRAILAHGALVGMRKTLVFYQGRAPK

GTRTEWVMHEFRLEEERHRCQLKEDWVLCRVFYKSRTSSPRPPSEEVC-TFFSELDSGTA

RGPK---SNSNSKLELTPNWS-----------NG----

>TraesCS7A01G317100.1

MSNGQSCVPPGFRFHPTEEELLNYYLRKKVASEEIDLDVIRDVDLNKLEPWDIQEKCKIG

QNDWYFFSHKDKKYPTGTRTNRATAAGFWKATGRDKAIYNAVKRIGMRKTLVFYKGRAPH

GQKSDWIMHEYRLDDPSSDAGQEDGWVVCRVFKKKHHHKDAGAGNGKRDDG-----GKAA

SSP--LY---CKQESPRPRT-----------RDGTDWA

>TraesCS7A01G318500.1

M-EHVETVMPGFRFHPTEEELIEFYLRRKVDGKRFNIDLIASVDLYRYDPWDLPALASIG

DKEWFFYVPRDRKYRNGDRPNRVTPSGYWKATGADRMVKVVERSIGLKKTLVFYVGKAPK

GLRSSWIMNEYRLPHGET-ERYQKEISLCRVYKRPGIDDNFHLTGTTASRAAATRHSSSA

AAP----TTSTEEDHNNPAVMLNSNSSAMAT---IPID

>TraesCS7A01G326000.1

M-SSCNTVPPGFRFHPTEEELVGYYLARKVSSHKIDLDIIQEVDLYRIEPWDLQERCGKG

TTEYYFFSYKDRKYPSGTRTNRATAAGFWKATGRDKPVLSSSAVIGMRKTLVFYRGRAPN

GRKTDWIIHEYRLQSNEHAPTQEEGWVVCRAFVKPVPSQQHRLSYGGASDN-----AGGG

--PHDQHPSDLPPPLQSPTFDGGCSSADQQLAAGIDWN

>TraesCS7A01G334800.1

M-GGGGDLPPGFRFHPTDEELITYYLLRKVVDCGFSARAIAEIDLNKCEPWELPDKACK-

EKEWYFYSLRDRKYPTGLRTNRATGAGYWKATGKDREIRSARALVGMKKTLVFYRGRAPK

GQKTQWVMHEFRLEGVYANNTTRDEWVIAKIFVKPGAVPPSRKARYSSADTSCFSDASSA

SSPFSQYAPRLVQAMFLPGGLLGIGGGAFQHSSGYEIK

>TraesCS7A01G349500.1

M-GGALSVPPGFRFHPTDEELLYYYLRKKVAYEAIDLDVIREIDLNKLEPWDLKDRCRIG

QNEWYFFSHKDKKYPTGTRTNRATTAGFWKATGRDKAIFLGSRRIGMRKTLVFYVGRAPH

GKKTDWIMHEYRLDED--VEVQEDGWVVCRVFTKKSYTRGMNPAEMADDDELLHPFSAAG

HNPHLQHSMQLPQLSFLPGA-KLTSSGTIGMFPGTDWS

>TraesCS7A01G357500.1

TA-LDVGLPAGVKFDPTDQELIEH-LEAKVSGHPL-IEFIPTIGICYTHPEKLPGVSKDG

-LSRHFFHRPSKAYTTGTRKRRKIQPPRWHKTGKTRPVAVGGRQRGCKKILVLYTNFHRK

PEKTNWVMHQYHLGESEE---REGELVVSKIFYQTQPRQCG--V------GDGASAGAGR

NI---HHHVSTPTDIAAPPVHSRRSKGGETS--GEDTE

>TraesCS7A01G375300.1

MADNRHGFPHGYHFVPDDAELLRL-LEDMIAGRALPLSIFHGVRIRNYHPAELHELYKA-

AGSIYFYNQR--EFS-GSRPGRTAKDGWWKASGGGLPLVRRGLVVGYKLTLVFYEKRPKP

DQKTDWIIKEYTIAG---PNRKASEMALYRLYNKSENRSKEKKKAKEQEEENTFASGQPR

AGPSSQHPPPAPAAAASPAFHQVAASPAFDMFAAKDIP

>TraesCS7A01G464100.1

M--VEARLPPGFRFHPRDDELVLDYLAKKLGGGGYGCPTMVDVDLNKIEPWDLPEIACIG

GKEWYFYSLRDRKYATGQRTNRATESGYWKATGKDRSISRKGLLVGMRKTLVFYQGRAPK

GKKTEWVMHEFRKEGQGDKLPLKEDWVLCRVFYKTRATVAKPPTGSSYNESAAATSSTVQ

ASPAA--LTRFEQGREAPAQG----------QDGFEYL

>TraesCS7A01G464800.1

M--VEARLPPGFRFHPRDDELVLDYLSRKLGGGAYGCPAMVDVDLNKIEPWDLPEIACIG

GKEWYFYSLRDKKYATGQRTNRATESGYWKATGKDRAISRKGLLVGMRKTLVFYEGRAPK

GKKTEWVMHEFRKEGQGDKLPLKEDWVLCRVFYKTRTTIAKPSTGSNYNDSAAATSSTVQ

SSPAA--LTRFEQGREAPAQG----------QDGFEYL

>TraesCS7A01G549000.1

ME-----LLPGFRFHPTEEELLEFYLKHHVTRNNAPFDIIPTVHLYRHDPWDLPGLAAIS

EREWYFFVPRDRKHASGGRPSRTTERGFWKATGSDRAVRCAARLVGLKKTLVYYQGRAPR

GTKTDWVMNEYRLPDLAGGAGEQQDVVLCKVYRKAVSLKELEQRVA-MEELARTRSGSPD

EA---HH-------VARPPAM-----------------

>TraesCS7A01G569100.1

M-QKQLVLPPGFRFHPTDEEIIKFYVVPKVLDEAFVAAAIEDVNLNKYEPWELPEKAKMG

EKEWYFYSRKDRKYPTGIRTNRATETGYWKATGKDKEIFQPPKLIGMKKTLVFYKGRAPR

GEKTNWIMHEYRLESSKKNMASKEQWVVCRIFHKSTGLKKMVTPSYDAEHQQGFVDSTCA

GTPMNHQHIGADQGGAQPGGGAATAVGETSLNMGMDDI

>TraesCS7A01G569300.1

M-QQKLELPPGFRFHPTDEEIIKFYVVPKVLDEAFVAAAIEDVNLNKYEPWELPEKAKMG

EKEWYFYCRKDRKYPTGIRTNQAKNAGYWKATGKDKEIFHPPTLIGMKKTLVFYKGRAPR

GEKTNWIMHEYRLESNKQNASSKEQWVVCRIFHKSAGLKKVVMPSYVAEHQQGFADSSLA

GAPMNHHYVGADRGGAEPGCGAATAVGKTSSNMGMDDM

>TraesCS7B01G004900.1

MERLVLSLPPGYHFAPTDVELIVHYLRRKMDGHPPHLPVFKDVPITDYRPEQITEVFDCG

EERWYFFTKRTRKYATGNRPDRTTGRGYWKATGPQRLIRTGPPLVGRRRTLVFYTGP-DE

AAKTAWTMYEYENLTSEENADKLGEWVLCTIQRQKSQRVGDKGESKAKGKAQKGAD----

ASPLMQHYSTTPYQ---PATS----------YAG----

>TraesCS7B01G056300.1

M-RQQLKFPQGFRFHPTDVEIITSYLVPKVLNKAFDPIAIGEVDLNKCEPWELPEKAKMG

EKEWYFFSQKDRKYPTGIRTNRATTSGYWKATGKDKEIFHHVSLIGMKKTLVFYKGRAPR

GEKTNWVMHEYRLECGKQNASSKEEYVVCRIFHKSTGLKKVVVSSYAAEEQHAFHESSLV

GAPMNHHQIGADEGGVEPGSGVATTADEISSNMGTDDI

>TraesCS7B01G077700.1

MADPALALPPGVYFNPTNEEIVRTYLNGWIAGHGTEAAVVIEDDVYGDQPDVLPASTRDS

DPSWWFRCHC-KQATRGGRGDRGVTGGFWKLEQTTKEVRCEAEHLGFKRTFGFYVEHEGE

KEKTRWLMEEYTNVDSPDG---RNLPALYRIYLTPRDPGKKRRKTSGGDDH-----AARV

SSP--EY-------DDSPELRVTMDD-GMPAEMG----

>TraesCS7B01G094000.1

M-QKLLNLPPGFRFQPTDMEIITFYLVPKVLKKVFNTMVVGEVDLNKCEPWDLLKKVSMG

DKGRYFFSQKDLKYSTGIRTNRATKAGYWKATGKDKEIVHPPSIIGMKKTLVFYKGRAPK

GEKTNWIMHEYRLESGKQNASSKGEYVICRIFLKSIGLKKVMMPSYDGEQQQGFLESSLA

EAPMNHY-------------------------------

>TraesCS7B01G100300.1

M-QKHLNLPAGFRFHPTDLEIITFYLVRKVLKKPFDVIVIEEVDLNKCEPWDLPNNVNMG

EKDQYFFSKKDLKYPTGVRTNWATNAGYWKATGKDKEILHPPSLIGMKKTLVFYKGRAPR

GEKTNWIMHEYRLEMGKQNGSSKEEYVVCRIFHKSTGLKKVVTPSYSAEQQQGFLESSLE

GAPMNHRHMSAEQGGVDPGSG-----------------

>TraesCS7B01G111300.1

MEDQH------YRLNPTDVDAVTYYL-PRLAGQTLHAKFIHHVDIYSCEPKDLPVPQAAS

SGDRFFFTTRKSKNGSKTQSVRTAGGGTWTVNATTAVKH-AGVEVGERKNLSFR----KK

GKSTGWVMEEYKCLLPEAG-----VKVFCKIHLAQHPPDAARQESAA-QEPQPE--SMHA

-APKR-MHSGVASSAQATEISSSIARSTYEEADG-DIE

>TraesCS7B01G111400.1

MEDQH------YRLNPTDVDAVTYYL-PRLACQTLHAKFIHHVDIYSCEPKDLPVPQATS

SGDRFFFTTRKSKNGSKTQSVRTAGGGTWTVNATTAVKH-AGVEVGERKNLSFR----KK

GKSTGWVMEEYKCLLPEAG-----VKVFCKIHLAQHPPDAARQESAA-QEPQPE--SMHA

-APKR-MHSGVASSAQATEISSSIARSTYEEADG-DIE

>TraesCS7B01G111500.1

MEDQH------YRLNPTDVDAVTYYL-PRLAGQTLHAKFIHHVDIYSCEPKDLPVPQAAS

SGDRFFFTTRKSKNGSKTQSVRTAGGGTWTVNATTAVKH-AGVEVGERKNLSFR----KK

GKSTGWVMEEYKCLLPEAG-----VKVFCKIHLAQHPPDAARQESAA-QEPQPE--SMHA

-APKR-MHSGVASSAQATEISSSIARSTSEEADG-DIE

>TraesCS7B01G115400.1

IADHQPGLPKGVKFDPSDQELLGH-LLAKHKAHPF-IEFIPTVGICYTHPQKLPGVKQDG

-SVSHFFHRTFKAYNTGTRKRRKINTSRWHKTGKTKPVMIDGQHLGCKKIMVLYMSTGGK

PKKTNWVIHQYHLGTGED---QNGEYVVSKLFFQQQNGEKS--AL--NTDVMET--AEED

-------GDNPPSQ--DPNLWAQLPVEDL-----KDLQ

>TraesCS7B01G116200.1

MEDHH------FRLNPTEVEAVTYYL-PRLSGETLHAKLIHHVNISGCEPKDLPVPQAVS

SGDRFFFTTCKSKNGSKLQSVRGAGGGTWSIQKTTEICH-AGCKVGEVKNLSFK----KK

GKSTGWVMEEYRCLLPEAG-----VKVFCKMHLAQHAPDAARQESEA-QQQQPE--STHA

-APKR-MRSDV---AQAPEISSSIARSTSEEEDG-DLE

>TraesCS7B01G116300.1

MEDHH------YRLNPTEVEAVTYYL-PRLSGETLHAKLIHSVEISGCEPKDLPAPQAVS

SGDRFFFTTCKSKNGSKLQSVRGAGGGTWSIQKTTEICH-AGCKVGEVKNLSFK----KQ

GKSTGWVMEEYRCLLPEAG-----VKVFCKMHLAQHAPDAARQESQA-QQQQPE--SAHA

-APKR-M-------AAAPV--DDMVRFCCTI----DEL

>TraesCS7B01G116400.1

MEDHH------YRLSPTEVEAVTYYL-PRLSGETLHVKLINRVEISGCESKDLPVPQAVS

SGDRFFFTTCKSKNGSKLQSVRGAGGGTWSIQKTTEIIH-AGCKVGEVKNLSFK----KK

GKSTGWVMEEYRCLLPEAG-----VKVFCKMHLAQHAPDAARQESAA-REAQTE--STHA

-APKR-MRSDV---AQAPEISSSIARSSSEEEDG-DLE

>TraesCS7B01G160900.1

MEEVLRQLPPGFRFRPTDEELVVQYLRRKALALPLPAAVISDVNLYSLDPWDIP-----G

EGEKYYFAVRPAGAKRGGR--TTTASGCWKASARERPVVVSRHLVGVKKFLAFVPRRGPA

PAQIGWVMHEYRLALPHHAEAGTQEWVVCRIFQRDRSSSSFNRQTPGGTDAHRTM-SSSS

SS------------------------------------

>TraesCS7B01G187600.1

M--ELKALPLGFRFHPTDEELVRHYLKGKITGQINEVEVIPEIDVCKCEPWDLPDKALIR

DPEWFFFAPKDRKYPNGSRSNRATEAGYWKATGKDRVIKSKGHMIGMKKTLVFHRGRAPK

GERTGWIMHEYRTTEPEFESGEQGGYVLYRLFQKQLEKTERDRSGYSNEEAITLINGNSG

GSPNSQYSADVDDSKSVPSNCSSSTDSSFTTNEGVDTD

>TraesCS7B01G196900.1

M-DDPTSLAPGFRFHPTDEELVSYYLKRKVLGRPLKVDAIAEVDLYKVEPWDLPARSRLR

DSQWYFFSRLDRKHANRARTNRATAGGYWKTTGKDREVRHGARVVGMKKTLVFHAGRAPK

GERTNWVMHEYRLEGDGAAGIPQDSFVVCRIFQKAGPGPQN--AQYAVEEEEADED----

PGPSDQY---LPHSSVKPEDDVQQSAGSSSHEAGPDSQ

>TraesCS7B01G205600.1

M--MEARMPPGFRFHPRDEELVLDYLLHKLTGRRYGGVDIVDVDLNKCEPWDLPEAACVG

GREWYFFSLRDRKYATGQRTNRATRSGYWKATGKDRAILAHGALVGMRKTLVFYQGRAPK

GTRTEWVMHEFRLEEERHRCQLKEDWVLCRVFYKSRTTSPRPPSEEAC-TFFSELGSGTA

RGPK---SNSNSKLELTPNWS-----------NG----

>TraesCS7B01G219400.1

M-EHVETVMPGFRFHPTEEELIEFYLRRKVDGKRFNIDLIASVDLYRYDPWDLPALASIG

DKEWFFYVPRDRKYRNGDRPNRVTPSGYWKATGADRMVKVVERSIGLKKTLVFYVGKAPK

GLRSSWIMNEYRLPHGET-ERYQKEISLCRVYKRPGIDDNFHLTGTTGSRAAATRHSSSA

AAP----TTSTEEDHNNPAVMLNTNSSAMAT---IPIE

>TraesCS7B01G226600.1

M-SSCNTVPPGFRFHPTEEELVGYYLARKVSSHKIDLDIIQEVDLYRIEPWDLQERCGKG

TTEYYFFSYKDRKYPSGTRTNRATAAGFWKATGRDKPVLSSSSVIGMRKTLVFYRGRAPN

GRKTDWIIHEYRLQSNEHAPTQEEGWVVCRAFVKPVPNQQHRLSYGGASDN-----AGGG

--PHDQHPSDLPPPLQSPTFDGGCSSADQQLAAGIDWN

>TraesCS7B01G233300.1

M-GGALSVPPGFRFHPTDEELLYYYLRKKVAYEAIDLDVIREIDLNKLEPWDLKDRCRIG

QNEWYFFSHKDKKYPTGTRTNRATTAGFWKATGRDKAIFLGSRRIGMRKTLVFYVGRAPH

GKKTDWIMHEYRLDED--VEVQEDGWVVCRVFTKKSYTRGMNPAGMADDDELLHPFSAAG

HSPHLQHSMQLPQLSFLPGA-KLTSSGTNGMFPGTDWS

>TraesCS7B01G246300.1

M-GGGGELPPGFRFHPTDEELITYYLLRKVVDCGFSARAIAEIDLNKCEPWELPDKACK-

EKEWYFYSLRDRKYPTGLRTNRATGAGYWKATGKDREIRSARALVGMKKTLVFYRGRAPK

GQKTQWVMHEFRLEGVYANNTTRDEWVIAKIFVKPGAVPPSRKARYSSADTSCFSDASSA

SSPFSQYGSRLVQAMFLPGGLLGMGGGTFQHPSGYDIK

>TraesCS7B01G252100.1

M-HNRHGFPQGYHFVPSDLELIRL-LEDIIAGRPLPLTIFRNVRIRDYHPAELYERYKA-

AGSIYFFSE--REFP-GGRPRRFTKDGWWKASGGGESLRRRGLTVGSKLTLVFYDKKPKV

GVKTDWAIKEYTKIVDKK---KAEEMALYRLYKMNK-------PANAQEDENTPNSGQTH

AGPAVEH---PPAAAVSPSSPQVAASSSSPTFAGEDIP

>TraesCS7B01G270300.1

TA-LDVGLPAGVKFDPTDQELIEH-LEAKVSGHPL-IEFIPTIGICYTHPEKLPGVSKDG

-LSRHFFHRPSKAYTTGTRKRRKIQPPRWHKTGKTRPVAVGGRQRGCKKILVLYTNFHRK

PEKTNWVMHQYHLGESEE---REGELVVSKIFYQTQPRQCG--V------GDGASASAGT

TV---HHQVSTPTDI---PVHSRRSKGGETS--GEGTE

>TraesCS7B01G364600.1

M--VEARLPPGFRFHPRDDELVLDYLTRKLGGGAYGCPTMVDVDLNKIEPWDLPEIACIG

GKEWYFYSLRDRKYATGQRTNRATESGYWKATGKDRSISRKGLLVGMRKTLVFYQGRAPK

GKKTEWVMHEFRKEGQGDKLPLKEDWVLCRVFYKTRATVAKPPTGSSYNDSAAATSSTVQ

SSPAA--LTRFEQGREAPAQG----------QDGFEYL

>TraesCS7B01G365300.1

M--VEARLPPGFRFHPRDDELVLDYLSRKLGGGAYGCPAMVDVDLNKIEPWDLPEIACIG

GKEWYFYSLRDRKYATGQRTNRATESGYWKATGKDRAISRKGLLVGMRKTLVFYEGRAPK

GKKTEWVMHEFRKEGQGDKLPLKEDWVLCRVFYKTRTTIAKPPTGSSYNGSAAATSSTVQ

SSPAA--LTRFEQGREAPAQG----------QDGFEYL

>TraesCS7B01G461700.1

MA-SEQQIPT-PQLQPGDDRNLVSILRRRVAAEPFAASFIQEADVYLAAPAELPGMD--G

ETAWYFFSPANYHETPGARRRRKVGAGCWHPEAGKVPILGPGKPVGAKRKLSYVSAKPYR

NKSLGWIMVEIGLEQQQA----DQQLVLCKLYKSRSRRDAEDEAAAASQERKTTTSASPA

ASPLTQANTQMSQLTISPVLSTTAASSSVDKEVGKDIE

>TraesCS7B01G472200.1

ME-----LLPGFRFHPTEEELLEFYLKHHVTSSSAPFDIIPTVHLYRHDPWDLPGLAAIS

EREWYFFVPRDRKHAAGGRPSRTTERGFWKATGSDRAVRCAARLVGLKKTLVYYQGRAPR

GTKTDWVMNEYRLPDLAGGAGEQQDVVLCKVYRKAVSLKELEQRVA-MEELARARSGSPD

EA---HH-------VARPEAM-----------------

>TraesCS7B01G472600.1

ME-----LLPGFRFHPTEEELLEFYLKHHVTSNSAPFDIIPTVHLYRHDPWDLPGLAAIS

EREWYFLVPRDRKHAAGGRPSRTTERGFWKATGSDRAVRCAARLVGLKKTLVYYQGRAPR

GTKTDWVMNEYRLPDLAGGAGEQQDVVLCKVYRKAVSLKELEQRVA-MEELARARSGSPD

EA---HH-------VARPEAM-----------------

>TraesCS7B01G481400.1

M-EEEDSL-------PGEHTLVAV-LRRHATG--LP-AWVHEVNVYAASPVLLK-IAEDG

STAWFFLYTPARKEGSSSRCSRKAGGATWIEERTRPV--KAGVVIGHASTFTYGKGPKRR

TERLGWILVEVRLP----G----ETMCCAKLYRSPRKTSAAAPAAPSAANA-----AAPE

AAPLA--RSPAPAAPPAPADSTKLSDKGMSKEGG----

>TraesCS7B01G489500.2

M-QKQLVLPPGFRFHPTDEEIIKFYVVPKVLDEAFVAAAIEDVNLNKYEPWELPEKVKMG

EKEWYFYSRKDRKYPTGIRTNRATEAGYWKATGKDKEIFHPPTLIGMKKTLVFYKGRAPR

GEKTNWIMHEYRLESSKKNMASKEQWVVCRIFHKSTRLKKMVTLSYDAEHQQGFVDSTCV

GAPMNHHHMGADQGEAQPESGAATAVGETSPNMGMDDI

>TraesCS7B01G499400.1

MAEPQLRFPAGYHFRPTDEELLDVYLRAKIDGREPPLDVFMDVDILDWDPAELVEKRKAG

-EGRYFFTKRTEPAKNGER--RKLVKASWKATGCPGIIYRSAEKIGTKRILTYYSGGV--

-ECDKWSMNEYVMTGRAG----LDQWILCTIQEKLH-EAKSSKHAAGAEDTSSKGKSQPH

TSPETQH-------GQEPTPGNGMEHASGCQTPGGDFK

>TraesCS7D01G000200.1

MTAAAAVLPVGFRFRPTDEELVRHYLKAKIAGRAPDLLAIPDVDLAAVEPWDLPARSVIK

DPEWFFFARRDRKYPKSSRSCRSTAAGYWKATGKDRLIRAPGALIGVKKTLVFHRGRAPR

GARTPWIMHEYTATDPNPAGPQNDSFVLYRLFNKQDEETPAPISGPPSTSPPA---ASMV

TAPDIQEQQGIAARASQPVLAAGCSTESSSSMAGHNMA

>TraesCS7D01G008500.1

M-NTQPQVPPGFRFHPTDEELVDYYLRKKVASRRIDLNVIKDVDLYKIEPWDLQEKCRIG

QSDWYFFSHKDKKYPTGTRTNRATAAGFWKATGRDKPIYAKHCLVGMRKTLVYYKGRAPN

GQKSDWIMHEYRLETNENGPPQEEGWVVCRVFKKRLPTTRRDLDHDAIDDDGPFM-SPMR

SM---QYLNTIPHELESPSSFVSPDDHQINVQAATDWR

>TraesCS7D01G034000.1

-------METGFVFSPADHELTDLYLGGQIAGHPVFSTFIHHADVYSAAPAELHV-----

------------RDRDGNKKSKLRSKSYWHSESGKKPV---EAVGGYVQDFVYAIKKDGH

VERLGWRMKEYGLSTEHGG-----DFLE--------------------------------

--------------------------------------

>TraesCS7D01G062100.1

MSNGQSVVPPGFRFHPTEEELLTYYLAKKVASQRIDLDVIPDVDLNKLEPWDIQERCRIG

QNDWYLFSHKDKKYPTGTRTNRATAAGFWKATGRDKAIYSAAGRIGMRKTLVFYKGRAPH

GHKSDWIMHEYRLDDAVPSSAQEDGWVICRVFKKKNILVNQ--GQNGASSKLAGAASQSN

SS-QQLHTTPCKQE-TKPA--TACPNG--TLEYGTDWD

>TraesCS7D01G062300.1

MSNGQSVVPPGFRFHPTEEELLTYYLAKKVASQRIDLDVIRDVDLNKLEPWDIQERCRIG

QNDWYLFSHKDKKYPTGTRTNRATAAGFWKATGRDKAIYSAAGRIGMRKTLVFYKGRAPH

GHKSDWIMHEYRLDDAATSSAQEDGWVICRLFKKKNIAVQHQAGQNGASNNLVGAGSRSN

SS-IQQHTTSCKQE-T--TN--AGHNGGSTLEHSGDWD

>TraesCS7D01G062400.1

MSNGQSVVPPGFRFHPTEEELLTYYLAKKVASQRIDLDVIPDVDLNKLEPWDIQERCRIG

QNDWYLFSHRDKKYPTGTRTNRATAAGFWKATGRDKAIYSAAGRIGMRKTLVFYKGRAPH

GHKSDWIMHEYRLDDAV-SSTQEDGWVICRVFKKKNIVVQRQAGQNGASNQLVGAGSQSN

SS--QQHTASCEQE-TKPTN--ACHNGSSTLEYGGDWD

>TraesCS7D01G062600.1

MSNGQSVVPPGFRFHPTEEELLTYYLAKKVASQRIDLDVIPDVDLNKLEPWDIQARCRIG

QNDWYLFSHKDKKYPTGTRTNRATAAGFWKATGRDKPIYSAAGRIGMRKTLVFYKGRAPH

GHKSDWIMHEYRLDDAVASLAQEDGWVICRVF-KKNIVAQHQAGQSGASNKLVGAGSQSN

SS---MHAASCKQE-TKPTN--ACHSGSSTLEYGGDWD

>TraesCS7D01G063300.1

MSNGQSVVPPGFRFHPTEEELLTYYLVKKVASQRIALGVIPDIDLNKLEPWDIQARCRIG

QNDWYLFSHKDKKYPTGTRTNRATAAGFWKATGRDKPIYSAAGRIGMRKTLVFYKGRAPR

GHKSDWIMHEYRLDDAV-SSAQEDGWVICRVFKKKNIIVQRQAGQNGASNKLVGAGSRSN

SS---MPTSSCRHQ-TKPAN--ACHSSSSTLEYGGDWD

>TraesCS7D01G100400.1

MERLVLNLPPGYHFAPTDMELIVHYLRRKMDGHPPHLPIFKDVPITDYRPEQITEVFDCG

EERWYFFTKRTRKYATGNRPDRTTGRGYWKATGPQRLIRTGPPLVGRRRTLVFYTGP-DE

AAKTAWTMYEYENLTSEENADKLGEWVLCTIQRQKNQRSADKG--KAKGKAQKGAD----

ASPLMQHYSTTPYQ---PATS----------YAG----

>TraesCS7D01G154200.1

M-RQQLKFPQGFRFHPTDVEIITSYLVPKVLNKAFDPIAVGEVDLNKCEPWELPEKAKMG

EKEWYFFSQKDRKYPTGIRTNRATTAGYWKATGKDKEIFHHASLIGMKKTLVFYKGRAPR

GDKTNWVMHEYRLESGKQNASSKEEYVVCRIFHKSTGLKKVVMSSYAAEEQHGFLESSLV

GAPMNHHQIGADEGGVEPGSGVATTADEISSNMGTDGM

>TraesCS7D01G174400.1

MADPALALPPGVYFNPMNEEIVRTYLNGWIAGHGTEAAVVIEEDVYGDKPDVLPASTRDS

DPSWWFRCHC-KQATRGGRGDRSVTGGFWKLEQTTKEVRCEAEHLGFKRTFGFYVEHEGE

KEKTRWLMEEYTNVDSPDG---KNLPALYRIYLTPRDPGKKKRKQSGGDDYDD---AARV

SSP--EY-------DDSPEMRVTMNDGGIPAEMG----

>TraesCS7D01G189900.1

M-QQQLNLPPGFRFQPTDMEIITFYLVPKVLKKVFNTTVVEEVDLNKCEPWDLLKKVNMG

DKGRYFFSQKDLKYSIGIRTNGATKAGYWKATGKDKEIIHPPSIIGMKKTLVFYKGRAPK

GEKTNWIMHEYRLESGKQNASSKGEYVVCRIFHKSTGLKKVMMPSYDGEKQQGFLDSSLA

EAPMNHYEMSVDQGGVDPGSV-----------------

>TraesCS7D01G196300.1

M-QQHLNLPAGFRFHPTDVEIITFYLVHKVLKKPFDVIVIEEVDLNKCEPWDLPNNVNMG

EKDQYFFSKKDLKYPTGVRTNRATNAGYWKATGKDKEIVHPPSLVGMKKTLVFYKGRAPR

GEKTNWIMHEYRLEMGKQNATSKEEYVVCRIFHKSTGLKKVVMPSYSEEQQQGFLESSLA

GAPMNHHHMSAEQDGVDPGSGGATISSEISSNMGMDGM

>TraesCS7D01G205200.1

MEEQH------FRLNPTEVEAVTYYL-PRLAGET-HAKLIHDAHVYDCEPKDLPVPQAVS

SGDRFFFTTCKRAKGRTTRCARKAGGGTWTVNTTTVVTH-AGVDVGERKNLSFK----KQ

GKSTGWVMEEYRLLPPEAE-----EKVFCKIHLSQHAPDEARRESAA-QEPVPR--PEHA

-GPKR-I-------AAAPV--DDMAWLSCTMEEA-DME

>TraesCS7D01G207400.1

MEDQH------YRLNPTDVEAVTYYL-PRLAGQ-LHAKFIHHVDIYSCEPKDLPVPQAAS

SGDRFFLTTRKSKNGSKTQSVRTAAGGTWTVNATTTVRH-AGVEVGERKNLSFR----KK

GKSTGWVMEEYKCLLPEAG-----VKVFCKIHLAQHPPDAARQESAA-QEPQRE--STHA

-APKR-MHSGVASSAQAPEISSSIARSTSEEADG-DIE

>TraesCS7D01G210600.1

IADHQPGLPKGVKFDPSDQELLGH-LLAKHRAHPF-IEFIPTVGICYTHPQKLPGVKQDG

-SVSHFFHRTFKAYNTGTRKRRKINTSRWHKTGKTKPVMVDGQHLGCKKIMVLYMSTGGK

PKKTNWVMHQYHLGTGED---QNGEYVVSKLFFQQQNGQKS--AL--NTDVMET--AEEE

-------GDNPPSQ--DPNLWAQLPVEDL-----KDLQ

>TraesCS7D01G210900.1

MEDHH------YRLCPTEVEAVTYYL-PRLSGETLHAKLIHRVEISGCEPKDLPAPQAVS

SGDRFFFTTCKSKSGSKLQSVRGAGAGTWSIQKTTEICH-AGVKVGEVKNLSFK----KK

GKSTGWVMEEYRCLLPEAG-----VKVFCKMHLAQHAPDAARQESEA-QQQQPE--STHA

-APKR-MRSDV---AQAPEISSSIARSTSEEEDG-DLE

>TraesCS7D01G211300.1

MEDHH------YRLSPTEVEAVTYYL-PRLSGETLHAKLIHRVEISGCEPKDLPAPQAVS

SGDRFFFTTCKSKNGSKLQSVRGAGSGTWSIQKTTEICH-AGCKVGEIKNLSFK----KK

GKSTGWVMEEYRCLLPEAG-----VKVFCKMHLAQHAPDAARQESEA-QQQQRE--LE--

--------------------SSSIARSSSEEADG-DIE

>TraesCS7D01G211400.1

MEDHH------YRLSPTEVEAVTYYL-PRLSGQTLHAKLIHRVEISGCEPKDLPAPQAVS

SGDRFFFTTCKSKNGSRLQSVRGAGAGTWTIQKTTEICH-AGVKVGEVKNLSFK----KK

GKSTGWVMEEYRCLLPEAG-----VKVFCKMHLAQHAPDAARQESEA-QQQQPE--STHA

-AHKR-M-------AAAPV--DDTVRFCCTI----DEL

>TraesCS7D01G246100.1

M-AGEHGLPPGFRFHPTDEELVTFYLAAKVFNGACCGGDIAEVDLNRCEPWDLPEAARMG

EREWYFFSLRDRKYPTGLRTNRATGAGYWKATGKDREVLNAASLLGMKKTLVFYRGRAPR

GEKTKWVLHEYRLDGDFGRRSCKEEWVVCRIFHKAVDPYSKMMEMRNSPHHSFFQDQSQG

SGPAAAY-------PQEPPPT----------EMG----

>TraesCS7D01G263800.1

MEEVLRQLPPGFRFRPTDEELVVQYLRRKALALPLPAAVISDVNLYSLDPWDIP-----G

EGEKYYFAVRPAGAKRGGR--TTTASGCWKASARERPVVVSRHLVGVKKSLAFVPRRGPA

SAQTGWVMHEYRLALPHHAEAGTEEWVVCRIFQRDRSSSSSNRQTPGGTDAHHTM-SSSS

SS------------------------------------

>TraesCS7D01G295100.1

M--ELKALPLGFRFHPTDEELVRHYLKGKITGQINEVEVIPEIDVCKCEPWDLPDKALIR

DPEWFFFAPKDRKYPNGSRSNRATEAGYWKATGKDRVIKSKGHMIGMKKTLVFHRGRAPK

GERTGWIMHEYRTTEPEFESGEQGGYVLYRLFQKQLEKTERDRSGYSNEEAITLINGNSG

GSPNSQYSADVDDSKSAPSDCSTSTDSSFTTNKGVDTN

>TraesCS7D01G302000.1

M--MEARMPPGFRFHPRDEELVLDYLLHKLTGRRYGGVDIVDVDLNKCEPWDLPEAACVG

GREWYFFSLRDRKYATGQRTNRATRSGYWKATGKDRAILAHGALVGMRKTLVFYQGRAPK

GTRTEWVMHEFRLEEERHRCQLKEDWVLCRVFYKSRTTSPRPPSDEAC-TFFSELDSGTA

RGPK---SNSNSKLELTPNWS-----------NG----

>TraesCS7D01G314000.2

MSNGQSCVPPGFRFHPTEEELLNYYLRKKVASEEIDLDVIRDVDLNKLEPWDIQEKCKIG

QNDWYFFSHKDKKYPTGTRTNRATAAGFWKATGRDKAIYNAVKRIGMRKTLVFYKGRAPH

GQKSDWIMHEYRLDDPSSDAGQEDGWVVCRVFKKKHHHKDAGAGSGKRDDG-----GKAA

SSP--LY---CKQESPRPRT-----------RDGTDWA

>TraesCS7D01G315100.1

M-EHVETVMPGFRFHPTEEELIEFYLRRKVDGKRFNIDLIASVDLYRYDPWDLPALASIG

DKEWFFYVPRDRKYRNGDRPNRVTPSGYWKATGADRMVKVVERSIGLKKTLVFYVGKAPK

GLRSSWIMNEYRLPHGET-ERYQKEISLCRVYKRPGIDDNFHLTGTTGSRAAATRHSSSA

AAP----TTSTEEDHNNPAVMLNTNSSAMAT---IPID

>TraesCS7D01G329200.1

M-GGALSVPPGFRFHPTDEELLYYYLRKKVAYEAIDLDVIREIDLNKLEPWDLKDRCRIG

QNEWYFFSHKDKKYPTGTRTNRATTAGFWKATGRDKAIFLGSRRIGMRKTLVFYVGRAPH

GKKTDWIMHEYRLDED--VEVQEDGWVVCRVFTKKSYTRGVNPAEMADDDELLHPFSTAG

HNPHLQHSMQLPQLSFLPGA-KLTSSGANGMFPGTDWS

>TraesCS7D01G342300.1

M-GGGGDLPPGFRFHPTDEELITYYLLRKVVDCGFSARAIAEIDLNKCEPWELPDKACK-

EKEWYFYSLRDRKYPTGLRTNRATGAGYWKATGKDREIRSARALVGMKKTLVFYRGRAPK

GQKTQWVMHEFRLEGVYANNTTRDEWVIAKIFVKPGAVPPSRKVRYSSADTSCFSDASSA

SSPFSQYGSRLVQAMFLPGGLLGMGGGAFQHSSGYDMK

>TraesCS7D01G348100.1

M-DNRHGFPQGYHFVPSDLELIRL-LEDIIAGRPLPLTIFRNVRIREYHPAELYERYKA-

AGSIYFFSE--REFP-GGRPRRFTKDGWWKASGGGEALKRGGLTVGSKLTLVFYDKKPKV

GVKTDWAIKEYTKIVDKK---KAEEMALYRLYKMNK-------PANAQEDENTPDSGQPH

AGPAVEHPLPAPAAAVSPSSPQVAASSSSPMFVDEDIP

>TraesCS7D01G365200.1

TA-LDVGLPAGVKFDPTDQELIEH-LEAKVSGHPL-IEFIPTIGICYTHPEKLPGVSKDG

-LSRHFFHRPSKAYTTGTRKRRKIQPPRWHKTGKTRPVAVGGRQRGCKKILVLYTNFHRK

PEKTNWVMHQYHLGESEE---REGELVVSKIFYQTQPRQCG--I------GDGASASAGT

TV---HHHVSTPTDIAAPPVHSRRSKGGETS--GEGTE

>TraesCS7D01G371800.1

MADNRHGFPHGYHFVPDDPELLRL-LEDMIAGRALPLSIFHGVRIRNYHPAELHELYKA-

AGSIYFFNER--EFS-GSRPGRTAKDGWWKASGGGLPLVRRGLVVGYKLTLVFYEKRPKP

DVKTDWIVKEYTIVG---PNKKVSEMALYRLYNKSENKSKEKKKAKEHEEENTPASGQPH

AGPSSQHPPPAPAVASSPAFRQVAASPAFHMFSVKDIP

>TraesCS7D01G451700.1

M--VEARLPPGFRFHPRDDELVLDYLAKKLGGGGYGCPTMVDVDLNKIEPWDLPEIAFIG

GKEWYFYSLRDRKYATGQRTNRATESGYWKATGKDRSISRKGLLVGMRKTLVFYQGRAPK

GKKTEWVMHEFRKEGQGDKLPLKEDWVLCRVFYKTRATVAKPPTGSSYNDSAAATSSTVQ

SSPAG--LTRFEQGREAPAQG----------QDGFEYL

>TraesCS7D01G452500.1

M--VEARLPPGFRFHPLDDELVLDYLSRKLGGGAYGCPAMVDVDLNKIEPWDLPEIACIG

GKEWYFYSLRDRKYATGQRTNRATESGYWKATGKDRAISRKGLLVGMRKTLVFYEGRAPK

GKKTEWVMHEFRKEGQGDKLPLKEDWVLCRVFYKTRTTIAKAPTGSSYNDSAAATSSTVQ

SSPAA--LTRFEQGREAPAQG----------QDGFEYL

>TraesCS7D01G535300.1

ME-----LLPGFRFHPTEEELLEFYLKHHVTRNNAPFDIIPTVHLYRHDPWDLPGLAAIS

EREWYFFVPRDRKHASGGRPSRTTERGFWKATGSDRAVRCAARLVGLKKTLVYYQGRAPR

GTKTDWVMNEYRLPDLSGGAGEQQDVVLCKVYRKAVSLKELEQRVA-MEELARARSGSPD

EA---HH-------VARPPAM-----------------

>TraesCS7D01G543400.1

M-QQKLELPLGFRFHPTDEEIINSYVIPKVLDEAFVTAAIEDVNLNKYEPWELPEKAKMG

EKEWYFYSRKDRKYPTGIRTNRATGVGYWKATGKDKEIFHPPTLIGMKKTLIFYKGRAPT

GEKTNWIMHEYRLESNKEKAASKEQWVVCRIFHKSTGLKKVVMPSYVAEHQQGLTDSSLA

GAPMNHRYMGEDQSGAKPGCGAATAGGKTSSNMSMDDM

>TraesCS7D01G543500.2

M-QKQLVLPPGFRFHPTDEEIIKFYVVPKVLDEAFVVAAIEDVNLNKYEPWELPEKAKMG

EKEWYFYSRKDRKYPTGIRTNRATEAGYWKATGKDKEIFQPPTLIGMKKTLVFYKGRAPR

GEKTNWIMHEYRLESNKKNTASKEQWVVCRIFHKSTGLKKMVTPSYNAEHQQAFVDSTCA

GTPMNHHHMGADQGGAQPGSGATTAVGETSLNMGMDDI

>TraesCSU01G119900.1

MA-SSSVLPMGCRFSPSDADLICAYLRPMIASEPLPARFLHTADAYAADPAALPALAKTG

RRCWYFFGSAKALSGHNKHRSRAVGEGTWHAEKGRKAMLDGEGVVGYKQSFRYKPIHADG

SVEAVWLMVEFRMAHDQGG---ETVPVLCKVYQSPRKPRSASVTSPAGRKRKAGDDSAPV

EPPPEIH---MPFHPVVPESE-TVVHGGSVTYAGGDTT

>TraesCSU01G120000.1

MA-SSSILPQGCRFSPSDADLISFYLRPMIASEPLPARFLHSADAYAADPAALPALAKTG

RRCWYFFGSAKALSGHDKRRSRAVGEGTWHAEKGRAAVLDGERVVGYKQSFRYKPVHADG

SVEAVWLMVEFRMAHDQGG---ESVPVLCKVYQSPRKPRSASVTSPAERKRKARDDSAPV

EPPPEN--------PVVPESQ-TVLHGGSVTYAGGDTT

>TraesCSU01G137200.2

M--EMPPLPPGYRFHPTDVELTLYYLKRKLLGKKLLCNAVAEVDIYKHAPWDLPAKSSMG

DLQWYFFCTRGRKYSVGQRANRSTEGGYWKATGKDRQVVYENRTVGMKRTLVFHAGKAPK

GTRTDWVMYEYRLVQGEIAGVRLDDSVLCKVHKKSGPGPKIG-EQYAEEEE-----GNAS

PGPRAEQ---VPSSLLEPAAGGQVADGSSSQSNGLDLE

>TraesCSU01G163300.1

MS---LALEPGFRFHPTGQELVTYDLLRKVHGHH---AFIPEVHVYKHEPWELPGTSPTG

KVEWYFFAVRARKYPNGLRMGRATVAGFWKSTGKDRPVMHNGVIVGMKKTLVFHTGRAPG

GTRTDWVMHEYRLQGQRN-HHIQDAYALCRVFKKNTVAPLIDLSSDAYEDPMQSVPADSH

KDP--KH---LIQSHNDPILVAYAGKEQMESHAG----

>TraesCSU01G174800.1

MEDHH------YRLSPTEVDAVTYYL-PRLSGETLHVKLIHRVEISGCEPKDLPVPQAVS

SGDRFFFTTCKSKNGSKLQSVRGAGGGTWTIQKTTEICH-AGVKVGEVKNLSFK----KK

GKSTGWVMEEYRCLLPEAG-----VKVFCKMHLTQHAPDAARQESEA-QQQQPE--STHA

-APKR-MRSDV---AQAPEISSSIARSTTEKEYG-DLE

>TraesCSU01G230600.1

MEDHH------YRLSPTEVDAVTYYL-PRLSGETLHVKLIHRANISGCEPKDLPVPQAVS

SGDRFFFTTCKSKNGSKHQSLRGAGTGTWTIQKTTEICH-AGVKVGEVKNLSFK----KK

GKSTGWVMEEYRCLLPEAG-----VKVFCKMHLAQHAPDAARQESEA-QQPQPE--STHA

-APKR-M-------AAAPV--DDMARFCCTI----DEL
