# Supplementary material for: Comprehensive analysis of pathogen-responsive wheat NAC transcription factors: new candidates for crop improvement
Source: G3 (Bethesda). 2022 Sep 21;12(11):jkac247. doi: 10.1093/g3journal/jkac247 (PMC9635653; doi:10.1093/g3journal/jkac247)
Supplement: jkac247_Supplemental_Results [file jkac247_supplemental_results.pdf]

Supplemental Results

Assessment of the role of the fungal-responsive *TaNACs* in bacterial disease

A study was performed to determine how many of the fungal-responsive *TaNACs* were involved in the response to a bacterial disease (bacterial leaf streak disease caused by *Xanthomonas translucens*). This study also determined if any of the additional bacterial-responsive *TaNACs* identified through this analysis would cluster into the subclades determined in the current study as being enriched with fungal-responsive *TaNAC*s. Analysis of the study on wheat leaves infected with *Xanthomonas translucens* (Garcia-Seco et al., 2017) (File S5) revealed 51 pathogen-responsive *TaNAC*s. 39 (76%) of those were already determined as being responsive to five fungal pathogens (Table 1). A higher proportion of *Xanthomonas*-responsive *TaNACs* (43%) were responsive to only hemibiotrophic fungi than to both hemibiotrophic and biotrophic fungi (27%), while 6% were responsive to only biotrophic fungi. 36 (92%) of those responsive to fungi clustered in subclades enriched with pathogen-responsive *TaNAC*s 12 (24%) were specific for *X. translucens,* and four of the 12 specific for *X. translucens* were placed in subclades enriched with pathogen-responsive *TaNAC*s (*TaNAC016-B1 [TraesCS3B02G093300]* in 'a2', *TaNAC040-D1 [TraesCS7D02G283800]* in 'b6', *TaNAC015-B3-1 [TraesCS2B02G118200]* in 'd5', *TaNAC104-B1 [TraesCS2B02G4841000]* in 'd1.2').

Validation of the responsiveness of select *TaNAC* genes to *Fusarium graminearum, F. pseudograminearum and P. striiformis*

An *in-silico* study was conducted to validate the pathogen-responsiveness of the *TaNACs* using data extracted from publicly available RNA-seq studies that were both independent to, and at comparable time points to, those used in the initial analysis. *TaNACs* selected for study were the 5 most responsive to *F. graminearum* and *P. striiformis,* and the two *TaNACs* that were responsive to *F. pseudograminearum;* no public datasets at comparable time points were available for *B. graminis* and *Z. tritici.* Transcript per million (tpm) data for the select genes were extracted from the RNA-seq studies on wheat infected with either *F. graminearum, F. pseudograminearum* or *P. striiformis* (Kugler et al., 2013; Dobon et al., 2016; Powell et al., 2017; Table 1). The Wheat expression browser database (http://www.wheat-expression.com/) contained tpm values for the pathogen-responsive genes from the RefSeq v1.1 wheat genome annotation. We also searched the literature to extract tpm values or differential expression data

on for these genes from other datasets; however, the data were either not available or did not contain information about the gene that could be converted to the RefSeq v1.1 genome annotation. The five *TaNACs* induced by *F. graminearum* at 48 hpi in NIL 51 were also significantly induced by the pathogen at 50 hpi in cv. CM-82036 (Table S11). Also, the five *TaNACs* induced by *P. striiformis* in the wheat line N9134 at 1 dpi were also significantly induced by the fungus at the same timepoint in cv. Vuka (Table S11). The two *TaNACs* differentially expressed upon infection with *F. pseudograminearum* in resistant NIL1 at 3 dpi, were also significantly induced by the pathogen in cv. Chara at 3 dpi (Table S11). Hence this analysis validated that candidate *TaNACs* were pathogen-responsive in independent studies.

Comparison of pathogen-responsive *TaNACs* across studies

A study was done to compare *TaNACs* responsive to *Blumeria graminis* and/or *Puccinia striiformis* from the current study with the ones delineated in studies from Lv et al. (2020) and Ma et al. (2021). Herein, we identified 45 and 46 *TaNACs* that were responsive to *B. graminis* and *P. striiformis*, respectively, based on the RNA-seq study (E-MTAB-4289; Zhang et al., 2014). From the same RNA-seq dataset, Lv et al. (2020) identified 41 *TaNAC* unigenes responsive to *B. graminis* and 41 *TaNAC* unigenes responsive to *P. striiformis*. But it lacked information on the RefSeq gene annotation, thus these could not be compared with the *TaNACs* responsive to *B. graminis* and *P. striiformis* identified herein. However, Lv et al. (2020) provided RefSeq gene annotation of the 25 genes responsive to both *P. striiformis* and *B. graminis* that were validated by RT-qPCR, which were then cross-compared with the *TaNACs* identified to be responsive to the same pathogens herein. Of the 25 genes responsive to *B. graminis* and *P. striiformis*, six and eight were also responsive to *B. graminis* and *P. striiformis* in this study, respectively (Tables S12 and S13). All these common genes except *B. graminis*-responsive *TaNAC031-A2* were located in subclades enriched in pathogen-responsive *TaNACs* ('f', 'e1.1', 'a1', 'a2', 'd1.2', 'd5', 'b6') and all these genes but *TaNAC031-A2* had a universal pathogen response (responsive to at least two different pathogens) (Tables S12 and S13; File S2). Similarly, Ma et al. (2021) identified 47 and 51 differentially expressed *TaNACs* in response to *B. graminis* and *P. striiformis,* respectively. Of the 47 and 51 *TaNACs* responsive to *B. graminis* and *P. striiformis,* six and 11 were responsive to the same pathogens herein, respectively (Tables S12 and S13). These six and 11 genes were located in subclades enriched in pathogen-responsive *TaNACs* ('a1', 'a2', 'b5', 'b6', 'd1.2', 'd5', 'g1.1'), and all these genes, except the *B. graminis*-responsive *TaNAC036-D1,* had a universal pathogen response in our study (Tables S12 and S13; File S2).
