# Supplementary material for: Comprehensive analysis of pathogen-responsive wheat NAC transcription factors: new candidates for crop improvement
Source: G3 (Bethesda). 2022 Sep 21;12(11):jkac247. doi: 10.1093/g3journal/jkac247 (PMC9635653; doi:10.1093/g3journal/jkac247)
Supplement: jkac247_Supplemental_Figure_S4 [file jkac247_supplemental_figure_s4.pdf]

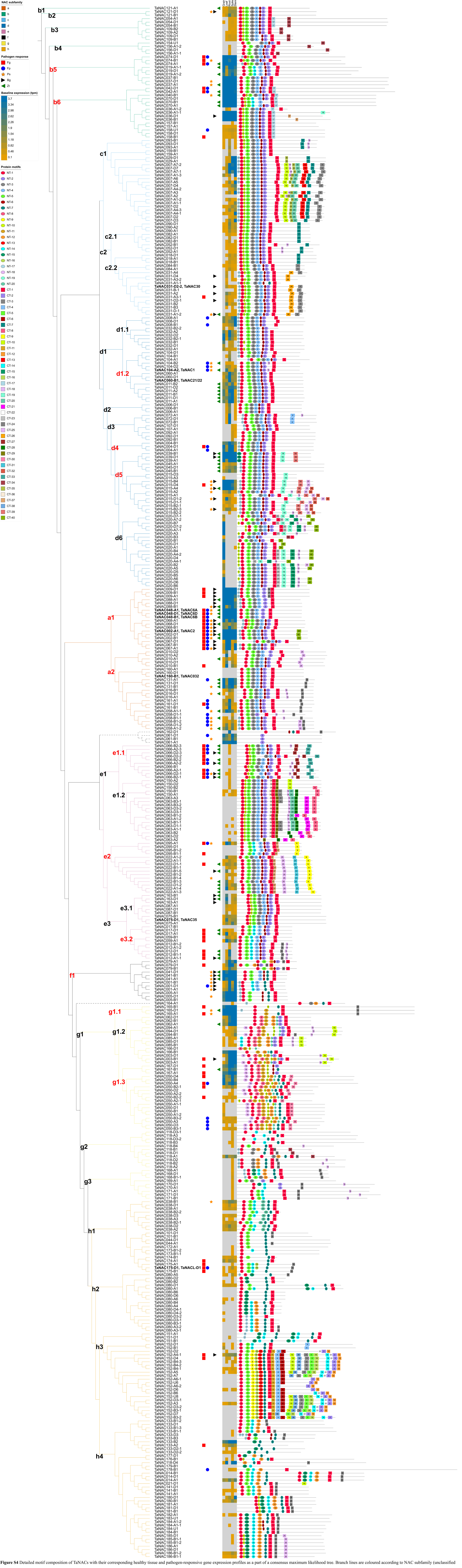

Figure S4 Detailed motif composition of TaNACs with their corresponding healthy tissue and pathogen-responsive gene expression profiles as a part of a consensus maximum likelihood tree. Branch lines are coloured according to NAC subfamily (unclassified clades and branches are indicated with a dashed line). Within a subfamily, TaNAC subclades are coded at the node based on the subfamily letter and a specific number. For example, 'e1' is the first subclade of subfamily 'e', and 'e1.1' and 'e1.2' are two distinct subclades within 'e1' subclade. Red font indicates subclades enriched with pathogen-responsive *TaNAC*s. TaNACs highlighted in bold are functionally charaterized in pathogen defense (Table 3). The coloured symbols denote the *TaNACs* that are differentially expressed upon infection with a pathogen. Pathogen abbrevations: Fg, *Fusarium graminearum*; Fp, *Fusarium pseudograminearum*; Ps, *Puccinia striifromis*; Bg, *Blumeria graminis;* Zt, *Zymoseptoria tritici.* Coloured scale represent tpm values of baseline expression in healthy tissues (grain, root, leaf, spike and stem): blue represents high expression and yellow represents low expression; gray represents no expression.
